# Nivolumab Plus Ipilimumab vs Nivolumab for Previously Treated Patients With Stage IV Squamous Cell Lung Cancer: The Lung-MAP S1400I Phase 3 Randomized Clinical Trial

**DOI:** 10.1001/jamaoncol.2021.2209

**Published:** 2021-07-15

**Authors:** Scott N. Gettinger, Mary W. Redman, Lyudmila Bazhenova, Fred R. Hirsch, Philip C. Mack, Lawrence H. Schwartz, Jeffrey D. Bradley, Thomas E. Stinchcombe, Natasha B. Leighl, Suresh S. Ramalingam, Susan S. Tavernier, Hui Yu, Joseph M. Unger, Katherine Minichiello, Louise Highleyman, Vassiliki A. Papadimitrakopoulou, Karen Kelly, David R. Gandara, Roy S. Herbst

**Affiliations:** 1 Yale Cancer Center, New Haven, Connecticut; 2 SWOG Statistical Center, Seattle, Washington; 3 Fred Hutchinson Cancer Research Center, Seattle, Washington; 4 University of California San Diego Moores Cancer Center, La Jolla; 5 Mount Sinai Health System, New York, New York; 6 University of California Davis Comprehensive Cancer Center, Sacramento; 7 Columbia University Medical College, New York, New York; 8 Washington University School of Medicine, St Louis, Missouri; 9 Duke University School of Medicine, Durham, North Carolina; 10 Princess Margaret Hospital, Toronto, Ontario, Canada; 11 Department of Hematology and Oncology, Emory University, Atlanta, Georgia; 12 Idaho State University School of Nursing, Pocatello; 13 The University of Texas MD Anderson Cancer Center, Houston

## Abstract

**Importance:**

Nivolumab plus ipilimumab is superior to platinum-based chemotherapy in treatment-naive advanced non–small cell lung cancer (NSCLC). Nivolumab is superior to docetaxel in advanced pretreated NSCLC.

**Objective:**

To determine whether the addition of ipilimumab to nivolumab improves survival in patients with advanced, pretreated, immunotherapy-naive squamous (Sq) NSCLC.

**Design, Setting, and Participants:**

The Lung Cancer Master Protocol (Lung-MAP) S1400I phase 3, open-label randomized clinical trial was conducted from December 18, 2015, to April 23, 2018, randomizing patients in a 1:1 ratio to nivolumab alone or combined with ipilimumab. The median follow-up in surviving patients was 29.5 months. The trial was conducted through the National Clinical Trials Network and included patients with advanced immunotherapy-naive SqNSCLC and a Zubrod score of 0 (asymptomatic) to 1 (symptomatic but completely ambulatory) with disease progression after standard platinum-based chemotherapy. Randomization was stratified by sex and number of prior therapies (1 vs 2 or more). Data were analyzed from May 3, 2018, to February 1, 2021.

**Interventions:**

Nivolumab, 3 mg/kg intravenously every 2 weeks, with or without ipilimumab, 1 mg/kg intravenously every 6 weeks, until disease progression or intolerable toxic effects.

**Main Outcomes and Measures:**

The primary end point was overall survival (OS). Secondary end points included investigator-assessed progression-free survival (IA-PFS) and response per Response Evaluation Criteria in Solid Tumors (RECIST) guidelines, version 1.1.

**Results:**

Of 275 enrolled patients, 252 (mean age, 67.5 years [range 41.8-90.3 years]; 169 men [67%]; 206 White patients [82%]) were deemed eligible (125 randomized to nivolumab/ipilimumab and 127 to nivolumab). The study was closed for futility at a planned interim analysis. Overall survival was not significantly different between the groups (hazard ratio [HR], 0.87; 95% CI, 0.66-1.16; *P* = .34). Median survival was 10 months (95% CI, 8.0-14.4 months) in the nivolumab/ipilimumab group and 11 months (95% CI, 8.6-13.7 months) in the nivolumab group. The IA-PFS HR was 0.80 (95% CI, 0.61-1.03; *P* = .09); median IA-PFS was 3.8 months (95% CI, 2.7-4.4 months) in the nivolumab/ipilimumab group and 2.9 months (95% CI, 1.8-4.0 months) in the nivolumab alone group. Response rates were 18% (95% CI, 12%-25%) with nivolumab/ipilimumab and 17% (95% CI, 10%-23%) with nivolumab. Median response duration was 28.4 months (95% CI, 4.9 months to not reached) with nivolumab/ipilimumab and 9.7 months with nivolumab (95% CI, 4.2-23.1 months). Grade 3 or higher treatment-related adverse events occurred in 49 of 124 patients (39.5%) who received nivolumab/ipilimumab and in 41 of 123 (33.3%) who received nivolumab alone. Toxic effects led to discontinuation in 31 of 124 patients (25%) on nivolumab/ipilimumab and in 19 of 123 (15%) on nivolumab.

**Conclusions and Relevance:**

In this phase 3 randomized clinical trial, ipilimumab added to nivolumab did not improve outcomes in patients with advanced, pretreated, immune checkpoint inhibitor–naive SqNSCLC.

**Trial Registration:**

ClinicalTrials.gov Identifier: NCT02785952

## Key Points

**Question:**

Does the addition of ipilimumab to nivolumab improve survival in patients with advanced chemotherapy-pretreated immunotherapy-naive squamous cell lung cancer?

**Findings:**

In this randomized clinical trial of 252 patients, the addition of ipilimumab to nivolumab did not lead to improved survival in patients with advanced chemotherapy-pretreated squamous cell carcinoma.

**Meaning:**

Combination therapy with nivolumab and ipilimumab is currently only indicated as first-line therapy in patients with advanced non–small cell lung cancer.

## Teaser

This phase 3 randomized clinical trial investigates if the addition of ipilimumab to nivolumab improves the survival outcome of patients with chemotherapy-pretreated immunotherapy-naive squamous cell lung cancer.

## Introduction

Programmed death 1 (PD-1) axis inhibitor monotherapies, including nivolumab, are currently standard salvage therapy for patients with immunotherapy-naive advanced non–small cell lung cancer (NSCLC) that has progressed on platinum-doublet chemotherapy.^1,2,3,4^ The PD-1 axis inhibitors pembrolizumab and atezolizumab have additionally been approved for use as first-line monotherapy in patients with advanced NSCLC expressing programmed death-ligand 1 (PD-L1) or in combination with chemotherapy regardless of tumor PD-L1 expression.^5,6,7,8,9,10,11^

Ipilimumab is an immune checkpoint inhibitor targeting cytotoxic T-lymphocyte–associated protein 4 (CTLA-4) that is approved for use as monotherapy in metastatic melanoma. The combination of ipilimumab with nivolumab has demonstrated superior efficacy compared with nivolumab alone in patients with advanced melanoma and is additionally approved in this setting.^12,13^ Phase 1 and 2 studies in patients with untreated advanced NSCLC showed promising early results with nivolumab plus ipilimumab, and 2 recent phase 3 trials in this population demonstrated superiority of the combination either alone or with chemotherapy compared with chemotherapy alone.^14,15,16,17^ Nivolumab plus ipilimumab is currently approved in the first-line setting for patients with metastatic PD-L1–expressing NSCLC and in combination with 2 cycles of platinum-doublet chemotherapy regardless of tumor PD-L1 expression.

The Lung Cancer Master Protocol (Lung-MAP) was designed as a biomarker-driven protocol to evaluate molecularly targeted therapies in biomarker-defined populations of patients with chemotherapy-pretreated advanced squamous (Sq) NSCLC. The initial iteration of the Lung-MAP protocol was designated S1400. ^18^ To enrich the probability of a patient participating, Lung-MAP includes both biomarker-driven studies and nonmatch studies for patients not eligible to participate in a biomarker-driven study. The second nonmatch trial within Lung-MAP (S1400), S1400I, evaluated the efficacy of nivolumab plus ipilimumab vs nivolumab alone in patients with immune checkpoint inhibitor–naive SqNSCLC. At the time this study was designed, nivolumab was approved as salvage therapy but not as initial treatment in patients with advanced NSCLC.

## Methods

### Study Design

This multicenter, open-label, phase 3 randomized clinical trial and substudy of Lung-MAP (S1400) was conducted from December 18, 2015, to April 23, 2018, through the National Clinical Trials Network and led by the SWOG Cancer Research Network. The trial protocol is available in Supplement 1, and the S1400 master protocol is available in Supplement 2. The trial compared nivolumab plus ipilimumab with nivolumab monotherapy in patients with chemotherapy-pretreated, immunotherapy-naive advanced SqNSCLC. To participate, each site required approval by the US National Cancer Institute central institutional review board or approval by their local institutional review board. Written informed consent was required from all patients before registration. This study followed the Consolidated Standards of Reporting Trials (CONSORT) reporting guideline.

### Eligibility

Patients must have been eligible for Lung-MAP (S1400) and not eligible for any of the actively accruing biomarker-driven substudies.^18,19^ Biomarker/drug pairs evaluated during this study have been described.^19,20,21,22,23,24^ Patients had histologically confirmed SqNSCLC with measurable disease by computed tomography or magnetic resonance imaging per Response Evaluation Criteria in Solid Tumors (RECIST) guidelines, version 1.1; were previously treated with platinum-doublet chemotherapy (within 1 year if given as part of curative-intent therapy without subsequent systemic therapy); and had a Zubrod performance status score of 0 (asymptomatic) to 1 (symptomatic but completely ambulatory). Patients must not have had prior treatment with an antibody or other drug specifically targeting T-cell costimulation or immune checkpoint pathways, including PD-1 and CTLA-4. Systemic treatment with either corticosteroids or other immunosuppressive medications within 14 days before substudy registration was not allowed. Patients could not have an active, known, or suspected autoimmune disease, HIV, hepatitis B, hepatitis C, or interstitial lung disease. Patients registered after September 1, 2016, who were able to complete a questionnaire in English participated in a patient-reported outcomes study.

### Biomarker Analysis

All patients in the Lung-MAP (S1400) study had biomarker screening by next-generation sequencing (Foundation Medicine) as previously described.^19^ Tumor mutational burden (TMB) was calculated as the number of somatic, coding, short variants, excluding known driver mutations, per megabase of the genome interrogated. Immunohistochemistry for PD-L1 staining and scoring was performed by the Clinical Laboratory Improvement Amendments–certified Biomarker Analysis Laboratory at the University of Colorado using the PD-L1 28-8 pharmDx kit (Dako) on the Dako Link 48 platform. The PD-L1 analysis was done after entry into the study and not used for stratification in randomization. For all stained specimens, 1 pathologist (H.Y.) scored all of the specimens, and a quality control pathologist scored 20% of the specimens. For discrepant results, a final score was determined by a consensus conference of the pathologists. Scoring was determined by the percentage of tumor cells with partial or complete cell membrane staining at any intensity.

### Randomization and Treatment

Patients were randomized in a 1:1 ratio to receive either nivolumab combined with ipilimumab or nivolumab alone. Randomization was stratified by sex (men vs women) and number of prior therapies (1 vs 2 or more) using a dynamic balancing algorithm.^25^

In both groups, nivolumab was administered intravenously at a dose of 3 mg/kg over 30 minutes on day 1 of 14-day cycles. For patients randomized to the nivolumab plus ipilimumab group, ipilimumab was administered intravenously at a dose of 1 mg/kg over 60 minutes on day 1 of every third cycle (ie, cycle 1, cycle 4, etc) starting 30 minutes after the end of nivolumab infusion. Disease assessments occurred every 6 weeks for the first year and every 3 months thereafter. Treatment was continued until disease progression or intolerable toxic effects. Postprogression therapy was allowed if the patient was experiencing clinical benefit in the opinion of the treating investigator.

### End Points

The primary end point for the trial was overall survival (OS), which was defined as the duration from randomization to death due to any cause. The OS for patients last known to be alive was censored at the date of last contact. Secondary end points included investigator-assessed progression-free survival (IA-PFS), response by RECIST 1.1, and toxic effects by the US National Cancer Institute’s new Patient-Reported Outcome Common Terminology Criteria for Adverse Events (PRO-CTCAE), version 4.03. The IA-PFS was defined as the duration from randomization to first occurrence of progression by RECIST 1.1, symptomatic deterioration, or death due to any cause. The IA-PFS for patients last known to be alive and free of progression or symptomatic deterioration was censored at the date of last disease assessment. Response was defined as the occurrence of a complete or partial response, confirmed or unconfirmed per RECIST 1.1 criteria. Response for patients not known to have a response was coded as nonresponse. Patient-reported symptoms were evaluated using the PRO-CTCAE items for diarrhea frequency, itching severity, fatigue severity, and interference with daily activities due to fatigue.^26^

### Statistical Analysis

The S1400I iteration of Lung-MAP was designed to have 90% power to rule out a hazard ratio (HR) equal to 1 if the true HR was 0.67 using a stratified log-rank test at the 1-sided 0.025 level. Assuming an accrual duration of 27 to 36 months, 9 months of follow-up, and a median OS of 9 months with nivolumab alone, 332 eligible patients were needed to achieve the 256 events required by design. Two interim analyses were planned at 50% and 75% of the expected deaths to evaluate early stopping for either efficacy or futility. Assessment of futility was based on testing the alternative hypothesis (HR = 0.67) using a modified stratified log-rank test for testing the non-null hypothesis at the 1-sided 0.0025 level. Assessment of early signs of efficacy was based on testing the null hypothesis using a stratified log-rank test at the 1-sided 0.0025 level.

Efficacy analyses (OS, IA-PFS, and response) were conducted on all randomized patients determined to be eligible (modified intention to treat). As is standard for National Clinical Trials Network trials, eligibility was confirmed after randomization. Safety analyses were conducted on all eligible patients who received at least 1 dose of study drug. Stratified log-rank tests using randomization stratification factors were used to analyze time-to-event data. Distribution of time-to-event outcomes was estimated using the Kaplan-Meier method, and the CI for medians was estimated using the Brookmeyer-Crowley method. Hazard ratios and associated 95% CIs were estimated with the use of a stratified Cox proportional hazards regression model. Descriptive statistics were used to summarize the characteristics of the patients. Analysis of the association between baseline factors, including TMB score and PD-L1 expression levels, with clinical outcomes was also done using a Cox proportional hazards regression model for OS and IA-PFS and logistic regression for response. The proportion of patients by arm experiencing a maximum postrandomization PRO-CTCAE score greater than 0 or greater than 3, both unadjusted and adjusted for the baseline PRO-CTCAE score, was compared using Fisher exact tests among patients with both baseline and follow-up scores.^27^

Prespecified biomarker analyses separately evaluated OS in the subset of patients with PD-L1 levels greater than or equal to 5% and for TMB levels greater than or equal to 10 mutations (mt)/Mb using a stratified log-rank test. Post hoc exploratory analysis of PD-L1 evaluated subgroup effects for 0%, 1% to 4%, 5% to 49%, 50% to 74%, and 75% and greater tumor PD-L1 expression to expand beyond the prespecified 5% cut point and for TMB levels by decile to evaluate whether there was evidence of a cut point for activity beyond the 10 mt/Mb cut point. Subsequent analyses evaluated subgroup effects of combined PD-L1 and TMB levels by using a Cox proportional hazard regression model including an interaction between continuous TMB score and treatment within the exploratory PD-L1 subgroups mentioned previously. If a signal of activity was observed (*P* < .10), evaluation of subgroup effects by TMB scores between the 20th and 70th percentiles would be pursued (to protect against small numbers, we did not use the full decile range) to evaluate whether the association with increasing TMB levels was increasing or whether there was a threshold above which activity was isolated. All statistical analyses were performed using SAS software, version 9.4 (SAS Institute Inc) and RStudio, version 1.4.1106 (R Core Team); data were analyzed from May 3, 2018, to February 1, 2021.

## Results

### Patient Characteristics and Treatment

**Figure 1.**
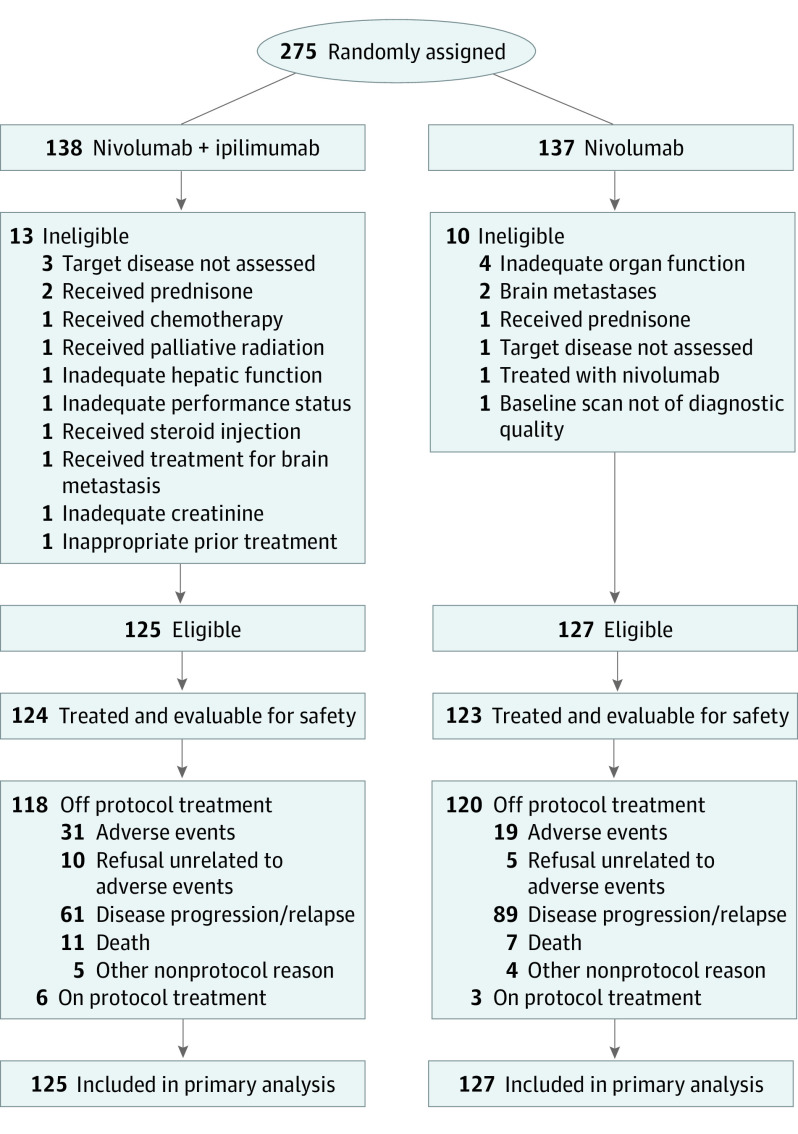
CONSORT Diagram

From December 18, 2015, to April 23, 2018, 275 patients were enrolled, of whom 252 patients were eligible for treatment (mean age, 67.5 years [range 41.8-90.3 years]; 169 men [67%]; 83 women [33%]; and 206 White patients [82%]). One hundred twenty-five patients were randomized to the nivolumab plus ipilimumab group and 127 to the nivolumab group. Details of patient enrollment, eligibility, and treatment are provided in Figure 1, and baseline characteristics are described in Table 1. The study was closed to accrual by the Data and Safety Monitoring Committee on April 23, 2018, after the first interim analysis demonstrated futility. The frequency of detection of individual alterations from the next-generation sequencing screening is detailed in eTable 1 in Supplement 3.

**Table 1.** Patient Characteristics

Variable	No. (%)		
	Nivolumab plus ipilimumab (n = 125)	Nivolumab (n = 127)	Total (N = 252)
Age, median (range), y	67.5 (41.8-83.4)	68.1 (48.6-90.3)	67.5 (41.8-90.3)
Men	83 (66)	86 (68)	169 (67)
Women	42 (34)	41 (32)	83 (33)
Race/ethnicity			
White	102 (82)	104 (82)	206 (82)
Black	17 (14)	16 (13)	33 (13)
Asian	0	4 (3)	4 (2)
Pacific Islander	0	1 (1)	1 (0.4)
Native American	1 (1)	2 (2)	3 (1)
Multiracial	1 (1)	0	1 (0.4)
Unknown	4 (3)	0	4 (2)
Hispanic	2 (2)	1 (1)	3 (1)
Unknown	3 (2)	0	3 (1)
No. of prior systemic therapies for stage IV or recurrent disease			
0^a^	35 (28)	35 (28)	70 (28)
1	73 (58)	77 (61)	150 (60)
2	6 (5)	7 (6)	13 (5)
≥3	3 (2)	1 (1)	4 (1)
Unknown	8 (6)	7 (6)	15 (6)
Zubrod performance score			
0	36 (29)	35 (28)	71 (28)
1	89 (71)	92 (72)	181 (72)
Weight loss in past 6 mo			
<5%	86 (69)	93 (73)	179 (71)
5% to <10%	25 (20)	18 (14)	43 (17)
10% to <20%	13 (10)	13 (10)	26 (10)
≥20%	1 (1)	3 (2)	4 (2)
Smoking status			
Current	48 (38)	54 (43)	102 (40)
Former	75 (60)	72 (57)	147 (58)
Never	2 (2)	1 (1)	3 (1)
Brain metastases at baseline	8 (6)	12 (9)	20 (8)
Liver metastases at baseline	23 (18)	30 (24)	53 (21)
Prior radiation therapy			
Localized radiation	36 (29)	49 (39)	85 (34)
Radiation with curative intent	41 (33)	45 (35)	86 (34)

^a^ Includes patients who received systemic therapy for stage I to III non–small cell lung cancer within 1 y of S1400I treatment.

A total of 124 patients in the nivolumab plus ipilimumab group and 123 patients in the nivolumab group received at least 1 dose of protocol treatment, with a median of 7 doses in both groups (ranges, 1-84 doses for nivolumab plus ipilimumab and 1-83 doses for nivolumab alone). Trial therapy was continued after progression in 38 of 116 patients (33%) in the nivolumab group and 37 of 104 patients (36%) in the nivolumab plus ipilimumab group. As of December 19, 2019, 9 patients were still receiving trial therapy (6 in the nivolumab plus ipilimumab group and 3 in the nivolumab group). A total of 50 patients (20%) discontinued due to toxic effects (31 of 124 [25%] in the nivolumab plus ipilimumab group and 19 of 123 [15%] in the nivolumab group). Median time to discontinuation of therapy due to toxic effects was 5.6 months (95% CI, 3.3-10.4 months) with nivolumab plus ipilimumab and 4.0 months (95% CI, 2.0-6.4 months) with nivolumab alone.

### Efficacy

**Figure 2.**
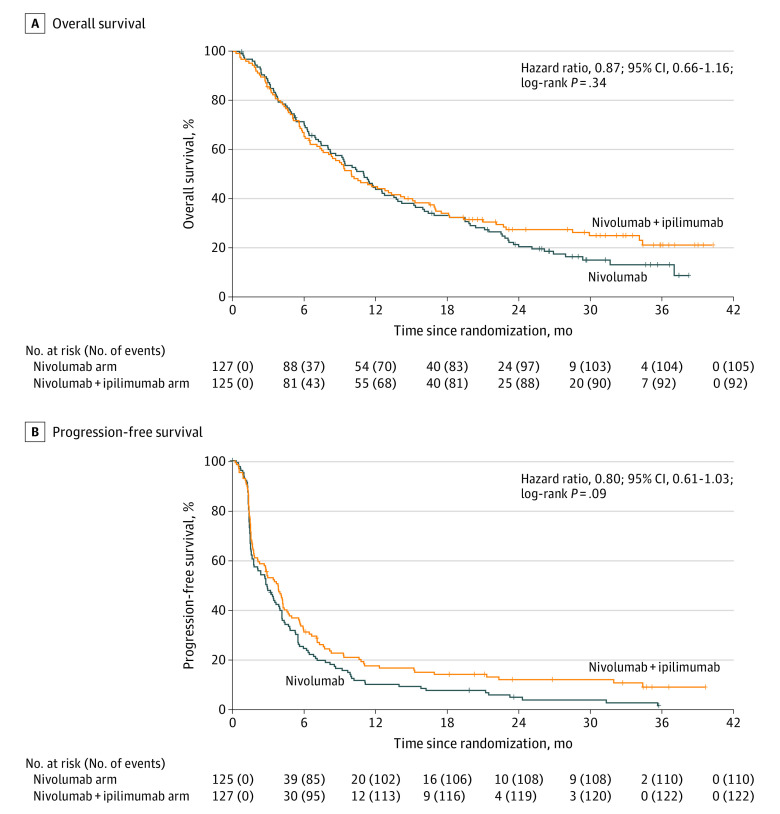
Kaplan-Meier Curves for Overall Survival and Progression-Free Survival A, Overall survival for nivolumab vs nivolumab plus ipilimumab. B, Progression-free survival for nivolumab vs nivolumab plus ipilimumab. Tick marks represent censored observations.

As of December 19, 2019, 197 deaths had been reported (92 [47%] in the nivolumab plus ipilimumab group and 105 [53%] in the nivolumab group). The median follow-up in surviving patients was 29.5 months (95% CI, 26.0-32.8 months). Overall survival was not significantly different between the groups (HR, 0.87; 95% CI, 0.66-1.16; *P* = .34) (Figure 2A). Median survival was 10 months (95% CI, 8.0-14.4 months) in the nivolumab plus ipilimumab group and 11 months (95% CI, 8.6-13.7 months) in the nivolumab group; 1- and 2-year OS rates were 45% (95% CI, 37%-55%) and 28% (95% CI, 21%-37%), respectively, in the nivolumab plus ipilimumab group and 44% (95% CI, 36%-54%) and 22% (95% CI, 15%-30%), respectively, in the nivolumab group.

Progression-free survival was not significantly different between the groups (HR, 0.80; 95% CI, 0.61-1.03; *P* = .09). Median IA-PFS was 3.8 months (95% CI, 2.7-4.4 months) in the nivolumab plus ipilimumab arm and 2.9 months (95% CI, 1.8-4.0 months) in the nivolumab group (Figure 2B); 1- and 2-year PFS rates were 17% (95% CI, 12%-25%) and 12% (95% CI, 7%-19%), respectively, in the combination arm and 10% (95% CI, 6%-17%) and 5% (95% CI, 2%-10%), respectively, in the monotherapy arm.

The objective response rate was 18% (95% CI, 12%-25%) with nivolumab plus ipilimumab and 17% (95% CI, 10%-23%) with nivolumab alone. There were 22 confirmed responses, including 2 complete responses, 20 partial responses, and 1 unconfirmed partial response, in the combination group and 15 confirmed responses, including 1 complete response, 14 partial responses, and 6 unconfirmed partial responses, in the nivolumab alone group. Median duration of response was 28.4 months (95% CI, 4.9 months to not reached) with nivolumab plus ipilimumab and 9.7 months (95% CI, 4.2-23.1 months) with nivolumab alone (eFigure 1 in Supplement 3). Removing the 6 unconfirmed responses on nivolumab, the median duration of response was 13.2 months (95% CI, 9.7-20.2 months).

### Tumor PD-L1 Expression and TMB

Tumor PD-L1 was known for 161 patients (64%), and TMB was known for 231 patients (92%); both were known for 149 patients (59%) (eTable 2 in Supplement 3). Baseline characteristics of patients with known tumor PD-L1 and TMB were similar to the total population (eTable 3 in Supplement 3). Median TMB was 10.9 mt/Mb (range, 2.2-67.1 mt/Mb), and 128 of 231 patients (55%) had a TMB greater than or equal to 10 mt/Mb. A total of 63 of 161 patients (39%) had PD-L1 less than 1%, 70 (44%) had at least 5% expression, and 38 (24%) had at least 50% PD-L1 expression.

**Figure 3.**
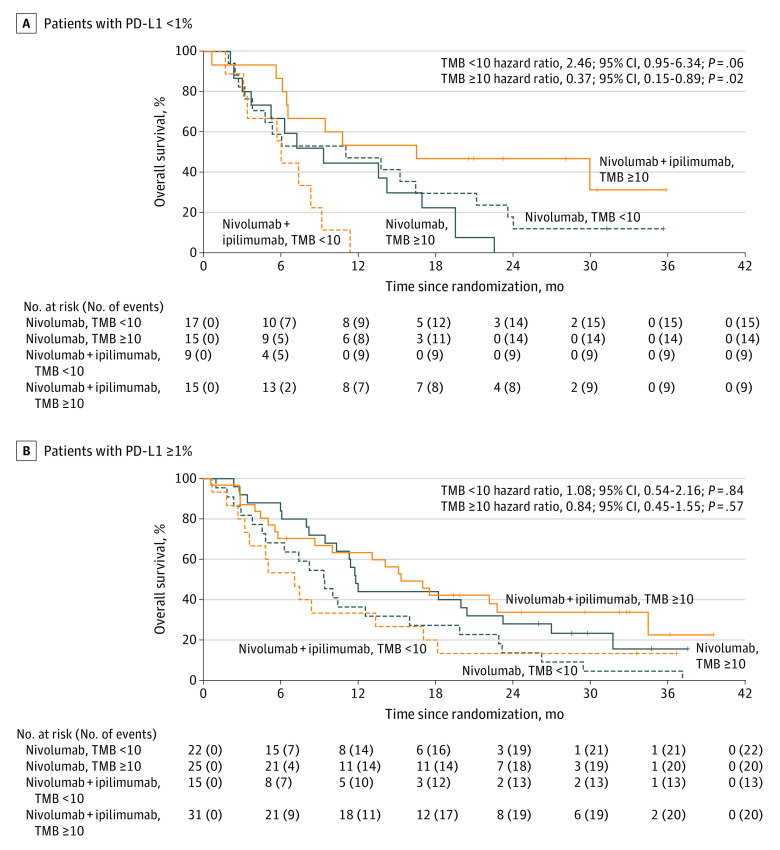
Overall Survival by Tumor Mutational Burden (TMB) and Programmed Death-Ligand 1 (PD-L1) Subsets Survival A, Nivolumab vs nivolumab plus ipilimumab by TMB in patients with PD-L1 < 1%. B, Nivolumab vs nivolumab plus ipilimumab by TMB in patients with PD-L1 ≥ 1%. Tick marks represent censored observations.

The TMB levels and PD-L1 score were not correlated (correlation coefficient = 0.09; *P* = .29) (eFigure 2 in Supplement 3). Treatment was not associated with OS in either of the prespecified thresholds (TMB≥10 mt/Mb and PD-L1 expression ≥5%) or for the exploratory thresholds for PD-L1 expression or TMB (*P* > .05 for all). Higher TMB levels, evaluated as a continuous measure, were associated with improved survival in the subset of patients with PD-L1 expression less than 1% (56 patients and 47 events; *P* = .06) (eTable 4 in Supplement 3), and the benefit was likely best isolated to the subset with TMB greater than or equal to 10 mt/Mb (eFigure 3 in Supplement 3). Subsequent analysis showed improved survival with nivolumab plus ipilimumab over nivolumab alone in patients with TMB greater than or equal to 10 mt/Mb and PD-L1 expression less than 1% (n = 15 for both arms; HR, 0.37; 95% CI, 0.15-0.89; *P* = .02) but survival detriment in patients with TMB less than 10 mt/Mb and PD-L1 expression less than 1% (n = 17 for nivolumab alone and n = 9 for nivolumab plus ipilimumab; HR, 2.46; 95% CI, 0.95-6.34; *P* = .06) (Figure 3). Of note, the exploratory nature of these combined analyses and the small number of patients in each subset limit conclusions. Tumor response in the 2 subsets is provided in eTable 5 in Supplement 3.

### Safety

Grade 3 or higher treatment-related adverse events (AEs) occurred in 49 of 124 patients (39.5%) receiving nivolumab plus ipilimumab and 41 of 123 patients (33.3%) receiving nivolumab alone (Table 2). The most common grade 3 and higher treatment-related events in patients receiving nivolumab plus ipilimumab or nivolumab alone included fatigue (11 of 124 [8.9%] and 7 of 123 [5.7%], respectively) and pneumonitis (9 of 124 [7.3%] and 6 of 123 [4.9%], respectively). Treatment-related deaths occurred in 3 of 124 patients (2.4%) who received nivolumab plus ipilimumab (dyspnea, respiratory failure, or death not otherwise specified) and in 1 of 123 patients (0.8%) who received nivolumab alone (pneumonitis). Immune-related AEs were reported in 82 of 124 patients (66%) on nivolumab plus ipilimumab and 73 of 123 patients (59%) on nivolumab alone (eFigure 4 in Supplement 3). The most common immune-related events of all grades for patients receiving nivolumab plus ipilimumab were rash (30 [24%]), dyspnea (23 [19%]), and diarrhea and hypothyroidism (22 [18%] for each) and for patients receiving nivolumab were diarrhea (26 [21%]), dyspnea (24 [20%]), and hypothyroidism (14 [11%]) (eFigure 4 in Supplement 3). Using PRO-CTCAE, there were no differences in AEs appreciated between groups (eTable 6 in Supplement 3).

**Table 2.** Grade 3 Events With at Least 5% Prevalence and All Grade 4 or 5 Adverse Events Attributable to Treatment

Variable	No. (%)							
	Nivolumab plus ipilimumab (n = 124)				Nivolumab (n = 123)			
	Grade 3	Grade 4	Grade 5	Any grade 3-5	Grade 3	Grade 4	Grade 5	Any grade 3-5
Acute kidney injury	0	1 (1)	0	1 (1)	1 (1)	0	0	1 (1)
ALT increased	4 (3)	1 (1)	0	5 (4)	2 (2)	0	0	2 (2)
AST increased	5 (4)	1 (1)	0	6 (5)	2 (2)	0	0	2 (2)
AV block complete	0	1 (1)	0	1 (1)	0	0	0	0
Cardiac arrest	0	1 (1)	0	1 (1)	0	0	0	0
Death NOS	0	0	1 (1)	1 (1)	0	0	0	0
Dyspnea	4 (3)	0	1 (1)	5 (4)	5 (4)	0	0	5 (4)
Fatigue	11 (9)	0	0	11 (9)	7 (6)	0	0	7 (6)
Heart failure	0	1 (1)	0	1 (1)	0	0	0	0
Hyperglycemia	0	1 (1)	0	1 (1)	1 (1)	0	0	1 (1)
Hyperkalemia	0	1 (1)	0	1 (1)	0	0	0	0
Hypertension	3 (2)	1 (1)	0	4 (3)	1 (1)	0	0	1 (1)
Hyponatremia	5 (4)	2 (2)	0	7 (6)	3 (2)	0	0	3 (2)
Hypoxia	3 (2)	1 (1)	0	4 (3)	0	1 (1)	0	1 (1)
Lipase increased	4 (3)	2 (2)	0	6 (5)	1 (1)	0	0	1 (1)
Lung infection	2 (2)	0	0	2 (2)	6 (5)	0	0	6 (5)
Myocarditis	0	1 (1)	0	1 (1)	0	0	0	0
Pneumonitis	8 (7)	1 (1)	0	9 (7)	5 (4)	0	1 (1)	6 (5)
Respiratory failure	0	1 (1)	1 (1)	2 (2)	0	0	0	0
Sepsis	0	1 (1)	0	1 (1)	0	1 (1)	0	1 (1)
Stroke	0	1 (1)	0	1 (1)	0	0	0	0
Thromboembolic event	0	1 (1)	0	1 (1)	1 (1)	0	0	1 (1)
Max grade, any adverse event^a^	39 (32)	7 (6)	3 (2)	49 (40)	39 (32)	1 (1)	1 (1)	41 (33)

Abbreviations: ALT, alanine aminotransferase; AST, aspartate aminotransferase; AV, atrioventricular; NOS, not otherwise specified.

^a^ Includes all grade 3 events regardless of prevalence (including grade 3 events with <5% prevalence).

## Discussion

Although nivolumab combined with ipilimumab has demonstrated superior efficacy to nivolumab alone in advanced melanoma and to first-line platinum-based chemotherapy in advanced NSCLC, the combination did not improve OS over nivolumab alone in patients with immunotherapy-naïve, pretreated advanced SqNSCLC. Prior trials have suggested that high TMB may predict greater benefit with nivolumab plus ipilimumab in advanced NSCLC, although optimal cutoff for TMB differs across studies. In our study, high TMB defined as a cutoff of 10 mt/Mb did not lead to a better outcome with combination therapy. Additional biomarker analyses from this trial, including tumor PD-L1 expression and tumor sequencing, did not identify individual tumor characteristics predictive of benefit from either treatment over the other. However, in patients with no tumor PD-L1 expression, high TMB predicted a benefit with nivolumab plus ipilimumab, whereas low TMB predicted inferior survival compared with nivolumab alone. Consistent with previous studies, TMB did not correlate with tumor PD-L1 status.

Subsequent to this trial, treatment paradigms for advanced NSCLC changed such that most patients will receive immunotherapy as front-line therapy. However, although the question posed by this trial is no longer as relevant, there is still a need to understand potential differences among various PD-1 axis inhibitors and whether first-line combination therapy with an anti–CTLA-4 agent will improve outcomes over PD-1 axis inhibitor therapy, either alone or with concurrent chemotherapy. In CheckMate 227,^16^ first-line therapy with nivolumab plus ipilimumab in patients with PD-L1–expressing NSCLC resulted in a numerically higher response rate and median duration of response (36.4% and 23.2 months, respectively) than with nivolumab monotherapy (27.5% and 15.5 months, respectively). Although not a primary end point of that trial, risk of progression was decreased with nivolumab plus ipilimumab compared with nivolumab alone (HR, 0.83; 95% CI, 0.71-0.97). Difference in OS did not reach statistical significance (HR, 0.90; 95% CI, 0.76-1.07). Among patients with tumor PD-L1 expression less than 1%, nivolumab plus ipilimumab resulted in longer OS and higher 2-year PFS rates (20% and 6%, respectively) than did chemotherapy, highlighting the need for other biomarkers in this population. Interestingly, exploratory analysis in the subgroup with PD-L1 less than 1% found that the PFS benefit with nivolumab plus ipilimumab was limited to patients with high TMB.

### Limitations

As discussed, the main limitation of our study is that the control group of nivolumab is no longer a commonly used salvage therapy for patients with advanced lung cancer; most patients will receive PD-1 axis inhibitor therapy in the first-line setting. Another limitation is that adequate tissue for determining tumor PD-L1 status was not required. Indeed, only 64% of patients had successful tumor PD-L1 analysis; among these patients, there did not appear to be major differences in PD-L1 expression between arms. Reduced numbers of patients with available results from both tumor PD-L1 and mutational burden assessment do limit conclusions from the combined subset analyses that evaluated the outcome presented above.

## Conclusions

In this multicenter, open-label, phase 3 randomized clinical trial and substudy of Lung-MAP (S1400), ipilimumab added to nivolumab did not improve outcomes in patients with advanced, pretreated, immune checkpoint inhibitor–naive SqNSCLC. At present, there is no immunotherapy option for patients who experience disease progression on PD-1 axis inhibitor therapy. At least 2 ongoing trials are evaluating nivolumab plus ipilimumab as salvage therapy in patients with progressive advanced NSCLC after PD-1 axis inhibitor therapy.^28,29^ These studies enroll both patients with acquired and primary resistance to PD-1 axis inhibitor therapy. Until these studies and others are completed, there will continue to be no role for combination therapy with nivolumab and ipilimumab in patients with pretreated advanced NSCLC.

## Notes

Supplement 1.
Trial Protocol

Supplement 2.
S1400 Master Protocol

Supplement 3. **eTable 1.** Gene Alterations Detected on FoundationOne Next-Generation Sequencing Screening **eTable 2.** Tumor Mutational Burden and PD-L1 Expression **eTable 3.** Patient Characteristics, Total Population and Population With PD-L1 and TMB Results **eTable 4.** Cox Model Results Evaluating the Interaction Between TMB as a Continuous Variable and Treatment Arm for the Association With Overall Survival Within PD-L1 Subsets **eTable 5.** Responses Reported by Treatment Arm and TMB in the Subset of Patients With PD-L1 Expression <1% **eTable 6.** Patient-Reported-Outcome Common Terminology Criteria for Adverse Events (PRO-CTCAE) by Arm (N, %) **eFigure 1.** Duration of Response by Treatment Arm **eFigure 2.** Distribution and Comparison of TMB Levels and PDL1 Expression Levels **eFigure 3.** Overall Survival Across Levels of TMB in Subgroup With Tumor PD-L1 < 1% **eFigure 4.** Immune-Related Adverse Events

Supplement 4.
Data Sharing Statement

## References

[1] Borghaei H, Paz-Ares L, Horn L, . Nivolumab versus docetaxel in advanced nonsquamous non-small-cell lung cancer. N Engl J Med. 2015;373(17):1627-1639. doi:10.1056/NEJMoa1507643 26412456 PMC5705936

[2] Brahmer J, Reckamp KL, Baas P, . Nivolumab versus docetaxel in advanced squamous-cell non-small-cell lung cancer. N Engl J Med. 2015;373(2):123-135. doi:10.1056/NEJMoa1504627 26028407 PMC4681400

[3] Herbst RS, Baas P, Kim DW, . Pembrolizumab versus docetaxel for previously treated, PD-L1-positive, advanced non-small-cell lung cancer (KEYNOTE-010): a randomised controlled trial. Lancet. 2016;387(10027):1540-1550. doi:10.1016/S0140-6736(15)01281-7 26712084

[4] Rittmeyer A, Barlesi F, Waterkamp D, ; OAK Study Group. Atezolizumab versus docetaxel in patients with previously treated non-small-cell lung cancer (OAK): a phase 3, open-label, multicentre randomised controlled trial. Lancet. 2017;389(10066):255-265. doi:10.1016/S0140-6736(16)32517-X 27979383 PMC6886121

[5] Reck M, Rodríguez-Abreu D, Robinson AG, ; KEYNOTE-024 Investigators. Pembrolizumab versus chemotherapy for PD-L1-positive non-small-cell lung cancer. N Engl J Med. 2016;375(19):1823-1833. doi:10.1056/NEJMoa1606774 27718847

[6] Mok TSK, Wu YL, Kudaba I, ; KEYNOTE-042 Investigators. Pembrolizumab versus chemotherapy for previously untreated, PD-L1-expressing, locally advanced or metastatic non-small-cell lung cancer (KEYNOTE-042): a randomised, open-label, controlled, phase 3 trial. Lancet. 2019;393(10183):1819-1830. doi:10.1016/S0140-6736(18)32409-7 30955977

[7] Gandhi L, Rodríguez-Abreu D, Gadgeel S, ; KEYNOTE-189 Investigators. Pembrolizumab plus chemotherapy in metastatic non-small-cell lung cancer. N Engl J Med. 2018;378(22):2078-2092. doi:10.1056/NEJMoa1801005 29658856

[8] Paz-Ares L, Luft A, Vicente D, ; KEYNOTE-407 Investigators. Pembrolizumab plus chemotherapy for squamous non-small-cell lung cancer. N Engl J Med. 2018;379(21):2040-2051. doi:10.1056/NEJMoa1810865 30280635

[9] Spigel DR, De Marinis F, Giaccone G, . IMpower110: interim overall survival (OS) analysis of a phase III study of atezolizumab (atezo) vs platinum-based chemotherapy (chemo) as first-line (1L) treatment (tx) in PD-L1–selected NSCLC. *Ann Oncol**.* 2019;30(suppl 5):V915. doi:10.1093/annonc/mdz293

[10] Socinski MA, Jotte RM, Cappuzzo F, ; IMpower150 Study Group. Atezolizumab for first-line treatment of metastatic nonsquamous NSCLC. N Engl J Med. 2018;378(24):2288-2301. doi:10.1056/NEJMoa1716948 29863955

[11] West H, McCleod M, Hussein M, . Atezolizumab in combination with carboplatin plus nab-paclitaxel chemotherapy compared with chemotherapy alone as first-line treatment for metastatic non-squamous non-small-cell lung cancer (IMpower130): a multicentre, randomised, open-label, phase 3 trial. Lancet Oncol. 2019;20(7):924-937. doi:10.1016/S1470-2045(19)30167-6 31122901

[12] Larkin J, Chiarion-Sileni V, Gonzalez R, . Combined nivolumab and ipilimumab or monotherapy in untreated melanoma. N Engl J Med. 2015;373(1):23-34. doi:10.1056/NEJMoa1504030 26027431 PMC5698905

[13] Wolchok JD, Chiarion-Sileni V, Gonzalez R, . Overall survival with combined nivolumab and ipilimumab in advanced melanoma. N Engl J Med. 2017;377(14):1345-1356. doi:10.1056/NEJMoa1709684 28889792 PMC5706778

[14] Hellmann MD, Rizvi NA, Goldman JW, . Nivolumab plus ipilimumab as first-line treatment for advanced non-small-cell lung cancer (CheckMate 012): results of an open-label, phase 1, multicohort study. Lancet Oncol. 2017;18(1):31-41. doi:10.1016/S1470-2045(16)30624-6 27932067 PMC5476941

[15] Ready N, Hellmann MD, Awad MM, . First-line nivolumab plus ipilimumab in advanced non-small-cell lung cancer (CheckMate 568): outcomes by programmed death ligand 1 and tumor mutational burden as biomarkers. J Clin Oncol. 2019;37(12):992-1000. doi:10.1200/JCO.18.01042 30785829 PMC6494267

[16] Hellmann MD, Paz-Ares L, Bernabe Caro R, . Nivolumab plus ipilimumab in advanced non-small-cell lung cancer. N Engl J Med. 2019;381(21):2020-2031. doi:10.1056/NEJMoa1910231 31562796

[17] Paz-Ares L, Ciuleanu TE, Cobo M, et al. First-line nivolumab plus ipilimumab combined with two cycles of chemotherapy in patients with non-small-cell lung cancer (CheckMate 9LA): an international, randomised, open-label, phase 3 trial. *Lancet Oncol.* 2021;22(2):198-211. doi:10.1016/S1470-2045(20)30641-0 33476593

[18] Herbst RS, Gandara DR, Hirsch FR, . Lung Master Protocol (Lung-MAP)—a biomarker-driven protocol for accelerating development of therapies for squamous cell lung cancer: SWOG S1400. Clin Cancer Res. 2015;21(7):1514-1524. doi:10.1158/1078-0432.CCR-13-3473 25680375 PMC4654466

[19] Redman MW, Papadimitrakopoulou VA, Minichiello K, . Biomarker-driven therapies for previously treated squamous non-small-cell lung cancer (Lung-MAP SWOG S1400): a biomarker-driven master protocol. Lancet Oncol. 2020;21(12):1589-1601. doi:10.1016/S1470-2045(20)30475-7 33125909 PMC8109255

[20] Langer CJ, Redman MW, Wade JL III, . SWOG S1400B (NCT02785913), a phase II study of GDC-0032 (taselisib) for previously treated PI3K-positive patients with stage IV squamous cell lung cancer (Lung-MAP substudy). J Thorac Oncol. 2019;14(10):1839-1846. doi:10.1016/j.jtho.2019.05.029 31158500 PMC7017958

[21] Edelman MJ, Redman MW, Albain KS, . SWOG S1400C (NCT02154490)—a phase II study of palbociclib for previously treated cell cycle gene alteration-positive patients with stage IV squamous cell lung cancer (Lung-MAP Substudy). J Thorac Oncol. 2019;14(10):1853-1859. doi:10.1016/j.jtho.2019.06.027 31302234 PMC6764876

[22] Aggarwal C, Redman MW, Lara PN Jr, . SWOG S1400D (NCT02965378), a phase II study of the fibroblast growth factor receptor inhibitor AZD4547 in previously treated patients with fibroblast growth factor pathway-activated stage IV squamous cell lung cancer (Lung-MAP substudy). J Thorac Oncol. 2019;14(10):1847-1852. doi:10.1016/j.jtho.2019.05.041 31195180 PMC6901020

[23] Owonikoko TK, Redman MW, Byers LA, . A phase 2 study of talazoparib in patients with homologous recombination repair-deficient squamous cell lung cancer: Lung-MAP Substudy S1400G. *Clin Lung Cancer.* 2021;22(3):187-194.E1. doi:10.1016/j.cllc.2021.01.001 33583720 PMC8637652

[24] Waqar SN, Redman MW, Arnold SM, . A phase II study of telisotuzumab vedotin in patients with c-MET-positive stage IV or recurrent squamous cell lung cancer (LUNG-MAP sub-study S1400K, NCT03574753). *Clin Lung Cancer.* 2021;22(3):170-177. doi:10.1016/j.cllc.2020.09.013 33221175 PMC8044254

[25] Pocock SJ, Simon R. Sequential treatment assignment with balancing for prognostic factors in the controlled clinical trial. Biometrics. 1975;31(1):103-115. doi:10.2307/2529712 1100130

[26] Basch E, Reeve BB, Mitchell SA, . Development of the National Cancer Institute’s patient-reported outcomes version of the Common Terminology Criteria for Adverse Events (PRO-CTCAE). J Natl Cancer Inst. 2014;106(9):dju244. doi:10.1093/jnci/dju244 25265940 PMC4200059

[27] Basch E, Rogak LJ, Dueck AC. Methods for implementing and reporting Patient-Reported Outcome (PRO) measures of symptomatic adverse events in cancer clinical trials. Clin Ther. 2016;38(4):821-830. doi:10.1016/j.clinthera.2016.03.011 27045992 PMC4851916

[28] An investigational immune-therapy study to test combination treatments in patients with advanced non-small cell lung cancer (FRACTION-Lung). ClinicalTrials.gov identifier: NCT02750514. Updated March 22, 2021. Accessed April 5, 2021. https://clinicaltrials.gov/ct2/show/NCT02750514

[29] Ipilimumab and nivolumab in patients with anti-PD-1-axis therapy-resistant advanced non-small cell lung cancer. ClinicalTrials.gov identifier:NCT03262779. Updated December 22, 2020. Accessed April 5, 2021. https://clinicaltrials.gov/ct2/show/NCT03262779

