# Supplementary material for: Nivolumab Plus Ipilimumab vs Nivolumab for Previously Treated Patients With Stage IV Squamous Cell Lung Cancer: The Lung-MAP S1400I Phase 3 Randomized Clinical Trial
Source: JAMA Oncol. 2021 Jul 15;7(9):1368–77. doi: 10.1001/jamaoncol.2021.2209 (PMC8283667; doi:10.1001/jamaoncol.2021.2209)
Supplement: Supplement 1. — Trial Protocol [file jamaoncol-e212209-s001.pdf]

S1400I: (Non-Match sub-study): Nivolumab plus Ipilimumab versus Nivolumab

A BIOMARKER-DRIVEN MASTER PROTOCOL FOR PREVIOUSLY TREATED SQUAMOUS CELL LUNG CANCER

A PHASE III RANDOMIZED STUDY OF NIVOLUMAB PLUS IPILIMUMAB VERSUS NIVOLUMAB FOR PREVIOUSLY TREATED PATIENTS WITH STAGE IV SQUAMOUS CELL LUNG CANCER AND NO MATCHING BIOMARKER (LUNG-MAP SUB-STUDY)

Bristol Myers Squibb Protocol #: CA209-489

NCT #02785952

This is a potential FDA registration study. There will be additional centralized and on-site monitoring conducted in addition to routine audits. Sites must also maintain a study specific Trial Master File for this study (https://swog.org/Visitors/QA/Index.asp).

CLOSED EFFECTIVE 04/23/2018

SUB-STUDY CHAIRS:

Scott N. Gettinger, M.D.
(Medical Oncology)
NCTN Group: SWOG
Yale Cancer Center
MYSM School of Medicine
333 Cedar Street
New Haven, CT 06520
Phone: 203/785-7564
FAX: 203/785-3788
E-mail: scott.gettinger@yale.edu

Lyudmila A. Bazhenova, M.D.
(Medical Oncology)
NCTN Group: Alliance
UC San Diego Moores Cancer Center
3855 Health Sciences Dr.
La Jolla, CA 92093
Phone: 858/822-6189
FAX: 858/822-6190
E-mail: lbazhenova@ucsd.edu

BIOSTATISTICIANS:

Mary Redman, Ph.D.
Katherine Griffin, M.S.
Jieling Miao, M.S.
James Moon, M.S.
Joseph Unger, Ph.D. (Patient Reported Outcomes)
SWOG Statistics and Data Management Center
1100 Fairview Ave N, M3-C102
P.O. Box 19024
Seattle, WA 98109-1024
Phone: 206/667-4623
FAX: 206/667-4408
E-mail: mredman@fredhutch.org
E-mail: kgriffin@fredhutch.org
E-mail: jmiao@fredhutch.org
E-mail: jmoon@fredhutch.org
E-mail: junger@fredhutch.org

STUDY AGENTS:

Available from Pharmaceutical Collaborator:
Nivolumab (NSC 748726) (SWOG IND-119672)
Ipilimumab (NSC 732442) (SWOG IND-119672)

NURSE PRO/QOL STUDY COORDINATOR

Susan S. Tavernier, PhD, APRN-CNS, AOCN
Assistant Professor
Accelerated Nursing Program Coordinator
Idaho State University School of Nursing
Phone: 208/373-1783
Email: tavesusa@isu.edu

TABLE OF CONTENTS

TITLE 1
TABLE OF CONTENTS 2
CANCER TRIALS SUPPORT UNIT (CTSU) ADDRESS AND CONTACT INFORMATION 4
SCHEMA 5
1.0 OBJECTIVES 6
1.1 Primary Objective 6
1.2 Secondary Objective(s) 6
1.3 Translational Medicine Objectives 6
2.0 BACKGROUND 7
3.0 DRUG INFORMATION 10
3.1 Nivolumab (BMS-936558, MDX1106, Opdivo®) (NSC # 748726) (IND-119672) 10
3.2 Ipilimumab (BMS-734016, MDX-010, YERVOY®) (NSC 732442) (IND-119672) 18
4.0 STAGING CRITERIA 26
5.0 ELIGIBILITY CRITERIA 26
5.1 Sub-Study Specific Disease Related Criteria 27
5.2 Sub-Study Specific Clinical/Laboratory Criteria 27
5.3 Common Eligibility Criteria for all Sub-Studies 28
6.0 STRATIFICATION FACTORS 30
6.1 Patients will be randomized between nivolumab with or without ipilimumab using block randomization 30
6.2 Randomization will be stratified by: 30
7.0 TREATMENT PLAN 31
7.1 Pre-Medication and Supportive Care 31
7.2 Treatment – Non-Match **S1400I** 31
7.3 Criteria for Removal from Protocol Treatment 32
7.4 Discontinuation of Treatment 32
7.5 Follow-Up Period 32
8.0 TOXICITIES TO BE MONITORED AND DOSE MODIFICATIONS 33
8.1 NCI Common Terminology Criteria for Adverse Events 33
8.2 General Considerations 33
8.3 Dose Modifications – Nivolumab and Ipilimumab, Arm 1 33
8.4 Dose Modifications – Nivolumab, Arm 2 35
8.5 Treatment Discontinuation Criteria Nivolumab and the Combination (Nivolumab with Ipilimumab) 37
8.6 Treatment Discontinuation Criteria Ipilimumab 38
8.7 Treatment of Nivolumab or Ipilimumab Infusion Reactions 39
8.8 Dose Modification and Management for Cardiomyopathy Myocarditis 41
8.9 Management Algorithms for Immuno-Oncology Agents 41
8.10 Dose Modification Contacts 48
8.11 Adverse Event Reporting 48
9.0 STUDY CALENDAR 49
10.0 CRITERIA FOR EVALUATION AND ENDPOINT ANALYSIS 52
10.1 Measurability of Lesions 52
10.2 Objective Status at Each Disease Evaluation 54
10.3 Best Response 55
10.4 Performance Status 56
10.5 Time to Death 56
10.6 Investigator-Assessed Progression-Free Survival (IA-PFS) 57
10.7 Progression-Free Survival by Central Review 57
10.8 Duration of Response (DoR) 57
11.0 STATISTICAL CONSIDERATIONS 57
11.1 Primary Objective 57
11.2 Sample Size with Power Justification 57
11.3 Analysis Plan 58
11.4 Data and Safety Monitoring Committee 59
11.5 Translational Medicine 59

CLOSED EFFECTIVE 04/23/2018

SWOG Authorized Document

12.0 DISCIPLINE REVIEW 59
13.0 REGISTRATION GUIDELINES 59
13.1 Registration Timing 59
14.0 DATA SUBMISSION SCHEDULE 60
14.1 Data Submission Requirements 60
14.2 Master Forms 60
14.3 Data Submission Procedures 60
14.4 Data Submission Overview and Timepoints 61
15.0 SPECIAL INSTRUCTIONS 63
15.1 SWOG Specimen Tracking System (STS) 63
15.2 Correlative Studies and Banking (Optional for Patients) 63
15.3 PD-L1 IHC Testing 64
15.4 Radiology Review (Required) 64
15.5 **S1400I** PRO Questionnaire Administration Instructions 65
16.0 ETHICAL AND REGULATORY CONSIDERATIONS 66
16.1 ADVERSE EVENT REPORTING REQUIREMENTS 66
17.0 BIBLIOGRAPHY 70
18.0 APPENDIX 71
18.1 Translational Medicine - PD-L1 IHC Testing 72
18.2 Translational Medicine – S1400I Patient Reported Outcomes (PRO) 76

CLOSED EFFECTIVE 04/23/2018

SWOG Authorized Document

CANCER TRIALS SUPPORT UNIT (CTSU) ADDRESS AND CONTACT INFORMATION

**For regulatory requirements:**	**For patient enrollments:**	**For study data submission:**
Regulatory documentation must be submitted to the CTSU via the Regulatory Submission Portal:<br><br>(Sign in at www.ctsu.org, and select the Regulatory Submission sub-tab under the Regulatory tab.)<br><br>Institutions with patients waiting that are unable to use the Portal should alert the CTSU Regulatory Office immediately at 1-866-651-2878 to receive further instruction and support.<br><br>Contact the CTSU Regulatory Help Desk at 1-866-651-2878 for regulatory assistance.	Please refer to the patient enrollment section of the protocol for instructions on using the Oncology Patient Enrollment Network (OPEN) which can be accessed at https://www.ctsu.org/OPEN_SYSTEM/ or https://OPEN.ctsu.org.<br><br>Contact the CTSU Help Desk with any OPEN-related questions at ctsucontact@westat.com.	Data collection for this study will be done exclusively through Medidata Rave. Please see the data submission section of the protocol for further instructions.<br><br><u>Other Tools and Reports</u>: Institutions participating through the CTSU continue to have access to other tools and reports available on the SWOG Workbench via the SWOG website (www.swog.org).

The most current version of the **study protocol and all supporting documents** must be downloaded from the protocol-specific Web page of the CTSU Member Web site located at https://www.ctsu.org. Access to the CTSU members' website is managed through the Cancer Therapy and Evaluation Program - Identity and Access Management (CTEP-IAM) registration system and requires user log on with CTEP-IAM username and password. Permission to view and download this protocol and its supporting documents is restricted and is based on person and site roster assignment housed in the CTSU RSS.

CTSU sties should follow procedures outlined in the protocol for site registration. Patient Enrollment, Adverse Event Reporting, Data Submission (including ancillary studies), and Drug Procurement:

For patient eligibility questions contact the SWOG Statistics and Data Management Center by phone or email:

206-652-2267
S1400question@crab.org

For treatment or toxicity related questions contact S1400IMedicalquery@swog.org.

For non-clinical questions (i.e. unrelated to patient eligibility, treatment, or clinical data submission) contact the CTSU Help Desk by phone or e-mail:

CTSU General Information Line:
888-823-5923
S1400contact@westat.com

All calls and correspondence will be triaged to the appropriate CTSU representative.

The CTSU Web site is located at https://www.ctsu.org

CLOSED EFFECTIVE 04/03/2018

SCHEMA

Screening/Pre-Screening Registration

↓

Common Broad Platform CLIA Biomarker Profiling *

↓

If NOT eligible for biomarker driven sub-studies **

↓

S1400I
(Non-Match)

Arm 1
Nivolumab plus
Ipilimumab

↓

Progression Ø

Arm 2
Nivolumab

↓

Progression Ø

* Archival formalin-fixed paraffin-embedded (FFPE) tumor, fresh core needle biopsy if needed

** Notification of sub-study assignment will be provided by the SWOG Statistics and Data Management Center (see Section 11.0 in **S1400** for details).

Ø Upon progression (as defined in Section 10.2d in **S1400**), patients may be eligible for another sub-study. The new sub-study assignment will be determined by the SWOG Statistics and Data Management Center. (see Section 14.4).

CLOSED EFFECTIVE 04/23/2018

SWOG Authorized Document

1.0 OBJECTIVES

1.1 Primary Objective

To compare overall survival (OS) in patients with advanced stage refractory SCCA of the lung randomized to nivolumab plus ipilimumab versus nivolumab

1.2 Secondary Objective(s)

a. To compare investigator-assessed progression-free survival (IA-PFS) in patients with advanced stage refractory SCCA of the lung randomized to nivolumab plus ipilimumab versus nivolumab.

b. To compare the response rates (confirmed and unconfirmed, complete and partial) per RECIST 1.1 among patients randomized to receive nivolumab plus ipilimumab versus nivolumab.

c. To compare the response rates (confirmed only, complete and partial) per RECIST 1.1 among patients randomized to receive nivolumab plus ipilimumab versus nivolumab.

d. To evaluate the frequency and severity of toxicities associated with nivolumab plus ipilimumab versus nivolumab.

1.3 Translational Medicine Objectives

a. To evaluate if there is a differential treatment effect on OS, IA-PFS, and Response by tumor PD-L1 expression status.

b. S1400I Patient Reported Outcomes (PRO) Objectives

1. To compare symptom status by treatment arm using a validated PRO symptom measure, the M. D. Anderson Symptom Inventory (MDASI-LC) Severity Score.

2. To identify PRO-based symptoms prognostic for time to progression.

3. To develop a statistical model that identifies a PRO-based symptom score optimally prognostic for survival outcomes.

4. To evaluate functional status/interference of symptoms with life as a prognostic variable for time to progression.

5. To compare treatment-related toxicities by treatment arm at each assessment time.

6. To compare EQ-5D Index scores by treatment arm.

7. To collect psychometric information (reliability and validity) data for the Non-Small Cell Lung Cancer Symptom Assessment Questionnaire (NSCLC-SAQ).

CLOSED EFFECTIVE 04/23/2018

SWOG Authorized Document

2.0 BACKGROUND

Introduction

Non-small cell lung cancer (NSCLC) remains the leading cause of cancer-related mortality worldwide, accounting for approximately 18% of all cancer deaths. *(1)* Despite treatment with standard platinum-based doublet chemotherapy, patients with metastatic NSCLC have a median survival of approximately 10 months, and a 5-year survival rate of less than 5%. *(2)*

Immunotherapeutic approaches recently have demonstrated clinical efficacy in several cancer types, including melanoma and hormone-refractory prostate cancer. *(3)* Tumors may modulate and evade the host immune response through a number of mechanisms, including down regulation of tumor-specific antigen expression and presentation, secretion of anti-inflammatory cytokines, and upregulation of inhibitory ligands. T cell checkpoint regulators such as CTLA-4 and programmed death-1 (PD-1, CD279) are cell surface molecules that, when engaged by their cognate ligands, induce signaling cascades down-regulating T cell activation and proliferation. One proposed model by which therapeutic T cell checkpoint inhibitors derive antitumor activity is through breaking of immune tolerance to tumor cell antigens.

Nivolumab (BMS-936558) is a fully human, IgG4 (kappa) isotype mAb that binds PD-1 on activated immune cells and disrupts engagement of the receptor with its ligands PD-L1 (B7-H1/CD274) and PD-L2 (B7-DC/CD273), thereby abrogating inhibitory signals and augmenting the host antitumor response. In early clinical trials, nivolumab has demonstrated activity in several tumor types, including melanoma, renal cell cancer (RCC), and NSCLC. *(4)*

Nivolumab (Opdivo®) 3 mg/kg intravenously (IV) every 2 weeks was recently approved in the U.S. to treat patients with metastatic squamous cell NSCLC with progression on or after platinum-based chemotherapy. *(5,6)* The approval was based on the results of CA209017 (CHECKpoint pathway and nivoluMAb clinical Trial Evaluation-017 [CheckMate-017]), a randomized trial of nivolumab 3 mg/kg IV every 2 weeks versus docetaxel 75 mg/m^2 IV every 3 weeks in 272 patients. The median OS for patients in the nivolumab arm was 9.2 months versus 6 months for those in the docetaxel arm (HR = 0.59). Improvement in survival was observed for nivolumab regardless of PD-L1 expression, though there was a trend for better efficacy for those with PD-L1+ tumors. *(7)* A single arm trial (CA209063, CheckMate-063) of 117 patients with metastatic squamous cell NSCLC, with progression after platinum-based chemotherapy and at least one additional systemic regimen, showed a 15% overall objective response rate (ORR), of whom 59% had response durations of 6 months or longer. *(8)*

A second Phase 3 study, CA209057 was recently stopped at a preplanned interim analysis by the independent Data Monitoring Committee (DMC), demonstrating superior overall survival of nivolumab 3 mg/kg IV every 3 weeks versus docetaxel 75 mg/m2 IV every 3 weeks in patients with previously-treated non-squamous NSCLC with a 27% reduction in risk of death (HR = 0.73; P = 0.0015). *(9)* Interaction p-values reported for PD-L1 expression subgroups by each of the pre-defined expression levels suggested a clinically important signal of a predictive association. Nivolumab also significantly improved ORR versus docetaxel (P=0.0246), with ORR as high as 36% in subjects with PD-L1 expressing tumors. OS approximately doubled with nivolumab versus docetaxel across the PD-L1 expression continuum. In contrast, no difference in OS was seen between nivolumab and docetaxel when PD-L1 was not expressed in the tumor.

Nivolumab monotherapy has also been evaluated in first-line NSCLC, where it showed promising activity regardless of histology. *(10)* As in pretreated NSCLC, activity with nivolumab monotherapy is pronounced in PD-L1+ NSCLC.

In general, nivolumab also has been well tolerated to date, with a favorable safety profile relative to anticipated toxicities based on an immunostimulatory mechanism of action. *(11)*

CLOSED EFFECTIVE 04/25/2018

SWOG Authorized Document

Combining immunotherapeutic agents with different mechanisms of action offers the possibility of a synergistic response. PD-1 and CTLA-4 are both co-inhibitory molecules, but evidence suggests that they use distinct mechanisms to limit T cell activation. Preliminary indirect data from peripheral T cell assessments suggest that a given T-cell checkpoint inhibitor may modulate host immune cell phenotype rendering them more susceptible to alternate checkpoint inhibitors and thereby enhancing anti-tumor activity.

Preclinical testing data confirmed that the combination of PD-1 and CTLA-4 receptor blockade may improve antitumor activity. In vitro combinations of nivolumab plus ipilimumab increase IFN-γ production 2- to 7-fold over either agent alone in a mixed lymphocyte reaction. Increased antitumor activity of the combination was also observed in 3 of 5 syngeneic murine cancer models. In a murine melanoma vaccine model, blockade with either CTLA-4 or PD-1 antibodies increased the proportion of CTLA-4 and PD-1-expressing CD4/CD8 tumor infiltrating T effector cells, and dual blockade increased tumor infiltration of T effector cells and decreased intratumoral T regulatory cells, as compared to either agent alone. *(12)*

Several clinical studies of the combination of nivolumab with ipilimumab have been conducted or are planned. In a Phase 1 clinical study of the combination of nivolumab plus ipilimumab in advanced melanoma (CA209004), there was a 41% response rate, including a 17% complete response rate (CR). *(13)* A randomized Phase 2 study (CA209069) comparing nivolumab plus ipilimumab versus ipilimumab showed an ORR of 61%, including a 22% complete response rate, in previously untreated, advanced melanoma patients with BRAF wild-type mutation status, versus 11 % for ipilimumab alone. *(14)* In addition, the combination regimen decreased the risk of melanoma progression or death compared to ipilimumab alone by 60%. Similar results were also observed in BRAF mutation-positive patients.

In a Phase 1 study in patients with NSCLC (CA209012), the combination of nivolumab plus ipilimumab is being evaluated at several different doses and schedules (see Rationale for Dose Selection and Schedule below). While the schedule evaluated in melanoma was not found to be tolerable in NSCLC, the study has identified alternative schedules with acceptable tolerability profiles.

CA209227 (CheckMate-227) is a randomized, open-label Phase 3 trial of nivolumab monotherapy or nivolumab plus ipilimumab versus platinum doublet chemotherapy in previously untreated subjects with Stage IV or recurrent PD-L1+ NSCLC, and nivolumab plus ipilimumab (2 different schedules) versus platinum doublet chemotherapy in subjects with Stage IV or recurrent PD-L1- NSCLC. The objectives of this study are to determine whether:

- nivolumab monotherapy or nivolumab plus ipilimumab improves OS and PFS compared with platinum doublet chemotherapy in subjects with PD-L1+ Stage IV or recurrent NSCLC, and
- nivolumab plus ipilimumab improves OS and PFS compared with platinum doublet chemotherapy in subjects with PD-L1- Stage IV or recurrent NSCLC.

Rationale for dose selections and schedules of nivolumab in combination with ipilimumab.

Nivolumab plus ipilimumab combination has been also evaluated as first-line therapy in patients with advanced NSCLC. In CA209012, early combination cohorts evaluated 2 dosing schedules.

nivolumab 1 mg/kg + ipilimumab 3 mg/kg, q 3 weeks x4, followed by nivolumab 3 mg/kg q 2 weeks (n=24);

nivolumab 3 mg/kg + ipilimumab 1 mg/kg, q 3 weeks x4, followed by nivolumab 3 mg/kg q 2 weeks (n=25)

These regimens resulted in significant toxicity, with 39% of patients discontinuing treatment due to a treatment-related adverse event.

CLOSED EFFECTIVE 04/23/2018

SWOG Authorized Document

Thus, additional combination cohorts were initiated, using lower doses of both nivolumab and ipilimumab, or less frequent dosing of ipilimumab. Data from these cohorts demonstrate that both nivolumab 1 mg/kg + ipilimumab 1 mg/kg q 3 weeks with nivolumab maintenance 3 mg/kg q2w, as well as ipilimumab at 1 mg/kg q6w is tolerable, when given with nivolumab 3 mg/kg q2w.

Overall, the safety data are not dissimilar to what has been observed with nivolumab alone (i.e., in CA209012). Of particular note, the rate of discontinuation due to drug-related AEs was 13% and 11% in these combinations arms compared to 10% in the nivolumab monotherapy arm.

Table 1: Treatment-related adverse events from selected cohorts in CA209012

Arm	No. Subjects/ arm	Follow-up time (median, wks)	No. Subjects still on treatment	No. Subjects with drug-related AEs	No. Subjects with Grade 3-4 drug-related AEs	No. subjects d/c due to drug-related AEs (all grades
[a]	31	57	9 (29%)	23 (74%)	9 (29%)	4 (13%)
[b]	37	18	19 (51%)	20 (54%)	9 (24%)	4 (11%)
[c]	52	62	5 (10%)	37 (71%)	10 (19%)	5 (10%)

[a] nivolumab 1 mg/kg plus ipilimumab 1 mg/kg every 3 weeks x 4, followed by nivolumab 3 mg/kg every 2 weeks
[b] nivolumab 3 mg/kg every 2 weeks plus ipilimumab 1 mg/kg every 6 weeks
[c] nivolumab 3 mg/kg every 2 weeks

The significant toxicity observed in some of the other original cohorts resulted in limited exposure to treatment as demonstrated by the high discontinuation rate. Nonetheless, clinical activity was observed.

Table 2: Clinical Activity of Nivolumab/Ipilimumab Combination by Schedule and PD-L1 Status, using 1% Cutoff

	ORR % (n/N)	OS-12 mo	mOS (mos)	mPFS (wks)	PFS-24wk
**Nivolumab 1 mg/kg + Ipilimumab 3 mg/kg q3w x4, followed by Nivolumab 3 mg/kg q2w (arms G/H)**					
PD-L1+	17% (2/12)	83%	NR[a]	33.7	51%
PD-L1-	11% (1/9)	56%	19.8	12.4	38%
PD-L1 unk	0 (0/3)	33%	10.9	8.7	NC[b]
**Nivolumab 3 mg/kg + Ipilimumab 1 mg/kg q3w x4, followed by Nivolumab 3 mg/kg q2w (arms I/J)**					
PD-L1+	25% (4/16)	50%	16.5	14.4	33%
PD-L1-	0 (0/6)	33%	4.4	10.6	50%
PD-L1 unk	33%	33%	10.8	15.4	100%

[a] NR= not reached
[b] NC= not calculated

Based on these data, nivolumab (3 mg/kg every 2 weeks, i.e., the FDA approved dose in pretreated squamous NSCLC) with the highest dose and frequency of ipilimumab feasible (1 mg/kg every 6 weeks) will be evaluated.

<u>HIV, HBV, and HCV Exclusion Rationale</u>

Patients with known HIV/HBV/HCV infection will be excluded from this study. Immune checkpoint inhibition is a relatively new class of therapy. While HIV patients have been studied with anti-CTLA4 therapy, the unique interactions of PD-1/PD-L1 inhibitors and HIV may be difficult to predict and require further study. Since many of these patients may already be highly suppressed with

CLOSED EFFECTIVE 04/25/2018

SWOG Authorized Document

antiretroviral therapy, standard assays may not be sensitive enough to detect a safety signal of increasing viral load. Thus, these patients may need to be studied in trials with access to such sensitive assays to better inform drug development in this particular population. The impact of PD-1/PD-L1 inhibition in HIV patients may be beneficial, which is of great interest in drug development in the field of chronic infections generally. We have elected not to complicate our trial with this added and important question at this time.

3.0 DRUG INFORMATION

For information regarding Investigator's Brochures, please refer to SWOG Policy 15.

For this sub-study, nivolumab plus ipilimumab is investigational and is being provided under an IND held by SWOG. For INDs filed by SWOG, the protocol serves as the Investigator Brochure for the performance of the protocol. In such instances, submission of the protocol to the IRB should suffice for providing the IRB with information about the drug. However, in cases where the IRB insists on having the official Investigator Brochure from the company, requests may be submitted to the CTSU website by completing the CTSU Request for Clinical Brochure Form under the study's CTSU abstract page > Documents > Pharmacy Tab.

3.1 Nivolumab (BMS-936558, MDX1106, Opdivo®) (NSC # 748726) (IND-119672)

a. PHARMACOLOGY

Mode of Action: Nivolumab targets the programmed death–1 (PD-1, cluster of differentiation 279 [CD279]) cell surface membrane receptor. PD-1 is a negative regulatory receptor expressed by activated T and B lymphocytes. Binding of PD-1 to its ligands, programmed death–ligand 1 (PD-L1) and 2 (PD-L2), results in the down-regulation of lymphocyte activation. Nivolumab inhibits the binding of PD-1 to PD-L1 and PD-L2. Inhibition of the interaction between PD-1 and its ligands promotes immune responses and antigen-specific T-cell responses to both foreign antigens as well as self-antigens.

b. PHARMACOKINETICS

1. Distribution: Nivolumab has linear pharmacokinetics after single and multiple dosing within the range 0.1 mg/kg to 10 mg/kg. The volume distribution (Vd) is 8L.

2. Elimination: Clearance is independent of dose in the range 0.1 mg/kg to 10 mg/kg. The total body clearance is 9.5 mL/hr, and the elimination half-life of is approximately 26.7 days. Body weight normalized dosing showed approximately constant trough concentrations over a wide range of body weights.

c. ADVERSE EFFECTS

1. Adverse Effects: The Comprehensive Adverse Events and Potential Risks list (CAEPR) provides a single list of reported and/or potential adverse events (AE) associated with an agent using a uniform presentation of events by body system. Refer to the 'CTEP, NCI Guidelines: Adverse Event Reporting Requirements' http://ctep.cancer.gov/protocolDevelopment/electronic_applications/docs/aeguidelines.pdf for further clarification. *Frequency is provided based on*

CLOSED EFFECTIVE 04/23/2018

SWOG Authorized Document

2069 patients. Below is the CAEPR for BMS-936558 (Nivolumab, MDX-1106).

Version 2.3, June 18, 2018[1]

Adverse Events with Possible Relationship to BMS-936558 (Nivolumab, MDX-1106) (CTCAE 5.0 Term) [n= 2069]		
**Likely (>20%)**	**Less Likely (<=20%)**	**Rare but Serious (<3%)**
BLOOD AND LYMPHATIC SYSTEM DISORDERS		
	Anemia	
CARDIAC DISORDERS		
		Cardiac disorders - Other (cardiomyopathy)
		Myocarditis
		Pericardial tamponade[2]
		Pericarditis
ENDOCRINE DISORDERS		
	Adrenal insufficiency[3]	
	Hypophysitis[3]	
	Hyperthyroidism[3]	
	Hypothyroidism[3]	
EYE DISORDERS		
		Blurred vision
		Dry eye
		Eye disorders - Other (diplopia)[3]
		Eye disorders - Other (Graves ophthalmopathy)[3]
		Eye disorders - Other (optic neuritis retrobulbar)[3]
	Uveitis	
GASTROINTESTINAL DISORDERS		
	Abdominal pain	
	Colitis[3]	
		Colonic perforation[3]
	Diarrhea	
	Dry mouth	
		Gastritis
		Mucositis oral
	Nausea	
	Pancreatitis[4]	
GENERAL DISORDERS AND ADMINISTRATION SITE CONDITIONS		
Fatigue		
	Fever	
	Injection site reaction	
IMMUNE SYSTEM DISORDERS		

CLOSED

Adverse Events with Possible Relationship to BMS-936558 (Nivolumab, MDX-1106) (CTCAE 5.0 Term) [n= 2069]		
**Likely (>20%)**	**Less Likely (<=20%)**	**Rare but Serious (<3%)**
		Allergic reaction[3]
		Autoimmune disorder[3]
		Cytokine release syndrome[5]
		Immune system disorders - Other (GVHD in the setting of allotransplant)[3,6]
		Immune system disorders - Other (sarcoid granuloma)[3]
INJURY, POISONING AND PROCEDURAL COMPLICATIONS		
	Infusion related reaction[7]	
INVESTIGATIONS		
	Alanine aminotransferase increased[3]	
	Aspartate aminotransferase increased[3]	
	Blood bilirubin increased[3]	
	Creatinine increased	
	Lipase increased	
	Lymphocyte count decreased	
	Neutrophil count decreased	
	Platelet count decreased	
	Serum amylase increased	
METABOLISM AND NUTRITION DISORDERS		
	Anorexia	
		Hyperglycemia
		Metabolism and nutrition disorders - Other (diabetes mellitus with ketoacidosis)[3]
MUSCULOSKELETAL AND CONNECTIVE TISSUE DISORDERS		
	Arthralgia	
		Musculoskeletal and connective tissue disorder - Other (polymyositis)
		Myositis
		Rhabdomyolysis

CLOSED

SWOG Authorized Document

Adverse Events with Possible Relationship to BMS-936558 (Nivolumab, MDX-1106) (CTCAE 5.0 Term) [n= 2069]		
**Likely (>20%)**	**Less Likely (<=20%)**	**Rare but Serious (<3%)**
NERVOUS SYSTEM DISORDERS		
		Encephalopathy[3]
		Facial nerve disorder[3]
		Guillain-Barre syndrome[3]
		Myasthenia gravis[3]
		Nervous system disorders - Other (demyelination myasthenic syndrome)
		Nervous system disorders - Other (encephalitis)[3]
		Nervous system disorders - Other (meningoencephalitis)
		Nervous system disorders - Other (meningoradiculitis)[3]
		Nervous system disorders - Other (myasthenic syndrome)
		Peripheral motor neuropathy
		Peripheral sensory neuropathy
		Reversible posterior leukoencephalopathy syndrome[3]
RENAL AND URINARY DISORDERS		
		Acute kidney injury[3]
RESPIRATORY, THORACIC AND MEDIASTINAL DISORDERS		
	Pleural effusion[3]	
	Pneumonitis[3]	
		Respiratory, thoracic and mediastinal disorders - Other (bronchiolitis obliterans with organizing pneumonia)[3]
SKIN AND SUBCUTANEOUS TISSUE DISORDERS		
		Erythema multiforme[3]
	Pruritus[3]	
	Rash maculo-papular[3]	

CLOSED E

SWOG Authorized Document

Adverse Events with Possible Relationship to BMS-936558 (Nivolumab, MDX-1106) (CTCAE 5.0 Term) [n= 2069]		
**Likely (>20%)**	**Less Likely (<=20%)**	**Rare but Serious (<3%)**
		Skin and subcutaneous disorders -Other (bullous pemphigoid)
	Skin and subcutaneous disorders - Other (Sweet's Syndrome)[3]	
	Skin hypopigmentation[3]	
		Stevens-Johnson syndrome
		Toxic epidermal necrolysis

1 This table will be updated as the toxicity profile of the agent is revised. Updates will be distributed to all Principal Investigators at the time of revision. The current version can be obtained by contacting PIO@CTEP.NCI.NIH.GOV. Your name, the name of the investigator, the protocol and the agent should be included in the e-mail.

2 Pericardial tamponade may be related to possible inflammatory reaction at tumor site.

3 BMS-936558 (Nivolumab, MDX-1106) being a member of class of agents involved in the inhibition of "immune checkpoints", may result in severe and possibly fatal immune-mediated adverse events probably due to T-cell activation and proliferation. This may result in autoimmune disorders that can include (but are not limited to) autoimmune hemolytic anemia, acquired anti-factor VIII immune response, autoimmune aseptic meningitis, autoimmune hepatitis, autoimmune nephritis, autoimmune neuropathy, autoimmune thyroiditis, bullous pemphigoid, exacerbation of Churg-Strauss Syndrome, drug rash with eosinophilia systemic symptoms [DRESS] syndrome, facial nerve disorder (facial nerve paralysis), limbic encephalitis, hepatic failure, pure red cell aplasia, pancreatitis, ulcerative and hemorrhagic colitis, endocrine disorders (e.g., autoimmune thyroiditis, hyperthyroidism, hypothyroidism, autoimmune hypophysitis/hypopituitarism, thyrotoxicosis, and adrenal insufficiency), sarcoid granuloma, myasthenia gravis, polymyositis, and Guillain-Barre syndrome.

4 Pancreatitis may result in increased serum amylase and/or more frequently lipase.

5 Cytokine release syndrome may manifest as hemophagocytic lymphohistiocytosis with accompanying fever and pancytopenia.

6 Complications including hyperacute graft-versus-host disease (GVHD), some fatal, have occurred in patients receiving allo stem cell transplant (SCT) after receiving BMS-936558 (Nivolumab, MDX-1106). These complications may occur despite intervening therapy between receiving BMS-936558 (Nivolumab, MDX-1106) and allo-SCT.

7 Infusion reactions, including high-grade hypersensitivity reactions which have been observed following administration of nivolumab, may manifest as fever, chills, shakes, itching, rash, hypertension or

CLOSED EFFECTIVE 04/23/2018

SWOG Authorized Document

hypotension, or difficulty breathing during and immediately after administration of nivolumab.

Adverse events reported on BMS-936558 (Nivolumab, MDX-1106) trials, but for which there is insufficient evidence to suggest that there was a reasonable possibility that BMS-936558 (Nivolumab, MDX-1106) caused the adverse event:

BLOOD AND LYMPHATIC SYSTEM DISORDERS - Leukocytosis
CARDIAC DISORDERS - Atrial fibrillation; Atrioventricular block complete; Heart failure; Ventricular arrhythmia
EAR AND LABYRINTH DISORDERS - Vestibular disorder
EYE DISORDERS - Eye disorders - Other (iridocyclitis); Optic nerve disorder; Periorbital edema
GASTROINTESTINAL DISORDERS - Constipation; Duodenal ulcer; Flatulence; Gastrointestinal disorders - Other (mouth sores); Vomiting
GENERAL DISORDERS AND ADMINISTRATION SITE CONDITIONS - Chills; Edema limbs; Malaise; Pain
HEPATOBILIARY DISORDERS - Bile duct stenosis
IMMUNE SYSTEM DISORDERS - Anaphylaxis; Immune system disorders - Other (autoimmune thrombotic microangiopathy); Immune system disorders - Other (limbic encephalitis)
INFECTIONS AND INFESTATIONS - Bronchial infection; Lung infection; Sepsis; Upper respiratory infection
INVESTIGATIONS - Blood lactate dehydrogenase increased; GGT increased; Investigations - Other (protein total decreased); Lymphocyte count increased; Weight loss
METABOLISM AND NUTRITION DISORDERS - Dehydration; Hyperuricemia; Hypoalbuminemia; Hypocalcemia; Hyponatremia; Hypophosphatemia
MUSCULOSKELETAL AND CONNECTIVE TISSUE DISORDERS - Back pain; Musculoskeletal and connective tissue disorder - Other (musculoskeletal pain); Musculoskeletal and connective tissue disorder - Other (polymyalgia rheumatica); Myalgia; Pain in extremity
NEOPLASMS BENIGN, MALIGNANT AND UNSPECIFIED (INCL CYSTS AND POLYPS) - Neoplasms benign, malignant and unspecified (incl cysts and polyps) - Other (histiocytic necrotizing lymphadenitis)
NERVOUS SYSTEM DISORDERS - Dizziness; Headache; Intracranial hemorrhage
PSYCHIATRIC DISORDERS - Insomnia
RENAL AND URINARY DISORDERS - Hematuria; Renal and urinary disorders - Other (tubulointerstitial nephritis)
RESPIRATORY, THORACIC AND MEDIASTINAL DISORDERS - Bronchospasm; Cough; Dyspnea; Hypoxia
SKIN AND SUBCUTANEOUS TISSUE DISORDERS - Alopecia; Dry skin; Hyperhidrosis; Pain of skin; Photosensitivity; Rash acneiform; Skin and subcutaneous tissue disorders - Other (rosacea)
VASCULAR DISORDERS - Flushing; Hypertension; Hypotension; Vasculitis

Note: BMS-936558 (Nivolumab, MDX-1106) in combination with other agents could cause an exacerbation of any adverse event currently known to be caused by the other agent, or the combination may result in events never previously associated with either agent.

2. Pregnancy and Lactation:

CLOSED EFFECTIVE 04/23/2018

SWOG Authorized Document

Pregnancy: Adverse events were observed in animal reproduction studies. Nivolumab may be expected to cross the placenta; effects to the fetus may be greater in the second and third trimesters. Based on its mechanism of action, nivolumab is expected to cause fetal harm if used during pregnancy. Women of childbearing potential (WOCBP) receiving nivolumab must continue contraception for a period of 5 months after the last dose of nivolumab. Men receiving nivolumab and who are sexually active with WOCBP must continue contraception for a period of 7 months after the last dose of nivolumab.

Lactation: It is not known if nivolumab is excreted into breast milk. Due to the potential for serious adverse reactions in the nursing infant, the manufacturer recommends women to discontinue breastfeeding during treatment with nivolumab.

3. Potential Drug Interactions: The indirect drug-drug interaction potential of nivolumab was assessed using systemic cytokine modulation data for cytokines known to modulate CYP enzymes. There were no meaningful changes in cytokines known to have indirect effects on CYP enzymes across all dose levels of nivolumab. This lack of cytokine modulation suggests that nivolumab has no or low potential for modulating CYP enzymes, thereby indicating a low risk of therapeutic protein-drug interaction.

d. DOSING & ADMINISTRATION

See Section 7.0 Treatment Plan

Nivolumab injection is to be administered as a 30 minute IV infusion through a 0.2 micron to 1.2 micron pore size, low-protein binding membrane in-line filter. DO NOT administer as IV push or bolus injection.

e. HOW SUPPLIED

1. Description: Nivolumab Injection is a clear to opalescent, colorless to pale yellow liquid; light (few) particulates may be present. The drug product is a sterile, nonpyrogenic, single-use, isotonic aqueous solution formulated in sodium citrate dihydrate, sodium chloride, mannitol, diethylenetriaminepentacetic acid (pentetic acid) and polysorbate 80 (Tween® 80), and water for injection. Dilute solutions of hydrochloric acid and/or sodium hydroxide may be used for pH adjustment (pH 5.5-6.5).

2. How Supplied: Nivolumab is supplied by Bristol-Myers Squibb and distributed by the Pharmaceutical Management Branch, CTEP/DCTD/NCI as 100 mg vials (10 mg/mL) with a 0.7 mL overfill. It is supplied in 10 mL type I flint glass vials, with fluoropolymer film-laminated rubber stoppers and aluminum seals.

f. STORAGE, PREPARATION & STABILITY

1. Storage: Vials of nivolumab injection must be stored at 2°-8°C (36°-46°F) and protected from light and freezing. The unopened vials can be stored at room temperature (up to 25°C, 77°F) and room light for up to 48 hours.

If a storage temperature excursion is identified, promptly return Nivolumab to 2° - 8°C and quarantine the supplies. Provide a detailed report of the excursion (including documentation of temperature monitoring and

CLOSED EFFECTIVE 04/23/2018

SWOG Authorized Document

duration of the excursion) to PMBAfterHours@mail.nih.gov for determination of suitability.

2. Preparation: Nivolumab injection can be infused undiluted (10 mg/mL) or diluted with 0.9% Sodium Chloride Injection, USP or 5% Dextrose. When the dose is based on patient weight (i.e., mg/kg), nivolumab injection can be infused undiluted or diluted to protein concentrations as low as 0.35 mg/mL. When the dose is fixed (eg, 240 mg, 360 mg, or 480 mg flat dose), nivolumab injection can be infused undiluted or diluted so as not to exceed a total infusion volume of 160 mL. For patients weighing less than 40 kilograms (kg), the total volume of infusion must not exceed 4 mL per kg of patient weight. During drug product preparation and handling, vigorous mixing or shaking is to be avoided.

3. Compatibility: Nivolumab infusions are compatible with polyvinyl chloride (PVC) or polyolefin containers and infusion sets, and glass bottles.

4. Stability: Shelf-life stability studies of the intact vials are ongoing.

   The administration of undiluted and diluted solutions of nivolumab must be completed within 24 hours of preparation. If not used immediately, the infusion solution may be stored up to 24 hours in a refrigerator at 2°-

   8°C (36°-46°F) and a maximum of 8 hours of the total 24 hours can be at room temperature (up to25°C, 77°F) and room light. The maximum 8-hour period under room temperature and room light conditions includes the product administration period.

   CAUTION: The single-use dosage form contains no antibacterial preservative or bacteriostatic agent. Therefore, it is advised that the product be discarded 8 hours after initial entry.

g. DRUG ORDERING & ACCOUNTABILITY

1. Drug ordering: Study specific supplies will be provided to sites once a patient has been randomized. Starter supplies will not be provided. NCI-supplied agents may be requested by the Principal Investigator (or their authorized designee) at each participating institution. Pharmaceutical Management Branch (PMB) policy requires that drug be shipped directly to the institution where the patient is to be treated. PMB does not permit the transfer of agents between institutions (unless prior approval from PMB is obtained). The CTEP assigned protocol number (**S1400I**) must be used for ordering all CTEP supplied investigational agents. The responsible investigator at each participating institution must be registered with CTEP, DCTD through an annual submission of FDA Form 1572 and a CV. If there are several participating investigators at one institution, CTEP-supplied investigational agents for the study should be ordered under the name of one lead investigator at that institution. Active CTEP-registered investigators and investigator-designated shipping designees and ordering designees can submit agent requests through the PMB Online Agent Order Processing (OAOP) application <https://eapps-ctep.nci.nih.gov/OAOP/pages/login.jspx>. Access to OAOP requires the establishment of a CTEP Identity and Access Management (IAM) account < https://ctepcore.nci.nih.gov/iam/index.jsp > and the maintenance of an "active" account status and a "current" password.

2. Drug Handling and Accountability (NCI logs or other)

CLOSED EFFECTIVE 04/23/2018

a. Drug Accountability: The investigator, or a responsible party designated by the investigator, must maintain a careful record of the receipt, disposition, and return of all drugs received from the PMB using the Drug Accountability Record Form available on the NCI home page (http://ctep.cancer.gov).

b. Electronic logs are allowed as long as a print version of the log process is the exact same appearance as the current NCI DARF. If the trial is a placebo control trial – indicate that separate DARFs are needed for each patient to also include the placebo drug supply.

3. Drug return and/or disposition instruction (include forms if needed)

a. All undispensed drug supplies should be returned to the PMB. When it is necessary to return study drug (e.g., sealed bottles remaining when PMB sends a stock recovery letter), investigators should return the study drug to the PMB using the NCI Return Agent Form available on the NCI home page (http://ctep.cancer.gov).

b. Drug expiration: (If packaging does not have expiration date, check with drug ordering designee and/or PI at site to confirm receipt of ongoing stability testing letter from NCI when internal drug audits are being performed on a quarterly basis. If packaging has expiration date, indicate drug expiration date on the DARF under Manufacturer and Lot # and use the drug lots with shorter expiration date first).

4. Contact Information and Useful Links

Questions about drug orders, transfers, returns or accountability should be addressed to the PMB by calling 240/276-6575 Monday through Friday between 8:30 am and 4:30 pm Eastern Time or by email: PMBAfterHours@mail.nih.gov.

- CTEP Forms, Templates, Documents: http://ctep.cancer.gov/forms/
- RCR Help Desk: RCRHelpDesk@nih.gov
- PMB policies and guidelines: http://ctep.cancer.gov/branches/pmb/agent_management.htm
- PMB Online Agent Order Processing (OAOP) application: https://eapps-ctep.nci.nih.gov/OAOP/pages/login.jspx
- CTEP Identity and Access Management (IAM) account: https://ctepcore.nci.nih.gov/iam/index.jsp
- CTEP Associate Registration and IAM account help: ctepreghelp@ctep.nci.nih.gov

3.2 Ipilimumab (BMS-734016, MDX-010, YERVOY®) (NSC 732442) (IND-119672)

a. PHARMACOLOGY

Mechanism of Action: Cytotoxic T-lymphocyte antigen-4 CTLA-4 is a negative regulator of T-cell activity. Ipilimumab is a full human monoclonal immunoglobin (lg) antibody that binds to CTLA-4 and blocks the interaction of CTLA-4 with its ligands, CD80/CD86. Blockade of CTLA-4 has been shown to augment T-cell activation and proliferation, including the activation and proliferation of tumor

CLOSED EFFECTIVE 04/26/2016

SWOG Authorized Document

infiltrating T-effector cells. Inhibition of CTLA-4 signaling can also reduce T-regulatory cell function, which may contribute to a general increase in T cell responsiveness, including the anti-tumor immune response.

b. PHARMACOKINETICS

1. Absorption: No formal pharmacokinetic drug interaction studies have been conducted with ipilimumab. Ipilimumab is not expected to have pharmacokinetic drug-drug interactions, since it is not metabolized by CYP450 or other drug metabolizing enzymes.

2. Distribution: Ipilimumab is confined mainly to the extracellular fluid. Peak concentration (Cmax), trough concentration (Cmin), and area under the plasma concentration versus time curve (AUC) of ipilimumab increased dose proportionally within the dose range examined ipilimumab is confined mainly to the extracellular fluid. Peak concentration (Cmax), trough concentration (Cmin), and area under the plasma concentration versus time curve (AUC) of ipilimumab increased dose proportionally within the dose range examined. Based on population pharmacokinetic analysis, the mean volume of distribution (% coefficient of variation) at steady state was 7.47 liters (10%)

3. Metabolism: Not applicable. Monoclonal antibodies are usually degraded into amino acids and small peptides, independently from CYP450 or other drug-metabolizing enzymes.

4. Elimination: Clearance increased with body weight, but no dose adjustment is required with dosing on a mg/kg basis. Upon repeated dosing every 3 weeks, the clearance (CL) of ipilimumab was found to be time-invariant, and systemic accumulation was 1.5-fold or less. The mean value (% coefficient of variation) generated through population pharmacokinetic analysis for the terminal half-life (t1/2) was 15.4 days (34%) and for CL was 16.8 mL/h (38%).

c. ADVERSE EFFECTS

1. Adverse Effects: The Comprehensive Adverse Events and Potential Risks list (CAEPR) provides a single list of reported and/or potential adverse events (AE) associated with an agent using a uniform presentation of events by body system. Refer to the 'CTEP, NCI Guidelines: Adverse Event Reporting Requirements' http://ctep.cancer.gov/protocolDevelopment/electronic_applications/docs/aeguidelines.pdf for further clarification. Frequency is provided based on 2678 patients. Below is the CAEPR for Ipilimumab (MDX-010).

Version 2.10, March 29, 2019[1]

Adverse Events with Possible Relationship to Ipilimumab (MDX-010) (CTCAE 5.0 Term) [n= 2678]		
**Likely (>20%)**	**Less Likely (<=20%)**	**Rare but Serious (<3%)**
BLOOD AND LYMPHATIC SYSTEM DISORDERS		
		Blood and lymphatic system disorders - Other (acquired hemophilia)
CARDIAC DISORDERS		

CLOSED EFFECTIVE 04/25/2018

SWOG Authorized Document

Adverse Events with Possible Relationship to Ipilimumab (MDX-010) (CTCAE 5.0 Term) [n= 2678]		
**Likely (>20%)**	**Less Likely (<=20%)**	**Rare but Serious (<3%)**
	Atrial fibrillation	
		Myocarditis[2]
		Pericardial effusion
EAR AND LABYRINTH DISORDERS		
	Hearing impaired	
ENDOCRINE DISORDERS		
	Adrenal insufficiency[2]	
	Hyperthyroidism[2]	
	Hypophysitis[2]	
	Hypopituitarism[2]	
	Hypothyroidism[2]	
	Testosterone deficiency[2]	
EYE DISORDERS		
	Eye disorders - Other (episcleritis)[2]	
	Uveitis[2]	
GASTROINTESTINAL DISORDERS		
	Abdominal pain	
	Colitis[2]	
		Colonic perforation[3]
	Constipation	
Diarrhea		
	Enterocolitis	
	Esophagitis	
		Ileus
Nausea		
	Pancreatitis[2]	
	Vomiting	
GENERAL DISORDERS AND ADMINISTRATION SITE CONDITIONS		
	Chills	
Fatigue		
	Fever	
		General disorders and administration site conditions - Other (systemic inflammatory response syndrome [SIRS])
		Multi-organ failure
HEPATOBILIARY DISORDERS		
	Hepatobiliary disorders - Other (hepatitis)[2]	
IMMUNE SYSTEM DISORDERS		
	Autoimmune disorder[2]	
		Immune system disorders - Other (GVHD in the setting of allotransplant)[4]
INFECTIONS AND INFESTATIONS		
		Infections and infestations - Other (aseptic meningitis)[2]

CLOSED

Adverse Events with Possible Relationship to Ipilimumab (MDX-010) (CTCAE 5.0 Term) [n= 2678]		
**Likely (>20%)**	**Less Likely (<=20%)**	**Rare but Serious (<3%)**
INJURY, POISONING AND PROCEDURAL COMPLICATIONS		
	Infusion related reaction	
INVESTIGATIONS		
	Alanine aminotransferase increased	
	Aspartate aminotransferase increased	
		Lymphocyte count decreased
	Neutrophil count decreased	
	Weight loss	
METABOLISM AND NUTRITION DISORDERS		
	Anorexia	
	Dehydration	
	Hyperglycemia	
		Metabolism and nutrition disorders - Other (exacerbation of pre-existing diabetes mellitus)
MUSCULOSKELETAL AND CONNECTIVE TISSUE DISORDERS		
	Arthralgia	
	Arthritis	
		Generalized muscle weakness
	Musculoskeletal and connective tissue disorder - Other (polymyositis)[2]	
NERVOUS SYSTEM DISORDERS		
		Ataxia
	Facial nerve disorder[2]	
	Guillain-Barre syndrome[2]	
	Headache	
	Myasthenia gravis[2]	
		Nervous system disorders – Other (immune-mediated encephalitis) [2]
		Peripheral motor neuropathy
		Peripheral sensory neuropathy
	Trigeminal nerve disorder	
PSYCHIATRIC DISORDERS		
		Psychiatric disorders - Other (mental status changes)
RENAL AND URINARY DISORDERS		
	Acute kidney injury	

CLOSED EFFECTIVE

SWOG Authorized Document

Adverse Events with Possible Relationship to Ipilimumab (MDX-010) (CTCAE 5.0 Term) [n= 2678]		
**Likely (>20%)**	**Less Likely (<=20%)**	**Rare but Serious (<3%)**
	Renal and urinary disorders - Other (granulomatous tubulointerstitial nephritis)	
RESPIRATORY, THORACIC AND MEDIASTINAL DISORDERS		
	Pneumonitis	
		Respiratory failure
		Respiratory, thoracic and mediastinal disorders - Other (bronchiolitis obliterans with organizing pneumonia)
		Respiratory, thoracic and mediastinal disorders - Other (lung infiltration)
SKIN AND SUBCUTANEOUS TISSUE DISORDERS		
		Erythema multiforme
	Pruritus	
Rash maculo-papular		
	Skin and subcutaneous disorders - Other (Sweet's syndrome)	
		Stevens-Johnson syndrome
		Toxic epidermal necrolysis
	Urticaria	
VASCULAR DISORDERS		
	Hypotension	

[1] This table will be updated as the toxicity profile of the agent is revised. Updates will be distributed to all Principal Investigators at the time of revision. The current version can be obtained by contacting PIO@CTEP.NCI.NIH.GOV. Your name, the name of the investigator, the protocol and the agent should be included in the e-mail.

[2] Ipilimumab can result in severe and fatal immune-mediated adverse events probably due to T-cell activation and proliferation. These can include (but are not limited to) autoimmune hemolytic anemia, acquired anti-factor VIII immune response, autoimmune aseptic meningitis, autoimmune hepatitis, autoimmune thyroiditis, hepatic failure, pure red cell aplasia, pancreatitis, ulcerative and hemorrhagic colitis, endocrine disorders (e.g., autoimmune thyroiditis, hyperthyroidism, hypothyroidism, autoimmune hypophysitis/hypopituitarism, and adrenal insufficiency), ocular manifestations (e.g., uveitis, iritis, conjunctivitis, blepharitis, and episcleritis), sarcoid granuloma, myasthenia gravis, polymyositis, and Guillain-Barre syndrome. The majority of these reactions manifested early during treatment; however, a minority occurred weeks to months after discontinuation of ipilimumab especially with the initiation of additional treatments.

[3] Late bowel perforations have been noted in patients receiving MDX-010 (ipilimumab) in association with subsequent IL-2 therapy.

CLOSED EFFECTIVE

SWOG Authorized Document

[4] Complications including hyperacute graft-versus-host disease (GVHD), may occur in patients receving allo stem cell transplant (SCT) after receiving Ipilimumab (MDX-010). These complications may occur despite intervening therapy between receiving Ipilimumab (MDX-010) and allo-SCT.

[5] In rare cases diplopia (double vision) has occurred as a result of muscle weakness (Myasthenia gravis).

[6] Gastrointestinal hemorrhage includes Anal hemorrhage, Cecal hemorrhage, Colonic hemorrhage, Duodenal hemorrhage, Esophageal hemorrhage, Esophageal varices hemorrhage, Gastric hemorrhage, Hemorrhoidal hemorrhage, Ileal hemorrhage, Intra-abdominal hemorrhage, Jejunal hemorrhage, Lower gastrointestinal hemorrhage, Oral hemorrhage, Pancreatic hemorrhage, Rectal hemorrhage, Retroperitoneal hemorrhage, and Upper gastrointestinal hemorrhage under the GASTROINTESTINAL DISORDERS SOC.

[7] Infection includes all 75 sites of infection under the INFECTIONS AND INFESTATIONS SOC.

Adverse events reported on ipilimumab (MDX-010) trials, but for which there is insufficient evidence to suggest that there was a reasonable possibility that ipilimumab (MDX-010) caused the adverse event:

BLOOD AND LYMPHATIC SYSTEM DISORDERS - Anemia; Blood and lymphatic system disorders - Other (pure red cell aplasia)[2]; Febrile neutropenia
CARDIAC DISORDERS - Conduction disorder; Restrictive cardiomyopathy
EYE DISORDERS - Extraocular muscle paresis[5]; Eye disorders - Other (retinal pigment changes)
GASTROINTESTINAL DISORDERS - Colonic ulcer; Dyspepsia; Dysphagia; Gastrointestinal disorders - Other (gastroenteritis); Gastrointestinal hemorrhage[6]; Proctitis
GENERAL DISORDERS AND ADMINISTRATION SITE CONDITIONS - Flu like symptoms; Non-cardiac chest pain
HEPATOBILIARY DISORDERS - Hepatic failure[2]
IMMUNE SYSTEM DISORDERS - Allergic reaction
INFECTIONS AND INFESTATIONS - Infection[7]
INVESTIGATIONS - Creatinine increased; Investigations - Other (rheumatoid factor); Lipase increased; Platelet count decreased; Serum amylase increased; White blood cell decreased
METABOLISM AND NUTRITION DISORDERS - Tumor lysis syndrome
MUSCULOSKELETAL AND CONNECTIVE TISSUE DISORDERS - Back pain; Joint range of motion decreased; Myalgia; Pain in extremity
NEOPLASMS BENIGN, MALIGNANT AND UNSPECIFIED (INCL CYSTS AND POLYPS) - Tumor pain
NERVOUS SYSTEM DISORDERS - Dizziness; Dysphasia; Ischemia cerebrovascular; Seizure
PSYCHIATRIC DISORDERS - Anxiety; Confusion; Depression; Insomnia
RENAL AND URINARY DISORDERS - Proteinuria
RESPIRATORY, THORACIC AND MEDIASTINAL DISORDERS - Allergic rhinitis; Cough; Dyspnea; Laryngospasm
SKIN AND SUBCUTANEOUS TISSUE DISORDERS - Alopecia; Dry skin; Hyperhidrosis; Skin hypopigmentation
VASCULAR DISORDERS - Flushing; Hypertension; Vascular disorders - Other (temporal arteritis)

CLOSED EFFECTIVE 04/23/2018

SWOG Authorized Document

Note: Ipilimumab (MDX-010) in combination with other agents could cause an exacerbation of any adverse event currently known to be caused by the other agent, or the combination may result in events never previously associated with either agent.

2. Pregnancy and Lactation: There are no adequate and well-controlled studies of Ipilimumab in pregnant women. Use of Ipilimumab during pregnancy only if the potential benefit justifies the potential risk to the fetus. Human IgG1 is known to cross the placental barrier and ipilimumab is an IgG1; therefore, ipilimumab has the potential to be transmitted from the mother to the developing fetus.

   It is not known whether ipilimumab is secreted in human milk. Because many drugs are secreted in human milk and because of the potential for serious adverse reactions in nursing infants from ipilimumab, a decision should be made whether to discontinue nursing or to discontinue ipilimumab, taking into account the importance of ipilimumab to the mother.

3. Drug Interactions: No formal pharmacokinetic drug interaction studies have been conducted with ipilimumab. Ipilimumab is not expected to have pharmacokinetic drug-drug interactions, since it is not metabolized by CYP450 or other drug metabolizing enzymes

d. DOSING & ADMINISTRATION

See Section 7.0 Treatment Plan.

Ipilimumab injection is to be administered as an infusion with an in-line, sterile, nonpyrogenic, low-protein-binding filter (pore size of 0.2 micrometer to 1.2 micrometer). Do not administer as IV push or bolus injection.

e. HOW SUPPLIED

1. Ipilimumab will be supplied free of charge by Bristol-Myers-Squibb (BMS) and distributed by NCI/DCTD/CTEP.

2. Ipilimumab injection is supplied as 200 mg/40 mL (5 mg/mL). It is formulated as a clear to slightly opalescent, colorless to pale yellow, sterile, nonpyrogenic, single-use, isotonic aqueous solution that may contain particles.

   Each vial is a Type I flint glass vial with gray butyl stoppers and sealed with aluminum seals.

	**Process C**
**Component**	**200 mg/ vial[a]**
Ipilimumab	213 mg
Sodium Chloride, USP	249 mg
TRIS-hydrochloride	134.3 mg
Diethylenetriamine pentacetic acid	1.67 mg
Mannitol, USP	426 mg
Polysorbate 80 (plant-derived)	4.69 mg
Sodium Hydroxide	QS to pH 7
Hydrochloric acid	QS to pH 7
Water for Injection	QS: 42.6 mL
Nitrogen[b]	Processing agent

CLOSED EFFECTIVE 04/23/2018

SWOG Authorized Document

[a] Includes 2.6 mL overfill.
[b] Nitrogen is used to transfer the bulk solution through the pre-filled and sterilizing filters into the aseptic area.

f. STORAGE, PREPARATION& STABILITY

1. Store intact vials of ipilimumab refrigerated at (2^0 to 8^0C), protected from light. Do not freeze.

2. Ipilimumab is given undiluted or further diluted in 0.9% NaCl Injection, USP or 5% Dextrose Injection, USP in concentrations between 1 mg/mL and 4 mg/mL. Ipilimumab is stable in a polyvinyl chloride(PVC), non-PVC/non DEHP (di-(2-ethylhexyl) phthalate) IV bag or glass container up to 24 hours refrigerated at (2^0 to 8^0C) or at room temperature/room light.

3. The product may be infused using a volumetric pump at the protocol-specific dose(s) and rate(s) through a PVC IV solution infusion set with an in-line, sterile, nonpyrogenic, low-protein-binding filter (pore size of 0.2 micrometer to 1.2 micrometer).

4. Do not administer ipilimumab as an IV push or bolus injection.

   Stability of prepared IV ipilimumab solution is stable up to 24 hours refrigerated at (2^0 to 8^0C) or at room temperature/ room light.

5. Partially used vials or empty vials of ipilimumab injection should be discarded at the site according to appropriate drug disposal procedures.

g. DRUG ORDERING & ACCOUNTABILITY

1. Drug ordering: Study specific supplies will be provided to sites once a patient has been randomized. Starter supplies will not be provided. NCI-supplied agents may be requested by the Principal Investigator (or their authorized designee) at each participating institution. Pharmaceutical Management Branch (PMB) policy requires that drug be shipped directly to the institution where the patient is to be treated. PMB does not permit the transfer of agents between institutions (unless prior approval from PMB is obtained). The CTEP assigned protocol number (**S1400I**) must be used for ordering all CTEP supplied investigational agents. The responsible investigator at each participating institution must be registered with CTEP, DCTD through an annual submission of FDA Form 1572 and a CV. If there are several participating investigators at one institution, CTEP-supplied investigational agents for the study should be ordered under the name of one lead investigator at that institution. Active CTEP-registered investigators and investigator-designated shipping designees and ordering designees can submit agent requests through the PMB Online Agent Order Processing (OAOP) application <https://eapps-ctep.nci.nih.gov/OAOP/pages/login.jspx>. Access to OAOP requires the establishment of a CTEP Identity and Access Management (IAM) account <https://eapps-ctep.nci.nih.gov/iam/> and the maintenance of an "active" account status and a "current" password.

2. Drug Handling and Accountability (NCI logs or other)

   a. Drug Accountability: The investigator, or a responsible party designated by the investigator, must maintain a careful record of

CLOSED EFFECTIVE 04/28/2018

SWOG Authorized Document

the receipt, disposition, and return of all drugs received from the PMB using the Drug Accountability Record Form available on the NCI home page (http://ctep.cancer.gov).

b. Electronic logs are allowed as long as a print version of the log process is the exact same appearance as the current NCI DARF. If the trial is a placebo control trial – indicate that separate DARFs are needed for each patient to also include the placebo drug supply.

3. Drug return and/or disposition instruction

a. Drug Returns: All unused drug supplies should be returned to the PMB. When it is necessary to return study drug (e.g., sealed vials remaining when expired vials are recalled by the PMB), investigators should return the study drug to the PMB using the NCI Return Agent Form available on the NCI home page (http://ctep.cancer.gov).

b. Drug Expiration: Shelf life stability studies of the intact vials of ipilimumab are on-going.

4. Contact Information and Useful Links

Questions about drug orders, transfers, returns or accountability should be addressed to the PMB by calling 240/276-6575 Monday through Friday between 8:30 am and 4:30 pm Eastern Time or by email: PMBAfterHours@mail.nih.gov.

- CTEP Forms, Templates, Documents: http://ctep.cancer.gov/forms/
- NCI CTEP Investigator Registration: RCRHelpDesk@nih.gov
- PMB policies and guidelines: http://ctep.cancer.gov/branches/pmb/agent_management.htm
- PMB Online Agent Order Processing (OAOP) application: https://eapps-ctep.nci.nih.gov/OAOP/pages/login.jspx
- CTEP Identity and Access Management (IAM) account: https://ctepcore.nci.nih.gov/iam/index.jsp
- CTEP Associate Registration and IAM account help: ctepreghelp@ctep.nci.nih.gov

4.0 STAGING CRITERIA

See Section 4.0 of **S1400** for staging criteria.

5.0 ELIGIBILITY CRITERIA

Patient must meet the eligibility criteria below to be eligible for **S1400I**. If the patient does not meet the sub-study specific eligibility criteria listed in Section 5.1 and Section 5.2 of **S1400I**, but meets the common sub-study criteria listed in Section 5.3 of **S1400I**, submit the **S1400** Request for Sub-Study Reassignment Form for sub-study reassignment. Each of the criteria in the following section must met in order for a patient to be considered eligible for registration. For each criterion requiring test results and dates, please record this information on the Onstudy Form and submit via Medidata Rave® (see Section 14.0). Any potential eligibility issues should be addressed to the SWOG Statistics and Data Management Center (SDMC) in Seattle at 206/652-2267 or

CLOSED EFFECTIVE 04/23/2018

S1400question@crab.org prior to registration. NCI policy does not allow for waiver of any eligibility criterion (http://ctep.cancer.gov/protocolDevelopment/policies_deviations.htm).

In calculating days of tests and measurements, the day a test or measurement is done is considered Day 0. Therefore, if a test is done on a Monday, the Monday 4 weeks later would be considered Day 28. This allows for efficient patient scheduling without exceeding the guidelines. **If Day 7, 14, 16, 28 or 42 falls on a weekend or holiday, the limit may be extended to the next working day.**

5.1 Sub-Study Specific Disease Related Criteria

a. Patients must have been assigned to **S1400I**.

b. Patients must not have had prior treatment with an anti-PD-1, anti-PD-L1, anti-PD-L2, anti-CTLA-4 antibody, or any other antibody or drug specifically targeting T-cell costimulation or immune checkpoint pathways.

c. Patients must not have an active, known, or suspected autoimmune disease. Patients are permitted to enroll if they have vitiligo, type I diabetes mellitus, hypothyroidism only requiring hormone replacement, psoriasis not requiring systemic treatment, or conditions not expected to recur in the absence of an external trigger.

5.2 Sub-Study Specific Clinical/Laboratory Criteria

a. Patients must not have any known allergy or reaction to any component of the nivolumab and ipilimumab formulations.

b. Patients must not have received systemic treatment with corticosteroids (> 10 mg daily prednisone or equivalent) or other immunosuppressive medications within 14 days prior to sub-study registration. Inhaled or topical steroids, and adrenal replacement doses ≤ 10 mg daily prednisone or equivalent are permitted in the absence of active autoimmune disease.

c. Patients must not have a known positive test for hepatitis B virus surface antigen (HBV sAg) or hepatitis C virus ribonucleic acid (HCV antibody) indicating acute or chronic infection. Patients with a positive hepatitis C antibody with a negative viral load are allowed. [*This criterion replaces common eligibility criteria in Section 5.3m.*]

d. Patients must not have known history of testing positive for human immunodeficiency virus (HIV) or known acquired immunodeficiency syndrome (AIDS). [*This criterion replaces common eligibility criteria in Section 5.3n.*]

e. Patients must not have interstitial lung disease that is symptomatic or disease that may interfere with the detection or management of suspected drug-related pulmonary toxicity.

f. Patients must also be offered participation in banking for future use of specimens as described in Section 15.0.

g. Patients must have a Lipase, Amylase, TSH with reflex Free T3/T4 performed within 7 days prior to sub-study registration. Additional timepoints are noted in Section 9.0, Study Calendar. [Note: For the Canadian sites, testing for lipase only is acceptable.]

h. Patients must not have any Grade III/IV cardiac disease as defined by the New York Heart Association Criteria (i.e., patients with cardiac disease resulting in

CLOSED EFFECTIVE 04/23/2018

SWOG Authorized Document

marked limitation of physical activity or resulting in inability to carry on any physical activity without discomfort), unstable angina pectoris, and myocardial infarction within 6 months, or serious uncontrolled cardiac arrhythmia (see Section 18.1b).

1. Patients with a history of congestive heart failure (CHF) or at risk because of underlying cardiovascular disease or exposure to cardiotoxic drug should have an EKG and echocardiogram performed to evaluate cardiac function as clinically indicated.

2. Patients with evidence of congestive heart failure (CHF), myocardial infarction (MI), cardiomyopathy, or myositis should have a cardiac evaluation including lab tests and cardiology consultations as clinically indicated including EKG, CPK, troponin, and echocardiogram.

i. Patients who can complete PRO forms in English are required to complete a pre-study **S1400I** Patient Reported Outcomes (PRO) Questionnaire and a pre-study **S1400I** EQ-5D Questionnaire within 14 days prior to registration (see Section 18.2 of **S1400I**). NOTE: Patients enrolled to **S1400I** prior to 9/1/2016 are not eligible for the PRO study.

5.3 Common Eligibility Criteria for all Sub-Studies

The **S1400** Common Eligibility Criteria have been incorporated into Section 5.0 of each sub-study for ease of reference.

a. Patients whose biomarker profiling results indicate the presence of an EGFR mutation or EML4/ALK fusion are not eligible. Due to existence of approved therapies the biomarker exclusion rules are as follows:

Gene	Alteration type	Ineligible Alteration
EGFR	Substitution	L858R, T790M, A289V, G719A, S768I, G719C, R108K, G598V, R222C, L62R, L861Q, P596L, V774M
	Indel	non-frame shifting insertions or deletions between amino acids 740 and 780, in exons 19 and 20, transcript NM_005228
	Fusion	None
	Amplification	None
ALK	Substitution	None
	Indel	None
	Fusion	EML4-ALK, CLIP4-ALK, CLTC-ALK, KIF5B-ALK, NPM1-ALK, RANB2-ALK, STRN-ALK, TFG-ALK
	Amplification	None

b. Patients must have progressed (in the opinion of the treating investigator) following the most recent line of therapy.

c. Patients must not have received any prior systemic therapy (systemic chemotherapy, immunotherapy or investigational drug) within 21 days prior to sub-

CLOSED EFFECTIVE 04/23/2018

SWOG Authorized Document

study registration. Patients must have recovered (≤ Grade 1) from any side effects of prior therapy. Patients must not have received any radiation therapy within 14 days prior to sub-study registration. (See 5.3e for criteria regarding therapy for CNS metastases).

d. Patients must have measurable disease (see Section 10.0) documented by CT or MRI. The CT from a combined PET/CT may be used to document only non-measurable disease unless it is of diagnostic quality as defined in **S1400** Section 10.1c. Measurable disease must be assessed within 28 days prior to sub-study registration. Pleural effusions, ascites and laboratory parameters are not acceptable as the only evidence of disease. Non-measurable disease must be assessed within 42 days prior to sub-study registration. All disease must be assessed and documented on the Baseline Tumor Assessment Form. Patients whose only measurable disease is within a previous radiation therapy port must demonstrate clearly progressive disease (in the opinion of the treating investigator) prior to registration. See **S1400** Sections 15.0 and 18.1c for guidelines and submission instructions for required central radiology review.

e. Patients must have a CT or MRI scan of the brain to evaluate for CNS disease within 42 days prior to sub-study registration. Patient must not have leptomeningeal disease, spinal cord compression or brain metastases unless: (1) metastases have been locally treated and have remained clinically controlled and asymptomatic for at least 14 days following treatment prior to registration, AND (2) patient has no residual neurological dysfunction and has been off corticosteroids for at least 24 hours prior to sub-study registration.

f. Patients must have fully recovered from the effects of major surgery at least 14 days prior to sub-study registration.

g. Patients must not be planning to receive any concurrent chemotherapy, immunotherapy, biologic or hormonal therapy for cancer treatment. Concurrent use of hormones for non-cancer-related conditions (e.g., insulin for diabetes and hormone replacement therapy) is acceptable.

h. Patients must have an ANC ≥ 1,500/mcl, platelet count ≥ 100,000 mcl, and hemoglobin ≥ 9 g/dL obtained within 28 days prior to sub-study registration.

i. Patients must have adequate hepatic function as defined by serum bilirubin ≤ Institutional Upper Limit of Normal (IULN) and either ALT or AST ≤ 2 x IULN within 28 days prior to sub-study registration (if both ALT and AST are done, both must be ≤ 2 IULN). For patients with liver metastases, bilirubin and either ALT or AST must be ≤ 5 x IULN (if both ALT and AST are done, both must be ≤ 5 x IULN).

j. Patients must have a serum creatinine ≤ the IULN OR measured or calculated creatinine clearance ≥ 50 mL/min using the following Cockroft-Gault Formula:

$$\text{Calculated Creatinine Clearance} = \frac{(140 - \text{age}) \times (\text{actual body weight in kg})^{\dagger}}{72 \times \text{serum creatinine}^{*}}$$

Multiply this number by 0.85 if the patient is a female. These tests must have been performed within 28 days prior to sub-study registration.

†The kilogram weight is the patient weight with an upper limit of 140% of the IBW.
*Actual lab serum creatinine value with a minimum of 0.8 mg/dL.

k. Patients must have Zubrod performance status of 0-1 (see Section 10.4 in **S1400**) documented within 28 days prior to sub-study registration.

CLOSED EFFECTIVE 04/23/2018

SWOG Authorized Document

l. [*This common eligibility criteria has been removed as it conflicts with the sub-study specific criteria in Section 5.2h. A place holder remains to keep consistency across all sub-studies*].

m. [This common eligibility criteria has been removed as it conflicts with the *sub-study specific criteria in Section 5.2c. A place holder remains to keep consistency across all sub-studies*]

n. [*This common eligibility criteria has been removed as it conflicts with the sub-study specific criteria in Section 5.2d. A place holder remains to keep consistency across all sub-studies*]

o. Prestudy history and physical exam must be obtained within 28 days prior to sub-study registration.

p. No other prior malignancy is allowed except for the following: adequately treated basal cell or squamous cell skin cancer, *in situ* cervical cancer, adequately treated Stage I or II cancer from which the patient is currently in complete remission, or any other cancer from which the patient has been disease free for five years.

q. Patients must not be pregnant or nursing. Women/men of reproductive potential must have agreed to use an effective contraceptive method. A woman is considered to be of "reproductive potential" if she has had menses at any time in the preceding 12 consecutive months. In addition to routine contraceptive methods, "effective contraception" also includes heterosexual celibacy and surgery intended to prevent pregnancy (or with a side-effect of pregnancy prevention) defined as a hysterectomy, bilateral oophorectomy or bilateral tubal ligation. However, if at any point a previously celibate patient chooses to become heterosexually active during the time period for use of contraceptive measures outlined in the protocol, he/she is responsible for beginning contraceptive measures.

r. As a part of the OPEN registration process (see Section 13.4 in **S1400** for OPEN access instructions) the treating institution's identity is provided in order to ensure that the current (within 365 days) date of institutional review board approval for this study has been entered in the system.

s. Patients with impaired decision-making capacity are eligible as long as their neurological or psychological condition does not preclude their safe participation in the study (e.g., tracking pill consumption and reporting adverse events to the investigator).

t. Patients must be informed of the investigational nature of this study and must sign and give written informed consent in accordance with institutional and federal guidelines.

6.0 STRATIFICATION FACTORS

6.1 Patients will be randomized between nivolumab with or without ipilimumab using block randomization.

6.2 Randomization will be stratified by:

a. Gender (Male vs. Female)

b. Number of prior therapies (1 vs. 2 or more)

CLOSED EFFECTIVE 04/23/2018

SWOG Authorized Document

7.0 TREATMENT PLAN

For treatment or dose modification questions, please contact Drs. Scott Gettinger and Lyudmila A. Bazhenova at **S1400I**MedicalQuery@swog.org. For dosing principles or questions, please consult the SWOG Policy #38 "Dosing Principles for Patients on Clinical Trials" at https://www.swog.org/sites/default/files/docs/2017-11/Policy38.pdf.

7.1 Pre-Medication and Supportive Care

Premedication associated with standard drug administration and supportive care (including anti-diarrheals, antibiotics, diuretics or other medications) may be given as indicated by the current American Society of Clinical Oncology (ASCO) guidelines. Premedication for the use of prophylaxis for infusion reactions (e.g. diphenhydramine, acetaminophen, or other medications) may be given per institutional standard.

For patients that experience infusion reactions the following prophylactic pre-medications are recommended for future infusions: diphenhydramine 50 mg (or equivalent) and/or acetaminophen/paracetamol 325 to 1000 mg at least 30 minutes before additional nivolumab or ipilimumab administrations.

As there is potential for hepatic toxicity with nivolumab or nivolumab/ipilimumab combination, drugs with a predisposition to hepatoxicity should be used with caution in patients treated with nivolumab containing regimen.

The following medications are prohibited during the study (unless utilized to treat a drug related adverse event):

- Immunosuppressive agents
- Immunosuppressive doses of systemic corticosteroids
- Any concurrent anti-neoplastic therapy (i.e., chemotherapy, hormonal therapy, immunotherapy, extensive, non-palliative radiation therapy, or standard or investigational agents for treatment of NSCLC).

7.2 Treatment – Non-Match **S1400I**

Patients will be randomized to one of the following treatment arms:

Arm 1: Nivolumab plus Ipilimumab

Arm 2: Nivolumab

a. **Arm 1: Nivolumab plus Ipilimumab**

Agent	Dose	Route	Day	Schedule*
Nivolumab (BMS-936558)	3 mg/kg	IV 30 minutes	1	Q 14 days
Ipilimumab (BMS-734016)	1 mg/kg	IV 60 minutes	1	Q 42 days (q 3 cycles) **

* NOTE: A cycle of treatment is 14 days.

** NOTE: Ipilimumab will be administered on Day 1 of every third cycle (i.e. Cycle 1, Cycle 4, etc.) see Section 9.0. Note that nivolumab must be administered first. Ipilimumab to start 30 minutes after the end of nivolumab infusion.

CLOSED EFFECTIVE 04/23/2018

SWOG Authorized Document

Patients will be weighed prior to initiation of a new cycle of treatment. Dose recalculation based on weight change must be done if the patient experiences 10% or more weight gain or weight loss from the last-dosing weight. Following preparation of the dose, the entire contents of the IV bag should be administered (see Sections 3.1d and 3.2d).

Disease assessment are to take place every 6 weeks for the first year, then every 3 months. Treatment will continue until any of the criteria in Section 7.3 is met.

b. **Arm 2: Nivolumab**

Agent	Dose	Route	Day	Schedule*
Nivolumab (BMS-936558)	3 mg/kg	IV 30 minutes	1	Q 14 days

* NOTE: A cycle of treatment is 14 days.

Patients will be weighed prior to initiation of a new cycle of treatment. Dose recalculation based on weight change must be done if the patient experiences 10% or more weight gain or weight loss from the last-dosing weight. Following preparation of the dose, the entire contents of the IV bag should be administered (see Sections 3.1d).

Disease assessment are to take place every 6 weeks for the first year, then every 3 months. Treatment will continue until any of the criteria in Section 7.3 is met.

7.3 Criteria for Removal from Protocol Treatment

a. Progression of disease as defined in Section 10.2d in **S1400**. However, the patient may continue protocol treatment as long as the patient is continuing to clinically benefit from treatment in the opinion of the treating investigator. Patients should still be removed from protocol treatment for criteria below.

   * Upon progression, the **S1400** Request for New Sub-Study Assignment Form may be submitted to receive a new sub-study assignment (see Section 14.0).

b. Symptomatic deterioration (as defined in Section 10.2e of **S1400**).

c. Unacceptable toxicity.

d. Treatment delay for any reason > 84 days (12 weeks) (or as noted in Section 8.0).

e. The patient may withdraw from this study at any time for any reason.

7.4 Discontinuation of Treatment

All reasons for discontinuation of treatment must be documented in the Off Treatment Notice.

7.5 Follow-Up Period

All patients will be followed until death or 3 years after sub-study registration, whichever occurs first.

Note: Patients who enroll on a new sub-study following progression must continue follow-up on this sub-study, in addition to follow-up on the new sub-study.

CLOSED EFFECTIVE 04/23/2018

SWOG Authorized Document

8.0 TOXICITIES TO BE MONITORED AND DOSE MODIFICATIONS

8.1 NCI Common Terminology Criteria for Adverse Events

Two different versions of the NCI Common Terminology Criteria for Adverse Events (CTCAE) will be used on this study.

a. Serious Adverse Event (SAE) reporting

The CTCAE (NCI Common Terminology Criteria for Adverse Events) Version 5.0 will be utilized **for SAE reporting only**. The CTCAE Version 5.0 can be downloaded from the CTEP home page (https://ctep.cancer.gov) All appropriate treatment areas should have access to a copy of the CTCAE Version 5.0.

b. Routine toxicity reporting

This study will utilize the CTCAE Version 4.0 for routine toxicity reporting. A copy of the CTCAE Version 4.0 can be downloaded from the CTEP home page (https://ctep.cancer.gov). All appropriate treatment areas should have access to a copy of the CTCAE Version 4.0.

8.2 General Considerations

a. No dose reductions are allowed.

b. The maximum dose delay for any reason is 84 days (12 weeks).

c. Patients on IV steroids may be switched to an equivalent dose of oral corticosteroids (e.g., prednisone) at start of tapering or earlier, once sustained clinical improvement is observed. Lower bioavailability of oral corticosteroids should be taken into account when switching to the equivalent dose of oral corticosteroids.

d. Cardiotoxic drugs should be avoided during the course of study treatment.

8.3 Dose Modifications – Nivolumab and Ipilimumab, Arm 1

See Section 8.8 for dose modification and management for cardiomyopathy myocarditis.

a. Dose Delay Criteria for Combination Therapy with Nivolumab and Ipilimumab
Nivolumab and ipilimumab administration should be delayed for the following:

1. Any Grade ≥ 2 non-skin, drug-related adverse event, except for fatigue and laboratory abnormalities
2. Any Grade ≥ 3 skin drug-related AE
3. Any Grade ≥ 3 drug-related laboratory abnormality with the following exceptions for lymphopenia, AST, ALT, or total bilirubin or asymptomatic amylase or lipase:
   - Grade 3 lymphopenia does not require a dose delay
   - If a patient has a baseline AST, ALT, or total bilirubin that is within normal limits, delay dosing for drug-related Grade ≥ 2 toxicity
   - If a patient has baseline AST, ALT, or total bilirubin within the Grade 1 toxicity range, delay dosing for drug-related Grade≥ 3 toxicity
   - Any Grade ≥ 3 drug-related amylase or lipase abnormality that is not associated with symptoms or clinical manifestations of pancreatitis does not require dose delay. The Study Chairs, Drs Scott Gettinger and Lyudmila A. Bazhenova at

CLOSED EFFECTIVE 04/23/2018

SWOG Authorized Document

S1400I MedicalQuery@swog.org should be consulted for such Grade ≥ 3 amylase or lipase abnormalities.

4. Any AE, laboratory abnormality or inter-current illness which, in the judgment of the investigator, warrants delaying the dose of study medication.

   Patients receiving ipilimumab in combination with nivolumab that have drug-related toxicities that meet the criteria for dose delay, should have both drugs (ipilimumab and nivolumab) delayed until retreatment criteria are met. (Exceptions apply to the retreatment criteria after dose delay of ipilimumab and nivolumab for Grade ≥ 3 amylase and lipase abnormalities that are not associated with symptoms or clinical manifestations of pancreatitis and that are attributed to ipilimumab alone. (Refer to Section 8.6 further details.)

5. Nivolumab may be delayed until the next planned ipilimumab dose if the next ipilimumab dose is scheduled within the next 12 days. This will permit periodic ipilimumab dosing to be synchronized with nivolumab dosing.

6. Ipilimumab should be dosed at the specified interval regardless of any delays in intervening nivolumab doses. However, in order to maintain periodic synchronized dosing of ipilimumab and nivolumab, the dosing days of nivolumab and ipilimumab may be adjusted within the permitted +/- 5 day window, as long as consecutive nivolumab doses are given at least 12 days apart. Ipilimumab may be delayed beyond the 5 day window if needed to synchronize with the next nivolumab dose.

7. If an ipilimumab dose is delayed beyond 6 weeks from the prior ipilimumab dose, then subsequent ipilimumab doses should rescheduled to maintain the 6 week interval between consecutive ipilimumab doses.

8. A dose delay of ipilimumab which results in no ipilimumab dosing for > 12 weeks requires ipilimumab discontinuation, with exceptions as noted in Section 8.6.

b. Criteria to Resume Nivolumab and Ipilimumab Dosing

Patients may resume treatment with nivolumab and ipilimumab when drug-related AE(s) resolve(s) to Grade 1 or baseline value, with the following exceptions:

1. Patients may resume treatment in the presence of Grade 2 fatigue.

2. Patients who have not experienced a Grade 3 drug-related skin AE may resume treatment in the presence of Grade 2 skin toxicity.

3. Patients with baseline Grade 1 AST/ALT or total bilirubin who require dose delays for reasons other than a 2-grade shift in AST/ALT or total bilirubin may resume treatment in the presence of Grade 2 AST/ALT or total bilirubin.

CLOSED EFFECTIVE 04/23/2018

SWOG Authorized Document

4. Patients with combined Grade 2 AST/ALT and total bilirubin values meeting discontinuation parameters (Sections 8.5 and 8.6) should have treatment permanently discontinued.

5. Drug-related pulmonary toxicity, diarrhea, or colitis must have resolved to baseline before treatment is resumed.

6. Patients who received systemic corticosteroids for management of any drug-related toxicity must be off corticosteroids or have tapered down to an equivalent dose of prednisone +10 mg/day.

7. Drug-related endocrinopathies adequately controlled with only physiologic hormone replacement may resume treatment after consultation with the Study Chairs, Drs. Scott Gettinger and Lyudmila A. Bazhenova (**S1400I**MedicalQuery@swog.org).

8. Dose delay of ipilimumab which results in no ipilimumab dosing for > 12 weeks requires ipilimumab discontinuation, with exceptions as noted in Section 6.

9. In general, patients who meet criteria to resume ipilimumab will also have met criteria to resume nivolumab, so it should be feasible to synchronize dosing of both drugs when resuming ipilimumab. In order to facilitate this, the dosing days of nivolumab and ipilimumab may be adjusted within the permitted +/- 5 day window, as long as consecutive nivolumab doses are given at least 12 days apart.

10. One exception to note is when ipilimumab and nivolumab doses are delayed per investigator discretion due to drug- related Grade >3 amylase or lipase abnormalities not associated with symptoms or clinical manifestations of pancreatitis. If the investigator assesses the Grade >3 amylase or lipase abnormality to be related to ipilimumab and not related to nivolumab, nivolumab may be resumed when the amylase or lipase abnormality resolves to Grade < 3 but ipilimumab may only be resumed when the amylase or lipase abnormality resolves to Grade 1 or baseline. Investigator attribution of this toxicity to the ipilimumab dosing must be clearly noted in the patient's medical chart. The Study Chairs, Drs Scott Gettinger and Lyudmila A. Bazhenova (**S1400I**MedicalQuery@swog.org) should be consulted prior to resuming.

8.4 Dose Modifications – Nivolumab, Arm 2

See Section 8.8 for dose modification and management for cardiomyopathy myocarditis.

a. Dose Delay Criteria for Nivolumab

Nivolumab administration should be delayed for the following:

1. Any Grade ≥ 2 non-skin, drug-related adverse event, except for fatigue and laboratory abnormalities
2. Any Grade ≥ 3 skin drug-related AE
3. Any Grade ≥ 3 drug-related laboratory abnormality with the following exceptions for lymphopenia, AST, ALT, or total bilirubin or asymptomatic amylase or lipase:
   - Grade 3 lymphopenia does not require a dose delay
   - If a patient has a baseline AST, ALT, or total bilirubin that is within normal limits, delay dosing for drug-related Grade ≥ 2 toxicity

CLOSED EFFECTIVE 04/23/2018

- If a patient has baseline AST, ALT, or total bilirubin within the Grade 1 toxicity range, delay dosing for drug-related Grade≥ 3 toxicity
- Any Grade ≥ 3 drug-related amylase or lipase abnormality that is not associated with symptoms or clinical manifestations of pancreatitis does not require dose delay. The Study Chairs, Drs Scott Gettinger and Lyudmila A. Bazhenova at **S1400I**MedicalQuery@swog.org should be consulted for such Grade ≥ 3 amylase or lipase abnormalities.
- Any AE, laboratory abnormality or inter-current illness which, in the judgment of the investigator, warrants delaying the dose of study medication.

Patients who require delay of nivolumab should be re-evaluated weekly or more frequently if clinically indicated and resume nivolumab dosing when re-treatment criteria are met (see below).

b. Criteria to Resume Nivolumab Dosing

Patient may resume treatment with nivolumab when the drug-related AE(s) resolve(s) to Grade ≤ 1 or baseline, with the following exceptions:

1. Patients may resume treatment in the presence of Grade 2 fatigue.

2. Patients who have not experienced a Grade 3 drug-related skin AE may resume treatment in the presence of Grade 2 skin toxicity.

3. Patients with baseline Grade 1 AST/ALT or total bilirubin who require dose delays for reasons other than a 2-Grade shift in AST/ALT or total bilirubin may resume treatment in the presence of Grade 2 AST/ALT OR total bilirubin.

4. Patients with combined Grade 2 AST/ALT and total bilirubin values meeting discontinuation parameters (Section 8.5.) should have treatment permanently discontinued.

5. Drug-related pulmonary toxicity, diarrhea, or colitis must have resolved to baseline before treatment is resumed. Patients with persistent Grade 1 pneumonitis after completion of a steroid taper over at least 1 month may be eligible for retreatment if discussed with and approved by the Study Chairs, Drs Scott Gettinger and Lyudmila A. Bazhenova at S1400IMedicalQuery@swog.org.

6. Drug-related endocrinopathies adequately controlled with only physiologic hormone replacement may resume treatment after consultation with the Study Chairs, Drs Scott Gettinger and Lyudmila A. Bazhenova at (S1400IMedicalQuery@swog.org).

7. Patients who delay study treatment per investigator discretion due to any Grade ≥ 3 amylase or lipase abnormality that is not associated with symptoms or clinical manifestations of pancreatitis, and that is assessed by the investigator to be related to ipilimumab and not to nivolumab, may resume nivolumab when the amylase or lipase abnormality has resolved to Grade < 3. The Study Chairs, Drs Scott Gettinger and Lyudmila A. Bazhenova at

CLOSED EFFECTIVE 04/23/2018

SWOG Authorized Document

S1400IMedicalQuery@swog.org should be consulted prior to resuming nivolumab in such patients.

8. Dose delay of nivolumab which results in treatment interruption of > 6 weeks requires treatment discontinuation, with exceptions as noted in Section 8.5.

8.5 Treatment Discontinuation Criteria Nivolumab and the Combination (Nivolumab with Ipilimumab)

See Section 8.8 for dose modification and management for cardiomyopathy myocarditis.

Treatment with nivolumab should be permanently discontinued for any of the following:

a. Any Grade 2 drug-related uveitis or eye pain or blurred vision that does not respond to topical therapy and does not improve to Grade 1 severity within the re-treatment period OR requires systemic treatment

b. Any Grade ≥ 2 drug-related pneumonitis or interstitial lung disease that does not resolve to dose delay and systemic steroids (also see Pulmonary Adverse Event Management Algorithm);

c. Any Grade 3 drug-related bronchospasm, hypersensitivity reaction, or infusion reaction, regardless of duration;

d. Any Grade 3 non-skin, drug-related adverse event lasting > 7 days, with the following exceptions for uveitis, pneumonitis, bronchospasm, diarrhea, colitis, neurologic toxicity, hypersensitivity reactions, infusion reactions, endocrinopathies, and laboratory abnormalities:

1. Grade 3 drug-related uveitis, pneumonitis, bronchospasm, diarrhea, colitis, neurologic toxicity, hypersensitivity reaction, or infusion reaction of any duration requires discontinuation.
2. Grade 3 drug-related endocrinopathies adequately controlled with only physiologic hormone replacement do not require discontinuation.
3. Grade 3 drug-related laboratory abnormalities do not require treatment discontinuation except:
4. Grade 3 drug-related thrombocytopenia > 7 days or associated with bleeding requires discontinuation.
5. Any drug-related liver function test (LFT) abnormality that meets the following criteria require discontinuation (also see Hepatic Adverse Event Management Algorithm):
   - AST or ALT > 5-10x ULN for > 2 weeks
   - AST or ALT > 10x ULN
   - Total bilirubin > 5 x ULN
   - Concurrent AST or ALT > 3 x ULN **and** total bilirubin > 2 x ULN

e. Any Grade 4 drug-related adverse event or laboratory abnormality, except for the following events, which do not require discontinuation:

1. Grade 4 neutropenia ≤7 days
2. Grade 4 lymphopenia or leukopenia
3. Isolated Grade 4 amylase or lipase abnormalities that are not associated with symptoms or clinical manifestations of pancreatitis and decrease to < Grade 4 within 1 week of onset. The Study Chairs, Drs Scott Gettinger

CLOSED EFFECTIVE 04/23/2018

SWOG Authorized Document

and Lyudmila A. Bazhenova (**S1400I**MedicalQuery@swog.org) should be consulted for Grade 4 amylase or lipase abnormalities

4. Isolated Grade 4 electrolyte imbalances/abnormalities that are not associated with clinical sequelae and are corrected with supplementation/appropriate management within 72 hours of their onset
5. Grade 4 drug-related endocrinopathy adverse events such as adrenal insufficiency, ACTH deficiency, hyper- or hypothyroidism, or glucose intolerance, which resolve or are adequately controlled with physiologic hormone replacement (corticosteroids, thyroid hormones) or glucose controlling agents, respectively, may not require discontinuation after discussion with and approval from the Study Chairs, Drs. Scott Gettinger and Lyudmila A. Bazhenova (**S1400I**MedicalQuery@swog.org).

f. Dosing delays lasting > 6 weeks from the previous dose that occur for non-drug-related reasons may be allowed if approved by the Study Chairs, Drs. Scott Gettinger and Lyudmila A. Bazhenova (**S1400I**MedicalQuery@swog.org). Prior to re-initiating treatment in a patient with a dosing delay lasting > 6 weeks, the Study Chairs, Drs. Scott Gettinger and Lyudmila A. Bazhenova must be consulted. Tumor assessments should continue as per protocol even if dosing is delayed. Periodic study visits to assess safety and laboratory studies should also continue every 6 weeks or more frequently if clinically indicated during such dosing delays.

g. Any adverse event, laboratory abnormality, or intercurrent illness which, in the judgment of the Investigator, presents a substantial clinical risk to the patient with continued nivolumab dosing.

The assessment for discontinuation of nivolumab should be made separately from the assessment made for discontinuation of ipilimumab. Although there is overlap among the discontinuation criteria, if discontinuation criteria are met for ipilimumab but not for nivolumab, treatment with nivolumab may continue if ipilimumab is discontinued.

If a patient receiving combination therapy with nivolumab and ipilimumab meets criteria for discontinuation and the investigator is unable to determine whether the event is related to both or one study drug, the patient should discontinue both nivolumab and ipilimumab.

8.6 Treatment Discontinuation Criteria Ipilimumab

See Section 8.8 for dose modification and management for cardiomyopathy myocarditis.

Ipilimumab should be permanently discontinued if any of the following criteria are met:

a. Any Grade >2 drug-related uveitis or eye pain or blurred vision that does not respond to topical therapy and does not improve to Grade 1 severity within 2 weeks OR requires systemic treatment;

b. Any Grade >3 bronchospasm or other hypersensitivity reaction;

c. Any other Grade 3 non-skin, drug-related adverse with the following exceptions for laboratory abnormalities, grade 3 nausea and vomiting, grade 3 neutropenia and thrombocytopenia, and symptomatic endocrinopathies which resolved (with or without hormone substitution);

d. Any drug-related liver function test (LFT) abnormality that meets the following criteria require discontinuation:

CLOSED EFFECTIVE 04/12/2018

1. AST or ALT > 8x ULN
2. Total bilirubin > 5 x ULN
3. Concurrent AST or ALT > 3 x ULN and total bilirubin > 2 x ULN

e. Any Grade 4 drug-related adverse event or laboratory abnormality, except for the following events, which do not require discontinuation:

1. Grade 4 neutropenia < 7 days
2. Grade 4 lymphopenia or leukopenia
3. Isolated Grade 4 amylase or lipase abnormalities which are not associated with symptoms or clinical manifestations of pancreatitis. The Study Chairs, Drs. Scott Gettinger and Lyudmila A. Bazhenova (S1400IMedicalQuery@swog.org) should be consulted for Grade 4 amylase or lipase abnormalities.
4. Isolated Grade 4 electrolyte imbalances/abnormalities that are not associated with clinical sequelae and are corrected with supplementation/appropriate management within 72 hours of their onset
5. Grade 4 drug-related endocrinopathy adverse events such as adrenal insufficiency, ACTH deficiency, hyper- or hypothyroidism, or glucose intolerance, which resolve or are adequately controlled with physiologic hormone replacement (corticosteroids, thyroid hormones) or glucose controlling agents, respectively, may not require discontinuation after discussion with and approval from the Study Chairs, Drs. Scott Gettinger and Lyudmila A. Bazhenova (**S1400I**MedicalQuery@swog.org).

f. Any treatment delay resulting in no ipilimumab dosing for > 12 weeks with the following exceptions: Dosing delays to manage drug-related adverse events, such as prolonged steroid tapers, are allowed. Prior to re-initiating treatment in a patient with a dosing delay lasting > 12 weeks, the Study Chairs, Drs. Scott Gettinger and Lyudmila A. Bazhenova (**S1400I**MedicalQuery@swog.org) must be consulted. **Tumor assessments should continue as per protocol even if dosing is delayed.**

g. Dosing delays resulting in no ipilimumab dosing for > 12 weeks that occur for non-drug- related reasons may be allowed if approved by the Study Chairs, Drs. Scott Gettinger and Lyudmila A. Bazhenova (**S1400I**MedicalQuery@swog.org). Prior to re- initiating treatment in a patient with a dosing delay lasting > 12 weeks, the Study Chairs, Drs. Scott Gettinger and Lyudmila A. Bazhenova must be consulted. **Tumor assessments should continue as per protocol even if dosing is delayed.**

h. Any adverse event, laboratory abnormality, or intercurrent illness which, in the judgment of the Investigator, presents a substantial clinical risk to the patient with continued nivolumab dosing.

The assessment for discontinuation of ipilimumab should be made separately from the assessment made for discontinuation of nivolumab. Although there is overlap among the discontinuation criteria, if discontinuation criteria are met for ipilimumab but not for nivolumab, treatment with nivolumab may continue if ipilimumab is discontinued.

If a patient receiving combination therapy with nivolumab and ipilimumab meets criteria for discontinuation and the investigator is unable to determine whether the event is related to both or one study drug, the patient should discontinue both nivolumab and ipilimumab.

8.7 Treatment of Nivolumab or Ipilimumab Infusion Reactions

CLOSED EFFECTIVE 04/23/2018

SWOG Authorized Document

Since nivolumab and ipilimumab contain only human immunoglobulin protein sequences, they are unlikely to be immunogenic and induce infusion or hypersensitivity reactions. However, if such a reaction were to occur, it might manifest with fever, chills, rigors, headache, rash, pruritus, arthralgias, hypo- or hypertension, bronchospasm, or other symptoms. All Grade 3 or 4 infusion reactions should be reported within 24 hours to the Study Chairs, Drs. Scott Gettinger and Lyudmila A. Bazhenova (S1400IMedicalQuery@swog.org) and reported as an SAE if criteria are met. Infusion reactions should be graded according to NCI CTCAE (Version 4.0) guidelines.

Treatment recommendations are provided below and may be modified based on local treatment standards and guidelines, as appropriate:

a. For Grade 1:

Remain at bedside and monitor subject until recovery from symptoms. The following prophylactic premedications are recommended for future infusions: diphenhydramine 50 mg (or equivalent) and/or acetaminophen/paracetamol 325 to 1000 mg at least 30 minutes before additional nivolumab or ipilimumab administrations.

b. For Grade 2:

Stop the nivolumab or ipilimumab infusion, begin an IV infusion of normal saline, and treat the subject with diphenhydramine 50 mg IV (or equivalent) and/or acetaminophen/paracetamol 325 to 1000 mg; remain at bedside and monitor subject until resolution of symptoms. Corticosteroid and/or bronchodilator therapy may also be administered as appropriate. If the infusion is interrupted, then restart the infusion at 50% of the original infusion rate when symptoms resolve; if no further complications ensue after 30 minutes, the rate may be increased to 100% of the original infusion rate. Monitor subject closely. If symptoms recur, then no further nivolumab or ipilimumab will be administered at that visit. Administer diphenhydramine 50 mg IV, and remain at bedside and monitor the subject until resolution of symptoms. The amount of study drug infused must be recorded on the electronic case report form (eCRF).

For future infusions, the following prophylactic premedications are recommended: diphenhydramine 50 mg (or equivalent) and/or acetaminophen/paracetamol 325 to 1000 mg should be administered at least 30 minutes before nivolumab or ipilimumab infusions. If necessary, corticosteroids (up to 25 mg of SoluCortef or equivalent) may be used.

c. For Grade 3 or 4:

Immediately discontinue infusion of nivolumab or ipilimumab. Begin an IV infusion of normal saline and treat the subject as follows: Recommend bronchodilators, epinephrine 0.2 to 1 mg of a 1:1000 solution for subcutaneous administration or 0.1 to 0.25 mg of a 1:10,000 solution injected slowly for IV administration, and/or diphenhydramine 50 mg IV with methylprednisolone 100 mg IV (or equivalent), as needed. Subject should be monitored until the investigator is comfortable that the symptoms will not recur. Nivolumab or ipilimumab will be permanently discontinued. Investigators should follow their institutional guidelines for the treatment of anaphylaxis. Remain at bedside and monitor subject until recovery of the symptoms.

In case of late-occurring hypersensitivity symptoms (e.g., appearance of a localized or generalized pruritus within 1 week after treatment), symptomatic treatment may be given (e.g., oral antihistamine or corticosteroids)

CLOSED EFFECTIVE 04/13/2018

SWOG Authorized Document

8.8 Dose Modification and Management for Cardiomyopathy Myocarditis

- Drug will be held for Grade 2 cardiac dysfunction pending evaluation
- Drug will be permanently discontinued for Grade 3 or 4 cardiac dysfunction and Grade 2 events that do not recover to baseline or that reoccur
- Treatment with steroids as clinically indicated

**Cardiac ***	**Management/Next Dose for BMS-936558 (Nivolumab) + Ipilimumab Cardiac Toxicities**
≤ Grade 1	Hold dose pending evaluation and observation.** Evaluate for signs and symptoms of CHF, ischemia, arrhythmia or myositis. Obtain history EKG, CK (for concomitant myositis), CK-MB. Repeat troponin, CK and EKG 2-3 days. If troponin and labs normalize may resume therapy. If labs worsen or symptoms develop then treat as below. Hold pending evaluation.
Grade ≥2 with suspected myocarditis	Hold dose.** Admit to hospital. Cardiology consult. Rule out MI and other causes of cardiac disease. Cardiac Monitoring. Cardiac Echo. Consider cardiac MRI and cardiac biopsy. Initiate high dose methylprednisolone. If no improvement within 24 hours, add either infliximab, ATG or tacrolimus. Resume therapy if there is a return to baseline and myocarditis is excluded or considered unlikely.
Grade ≥2 with confirmed myocarditis	Off protocol therapy. Admit to CCU (consider transfer to nearest Cardiac Transplant Unit). Treat as above. Consider high dose methylprednisolone.<br>Add ATG or tacrolimus if no improvement. Off treatment.
**Including CHF, LV systolic dysfunction, Myocarditis, CPK, and troponin*<br>***Patients with evidence of myositis without myocarditis may be treated according as "other event"*<br>Note: The optimal treatment regimen for immune mediated myocarditis has not been established. Since this toxicity has caused patient deaths, an aggressive approach is recommended.	

8.9 Management Algorithms for Immuno-Oncology Agents

Immuno-oncology agents such as nivolumab and ipilimumab are associated with AEs that can differ in severity and duration than AEs caused by other therapeutic classes. Early recognition and management of AEs associated with immuno-oncology agents may mitigate severe toxicity. Management Algorithms have been developed to assist investigators in assessing and managing the following groups of AEs: (I-O).

CLOSED EFFECTIVE 04/20/2018

SWOG Authorized Document

a. Pulmonary Adverse Event Management

Toxicity	Toxicity Management and Follow-up
**Pulmonary (i.e., Pneumonitis)**	
Any Grade	Patients should be thoroughly evaluated to rule out non-inflammatory causes. If non-inflammatory cause treat accordingly. Evaluate with imaging and pulmonary consultation.
Grade 1	- Monitor for symptoms every 2-3 days<br>- Consider Pulmonary and Infectious Disease (ID) consults<br>- Re-image at least every 3 weeks<br><u>If worsens</u>:<br>- Treat as Grade 2 or 3-4
Grade 2	- Consider Pulmonary and ID consults<br>- Monitor symptoms daily, consider hospitalization<br>- 1.0 mg/kg/day methylprednisolone IV or oral equivalent<br>- Consider bronchoscopy, lung biopsy<br>- Re-image every 1-3 days<br><u>If improves</u>:<br>- When symptoms return to near baseline, taper steroids over at least 1 month, consider prophylactic antibiotics<br><u>If not improving after 2 weeks or worsening</u>:<br>- Treat as Grade 3-4
≥ Grade 3	- Hospitalize<br>- Pulmonary and ID consults<br>- 2-4 mg/kg/day methylprednisolone IV or oral equivalent<br>- Add prophylactic antibiotics for opportunistic infections<br>- Consider bronchoscopy, lung biopsy<br><u>If improves to baseline</u>:<br>- Taper steroids over at least 6 weeks<br><u>If not improving after 48 hours or worsening</u>:<br>- Add additional immunosuppression (e.g., infliximab, cyclophosphamide, intravenous immunoglobulin (IVIG), or mycophenolate mofetil)

CLOSED EFFECTIVE 04/23/2018

SWOG Authorized Document

b. Gastrointestinal Adverse

Toxicity	Toxicity Management and Follow-up
**Gastrointestinal (i.e., Diarrhea/Colitis)**	
Any Grade	Patients should be thoroughly evaluated to rule out non-inflammatory causes. If non-inflammatory cause treat accordingly. Opiates/narcotics may mask symptoms of perforation. Infliximab should not be used in cases of perforation or sepsis.
Grade 1	- Close monitoring for worsening symptoms.<br>- Educate patient to report worsening immediately<br>- Consider symptomatic treatment including hydration, electrolyte replacement, dietary changes (e.g., American Dietetic Association colitis diet), and loperamide.<br>If worsens:<br>- Treat as Grade 2 or 3/4
Grade 2	− Consider symptomatic treatment including hydration, electrolyte replacement, dietary changes (e.g., American Dietetic Association colitis diet), and loperamide.<br>If persists > 5-7 days or recurs:<br>- 0.5-1.0 mg/kg/day methylprednisolone IV or oral equivalent<br>- When symptoms improve to grade 1, taper steroids over at least 1 month, consider prophylactic antibiotics for opportunistic infections<br>If worsens or persists > 3-5 days with oral steroids:<br>- Treat as Grade 3/4
≥ Grade 3	- 1.0 to 2.0 mg/kg/day methylprednisolone IV or oral equivalent<br>- Add prophylactic antibiotics for opportunistic infections<br>- Consider lower endoscopy<br>If improves:<br>- Continue steroids until grade 1, then taper over at least 1 month<br>If persists > 3-5 days, or recurs after improvement:<br>- Add infliximab 5 mg/kg (if no contraindication).<br>Note: Infliximab should not be used in cases of perforation or sepsis

CLOSED EFFECTIVE 04/23/2018

c. Hepatic Adverse Event Management

Toxicity	Toxicity Management
**Hepatic (Elevated LFTs – ALT, AST, Total Bilirubin)**	
Any Grade	Patients should be thoroughly evaluated to rule out non-inflammatory causes. If non-inflammatory cause treat accordingly. Consider imaging for obstruction.
Grade 1	- Continue liver function tests (LFT) monitoring per protocol<br>If worsens:<br>- Treat as Grade 2 or 3/4
Grade 2	- Increase frequency of LFT monitoring to every 3 days until resolution to baseline. Resume routine monitoring<br>If elevations persist > 5-7 days or worsen:<br>- 0.5-1 mg/kg/day methylprednisolone IV or oral equivalent and when LFT returns to grade 1 or baseline, taper steroids over at least 1 month, consider prophylactic antibiotics for opportunistic infections
≥ Grade 3	- Increase frequency of LFT monitoring to every 1-2 days<br>- 1.0 to 2.0 mg/kg/day methylprednisolone IV or oral equivalent. The recommended starting dose for Grade 4 hepatitis is 2 mg/kg/day methylprednisolone IV.<br>- Add prophylactic antibiotics for opportunistic infections<br>- Consult gastroenterologist<br>If returns to Grade 2:<br>- Taper steroids over at least 1 month<br>If does not improve in >3-5 days, worsens or rebounds:<br>- Add mycophenolate mofetil 1 gram (g) twice daily (BID)<br>- If no response within an additional 3-5 days, consider other immunosuppressants per local guidelines

CLOSED EFFECTIVE 04/23/2018

SWOG Authorized Document

d. Renal Adverse Event Management

Toxicity	Toxicity Management
**Renal (i.e., Creatinine Increased)**	
Any Grade	Patients should be thoroughly evaluated to rule out non-inflammatory causes. If non-inflammatory cause treat accordingly.
Grade 1	- Monitor creatinine weekly until resolution to baseline; resume routine creatinine monitoring per protocol<br>If worsens:<br>- Treat as Grade 2 or 3/4
Grade 2-3	- Monitor creatinine every 2-3 days<br>- 0.5 to 1.0 mg/kg/day methylprednisolone IV or oral equivalent<br>- Consider renal biopsy<br>If returns to Grade 1:<br>- Taper steroids over at least 1 month, consider prophylactic antibiotics for opportunistic infections and routine creatinine monitoring per protocol<br>If elevations persist > 7 days or worsen:<br>- Treat as Grade 4
≥ Grade 4	- Monitor creatinine daily<br>- 1.0-2.0 mg/kg/day methylprednisolone IV or oral equivalent<br>- Consult nephrologist<br>- Consider renal biopsy<br>If returns to Grade 1:<br>- Taper steroids over at least 1 month and add prophylactic antibiotics for opportunistic infections

CLOSED EFFECTIVE 04/23/2018

SWOG Authorized Document

e. Skin Adverse Event Management

Toxicity	Toxicity Management
**Skin (i.e., Rash, Macula-papular)**	
Any Grade	Patients should be thoroughly evaluated to rule out non-inflammatory causes. If non-inflammatory cause treat accordingly.
Grade 1	Consider symptomatic treatment including oral antiprurities (e.g., diphenhydramine or hydrolyzine) and topical therapy (e.g., urea cream).
Grade 2	Consider symptomatic treatment including oral antiprurities (e.g., diphenhydramine or hydrolyzine) and topical therapy (e.g., urea cream).<br>If persists > 1-2 weeks or recurs:<br>- Consider skin biopsy<br>- Consider 0.5-1.0 mg/kg/day methylprednisolone IV or oral equivalent. Once improving, taper steroids over at least 1 month, consider prophylactic antibiotics for opportunistic infections<br>If worsens:<br>- Treat as Grade 3/4
Grade 3	- Consider skin biopsy<br>- Dermatology Consult<br>- 1.0-2.0 mg/kg/day methylprednisolone IV or oral equivalent<br>If improves to Grade 1:<br>- Taper steroids over at least 1 month and add prophylactic antibiotics for opportunistic infections
Grade 4	- Consider skin biopsy<br>- Dermatology Consult<br>- 1.0-2.0 mg/kg/day methylprednisolone IV or oral equivalent<br>If improves to Grade 1:<br>- Taper steroids over at least 1 month and add prophylactic antibiotics for opportunistic infections

f. Endocrinopathy Adverse Event Management

Toxicity	Toxicity Management
**Endocrinopathy (Endocrine Disorders - adrenal insufficiency, ACTH deficiency, hyper- or hypothyroidism, or glucose intolerance)**	
Any Grade	- Patients should be thoroughly evaluated to rule out non-inflammatory causes. If non-inflammatory cause treat accordingly. Consider visual field testing, endocrinology consultation, and imaging.<br>- Monitor patients for signs and symptoms of endocrinopathies. Non-specific symptoms include headache, fatigue, behavior changes, changed mental status, vertigo, abdominal pain, unusual bowel habits, hypotension and weakness.<br>- Patients should be thoroughly evaluated to rule out any alternative etiology (e.g., disease progression including brain metastases, infections, etc.)<br>- If a patient experiences an AE that is thought to be possibly of autoimmune nature (e.g., thyroiditis, pancreatitis, hypophysitis, diabetes insipidus), the investigator should send a blood sample for appropriate autoimmune antibody testing

CLOSED EFFECTIVE 04/23/2018

SWOG Authorized Document

<table>
<tr><th>Toxicity</th><th>Toxicity Management</th></tr>
<tr><td colspan="2">Endocrinopathy (Endocrine Disorders - adrenal insufficiency, ACTH deficiency, hyper- or hypothyroidism, or glucose intolerance)</td></tr>
<tr><td>Grade 1</td><td>(including those with asymptomatic TSH elevation)
Monitor patient with appropriate endocrine function test
- If TSH < 0.5X LLN, or TSH >2X ULN or consistently out of range in 2 subsequent measurements, include FT4 at subsequent cycles as clinically indicated and consider Endocrinology Consult.</td></tr>
<tr><td>Grade 2</td><td>(including those with symptomatic endocrinopathy)
− Discuss with Study Chair
− Initiate hormone replacement as needed for management
− Evaluate endocrine function, and as clinically indicated, consider pituitary scan
− For patients with abnormal lab/pituitary scan work up, consider short-term,1-2 mg/kg/day methylprednisolone IV or oral equivalent with relevant hormone therapy
- For patients with normal endocrine work up (lab or MRI scans), repeat labs in 1-3 week/MRI in 1 month.
- If improves (with or without hormone replacement):
  - Taper steroids over at least 1 month and consider prophylactic antibiotics for opportunistic infections
  - Patients with adrenal insufficiency may need to continue steroids with mineral corticoid component
- Suspicion of adrenal crisis (e.g., severe dehydration, hypotension, shock out of proportion to current illness)
  - Discuss with Study Chair
  - Rule out sepsis
  - Administer stress dose of IV steroids with mineral corticoid activity
  - Administer IV fluids
  - Consult endocrinologist
  - If adrenal crisis ruled out, then treat as above for symptomatic endocrinopathy</td></tr>
<tr><td>Grade 3</td><td rowspan="2">−Discuss with Study Chair
−Initiate empiric IV corticosteroids (e.g., methylprednisolone IV or oral equivalent) at 1 to 2 mg/kg/day
−Administer hormone replacement therapy as necessary
−For adrenal crisis, severe dehydration, hypotension, or shock: immediately initiate intravenous corticosteroids with mineralocorticoid activity
−Consult endocrinologist
- Once improving, gradually taper immunosuppressive steroids over ≥4 weeks</td></tr>
<tr><td>Grade 4</td></tr>
</table>

CLOSED EFFECTIVE 04/23/2018

SWOG Authorized Document

g. Neurological Adverse Event Management

Toxicity	Dose Modification	Toxicity Management
**Neurological Toxicity (Nervous System Disorders)**		
Any Grade	Patients should be thoroughly evaluated to rule out non-inflammatory causes. If non-inflammatory cause treat accordingly.	
Grade 1	- Continue to monitor the patient.<br>If worsens:<br>- Treat as Grade 2 or 3/4	
Grade 2	−Treat symptoms per local guidelines<br>−Consider 0.5 to 1.0 mg/kg/day methylprednisolone IV or oral equivalent<br>If worsens:<br>−Treat as Grade 3/4	
≥ Grade 3	−Obtain Neurology Consult<br>−Treat symptoms per local guidelines<br>−1.0-2.0 mg/kg/day methylprednisolone IV or oral equivalent<br>−Add prophylactic antibiotics for opportunistic infections<br>If improves to Grade 2:<br>−Taper steroids over at least 1 month<br>If worsens or atypical presentation:<br>−Consider IVIG or other immunosuppressive therapies per local guidelines	

8.10 Dose Modification Contacts

For treatment or dose modification questions, please contact Drs. Scott Gettinger and Lyudmila A. Bazhenova at **S1400I**MedicalQuery@swog.org. For dosing principles or questions, please consult the SWOG Policy #38 "Dosing Principles for Patients on Clinical Trials" at https://www.swog.org/sites/default/files/docs/2017-11/Policy38.pdf.

8.11 Adverse Event Reporting

Toxicities (including suspected reactions) that meet the expedited reporting criteria as outlined in Section 16.0 of the protocol must be reported to the Operations Office, Study Coordinator and NCI via CTEP-AERS, and to the IRB per local IRB requirements.

CLOSED EFFECTIVE 04/23/2018

Appended 11/18/15

CLOSED EFFECTIVE 04/23/2018

9.0 STUDY CALENDAR

		Cycle 1		Cycle 2		Cycle 3		Cycle 4		Cycle 5		Cycle 6					
**REQUIRED STUDIES**	PRE-STUDY	Wk 1	Wk 2	Wk 3	Wk 4	Wk 5	Wk 6	Wk 7	Wk 8	Wk 9	Wk 10	Wk 11	Wk 12	Subse-quent Cycles β	At Off Tx	Off Tx FU Prior to Prog Δ	Off Tx FU After Prog √
**PHYSICAL**																	
History & Physical Exam	X			X		X		X		X		X		X∞	X	X	
Weight & Performance Status	X			X		X		X		X		X		X	X	X	
Disease Assessment Ω	X							XΩ						X Ω		X Ω	
Toxicity Notation				X		X		X		X		X		X	X	Xȸ	Xȸ
Smoking Status Assessment	X														X		
**S1400I** PRO Questionnaire ☺	X☺			X		X		X		X		X		X☺		X☺	X☺
**S1400I** EQ-5D ☺	X☺					X		X		X				X☺		X☺	X☺
**LABORATORY**																	
CBC/Diff/Platelets/Hgb	X	X€		X		X		X		X		X		X	X	Xȸ	Xȸ
Serum Bilirubin	X	X€		X		X		X		X		X		X	X	Xȸ	Xȸ
ALT and AST	X	X€		X		X		X		X		X		X	X	Xȸ	Xȸ
Serum Creatinine/Calc CrCl	X	X€		X		X		X		X		X		X	X	Xȸ	Xȸ
Alkaline phosphatase	X	X€		X		X		X		X		X		X			
Blood urea nitrogen	X	X€		X		X		X		X		X		X			
Ca, Mg, Na, K, Cl	X	X€		X		X		X		X		X		X			
Glucose	X	X€		X		X		X		X		X		X			
Amylaseπ	X							X						Xπ			
Lipaseπ	X							X						Xπ			
TSH/w.reflex Free T3/T4 π	X							X						Xπ			
Albumin ¥	X																
Creatine phosphokinase (CPK)	X Ж							X Ж						X Ж			
Troponin	X Ж							X Ж						X Ж			
**X-RAYS AND SCANS**																	
CT or MRI for Disease Assessment Ω	X							XΩ						XΩ		X Ω	
Brain CT/MRI	X													X♦		X♦	
Image Submission Σ	X							X						X		X	
EKG	X Ж							X Ж						X Ж			
Echocardiogram (ECHO)	X Ж							X Ж						X Ж			

Appended 11/18/15

		Cycle 1		Cycle 2		Cycle 3		Cycle 4		Cycle 5		Cycle 6		Subse-quent Cycles β	At Off Tx	Off Tx FU Prior to Prog Δ	Off Tx FU After Prog √
**REQUIRED STUDIES**	PRE-STUDY	Wk 1	Wk 2	Wk 3	Wk 4	Wk 5	Wk 6	Wk 7	Wk 8	Wk 9	Wk 10	Wk 11	Wk 12				
**SPECIMEN SUBMISSION**																	
Tissue for Banking																	X§
Blood for Banking ƒ	X			X				X		X							Xð
**TREATMENT**																	
**Arm 1: Nivolumab/Ipilimumab**																	
Nivolumab		X		X		X		X		X		X		X			
Ipilimumab		X						X						X ш			
**Arm 2: Nivolumab**																	
Nivolumab		X		X		X		X		X		X		X			

NOTE: Forms are found on the protocol abstract page of the SWOG website (www.swog.org). Forms submission guidelines are found in Section 14.0.
NOTE: Unless indicated otherwise in the protocol, scheduled procedures and assessments (treatment administration, toxicity assessment for continuous treatment, disease assessment, specimen collection and follow-up activities) must follow the established SWOG guidelines as outlined in https://www.swog.org/sites/default/files/docs/2017-10/Best%20Practices%20upddate.pdf.

CLOSED EFFECTIVE 04/23/2018

Footnotes for Calendar:

Ω CT or MRI (the same method used at prestudy to meet the eligibility criteria in Section 5.0 of **S1400I**) must be repeated every 6 weeks (± 7 day window) for the first year, then every 3 months until disease progression and discontinuation of protocol treatment. If the patient remains on protocol treatment after progression due to clinical benefit (per Section 7.3), scans must continue per protocol schedule until treatment is discontinued. If the patient is removed from protocol treatment prior to progression, scans must continue per protocol schedule until progression.

♦ Only if patient has brain metastases at baseline. Scans must use the same modality as baseline and be repeated every 12 weeks (+/- 7 days) until disease progression.

∑ Submit scans as outlined in Section 14.0 and Section 15.0 of **S1400I**.

☺ The **S1400I** Patient Reported Outcomes (PRO) Questionnaire administered at Pre-study (within 14 days prior to **S1400I** registration) and at **Weeks 3, 5, 7, 9, 11, 13, 25 and 37**. The **S1400I** EQ-5D Questionnaire is administered at Prestudy (within 14 days prior to **S1400I** registration), **Weeks** 5, 7, 9, 13, 25, 37, and **Years** 1, 2, and 3. (see Section 18.2 of **S1400I**). For each timepoint, the Cover Sheet for PRO Questionnaires should also be completed. The scheduled PRO assessments should be completed even if the patient goes off treatment early. Note: Patients enrolled to **S1400I** prior to **9/1/2016** are not eligible for the PRO study.

β During continued treatment, items marked under physical and laboratory should be performed at every subsequent cycle, unless otherwise noted. Disease assessments are to take place every 6 weeks for the first year, then every 3 months. (± 7 days window).
Treatment and evaluation will continue until any of the criteria in Section 7.3 is met.

Δ After off treatment prior to progression, patients should be followed by repeating indicated laboratory tests every 3 months or more often as clinically indicated for the first year, then every 6 months for up to 3 years from date of sub-study registration. Disease assessment should continue every 6 weeks until progression.

√ After off treatment after progression, follow-up will occur (with lab tests and scans performed at the discretion of the treating physician) every 6 months for 2 years then at end of year 3 from date of sub-study study registration.

ƒ With patient's consent, additional research blood draws will be collected (see Section 15.0 of **S1400I**).

π Amylase, Lipase, and TSH w/ reflex Free T3/T4 are required at pre-study and must be repeated every 6 weeks while on treatment. Reflex to T3/T4 is not required for abnormal TSH if due to known hypothyroidism.

¥ Result of these tests do not determine eligibility but are recommended prior to sub-study registration.

§ With patient's consent, an additional research biopsy within 1 month after the time of first progression among patients who had a response to protocol treatment (in the opinion of the treating physician) must be collected (see Section 15.0 of **S1400I**).

€ If the pre-study tests are obtained within 14 days prior to treatment, the tests need not be repeated.

ȸ Assessments should continue until resolution of all acute adverse events.

ɯ Ipilimumab will be administered on Day 1 of every third cycle (i.e. Cycle 1, Cycle 4, Cycle 7 etc.).

ð Blood for Banking specimen must be collected at first progression after study treatment (see Section 15.0 of **S1400I**).

Ж CPK, troponin, EKG, ECHO are to be performed prestudy if clinically indicated (see Section 5.2) and are to be repeated every 6 weeks as clinically indicated while on treatment.

∞ During subsequent cycles, History & Physical Exams must be performed on Day 1 of every other cycle.

CLOSED EFFECTIVE 04/23/2018

10.0 CRITERIA FOR EVALUATION AND ENDPOINT ANALYSIS

10.1 Measurability of Lesions

a. **Measurable disease**: Measurable disease is defined differently for lymph nodes compared with other disease and will be addressed in a separate section below.

1. Lesions that can be accurately measured in at least one dimension (longest diameter to be recorded) as ≥ 2.0 cm by chest x-ray, by ≥ 1.0 cm with CT or MRI scans, or ≥ 1.0 cm with calipers by clinical exam. All tumor measurements must be recorded in decimal fractions of centimeters.

   The defined measurability of lesions on CT scan is based on the assumption that CT slice thickness is 0.5 cm or less. If CT scans have slice thickness greater than 0.5 cm, the minimum size for a measurable lesion should be twice the slice thickness.

2. Malignant lymph nodes are to be considered pathologically enlarged and measurable if it measures ≥ 1.5 cm in **SHORT AXIS** (greatest diameter perpendicular to the long axis of the lymph node) when assessed by scan (CT scan slice recommended being no greater than 0.5 cm).

b. **Non-measurable disease**: All other lesions (or sites of disease), including small lesions (longest diameter < 1.0 cm or pathologic lymph nodes with ≥ 1.0 cm to < 1.5 cm short axis), are considered non-measurable disease. Note: Lymph nodes that have a short axis < 1.0 cm (10 mm) are considered non-pathological and should not be recorded or followed. Bone lesions, leptomeningeal disease, ascites, pleural/pericardial effusions, lymphangitis cutis/pulmonitis, inflammatory breast disease, and abdominal masses (not followed by CT or MRI), are considered non-measurable as are previously radiated lesions that have not progressed.

c. Notes on measurability

1. For CT and MRIs, the same type of scanner should be used and the image acquisition protocol should be followed as closely as possible to prior scans. Body scans should be performed with breath-hold scanning techniques, if possible.

2. PET-CT: At present, the low dose or attenuation correction CT portion of a PET-CT is not always of optimal diagnostic CT quality for use with RECIST measurements. However, if the site can document that the CT performed as part of a PET-CT is of identical diagnostic quality to a diagnostic CT, then the CT portion of the PET-CT can be used for RECIST measurements and can be used interchangeably with conventional CT.

   NOTE REGARDING DIAGNOSTIC QUALITY:

   CT – Computed Tomograph Imaging

   In order for a CT to be of diagnostic quality to be used in determining measurable disease, the slice thickness needs to match the protocol Section 10.0.

CLOSED EFFECTIVE 04/23/2018

SWOG Authorized Document

Recommended Scan mode:	Multi-detector and/or helical
Contrast Enhancement:	IV and oral contrast unless contraindicated
Slice Section thickness:	maximum 5mm, preferable 2.5mm or less
Slice Increment:	continuous or overlapping sections; no gaps
Imaging Region:	Thoracic inlet through adrenal glands (and appropriate scans if disease exists elsewhere)
Image Matrix size:	512 × 512 or better
Image Reconstruction / Filter:	Institutional standard

If a CT scan is performed with a slice thickness greater than 5 mm then lesions must be twice the slice thickness. If any PET/Spiral CT is used at baseline where the CT is of diagnostic quality, follow-up scans can be done by a spiral CT.

If any PET/Conventional CT is used at baseline where the CT is of diagnostic quality, follow-up scans can be done by conventional CT.

Institutions will have to submit radiology reports documenting that the CT used in PET/CT is of diagnostic quality. No other methods of assessments are interchangeable.

MRI – Magnetic Resonance Imaging
MRI can be performed using a 1.5 or 3.0 T field strength. If a MRI is performed instead of a CT, the MRI can be performed according to institutions clinical standard of care protocols with slice thickness of no more than 5mm (in transverse).
If an MRI scan is performed with a slice thickness greater than 5 mm, then lesions must be twice and above the slice thickness.

PET/CT – Positron Emission Tomography with FDG
When a FDG PET/CT is performed, the emission scans should be started in the range of 60 – 75 min after FDG injection, otherwise use your institution protocols. It is necessary for follow up scans that they are performed in an **identical way** to the baseline with the same PET/CT scanner and a variation in timing of no more than +/- 10 min. Preferably, schedule the patient for both baseline and follow-up scans at the same time of day (AM or PM) to improve reproducibility.

3. Ultrasound: Ultrasound is not useful in assessment of lesion size and should not be used as a method of measurement.

4. Cystic lesions that meet the criteria for radiographically defined simple cysts should not be considered as malignant lesions (neither measurable nor non-measurable) since they are, by definition simple cysts.

5. If a target lesion becomes very small, some radiologists indicate that it is too small to measure. If the lesion is actually still present, a default measurement of 0.5 cm should be applied. If the radiologist believes the lesion has gone, a default measurement of 0.0cm should be recorded.

CLOSED EFFECTIVE 04/23/2018

10.2 Objective Status at Each Disease Evaluation

Objective Status is to be recorded at each evaluation. All measurable lesions up to a maximum of 2 lesions per organ 5 lesions in total, representative of all involved organs, should be identified as *target* lesions at baseline. All other lesions (or sites of disease) including any measurable lesions over and above the 5 target lesions should be identified as *non-target* lesions. Measurements must be provided for target measurable lesions, while presence or absence must be noted for non-target measurable and non-measurable disease.

For studies that use disease progression as an endpoint, whole body scanning at specific intervals is necessary to determine that progression is NOT present outside of the “target” areas. Therefore, in these studies it is not acceptable to image only the “target” areas of the body in follow-up scans. For study-specific imaging requirements, see the Study Calendar in the appropriate sub-study specific section.

a. **Complete Response (CR):** Complete disappearance of all target and non-target lesions (with the exception of lymph nodes mentioned below). No new lesions. No disease related symptoms. Any lymph nodes (whether target or non-target) must have reduction in short axis to < 1.0 cm. All disease must be assessed using the same technique as baseline.

b. **Partial Response (PR):** Applies only to patients with at least one measurable lesion. Greater than or equal to 30% decrease under baseline of the sum of appropriate diameters of all target measurable lesions. No unequivocal progression of non-measurable disease. No new lesions. All target measurable lesions must be assessed using the same techniques as baseline.

c. **Stable:** Does not qualify for CR, PR, Progression or Symptomatic Deterioration. All target measurable lesions must be assessed using the same techniques as baseline.

d. **Progression**: One or more of the following must occur: 20% increase in the sum of appropriate diameters of target measurable lesions over smallest sum observed (over baseline if no decrease during therapy) using the same techniques as baseline, as well as an absolute increase of at least 0.5 cm. Unequivocal progression of non-measurable disease in the opinion of the treating physician (an explanation must be provided). Appearance of any new lesion/site. Death due to disease without prior documentation of progression and without symptomatic deterioration (see Section 10.2e).

If a new lesion is equivocal, for example because of its small size, continued therapy and follow-up evaluation will clarify if it represents truly new disease. If repeat scans confirm there is definitely a new lesion, then progression should be declared using the date of the initial scan

Notes regarding new lesions: FDG-PET imaging can complement regular scans in identifying new lesions according to the following algorithm.

1. Negative FDG-PET at baseline, with a positive FDG-PET at follow-up is a sign of progression based on a new lesion.

2. No FDG-PET at baseline and a positive FDG-PET at follow-up corresponding to a potential new site of disease must have a confirmation

CLOSED EFFECTIVE 04/23/2018

SWOG Authorized Document

by anatomical assessment (e.g. CT, MRI, x-ray) as new site of disease to be considered progressive disease. In such a case, the date of progressive disease will be the date of the initial abnormal FDG-PET.

e. **Symptomatic deterioration**: Global deterioration of health status requiring discontinuation of treatment without objective evidence of progression. Efforts should be made to obtain objective evidence of progression after discontinuation.

f. **Assessment inadequate, objective status unknown**. Progression or symptomatic deterioration has not been documented, and one or more target measurable lesions have not been assessed or inconsistent assessment methods were used.

g. Objective status notes:

1. Non-measurable and non-target measurable disease do not affect Objective Status in determination of CR (must be absent--a patient who otherwise has a CR, but who has non-measurable or non-target measurable disease present or not assessed, will be classified as having a PR). However, non-measurable and non-target lesions are included in determination of progression (if new sites of disease develop or if unequivocal progression occurs in the opinion of the treating physician).

2. An objective status of PR or stable cannot follow one of CR. Stable can follow PR only in the rare case that tumor increases too little to qualify as progression, but enough that a previously documented 30% decrease no longer holds.

3. In cases for which initial flare reaction is possible (hypercalcemia, increased bone pain, erythema of skin lesions), objective status is not progression unless either symptoms persist beyond 4 weeks or there is additional evidence of progression.

4. Lesions that appear to increase in size due to presence of necrotic tissue will not be considered to have progressed.

5. For bone disease documented on bone scan only, increased uptake does not constitute unequivocal progression. However, increase in the soft tissue component of a lesion as measured by CT or MRI would constitute progression.

6. Appearance of new pleural effusions does not constitute unequivocal progression unless cytologically proven of neoplastic origin, since some effusions are a toxicity related to therapy or other medical conditions. Increase in the size of an existing effusion does not constitute unequivocal progression, since the fluid status of the patient could alter the size of the effusion.

7. If CR determination depends on a lesion for which the status is unclear by the required tests, it is recommended the residual lesion be investigated with biopsy or fine needle aspirate.

10.3 Best Response

This is calculated from the sequence of objective statuses.

CLOSED EFFECTIVE 04/03/2018

SWOG Authorized Document

a. CR: Two or more objective statuses of CR a minimum of four weeks apart documented before progression or symptomatic deterioration.

b. PR: Two or more objective statuses of PR or better a minimum of four weeks apart documented before progression or symptomatic deterioration, but not qualifying as CR.

c. Unconfirmed CR: One objective status of CR documented before progression or symptomatic deterioration but not qualifying as CR or PR.

d. Unconfirmed PR: One objective status of PR documented before progression or symptomatic deterioration but not qualifying as CR, PR or unconfirmed CR.

e. Stable/no response: At least one objective status of stable/no response documented at least six weeks after registration and before progression or symptomatic deterioration, but not qualifying as anything else above.

f. Increasing disease: Objective status of progression within 12 weeks of registration, not qualifying as anything else above.

g. Symptomatic deterioration: Objective status of symptomatic deterioration within 12 weeks of registration, not qualifying as anything else above.

h. Inadequate assessment, response unknown: Progression or symptomatic deterioration greater than 12 weeks after registration and no other response category applies.

10.4 Performance Status

Patients will be graded according to the Zubrod Performance Status Scale.

POINT	DESCRIPTION
0	Fully active, able to carry on all pre-disease performance without restriction.
1	Restricted in physically strenuous activity but ambulatory and able to carry out work of a light or sedentary nature, e.g., light housework, office work.
2	Ambulatory and capable of self-care but unable to carry out any work activities; up and about more than 50% of waking hours.
3	Capable of limited self-care, confined to bed or chair more than 50% of waking hours.
4	Completely disabled; cannot carry on any self-care; totally confined to bed or chair.

10.5 Time to Death

From date of sub-study registration (or date of screening/pre-screening registration if patient never enrolls in a sub-study) to date of death due to any cause. Patients last known to be alive are censored at date of last contact.

CLOSED EFFECTIVE 04/23/2018

SWOG Authorized Document

10.6 Investigator-Assessed Progression-Free Survival (IA-PFS)

From date of sub-study registration to date of first documentation of progression assessed by local review or symptomatic deterioration (as defined above), or death due to any cause. Patients last known to be alive without report of progression are censored at date of last disease assessment. For patients with a missing scan (or consecutive missing scans) whose subsequent scan determines progression, the expected date of the first missing scan (as defined by the disease assessment schedule) will be used as the date of progression.

10.7 Progression-Free Survival by Central Review

From date of sub-study registration to date of first documentation of progression assessed by central review or symptomatic deterioration (as defined above), or death due to any cause. Patients last known to be alive without report of progression are censored at date of last disease assessment. For patients with a missing scan (or consecutive missing scans) whose subsequent scan determines progression, the expected date of the first missing scan (as defined by the disease assessment schedule) will be used as the date of progression.

10.8 Duration of Response (DoR)

From date of first documentation of response (CR or PR) to date of first documentation of progression assessed by local review or symptomatic deterioration (as defined above), or death due to any cause among patients who achieve a response (CR or PR). Patients last known to be alive without report of progression are censored at date of last disease assessment. For patients with a missing scan (or consecutive missing scans) whose subsequent scan determines progression, the expected date of the first missing scan (as defined by the disease assessment schedule) will be used as the date of progression.

See **S1400I** Section 18.2 for criteria for evaluation and endpoint analysis of **S1400I** Patient Reported Outcomes (PRO).

11.0 STATISTICAL CONSIDERATIONS

This study will employ a modified version of Design #1: a Phase II/III design as described in **S1400** Section 11.1a. A complete description of the statistical design and analysis plan is included in Section 11.0 of **S1400**. This section includes details specific to **S1400I**.

11.1 Primary Objective

The primary objective is to compare overall survival (OS) with nivolumab in combination with ipilimumab versus nivolumab monotherapy in immune checkpoint-inhibitor naïve patients who are not eligible for the other biomarker-driven sub-studies in **S1400.**

11.2 Sample Size with Power Justification

The expected proportion of patients assigned to **S1400I** is 50%. The estimated monthly accrual rate to **S1400I** is an average rate of 6-8 patients per month in the first year and 12-18 patients per month thereafter.

This study is using a modified version of the Phase II/III Design #1 (see **S1400** Section 11.1). Due the expected mechanism of action of checkpoint inhibitors and their effects on PFS, this study will not include the phase II interim analysis. The design of the study is a straight Phase III design.

CLOSED EFFECTIVE 04/23/2018

SWOG Authorized Document

The sample size for this study is based on a design that targets a 50% improvement in median OS (HR=0.67) with 90% power and a 2.5% 1-sided type I error rate. This design requires 256 deaths. The expected number of PFS at the final analysis is 290 IA-PFS events. For this secondary objective, the analysis of IA-PFS will based on ruling out the null hypothesis of less than or equal to a 33% improvement (HR =0.75). With 290 IA-PFS events testing the IA-PFS null hypothesis of a HR equal to 0.75 at the 1-sided 0.05 level has 96.5% power. The approximate threshold for determining PFS is both clinically and statistically 47 significant is a 60% improvement in median PFS (HR = 0.62).

The total sample size is 332 eligible patients based on a median OS of 9 months. Assuming the ineligibility rate is 5%, the total accrual is 350 patients accrued over 27-36 months.

Accrual and time estimates assume a median 2-month delay between the **S1400** screening registration and sub-study registration for patients screened at progression and a median 9-month delay between screening and sub-study registration for patients pre-screened during prior treatment. Expected analysis times are stated from sub-study activation.

11.3 Analysis Plan

Primary analyses will be performed on an intent-to-treat basis. A stratified (using randomization stratification factors) log-rank test will be used to test the primary hypotheses related to OS comparing the two treatment arms at the levels specified below. A Cox proportional hazards (PH) model will be used to estimate the hazard ratios and associated confidence intervals. Analysis of response rates and toxicities will be performed using a chi-square or Fisher's exact test, as appropriate. The analysis of IA-PFS for the Phase III component will be done using a PH model score test (or in other words, a modified log-rank test at the PFS null hazard ratio of 0.75 [the 33% improvement in median PFS]).

Interim Analysis Plan:
Formal interim analyses are planned when 50% and 75% of the expected deaths have been observed with rules specified on the fixed-sample p-value scale. Evidence suggesting early termination of the trial for futility/harm will be if the alternative hypothesis of at least 50% improvement in OS for the experimental arm is rejected at the one-sided level 0.0025, using a PH model score test evaluated at the alternative hypothesis (e.g. Ha: HR=0.67 for OS). However, if the hazard ratio is between 1.0 and 1.10 at the first interim analysis at 50% analysis, the decision to either continue accrual or stop early for futility should also consider the shape of the survival curves evaluating the presence of non-proportional hazards with more limited follow-up. In such a case, the hazard ratio estimate at that time could be greater than 1 when in fact the hazard ratio estimate with a longer follow-up duration would show benefit with the investigational therapy.

Evidence suggesting early termination of the trial for superiority will evaluated at the second interim analysis alone (75% of expected OS events) and will be if the hypothesis of no difference in OS is rejected at the one-sided 0.0025 level using a log-rank test. A full description of the interim analyses is included in the statistical design and analysis plan in Section 11.0 of **S1400**. The following table details the anticipated analysis times based on an accrual rate of 180 and 360 patients per year to **S1400I.**

CLOSED EFFECTIVE 04/23/2018

SWOG Authorized Document

Summary of Interim and Final Analyses:

				144 patients/year		216 patients/year	
	Events	Estimated HR Boundary		N (eligible)	Time	N (eligible)	Time
Phase III interim analyses:		Futility	Efficacy				
50% OS	128	1.03	N/A	267	26	309	20
75% OS	192	0.94	0.67	332	33	332	25
Final Analysis OS	256			332	48	332	39
Final Analysis PFS	290						

* See note above regarding the futility considerations if the HR is between 1.0 and 1.10.

Sample size and analysis times for interim analyses are estimates. The actual numbers and timing will depend on the actual accrual rate and the event rates within the treatment arms. The analyses will occur upon the observation of the specified number of events.

If the study is closed for either futility or efficacy at any of the interim analyses, all secondary objectives will be evaluated.

11.4 Data and Safety Monitoring Committee

A Data and Safety Monitoring Committee will oversee the conduct of the study. The Committee consists of four members from outside of SWOG. Group members, 3 non-voting representatives from the National Cancer Institute (NCI), and the Group Statistician (non-voting). The members of this Committee will receive confidential reports every 6 months from the SWOG Statistics and Data Management Center, and will meet at the Group's bi-annual meetings as necessary. The Committee will be responsible for decisions regarding possible termination and/or early reporting of the study.

11.5 Translational Medicine

The statistical considerations for the **S1400I** Patient Reported Outcomes (PRO) are described in Section 18.2 of **S1400I**.

12.0 DISCIPLINE REVIEW

This section does not apply to this sub-study.

13.0 REGISTRATION GUIDELINES

See Section 13.0 of **S1400** for registration guidelines:

13.1 Registration Timing

Patients must plan to begin treatment within 7 calendar days after sub-study registration.

CLOSED EFFECTIVE 04/23/2018

SWOG Authorized Document

14.0 DATA SUBMISSION SCHEDULE

14.1 Data Submission Requirements

Data must be submitted according to the protocol requirements for **ALL** patients registered, whether or not assigned treatment is administered, including patients deemed to be ineligible. Patients for whom documentation is inadequate to determine eligibility will generally be deemed ineligible.

14.2 Master Forms

Master forms can be found on the protocol abstract page on the SWOG website (www.swog.org) and (with the exception of the sample consent form and the Registration Worksheet) must be submitted on-line via the Web; see Section 14.3 for details.

14.3 Data Submission Procedures

a. Data collection for this study will be done exclusively through the Medidata Rave clinical data management system. Access to the trial in Rave is granted through the iMedidata application to all persons with the appropriate roles assigned in Regulatory Support System (RSS). To access Rave via iMedidata, the site user must have an active CTEP-IAM account (check at https://ctepcore.nci.nih.gov/iam) and the appropriate Rave role (Rave CRA, Read-Only, Site Investigator) on either the LPO or participating organization roster at the enrolling site. To hold the Rave CRA role or CRA Lab Admin role, the user must hold a minimum of an AP registration type. To hold the Rave Site Investigator role, the individual must be registered as an NPIVR or IVR. Associates can hold read-only roles in Rave. If the study has a DTL, individuals requiring write access to Rave must also be assigned the appropriate Rave tasks on the DTL.

Upon initial site registration approval for the study in RSS, all persons with Rave roles assigned on the appropriate roster will be sent a study invitation e-mail from iMedidata. To accept the invitation, site users must log into the Select Login (https://login.imedidata.com/selectlogin) using their CTEP-IAM user name and password, and click on the “accept” link in the upper right-corner of the iMedidata page. Please note, site users will not be able to access the study in Rave until all required Medidata and study specific trainings are completed. Trainings will be in the form of electronic learnings (eLearnings), and can be accessed by clicking on the link in the upper right pane of the iMedidata screen.

Users that have not previously activated their iMedidata/Rave account at the time of initial site registration approval for the study in RSS will also receive a separate invitation from iMedidata to activate their account. Account activation instructions are located on the CTSU website, Rave tab under the Rave resource materials (Medidata Account Activation and Study Invitation Acceptance). Additional information on iMedidata/Rave is available on the CTSU members’ website under the Rave tab at www.ctsu.org/RAVE/ or by contacting the CTSU Help Desk at 1-888-823-5923 or by e-mail at ctsucontact@westat.com.

b. You may also access Rave® via the SWOG CRA Workbench via the SWOG website (http://swog.org).

For difficulties with the CRA Workbench, please email technicalquestion@crab.org

CLOSED EFFECTIVE 04/23/2018

SWOG Authorized Document

c. Institutions participating through the Cancer Trials Support Unit (CTSU) please refer to the CTSU Participation Table.

14.4 Data Submission Overview and Timepoints

a. WITHIN 7 DAYS OF SUB-STUDY REGISTRATION, SUBMIT:

S1400I Onstudy Form

Smoking Status Assessment Form

Baseline Tumor Assessment Form (RECIST 1.1)

Radiology reports from all scans performed to assess disease at baseline (NOTE: Upload reports via the Source Documentation: Baseline form in Rave®)

Submit to IROC via TRIAD for Central Radiology Review: Images from scans performed to assess disease at baseline as specified in Section 15.4.

b. IF PATIENT CONSENTS, SUBMIT SPECIMENS:

Specimens as specified in Section 15.0.

c. (For patients registered to **S1400I** after 9/1/2016 and participating in the **S1400I** PRO study) WITHIN 7 DAYS AFTER SUB-STUDY REGISTRATION AND AT WEEKS 3, 5, 7, 9, 11, 13, 25, AND 37, AND YEARS 1, 2, AND 3 SUBMIT:

S1400I Cover Sheet for Patient Reported Outcomes (PRO) Questionnaires

S1400I Patient Reported Outcomes (PRO) Questionnaire*
(Required at Prestudy, Weeks 3, 5, 7, 9, 11, 13, 25, and 37)

S1400I EQ-5D Questionnaire*
(Required at Prestudy, Weeks 5, 7, 9, 13, 25, 37, and Years 1, 2, and 3)

* NOTE: In addition to completing electronic forms, upload the patient-completed questionnaires via the Source Documentation: PRO form in Rave®. The scheduled PRO assessments should be completed even if the patient goes off treatment early.

d. IMMEDIATELY AFTER EACH CYCLE (Cycle = 14 days) OF TREATMENT, SUBMIT:

S1400I Treatment Form

S1400I Adverse Event Form

S1400I Laboratory Values Form

For Cycle 1 only: submit the **S1400I** Pre-Treatment Laboratory Values Form

e. WITHIN 7 DAYS AFTER EVERY DISEASE ASSESSMENT (WHILE ON TREATMENT, AND OFF TREATMENT PRIOR TO DISEASE PROGRESSION [see **S1400I** Section 9.0 for Disease Assessment Schedule]), SUBMIT:

CLOSED EFFECTIVE 04/23/2018

SWOG Authorized Document

Follow-Up Tumor Assessment Form (RECIST 1.1) documenting results of assessment

Radiology reports from all scans performed to assess disease at follow-up (NOTE: Upload reports via the Source Documentation: Follow-up form in Rave®)

Submit to IROC via TRIAD for Central Radiology Review: Images from scans performed to assess disease as specified in Section 15.4.

f. WITHIN 7 DAYS OF DISCONTINUATION OF TREATMENT, SUBMIT:

Off Treatment Notice documenting reasons for off treatment

S1400I Treatment Form

S1400I Adverse Event Form

S1400I Laboratory Values Form

Smoking Status Assessment Form

g. ONCE OFF TREATMENT SUBMIT EVERY 6 MONTHS FOR THE FIRST 2 YEARS FROM **S1400I** REGISTRATION, THEN AT THE END OF YEAR 3 SUBMIT:

Advanced NSCLC Follow-Up Form

Late Effects Form (if prior to treatment for progression or relapse or a second primary, and prior to non-protocol treatment, the patient experiences any severe [Grade ≥ 3] long term toxicity that has not been previously reported.)

Note: Patients who enroll on a new sub-study following progression must continue follow-up on this sub-study, in addition to follow-up on the new sub-study. See Section 14.4j.

h. WITHIN 7 DAYS OF PROGRESSION/RELAPSE, SUBMIT:

Site(s) of Progression or Relapse Form

Follow-Up Tumor Assessment Form (RECIST 1.1)

Radiology reports from all scans performed to assess disease at follow-up (NOTE: Upload reports via the Source Documentation: Follow-up form in Rave®)

Submit to IROC via TRIAD for Central Radiology Review: Images from scans performed to assess disease as specified in Section 15.4.

i. WITHIN 28 DAYS OF KNOWLEDGE OF DEATH:

Submit the Notice of Death documenting death information. In addition, if the patient was still on protocol treatment, submit materials specified in Section 14.4f or if patient was no longer on treatment, submit a final Advanced NSCLC Follow-Up Form.

j. Data Submission FOR PATIENTS WHO HAVE PROGRESSED AND WISH TO REGISTER TO A NEW SUB-STUDY:

WITHIN 7 DAYS OF PROGRESSION/RELAPSE:

Submit the **S1400** Request for New Sub-Study Assignment Form under **S1400** in Rave® Continue follow-up on **S1400I** per Sections 9.0 and 14.4g . See Section

CLOSED EFFECTIVE 04/23/2018

SWOG Authorized Document

14.6 of **S1400** for additional data submission requirements following request for new sub-study assignment.

15.0 SPECIAL INSTRUCTIONS

15.1 SWOG Specimen Tracking System (STS)

See **S1400** Section 15.1 for SWOG Specimen Tracking System (STS) instructions.

15.2 Correlative Studies and Banking (Optional for Patients)

Specimens for correlative studies and banking (submitted to the SWOG Biospecimen Bank – Solid Tissue, Myeloma and Lymphoma Division, Lab #201) are considered optional for the patient:

a. With patient's consent, specimens must be collected and submitted as follows:

1. Peripheral Blood:

Specimens must be collected at the following times:

- Prestudy (see Section 15.3 of **S1400I)**
  Note: If a patient provided blood at pre-screening or screening (see Section 15.3 of **S1400**) and registration to the sub-study is within 42 days from registration to **S1400**, then no additional pre-study blood specimen is required.
- Weeks 3, 7, 9. - Patients that go off treatment are not required to continue to submit specimens.
- First progression (irRC-Progression defined in Section 10.2 of **S1400I**) after study treatment

Collect approximately 8-10 mL of blood in EDTA tubes. Blood should be processed within one hour after venipuncture. If immediate processing within this time frame is not possible, then refrigerate (4°C) blood in EDTA tubes. The approximate time from collection to processing should be recorded as part of the patient's source documentation. EDTA tubes must be centrifuged at 800 g for 10 minutes at 4˚C for the collection of plasma. [Note: Sites that do not have a refrigerated centrifuge should spin at room temperature and ensure specimens are placed on ice (regular, not dry) immediately after being drawn and process rapidly.] Using a pipette, transfer the plasma to a15-mL centrifuge tube. Remove the buffy coat layer (thin white or gray layer of cells between the plasma and red blood cells) and split between two appropriately labeled 2-mL cryovials.

Spin the plasma in the 15-mL centrifuge tube at 800 x g for an additional 10 minutes. Avoiding any pelleted material, pipette the plasma into labeled cryovials at 0.5 ml aliquots. Plasma must be clear before freezing; no cells or debris should be present.

Plasma and buffy coat vials must be placed upright in a -80˚C freezer immediately after processing to ensure long-term viability.

Frozen plasma and buffy coat specimens should be shipped to the SWOG Biospecimen Bank on dry ice.

CLOSED EFFECTIVE 04/23/2018

SWOG Authorized Document

2. New Biopsy of Tumor at Time of Progression among responders to protocol treatment:

A new biopsy is strongly requested from patients who responded to protocol treatment (in the opinion of the treating physician) and then experienced disease progression for molecular analysis of molecular characteristics associated with mechanisms of resistance. New biopsy should be either bronchoscopy/surgical biopsy or CT guided biopsy.

Specimens should be collected at the following time point: within one month after progression.

Process the biopsy as FFPE material. The minimum requirement is a block or 12 unstained, charged, and unbaked 4-5 micron sections.

FFPE specimens (block or slides) should be shipped to the SWOG Biospecimen Bank at ambient temperature.

b. Specimen Submission

Samples for multiple patients may be shipped in batches, to the SWOG Biospecimen Bank – Solid Tissue, Myeloma and Lymphoma Division, Lab #201, at least every 3 months if not more frequently.

Specimen collection and submission instructions can be accessed on the SWOG Biospecimen Resources webpage (https://www.swog.org/member-resources/biospecimen-resources).

c. Specimen collection kits are not being provided for this submission; sites must use institutional supplies.

15.3 PD-L1 IHC Testing

Tissue remaining after the screening NGS testing will be sent from the SWOG Biospecimen Bank to University of Colorado Denver HSC. for PD-L1 Testing (see Section 18.1 for details). The specimen will be kept until there are no additional sub-studies for the patient to enroll in or the tissue is used up, whichever happens first. If the patient consented to future testing in **S1400**, any leftover tissue will remain at the SWOG Biospecimen Bank for future exploratory analysis.

15.4 Radiology Review (Required)

CT, PET/CT, and/or MRI images must be locally read and interpreted by the local site radiology service. Imaging exams must then be submitted to the Imaging and Radiation Oncology Core (IROC) at Ohio via TRIAD Imaging Submission procedures for central data collection and quality control (QC) check as well as retrospective central review.

a. CT, PET/CT, and/or MRI images must be submitted to IROC Ohio for central review at the following timepoints:

- Baseline
- Every 6 weeks for the first year, then every 3 months until progression and discontinuation of treatment.

All study participants must have a CT (or MR or PET/CT) exam prior to sub-study entry. Participants must then undergo additional imaging every 6 weeks for the first year, then every 3 months until progression of disease and discontinuation of

CLOSED EFFECTIVE 04/23/2018

SWOG Authorized Document

treatment. The same imaging modality used for the pre-treatment exam must be used for the post-treatment exams (see Section 10.1c). Each exam should be performed per **S1400** Section 18.1c. IROC will perform a QC of the imaging exams.

Clinical management and treatment decisions will be made by the treating physician based on local site assessments and other clinical appropriate considerations.

Central review of scans will not be triggered if the study will not be submitted to the FDA for FDA approval of the investigational therapy. Central review of scans will be triggered only if deemed necessary for FDA evaluation. A detailed description of the central radiology PFS review, including image acquisition parameters and image submission instructions, can be found in **S1400** Section 18.1c.

b. TRIAD Digital Image Submission

TRIAD is the American College of Radiology's (ACR) image exchange application. TRIAD provides sites participating in clinical trials a secure method to transmit DICOM RT and other objects. TRIAD anonymizes and validates the images as they are transferred.

1. TRIAD Access Requirements:

TRIAD will be the sole means of image transfer to the IROC Ohio. TRIAD should be installed prior to study participant enrollment to ensure prompt secure, electronic submission of imaging.

- Site staff who submit images through TRIAD will need to be registered with the Cancer Therapy Evaluation Program (CTEP) and have a valid and active CTEP-IAM account (see **S1400** Section 13.2).
- To submit images, the site user must be on the site's affiliate rosters and be assigned the 'TRIAD site user' role on the CTSU roster. Users should contact the site's CTSU Administrator or Data Administrator to request assignment of the TRIAD site user role.

2. TRIAD Installations:

After a user receives a CTEP-IAM account with the proper user role, he/she will need to have the TRIAD application installed on his/her workstation to be able to submit images. TRIAD installation documentation can be found by following this link https://triadinstall.acr.org/triadclient/

This process can be done in parallel to obtaining your CTEP-IAM account username and password.

If you have any questions regarding this information, please send an e-mail to the TRIAD Support mailbox at TRIAD-Support@acr.org.

15.5 **S1400I** PRO Questionnaire Administration Instructions

Instructions for administration of the **S1400I** Patient Reported Outcomes (PRO) Questionnaire and the **S1400I** EQ-5D Questionnaire are described in Section 18.2 of **S1400I**.

CLOSED EFFECTIVE 04/23/2018

SWOG Authorized Document

16.0 ETHICAL AND REGULATORY CONSIDERATIONS

16.1 ADVERSE EVENT REPORTING REQUIREMENTS

a. Purpose

Adverse event data collection and reporting, which are required as part of every clinical trial, are done to ensure the safety of patients enrolled in the studies as well as those who will enroll in future studies using similar agents. Adverse events are reported in a routine manner at scheduled times during a trial. (Directions for routine reporting are provided in Section 14.0.) Additionally, certain adverse events must be reported in an expedited manner to allow for timelier monitoring of patient safety and care. The following guidelines prescribe expedited adverse event reporting for this protocol.

b. Reporting method

This study requires that expedited adverse events be reported using the Cancer Therapy Evaluation Program Adverse Event Reporting System (CTEP-AERS). CTEP's guidelines for CTEP-AERS can be found at http://ctep.cancer.gov. A CTEP-AERS report must be submitted to the SWOG Operations Office electronically via the CTEP-AERS Web-based application located at: http://ctep.cancer.gov/protocolDevelopment/electronic_applications/adverse_events.htm.

c. When to report an event in an expedited manner

Some adverse events require 24-hour notification (refer to Table 16.1) via CTEP-AERS. When Internet connectivity is disrupted, a 24-hour notification is to be made to SWOG by telephone at 210/614-8808 or by email at adr@swog.org. Once Internet connectivity is restored, a 24-hour notification that was made by phone or using adr@swog.org must be entered electronically into CTEP-AERS by the original submitter at the site.

When the adverse event requires expedited reporting, submit the report within the number of calendar days of learning of the event, as specified in Table 16.1.

d. Other recipients of adverse event reports

The SWOG Operations Office will forward reports and documentation to the appropriate regulatory agencies and drug companies as required.

Adverse events determined to be reportable to the Institutional Review Board responsible for oversight of the patient must be reported according to local policy and procedures.

e. **Expedited reporting for investigational agents**

Expedited reporting is required if the patient has received at least one dose of the investigational agent(s) as part of the trial. Reporting requirements are provided in Table 16.1. The investigational agent(s) used in Arm 1 and Arm 2 of this study are nivolumab and ipilimumab. [Please note – For this sub-study the post dosage expedited reporting requirement window has been extended to **100** days rather than the normal 30 day requirement.] If there is any question about the reportability of an adverse event or if on-line CTEP-AERS cannot be used, please telephone

CLOSED EFFECTIVE 04/23/2018

SWOG Authorized Document

or email the SAE Specialist at the Operations Office, 210/614-8808 or adr@swog.org, before preparing the report.

Table 16.1:
Late Phase 2 and Phase 3 Studies: Expedited Reporting Requirements for Adverse Events that Occur on Studies under a Non-CTEP IND within 100 Days of the Last Administration of the Investigational Agent/Intervention[1] Nivolumab plus Ipilimumab, Arm 1 and Nivolumab Arm 2:

FDA REPORTING REQUIREMENTS FOR SERIOUS ADVERSE EVENTS (21 CFR Part 312)

NOTE: Investigators **MUST** immediately report to the sponsor (NCI) **ANY** Serious Adverse Events, whether or not they are considered related to the investigational agent(s)/intervention (21 CFR 312.64)

An adverse event is considered serious if it results in **ANY** of the following outcomes:

1) Death
2) A life-threatening adverse event
3) An adverse event that results in inpatient hospitalization or prolongation of existing hospitalization for ≥ 24 hours
4) A persistent or significant incapacity or substantial disruption of the ability to conduct normal life functions
5) A congenital anomaly/birth defect.
6) Important Medical Events (IME) that may not result in death, be life threatening, or require hospitalization may be considered serious when, based upon medical judgment, they may jeopardize the patient or subject and may require medical or surgical intervention to prevent one of the outcomes listed in this definition. (FDA, 21 CFR 312.32; ICH E2A and ICH E6).

ALL SERIOUS adverse events that meet the above criteria **MUST** be immediately reported to the NCI via CTEP-AERS within the timeframes detailed in the table below.

Hospitalization	Grade 1 Timeframes	Grade 2 Timeframes	Grade 3 Timeframes	Grade 4 & 5 Timeframes
Resulting in Hospitalization ≥ 24 hrs	10 Calendar Days			24-Hour 5 Calendar Days
Not resulting in Hospitalization ≥ 24 hrs	Not required		10 Calendar Days	

NOTE: Protocol specific exceptions to expedited reporting of serious adverse events (if applicable) are found in the Specific Protocol Exceptions to Expedited Reporting (SPEER) portion of the CAEPR.

Expedited AE reporting timelines are defined as:

- “24-Hour; 5 Calendar Days” - The AE must initially be reported via CTEP-AERS within 24 hours of learning of the AE, followed by a complete expedited report within 5 calendar days of the initial 24-hour report.
- “10 Calendar Days” - A complete expedited report on the AE must be submitted within 10 calendar days of learning of the AE.

[1]Serious adverse events that occur more than 100 days after the last administration of investigational agent/intervention and have an attribution of possible, probable, or definite require reporting as follows:

Expedited 24-hour notification followed by complete report within 5 calendar days for:

- All Grade 4, and Grade 5 AEs

Expedited 10 calendar day reports for:

- Grade 2 adverse events resulting in hospitalization or prolongation of hospitalization
- Grade 3 adverse events

May 5, 2011

CLOSED EFFECTIVE 04/23/2018

SWOG Authorized Document

f. **Additional Instructions or Exceptions to CTEP-AERS Expedited Reporting Requirements for Late Phase 2 and Phase 3 Studies Utilizing an Agent under a non-CTEP-IND:**

1. **Group-specific instructions.**

Supporting Documentation Submission - Within 5 **calendar days** submit the following to the SWOG Operations Office by fax to 210-614-0006 or mail to the address below:

- Printed copy of the first page of the CTEP-AERS report
- Copies of clinical source documentation of the event
- If applicable, and they have not yet been submitted to the SWOG Statistics and Data Management Center, copies of Off Treatment Notice and/or Notice of Death.

g. ***Reporting Secondary Malignancy, including AML/ALL/MDS***

1. A secondary malignancy is a cancer caused by treatment for a previous malignancy (e.g., treatment with investigational agent/intervention, radiation or chemotherapy). A secondary malignancy is not considered a metastasis of the initial neoplasm.

SWOG requires all secondary malignancies that occur following treatment with an agent under a Non-NCI IND to be reported via CTEP-AERS. Three options are available to describe the event.

- Leukemia secondary to oncology chemotherapy (e.g., Acute Myelocytic Leukemia [AML])

- Myelodysplastic syndrome (MDS)

- Treatment-related secondary malignancy

Any malignancy possibly related to cancer treatment (including AML/MDS) should also be reported via the routine reporting mechanisms outlined in each protocol.

Second Malignancy: A second malignancy is one unrelated to the treatment of a prior malignancy (and is NOT a metastasis from the initial malignancy). Second malignancies require ONLY routine reporting via CDUS unless otherwise specified.

For more information see:
https://ctep.cancer.gov/protocolDevelopment/electronic_applications/docs/aeguidelines.pdf

2. Any supporting documentation should be submitted to CTEP per NCI guidelines for AE reporting located at https://ctep.cancer.gov/protocolDevelopment/electronic_applications/docs/aeguidelines.pdf

A copy of the report and the following supporting documentation must also be submitted to SWOG Operations Office within 30 days:

- a copy of the pathology report confirming the AML/ALL /MDS diagnosis

CLOSED EFFECTIVE 04/23/2018

SWOG Authorized Document

- (if available) a copy of the cytogenetics report

SWOG
ATTN: SAE Program
4201 Medical Drive, Suite 250
San Antonio, Texas 78229

NOTE: If a patient has been enrolled in more than one NCI-sponsored study, the report must be submitted for the most recent trial.

h. **Reporting Pregnancy, Fetal Death, and Death Neonatal**

1. **Pregnancy** Study participants who become pregnant while on study; that pregnancy should be reported in an expedited manner via CTEP-AERS as **Grade 3 "Pregnancy, puerperium and perinatal conditions – Other (pregnancy)"** under the **Pregnancy, puerperium and perinatal conditions** SOC.

   Additionally, the pregnancy outcome for patients on study should be reported via CTEP-AERS at the time the outcome becomes known, accompanied by the same Pregnancy Report Form used for the initial report.

2. **Fetal Death/ Pregnancy loss** Pregnancy loss is defined in CTCAE as "Death in utero." and should be reported expeditiously as Grade 4 "Pregnancy loss" under the Pregnancy, puerperium and perinatal conditions SOC.

3. **Death Neonatal** Neonatal death, defined in CTCAE as "Newborn death occurring during the first 28 days after birth" that is felt by the investigator to be at least possibly due to the investigational agent/intervention should be reported expeditiously.

   A neonatal death should be reported expeditiously as Grade 4, "Death neonatal" under the General disorders and administration SOC.

   *Fetal death and neonatal death should **NOT** be reported as a Grade 5 event. If reported as such, the CTEP-AERS interprets this as a death of the patient being treated.*

   NOTE: When submitting CTEP-AERS reports for "Pregnancy, "Pregnancy loss", or "Neonatal loss", the Pregnancy Information Form should also be completed and faxed with any additional medical information to 301-230-0159. The potential risk of exposure of the fetus to the investigational agent(s) or chemotherapy agent(s) should be documented in the "Description of Event" section of the CTEP-AERS report.

   The Pregnancy Information Form is available at
   http://ctep.cancer.gov/protocolDevelopment/adverse_effects.htm.

CLOSED EFFECTIVE 04/23/2018

SWOG Authorized Document

17.0 BIBLIOGRAPHY

1 Jemal A, Bray F, Center MM. et al. Global Cancer Statistics CA Cancer J Clin 61:69–90, 2011 [PMID: 21296855]

2 NCCN Clinical Practice Guidelines in Oncology. Non-small cell lung cancer. v.3.2014. www.nccn.org.

3 Mellman I, Coukos G, Dranoff G. Cancer immunotherapy comes of age. Nature 29:480-89, 2011. [PMID: 22193102]

4 Brahmer JR, Drake CG, Wollner I, et al. Phase I study of single-agent anti–programmed death-1 (MDX-1106) in refractory solid tumors: safety, Clinical Activity, Pharmacodynamics, and Immunologic Correlates. J Clin Oncol 28:3167-3175, 2010. [PMID: 20516446]

5 Opdivo Prescribing Information, 2015. http://packageinserts.bms.com/pi/pi_opdivo.pdf.

6 Brahmer J, Reckamp KL, Baas P, et al. N Nivolumab versus Docetaxel in Advanced Squamous-Cell Non-Small-Cell Lung Cancer. N Engl J Med. PMID [PMID: 26028407], 2015.

7 Spigel DR, Reckamp KL, Rizvi NA, et al. A phase III study (CheckMate 017) of nivolumab (NIVO; anti-programmed death-1 [PD-1]) vs. docetaxel (DOC) in previously treated advanced or metastatic squamous (SQ) cell non-small cell lung cancer (NSCLC). J Clin Oncol 33:5s, (suppl; abstr8009), 2015. http://meetinglibrary.asco.org/content/144815-156.

8 Rizvi NA, Mazières J, Planchard D, et al. Activity and safety of nivolumab, an anti-PD-1 immune checkpoint inhibitor, for patients with advanced, refractory squamous non-small-cell lung cancer (CheckMate 063): a phase 2, single-arm trial. Lancet Oncol Published online February 20, 2015. [PMID: 25704439]

9 Paz-Ares L, Horn L, Borghaei H, et al. Phase III, randomized trial (CheckMate 057) of nivolumab (NIVO) versus docetaxel (DOC) in advanced non-squamous cell (non-SQ) non-small cell lung cancer (NSCLC). J Clin Oncol 33, (suppl; abstrLBA109), 2015.

10 Gettinger SN, Hellman MD, Shepherd FA, et al. First-line monotherapy with nivolumab (NIVO; anti-programmed death-1 [PD-1]) in advanced non-small cell lung cancer (NSCLC): Safety, efficacy and correlation of outcomes with PD-1 ligand (PD-L1) expression. http://meetinglibrary.asco.org/content/144603-156. J Clin Oncol 33:5s (suppl; abstr8025), 2015.

11 Amos S M., Duong CPM, Westwood JA, et al. Autoimmunity associated with immunotherapy of cancer. Blood 118:499-509, 2011.[PMID: 21531979]

12 Curran MA, Montalvo W, Yagita H, et al. PD-1 and CTLA-4 combination blockade expands infiltrating T cells and reduces regulatory T and myeloid cells within B16 melanoma tumors. PNAS 107: 4275-80, 2010.[PMID: 20160101]

13 Sznol M, Kluger HM, Callahan MK, et al. Survival, response duration, and activity by BRAF mutation (MT) status of nivolumab (NIVO, anti-PD-1, BMS-936558, ONO-4538) and ipilimumab (IPI) concurrent therapy in advanced melanoma (MEL). J Clin Oncol 32:5s (suppl; abstrLBA 9003).

14 Postow MA, Chesney J, Pavlick AC, et al. Nivolumab and ipilimumab versus ipilimumab in untreated melanoma. N Engl J Med 372:2006-2017, 2015.[PMID: 25891304]

CLOSED EFFECTIVE 04/23/2018

SWOG Authorized Document

18.0 APPENDIX

18.1 Translational Medicine - PD-L1 IHC Testing

18.2 Translational Medicine – **S1400I** Patient Reported Outcomes (PRO)

CLOSED EFFECTIVE 04/23/2018

SWOG Authorized Document

18.1 Translational Medicine - PD-L1 IHC Testing

Objectives

1. To evaluate if PDL-1 protein expression as defined and used in previous nivolumab studies for NSCLC is associated with improved clinical outcomes (response, PFS, OS) in patients treated with PD-1 antibody (nivolumab).
2. To evaluate if PDL-1 protein expression as defined and used in previous nivolumab studies for NSCLC is predictive for improved clinical outcomes in patients treated with nivolumab + ipilimumab versus nivolumab alone.
3. To explore whether H-score assessment can be used as a predictive biomarker analysis in this setting.

Background

Treatment of patients with PDL-1 or PD-1 antibodies have demonstrated very encouraging effect in patients with advanced NSCLC and has led to FDA approval of nivolumab for 2nd Line therapy of patients with squamous lung cancer. Most recently superior outcome data has been presented in patients with non-squamous lung cancer with the same agents, and FDA approval is expected in this patient population, as well. However, conflicting data have been presented regarding the role of PDL-1 protein expression (IHC) as predictive biomarker for response and outcome (PFS/OS) for these new agents. Quite consistent in all studies is the fact that response and outcome seem to be better in patients with PDL-1 positive tumors compared to those with PDL-1 negative tumors, but not all the studies have shown statistical significant differences. In the nivolumab registration study for 2nd line therapy for squamous lung cancer some difference (not statistical significant) between the two groups was seen. However, no data yet is known for PDL-1 assay as a predictive biomarker for combination therapy, which will be studied in the current clinical trial.

No predictive data has yet been presented using an H-score system related to PDL-1, and this study provides an opportunity to also study alternating assessment methods using the same slides.

PD-L1 IHC testing will be performed on tumor specimens from patients registered to **S1400I**. Tumor specimens will be shipped from the SWOG Biospecimen Bank at Nationwide to University of Colorado Denver HSC on a bi-weekly basis. The SWOG Statistics and Data Management Center will provide Nationwide with the list of patient specimens to ship to University of Colorado Denver HSC. All specimens must be entered and tracked using the online SWOG Specimen Tracking System (STS). Shipments to University of Colorado Denver HSC will be accompanied by pathology report, SWOG patient ID, specimen ID, sample collection date, and sample type.

Experimental research techniques/tests employed and expertise

The DAKO assay previously used in nivolumab studies will be applied primarily with the use of the well-defined cut-offs (1%, 5%, 10%). In addition, an H-score evaluation will be applied as explorative. The H-score protein assessment for lung cancer was first described by the TM Study Chair (Dr. Hirsch) applied in EGFR in a publication in J clin Oncol in 2003. It has since been applied by other investigators for different targets in multiple publications, including the Hirsch lab. Dr. Hirsch's lab is currently involved in an international "comparison" project of the available PDL-1 assays used in the clinical trials by the different companies. Dr. Hirsch's lab is also involved in a large PDL-1 project supported by BMS and performed through NCTN, which has the goal to understand this specific assay's performance on different types of specimens (i. e. large specimens versus small biopsies versus cytology). Dr. Hirsch is co-chair for that study, and as such has special experience with the DAKO/BMS PDL-1 assay, which is planned to be used in this TM study proposal.

CLOSED EFFECTIVE 04/23/2018

SWOG Authorized Document

CLOSED EFFECTIVE 04/23/2018

Dr. Fred Hirsch is the key contact for questions regarding this translational medicine study:

Lab# 167
Fred R. Hirsch, M.D., Ph.D. (Translational Medicine)
University of Colorado Cancer Center
P.O. Box 6511, MS 8117
Aurora, CO 80045
Phone: 303/724-3858
E-mail: fred.hirsch@ucdenver.edu

Statistical Plan

Based on current tissue submission data, it is estimated that approximately 60% of eligible patients enrolled on S1400I will have sufficient tissue leftover after FMI genetic profiling to have PD-L1 expression analyzed. Furthermore, based on Rizvi, et al [Lancet Oncol. 2015 Mar; 16 (3):257-65. doi: 10.1016/S1470-2045(15)70054-9. Epub 2015 Feb 20]. It is anticipated that for approximately 13% of these patients the specimens submitted will turn out not to be evaluable for PD-L1 testing. Therefore, with 332 patient enrolled on S1400I, we expect 174 patients in all of the analysis described below.

First, we will evaluate if PD-L1 expression, as measured by the DAKO assay, is associated with improved clinical outcomes, overall survival (OS), progression-free survival (PFS), and response rate (confirmed and unconfirmed, complete and partial responses), in all patients treated with nivolumab (i.e. both study treatment arms combined). Using a definition of PD-L1 positivity as an IHC score of at least 5% (≥ 5%), it is expected, based on Rivzi et al, that of patients with evaluable specimens 35% will be PD-L1 positive and 65% will be PD-L1 negative.

Assuming that the median overall survival among PD-L1 negative patients is 9 months, with 174 patients enrolled over 36 months and an additional 12 months of follow-up, there will be approximately 83% power to detect a hazard ratio of 1.6, corresponding to a median OS of 14.4 months for PD-L1 positive patients, using a one-sided 0.05 level log-rank test.

Assuming a median PFS of 3.5 months in PD-L1 negative patients, there will be approximately 81% power to detect a hazard ratio of 1.5, corresponding to a median PFS of 5.25 months in PD-L1 positive patients, using a one-sided 0.05 level log-rank test.

In addition, we will assess whether patients who are PD-L1 positive have a better probability of response (confirmed and unconfirmed, complete and partial responses). With 174 patients we will have approximately 80% power to be able to detect a difference of 21% or greater using a one-sided 0.05 level test of proportions.

Next, we will evaluate whether PD-L1 status is predictive for improved overall survival for patients receiving the combination of nivolumab + ipilimumab by fitting a cox model with an interaction term for treatment effect. If we assume no treatment effect in the single agent nivolumab arm, and no treatment effect for PD-L1 negative patients receiving the combination, and further assuming the null median OS is 9 months, there will be approximately 83% power, using a one-sided test at the 0.15 type-1 error level, to detect a OS hazard ratio of 2, corresponding to an improvement to a median OS of 18 months in PD-L1 positive patients receiving the combination of ipilimumab plus nivolumab.

We will evaluate a treatment interaction effect for PFS in the same manner as described above. Once again, we assume no treatment effect in the single agent arm and no treatment effect for PD-L1 negative patients in the combination arm. If we further assume the null median PFS is 3.5 months, there will be approximately 81% power, using a one-sided test at the 0.15 type-1 error level, to detect a PFS hazard ratio of 1.9, corresponding

SWOG Authorized Document

to an improvement in median PFS to 6.65 months for PD-L1 positive patients receiving the combination of nivolumab plus ipilimumab.

To evaluate if there is a treatment interaction effect on the probability of response, a logistic regression model will be fit. However, power for testing the interaction term at the 0.15 level will be at best modest (< 70%) to detect a very large difference (OR > 4).

All of the analyses previously described will be repeated using the alternative cut-offs for defining PD-L1 positivity as an IHC score of at least 1% (≥ 1%) and again for an IHC score of at least 10% (≥ 10%).

In addition, we will investigate in a preliminary manner whether PD-L1 expression by IHC is associated with improved clinical outcomes (OS, PFS, response) among all patients treated with nivolumab (i.e. both arms combined) as well as explore whether there is a treatment interaction effect for patients treated with the combination of nivolumab plus ipilimumab. The raw marker data (H score) will be rescaled to quantiles and a Cox regression model will be fit treating the rescaled data as a continuous variable.

Assuming 36 months accrual and 12 months of follow-up, there will be 86% power to detect a 1.5 hazard ratio (i.e. a 50% improvement in median PFS) between patients with H score levels in the third quantile versus patients with H score levels in the first quantile using a one-sided test with an alpha = 0.05.

To evaluate if patients receiving the combination of nivolumab + ipilimumab with PD-L1 expression by IHC have a better PFS than patients with PD-L1 expression treated on the control arm, we will fit a Cox regression model with a treatment interaction term to evaluate if there is a difference in treatment effect associated with marker values. The H Score data will be rescaled to quantiles. Assuming 36 months accrual and 12 months of follow-up, there will be 81% power to detect a 1.9 treatment interaction hazard ratio between the third quartile and the first quartile.

The association between PD-L1 expression and overall survival will be analyzed with Cox regression techniques in the same manner described above. The association between PD-L1 expression and response rates (confirmed and unconfirmed, complete and partial responses) will be evaluated by fitting logistic regression models to the rescaled data. Patients whose exact response cannot be determined due to inadequate follow-up assessments will be included in the analysis as non-responders.

Laboratory
University of Colorado Denver HSC will serve as the central laboratory for testing PD-L1 expression in patients who register to **S1400I**.

Lab# 167
Fred R. Hirsch, M.D., Ph.D. (Translational Medicine)
University of Colorado Cancer Center
P.O. Box 6511, MS 8117
Aurora, CO 80045
Phone: 303/724-3858
E-mail: fred.hirsch@ucdenver.edu

CLOSED EFFECTIVE 04/23/2018

SWOG Authorized Document

Specimen Requirements

The preferred thickness is 4 micron unstained tissue sections; although 5 micron unstained tissue sections are allowable. For patients with tumor blocks at Nationwide, Nationwide will prepare 4 micron unstained slides. At least 100 tumor cells is defined to be sufficient viable tumor tissue for PD-L1 IHC testing. Fine needle aspirates are not acceptable. A minimum of 3 unstained tissue sections along with a hematoxilyn-eosin (H&E)-stained slide or Aperio H&E-stained image will be sent to University of Colorado Denver HSC for PD-L1 IHC testing for consented patients with sufficient tissue.

Specimen Flow Diagram

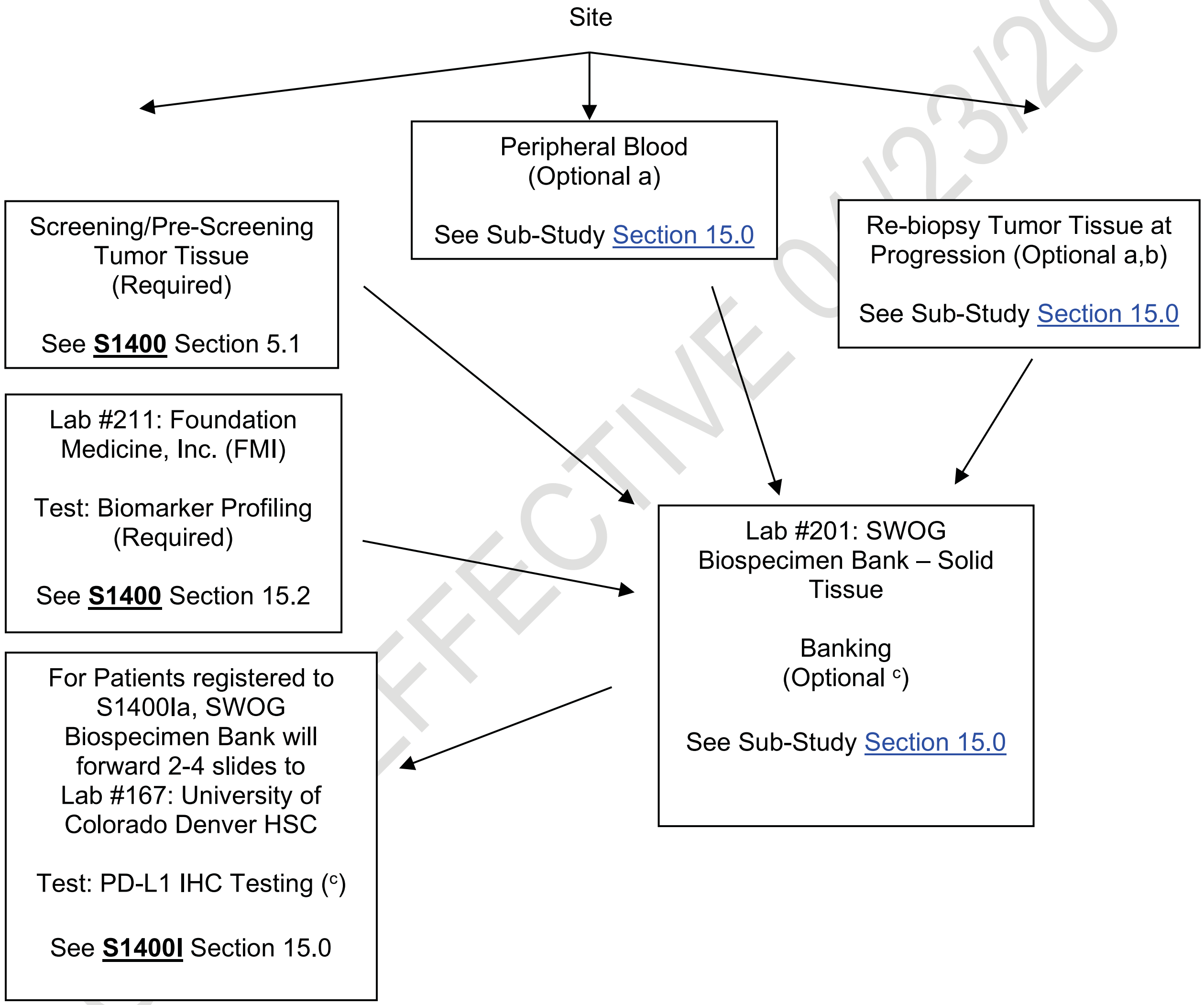

a With patient's consent.
b Among patients who initially responded to protocol treatment.
c Remaining tissue will be sent to the SWOG Biospecimen Bank-Solid Tissue, Myeloma and Lymphoma Division, Lab #201, for use of the Translational Medicine studies within any sub-study the patient is enrolled in. SWOG Biospecimen Bank will prepare and ship the required specimens to the appropriate laboratory. The specimen will be kept until there are no additional sub-studies for the patient to enroll in or the tissue is used up, whichever happens first. If the patient consented to future testing in **S1400**, any leftover tissue will remain at the SWOG Biospecimen Bank for future exploratory analysis.

CL[illegible] EFFECTIVE 0[illegible]/23/2018
SWOG Authorized Document

18.2 Translational Medicine – S1400I Patient Reported Outcomes (PRO)

Objectives

1. To compare symptom status by treatment arm using a validated PRO symptom measure, the M. D. Anderson Symptom Inventory (MDASI-LC) Severity Score.

2. To identify PRO-based symptoms prognostic for time to progression.

3. To develop a statistical model that identifies a PRO-based symptom score optimally prognostic for survival outcomes.

4. To evaluate functional status/interference of symptoms with life as a prognostic variable for time to progression.

5. To compare treatment-related toxicities by treatment arm at each assessment time.

6. To compare EQ-5D Index scores by treatment arm.

7. To collect psychometric information (reliability and validity) data for the Non-Small Cell Lung Cancer Symptom Assessment Questionnaire (NSCLC-SAQ).

Background

Advanced squamous cell lung cancer (NSCLC) is associated with a significant symptom burden. Patients with NSCLC suffer from high levels of physical symptoms, the most common being cough, shortness of breath, and chest pain. *(1)* These symptoms can have serious consequences on patient functionality and quality of life and may serve as a predictor of performance status. *(2)* The ability to provide direct evidence reflecting the improvement, stabilization, or worsening of disease symptoms is invaluable to demonstrating clinical benefit, beyond survival, and informing decision making. *(3,4,5,6)*

Lung-MAP is a large scale, biomarker driven set of single arm trials for patients with advanced stage squamous cell lung cancer whose molecular profile matches a targeted therapy for that profile; it is an example of an umbrella trial. *(7)* Depending on results, some of these trials will progress to Phase III, randomized trials. Patients whose molecular profile does not match a targeted therapy will be assigned to a specific "non-match" sub-study and randomized to either treatment with a new drug or drug combination or standard of care (SOC); trials other than **S1400I** are in development. The endpoints used in Lung-MAP (PFS, OS, and RR), currently, do not adequately capture the complex symptom profile experienced by patients, which may result from the disease, the therapy, or both. We propose to fill this potential gap in the knowledge base through the addition of a set of patient-reported outcomes (PROs). Patients on **S1400I** are randomized to either SOC (Nivolumab) or the combination regimen (Nivolumab + Ipilimumab). This trial structure provides an ideal setting for evaluating the information provided by PRO measures and assessing how well PRO measures track with disease symptoms and other clinical outcomes. In addition, we have the opportunity to obtain preliminary survivorship status for this group of patients at the three yearly follow-up assessments for clinical status; we propose using the EQ-5D for this purpose.

PRO Instrument

The Lung-MAP PRO instrument for **S1400I** will include the following items that assess self-reported symptom and functional status of patients with squamous cell lung cancer. Patients will be asked to complete a total of 40 items.

CLOSED EFFECTIVE 04/23/2018

1. **MDASI-LC** *(8,9,10,11,12)*: **21 items**. The M. D. Anderson Symptom Inventory – Lung Cancer (MDASI-LC) asks patients to rate the severity of 13 core symptoms that are common in cancer patients once treatment begins: fatigue, sleep disturbance, pain, drowsiness, poor appetite, nausea, vomiting, shortness of breath, numbness, difficulty remembering, dry mouth, distress, and sadness. Three additional lung-specific items were added for the MDASI Lung Cancer module (coughing, constipation, and sore throat). The MDASI-LC investigators *(13)* suggest elimination of the sore throat item if the treatment regimen does not include radiation therapy. As **S1400I** does not include radiation therapy, the MDASI-LC will not include this item. Patients rate each symptom's presence and greatest severity in the previous 24 hours on an 11-point (0–10) scale, with 0 representing “not present” and 10 representing “as bad as you can imagine.” The MDASI-LC questionnaire generates a mean Core symptom score for the 13 general symptoms and a mean Severity score for the Core plus two lung cancer symptom items for a total of 15 items. The MDASI-LC Interference score (six items) will also be included to address how symptoms interfere with the patient’s general activity/functional status in the last 24 hours; these items also use a 0 to10 response scale. *(14,15,16,17,18)* The MDASI-LC form will include 21 items for **S1400I**. Only the scores from the validated MDASI-LC questionnaire will be used to formally compare treatment arms in **S1400I**. This approach follows an accepted practice of only using validated PRO measures as endpoints in cancer clinical trials. *(19,20,21)*

2. **NSCLC-SAQ** *(22)*: **7 items.** The PRO Consortium’s NSCLC Working Group is developing the NSCLC-SAQ measure. Qualitative research has recently been conducted for this measure. An early report of qualitative research findings is available that addresses expert review to identify concepts as well as interviews with 51 patients to review draft concepts/items and patient understanding of these items; this report reflects data from the first of three waves of cognitive debriefing regarding the content validity of the NSCLC-SAQ. *(23)* To gather data that can be used in the validation of the new NSCLC-SAQ measure in the **S1400I**, we propose administering all seven items of the NSCLC-SAQ plus the MDASI-LC. There is conceptual overlap of the NSCLC-SAQ with the MDASI-LC, which generates some degree of patient response burden; this similarity in content will also increase the potential for a larger correlation between the two symptom measures. Allowing this redundancy is partly necessary in order to anchor the NSCLC-SAQ items to a known, validated instrument for documentation of psychometric properties of the new measure. The time frame does differ for these two measures, with the NSCLC-SAQ using a 7-day recall period to address symptom severity or frequency whereas the MDASI-LC uses the past 24 hours for responses. The response option format also differs with the MDASI using a 0 to 10 scale and the NSCLC-SAQ using five response options for each item.

3. Single Items Used for Validation of New Measure: **2 Items**. A single item, patient-rating of performance status *(24,25,2627)* based on the ECOG Performance Status measure *(28)* will be assessed as a surrogate for physical status. The MDASI-LC Interference score will also be used for this purpose. A second single item Global Rating of Change (GRC) in symptom status (Personal Communication, Charles Cleeland, February, 2016) will be obtained to identify minimally important differences in symptom status for both measures and to document the new measure’s responsiveness to change. *(29,30,31)* The symptom severity GRC item will ask patients to rate change in the severity of symptoms “ since the last time you completed this questionnaire” (much better, better, nearly the same, worse, much worse). The GRC item is included in the **S1400I** Patient Reported Outcomes (PRO) Questionnaire; this item is not to be administered at PreStudy but should be completed starting at Week 5 and at all subsequent assessments through week 37 post-registration.

CLOSED EFFECTIVE 04/23/2020
SWOG Authorized Document

4. **Four** Patient-Reported Outcomes Common Terminology Criteria for Adverse Events (PRO-CTCAE) *(32)* will be included to address three toxicities (fatigue, pruritis, and diarrhea) common to the two agents used in this trial. *(33)* Fatigue treatment-related toxicity will be assessed with the PRO-CTCAE Fatigue severity item (What was the SEVERITY of your FATIGUE, TIREDNESS, OR LACK of ENERGY at its WORST [none/Mild/Moderate/Severe/Very severe]). The degree to which fatigue interferes with daily functioning will also be assessed with one PRO-CTCAE item (How much did FATIGUE, TIREDNESS, OR LACK OF ENERGY INTERFERE with your usual or daily activities?) Pruritis and dermatitis will be assessed with the PRO-CTCAE Itchy Skin item (What was the SEVERITY of your ITCHY SKIN at its WORST [None/Mild/Moderate/Severe/Very severe]). Diarrhea will be assessed with the PRO-CTCAE Diarrhea item (How OFTEN did you have LOOSE OR WATERY STOOLS

   (DIARRHEA)[Never/Rarely/Occasionally/Frequently/Almost constantly/ Not applicable]). The time frame for these items is the last 7 days. Only Fatigue is assessed by the MDASI-LC, not the other two symptoms found to be associated with the two treatments. The MDASI-LC and the PRO-CTCAE response options differ, with the PRO-CTCAE options more tailored to the standard physician-rated CTCAEs. For this reason, we feel it is important to add these three symptoms and one fatigue interference item to the assessment package because of the interest in being able to separate disease- and treatment-related symptoms.

5. **S1400I** PRO study will also administer the EQ-5D health utility measure *(34,35,36,37)*: **6 Items**. These items addressing different health dimensions are rated with respect to no problems, some problems, or extreme problems. **S1400I** will include the single item visual analogue scale (VAS) to measure overall health status. We have used a version of the EQ-5D VAS in **S1007**. In this version, in addition to marking the point on the line reflecting the patient's "health state today", we ask the patient to write the number in the provided box on the form. This substantially improves data capture and does not require staff time to record this information. Because the EQ-5D will be administered both early in follow up with the other cited measures, as well as later in follow-up (at years 1, 2, & 3) to assess survivorship status, it will be presented as a separate form.

Patient Questionnaires: Instructions for Administration

Method:
The **S1400I** Patient Reported Outcomes (PRO) Questionnaire will be administered to patients via paper-and-pencil at nine time points: Prestudy (within 14 days prior to registration) and at the beginning of Weeks 3, 5, 7, 9, 11, 13, 25, and 37 weeks after **S1400I** registration. In addition, the **S1400I** EQ-5D Questionnaire will be administered at ten time points. Prestudy (within 14 days prior to registration), Weeks 5, 7, 9, 13, 25, 37, and at years 1, 2, and 3. The scheduled PRO assessments should be completed even if the patient goes off treatment early. Note: Patients registered to **S1400I** prior to 9/1/2016 are not eligible for the PRO study.

Frequency and Timing:
In general, the assessment schedule emphasizes the early (first 3 months) assessments in order to minimize missing data given the advanced stage disease status of study patients who have a median survival of 9 months and to coincide with patient visits so as to link clinical assessments with patient symptom assessments. The administration of four PRO-CTCAE items will occur at the same times physicians are rating CTCAEs in order to allow for a mapping of symptom and disease progression status as well as to provide an opportunity to see if early change in symptom status is associated with clinical outcomes as reported by Eton et al. *(38)* However, we will also include PRO assessments at two

CLOSED EFFECTIVE 04/13/2018

SWOG Authorized Document

later time points (25 and 37 weeks) in order to evaluate the extent of longer-term symptom and functional deterioration by treatment arm Exploratory analyses of the EQ-5D measure will also be conducted at years 1, 2, and 3 for preliminary data on survivorship outcomes at these later time points

As currently designed, the estimated time to administer the components of the PRO questionnaire is based on the estimate of 2-5 minutes for the MDASI and its module (21 items) *(39); a total of 10 minutes is estimated for* the addition of 7 more NSCLC-SAQ items and the inclusion of the additional items used for the NSCLC-SAQ validation. The time to complete the four PRO-CTCAE toxicity items will be very quick for patients, likely within one minute. *(40)* Also, patients were found to complete the EQ-5D in only a few minutes. *(41)* Therefore, we estimate that the inclusion of four PRO-CTCAE and the six EQ-5D items will add two to three minutes, so the total time to complete all 40 items will be approximately ≤ 15 minutes. The assessment schedule will also minimize data loss as collection will be linked with the timing of some clinical assessments and submitted simultaneously; staff and patient burden will also be minimized. For the Global Rating of Change (GRC) item, patients will be asked to respond with respect to change in symptom status since the last time the patient completed the questionnaire.

Four of the PRO sets of measures (MDASI-LC, NSCLC-SAQ, two single items measures to be used for validation of the NSCLC-SAQ, and the four PRO-CTCAE items) will be included in a separate, single form. This form, the **S1400I** Patient Reported Outcomes (PRO) Questionnaire will be administered only through week 37. In addition, the GRC item is not administered at PreStudy because it is addressing the patient's perception of change in symptom status. Patients are to be instructed to begin answering the GRC item at week 3.

We are also including longer term (years 1, 2, and 3) follow-up assessments using only the **S1400I** EQ-5D Questionnaire as a measure of survivorship status. The assessment times correspond to protocol-specified clinical outcome assessments. Patients will be asked to complete the **S1400I** EQ-5D Questionnaire in the clinic or by phone if the patient does not come to the clinic. The EQ-5D will be presented to patients in a separate form, the **S1400I** EQ-5D Questionnaire and this form will have a separate cover sheet, the **S1400I** Cover Sheet for the **S1400I** EQ-5D Questionnaire, because it is to be administered for a longer time period. Questionnaires may be administered by phone if needed in order to follow the PRO schedule for patients who discontinue study drug early.

Recall Period:
The recall period for the MDASI-LC is the last 24 hours; the recall period for the NSCLC-SAQ and the PRO-CTCAE items is the last seven days. Research staff should remind patients to answer questions accordingly. Note, studies have shown psychometric properties remain consistent independent of recall period. *(42)* The recall period for the EQ-5D is your health status on the day the measure is completed so its recall period is more similar to the MDASI-LC measure. *(43)*

Administration of Questionnaires:
1. The first time the patient completes the questionnaires: Please read to the patient the instructions attached to each section. Explain the specific administration times for this protocol. Patients should be directed to report all symptoms and limitations whether or not they are related to the cancer or its treatment.
2. It is permissible to assist patients with completing the questionnaires being careful not to influence the patient's response. Note what assistance was required and indicate reason in the Comments section of the **S1400I** Cover Sheets for the two PRO forms (e.g., elderly, too sick, etc.). Discourage family members from: 1) being present while the patient completes the questionnaire and/or 2) influencing patient responses to the questions.

CLOSED EFFECTIVE 04/23/2018

SWOG Authorized Document

3. It is very important to review the questionnaires after completion by the patient to be sure all of the questions have been answered and that only one answer is marked. If the patient has marked more than one answer per question, ask the patient which answer reflects how he/she is feeling. If the patient skipped a question, tell the patient that a question was not answered and ask if he/she would like to answer the question. Always give the patient the option to refuse. Indicate on the form by the question that the patient did not want to answer this question.
4. If a patient refuses or cannot complete the questionnaire for some reason, then this must be documented in the Comments section of the **S1400I** Cover Sheets, depending on the form being completed by the patient; he/she should be asked to do so at the next scheduled administration time.
5. If a patient cannot be available on the scheduled date of assessment for any reason, follow the established protocol windows per Best Practices for SWOG Studies. http://swog.org/Visitors/QA/Documents/Best%20Practices%20upddate.pdf. Alternatively, if a patient is too sick to complete the questionnaires in the clinic on the scheduled date, the questionnaire can be mailed to the patient or sent home with him/her. A telephone interview must be scheduled and completed within the allowed window (from the scheduled date of the assessment) per Best Practices for SWOG Studies. Patient responses to questionnaire items are to be obtained during the telephone interview while the patient is looking at a copy of the questionnaire. The date of the telephone interview is to be noted in the Comments section of the **S1400I** Cover Sheets for the PRO forms.

Additional Quality Control Procedures:

1. When a patient is registered to **S1400I**, a calendar should be made with dates of upcoming patient-completed questionnaires noted. A copy of this calendar can be given to the patient with the notation that the questionnaires should be completed before receiving treatment. You may wish to photocopy the Study Calendar, Section 9.0, and include the patient's name and specific dates. A copy of this should be kept in the patient file.
2. If a patient goes off protocol treatment, continue to administer the patient completed questionnaires according to the protocol-defined assessment schedule (time from registration date).
3. The Nurse PRO/QOL Study Coordinator, will monitor compliance on a regular basis using the Expectation Report and the Medidata RAVE system. Medidata RAVE will provide reminders of upcoming PRO assessments for a patient.

Anyone involved in the collection of quality of life/PRO data in SWOG trials should review the Patient Reported Outcome Training narrated slide program available on the SWOG website (www.swog.org, CRA Training, Tools of the Trade). This program is designed to standardize the way quality of life data are collected from patients. Contact cancercontrolquestion@crab.org for questions regarding the quality of life/PRO assessments OR the Nurse PRO/QOL Study Coordinator: Susan S. Tavernier, PhD, APRN-CNS, AOCN.

Nurse PRO/QOL Study Coordinator
Susan S. Tavernier, PhD, APRN-CNS, AOCN
Assistant Professor
Accelerated Nursing Program Coordinator
Idaho State University School of Nursing
Phone: 208/373-1783
Email: tavesusa@isu.edu

Eligibility

All patients must be eligible for the clinical component of **S1400I** and able to complete the PRO instruments in English. That is, the Prestudy PRO questionnaire forms are required

CLOSED EFFECTIVE 04/23/2018

SWOG Authorized Document

for study eligibility if the patient can complete them in English. If a patient is deemed ineligible during the trial but continues protocol treatment, he/she should continue the PRO assessments as scheduled.

Endpoint(s) to be Used in Analyses

For the **S1400I** protocol, progression free survival (PFS) and overall survival (OS) are the primary endpoints and the response rate (RR) is a secondary endpoint. The reduction of symptoms based on the validated PRO measures will also be considered a secondary endpoint. The PRO endpoints are described in the PRO Instrument section, above.

Experimental Research Techniques/Tests Employed and Expertise of PI

Research staff should be trained to administer PRO forms by viewing a training module available on the SWOG website [www.swog.org]; from the home page, the program can be found at the QUICKLINKS section as noted above. For example, this training program indicates that research staff should not influence the patient's responses to the questionnaires. Non-SWOG research staff can access the PRO training program directly on the SWOG home page. Research staff will need to enter the reported data into the online Medidata RAVE system.

Efforts to minimize missing data are critical to obtaining interpretable results. Again, the PRO training module addresses methods for reducing missing data.

Statistical Plan

Primary Endpoints

PRO assessments will occur in conjunction with the clinical follow-up schedule already in place for **S1400I** and are scheduled for baseline and weeks 3, 5, 7, 9, 11, 13, 25, and 37. Two primary endpoints will be assessed, corresponding to an early assessment at week 7 when clinically meaningful differences by arm may first occur, and a late assessment at week 13 to identify potentially long-term differences.

The primary endpoint is the MDASI-LC Severity score. With respect to a minimally important difference (MID) between arms, Mendoza et al. *(44)* identified a difference of 1.06 points in the MDASI severity index (MDASI core 13 items plus the lung-specific items) between lung cancer patients with good vs. poor ECOG performance status, suggesting that such a difference is clinically meaningful. Also, prior literature in other disease settings suggests an MID for the MDASI in the range of 0.98 to 1.21 points. *(45,46,47)* Based on these data, we specify an average difference of 1.0 point in the MDASI severity score to be considered clinically meaningful.

Relying on the work by Mendoza et al. *(48)*, the standard deviation for assessment of the severity scale at 6 weeks of chemotherapy treatment was 1.88 points and at 12 weeks was 1.71 points. (Cleveland, personal communication; Mendoza, personal communication). For power calculations, we assume a higher (more conservative) standard deviation of 2.0 points for both time points, corresponding in general to an approach wherein the MID is half a standard deviation. *(49,50)*

The study specifies a median overall survival of 9 months on the standard arm, which suggests that approximately 10% of patients will have died by the time of their assessment at week 7 under exponential assumptions. In addition, conservatively, we assume another 10% will drop out due to worsening disease, and 10% will be non-adherent. Note that in power calculations, the 10% non-adherence rate reduces the nominal effect size of a 1.0

CLOSED EFFECTIVE 04/13/2023

SWOG Authorized Document

point target difference to 0.90, while the total 20% dropout rate inflates the estimated sample size by a factor of 1/(1-0.2) or 25%. The study anticipates enrolling n=332 eligible patients. Using a two-arm normal design, a two-sided alpha=.025 test (to account for multiple comparisons using Bonferroni), and the parameters specified above, a difference of 1.0 points between arms can be identified with 92% power.

For the week 13 endpoint, with a median overall survival of 9 months on the standard arm, approximately 20% of patients will have died. In addition, we assume 15% will drop out due to worsening disease and 15% will be non-adherent. With all other parameters the same, a difference of 1.0 points between arms can be specified with 81% power.

Power will vary as a function of the observed standard deviation, the total dropout, and non-adherence as shown in the table below.

Table: Power as a Function of Observed SD and Dropout

		**Observed Standard Deviation**				
**Total Dropout***	**Non-Adherence**	**1.8**	**1.9**	**2.0**	**2.1**	**2.2**
**15%**	10%	98%	96%	94%	91%	88%
	15%	96%	94%	91%	88%	84%
**20%**	10%	97%	95%	**92%**	89%	86%
	15%	95%	92%	89%	85%	82%
**25%**	10%	96%	93%	90%	87%	84%
	15%	93%	90%	87%	83%	79%
**30%**	10%	94%	91%	88%	85%	81%
	15%	91%	88%	84%	80%	76%
**35%**	10%	92%	89%	86%	82%	78%
	15%	89%	85%	**81%**	77%	73%

* Includes dropout due to worsening disease and death.

Consistent with the design, the co-primary analyses of the week 7 and week 13 severity scores will be conducted using multiple linear regression analyses, adjusting for stratification factors and the baseline severity score as covariates. We will also conduct longitudinal modeling of the outcome measures over time. Power for the longitudinal analysis will be greater since the addition of all available MDASI severity scores over time will provide more information. For longitudinal modeling, linear mixed models will be used. The potential for differential dropout by arm will be mitigated by reminder notifications to site investigators to encourage proper assessment and submission of forms at every required time point for all patients. Dropout patterns will be monitored on an ongoing basis. Nonetheless the potential for non-random dropout exists. Cohort plots will be prepared to examine the extent to which missing data are informative (i.e., scores are higher (worse) for patients just before their data are missing for the subsequent assessment). If there is evidence of non-random dropout, pattern-mixture models will be utilized as a sensitivity analysis. *(51,52,53)* Covariates for longitudinal modeling will include intervention assignment, assessment time, their interaction, the baseline score, and a limited set of potential confounding variables (*e.g.,* age).

<u>Secondary Endpoints</u>

The following additional secondary endpoints will be examined:

A) Descriptive data will be presented, including the difference in the MDASI-LC mean Severity scores for the two treatment arms at baseline and weeks 3, 5, 7, 9, 11, 13, 25, and 37.

CLOSED EFFECTIVE 04/23/2018

SWOG Authorized Document

B) We will assess treatment differences at the remaining time points individually. That is, we will also assess the week 3, 5, 7, 9, 11, 13, 25, and 37 scores using a linear regression analysis adjusting for stratification factors and the baseline score.

C) We will examine the influence of treatment on the MDASCI total score (all 21 items) at the follow-up assessment times.

D) We will assess whether individual symptom items within the MDASI are prognostic for time to progression. The MDASI core item scale (13 total items), the MDASI Severity scale (core plus lung symptoms, 15 total items), and the MDASI total score (all 21 items) will also be examined for their potential to predict time to progression. First, we will examine whether each of the 21 individual items or the 3 index scores predicts progression in bivariate analyses, adjusted for treatment arm. Candidate items will be further explored in multivariable analyses adjusting for stratification and a limited number of additional covariates (e.g., age). Given that this objective is exploratory, candidate factors that are prognostic will rely on p<.05 and will not adjust for multiple comparisons.

Initial examinations will be based on baseline PRO measures. However, we will also consider the potential prognostic value of the difference between the baseline and Week 5 measures, since deterioration as reflected in the PRO measures may not have begun until after treatment initiation. A landmark approach will be used to avoid endogeneity between the predictor (PRO assessment) and outcome (progression).

E) We will develop a statistical model that identifies a PRO-based symptom scale that is optimally prognostic for survival outcomes. The first step will be identifying the individual items prognostic for progression at p<.05 as indicated above, with each variable considered on a continuous scale. We will use best subset selection, which determines the "best" model based on the global Chi-squared statistic. *(54)* The best 2, 3, 4, and 5 variable models will be identified. For final model determination, we will use the mean of the q variables and create an indicator variable, split at the median. We will then identify which of the q-variable models maximizes the hazard ratio in a Cox regression, adjusted for treatment and the stratification variables. We anticipate the model building process will incorporate resampled K-fold cross-validation to adjust for adaptive multiple model selection. This modeling approach will be repeated for overall survival.

F) We will examine three individual Interference items from the MDASI-LC that address physical function/functional status [General activity; Work (including work around the house); Walking]) to see if this PRO domain is prognostic for progression. Although we do not have a physical function scale as part of the **S1400I** PROs, the individual MDASI-LC items relevant to this domain can provide an exploratory examination of the value of early decline in physical function status for later clinical outcomes.

G) An exploratory analysis will compare the three treatment-related toxicities (fatigue, pruritis, and diarrhea) by treatment arm at each of the scheduled assessment times using the PRO-CTCAE item scores.

H) Additional exploratory analyses will describe differences in EQ-5D Index scores by treatment arm, including the examination of Index scores by arm

CLOSED EFFECTIVE 04/28/2018

SWOG Authorized Document

at years 1, 2, and 3 to allow characterization of long term survivorship status for the different treatment groups.

DSMC approval will be required in any instance where early reporting of PRO results is sought.

VALIDATION

Analysis of Preliminary Validation Data for the Unvalidated NSCLC-SAQ Items: Phase II Trials

Undimensionality. (55): An exploratory factor analysis (EFA) will be done to identify the number of factors present in the NSCLC-SAQ. Support for a single factor measure will be examined with respect to commonly used criteria (e.g., scree test, the percent variance accounted for by the first factor, etc.); a confirmatory factor analysis (CFA) will fit a single factor model to the data to see fit statistics (e.g., CFI, RMSEA, etc.).

Internal Consistency Reliability: Coefficient alpha will be calculated for the either a single set of 7 NSCLC-SAQ items or for the number of scores based on the number of factors suggested by the EFA and CFA. Alphas ≥ 0.70 are of interest. *(56)*

Validity (57):
Construct Validity. Known groups comparisons will be made for mean scores by a set of variables where the levels of each variable can be hypothesized to generate different mean scores. The SWOG data will examine differences in patient-reported Zubrod performance status *(58,59,60)* (0-1, vs. 2) with respect to each item of the NSCLC-SAQ as well as the total score(s) if supported by the analyses above. Patients with performance status=2 would be expected to report worse symptom status on the NSCLC-SAQ that patients categorized as 0-1. We will also examine the comparisons using the patient-reported performance status item.

Criterion Validity. Given support for a total score, we expect the correlation between the MDASI-LC and the NSCLC-SAQ to be at least 0.35 (medium effect size for a correlation and 0.50 (large effect size for a correlation. *(61)* Given that the MDASI-LC is an established measure of lung cancer symptoms, it becomes the criterion measure. Reliability of each of the two measures being correlated is also a factor in their correlation.

Responsiveness: The effect size for the change in NSCLC-SAQ total scores between randomization and week 13 will be examined; effect sizes between 1/3 and ½ of a standard deviation will be of interest. *(62)* In addition, using the single item patient-reported global rating of change (GRC) in symptom severity status, we will examine the mean scores for patients who report different levels of change (i.e., those who report much better, much worse, better, worse, nearly the same); we will also combine the much better and the better categories for a three-level measure of perceived change in symptom status. These latter analyses will allow preliminary identification of minimally important differences. *(63,64,65)*

Data analysis performed by: Joseph Unger, PhD

Disclosure of conflict of interest:
There are no conflicts of interest reported

Other considerations (including plan for financial support, grant submission, etc.): Additional support will be sought from the Lung-MAP PRO

CLOSED EFFECTIVE 04/23/2018

SWOG Authorized Document

collaborating organizations to conduct analyses of the validation data and to transfer any data from the SWOG Statistics and Data Management Center to any Lung-MAP PRO Working Group collaborators.

References for 18.2

1 Iyer S, Roughley A, Rider A, Taylor-Stokes G. The symptom burden of non-small cell lung cancer in the USA: a real-world cross-sectional study. Support Care Cancer 22, 181-187, 2014. [PMID: 24026981]

2 Gridelli C, Ardizzoni A, Le Chevalier T, Manegold C, Perrone F, Thatcher N, van Zandwijk N, Di Maio M, Martelli O, De Marinis F. Treatment of advanced non-small-cell lung cancer patients with ECOG performance status 2: results of an European Experts Panel. Annals of Oncology 15, 419-426, 2004. [PMID: 14998843]

3 Barney BJ, Wang XS, Lu C, et al. Prognostic value of patient-reported symptom interference in patients with late-stage lung cancer. Qual Life Res 22, 2143-2150, 2013. [PMID: 23371797]

4 Gotay CC, Kawamoto CT, Bottomley A, et al The prognostic significance of patient-reported outcomes in cancer clinical trials. J Clin Oncol 26(8), 1355-1363, 2008. [PMID: 18227528]

5 Eton DT, Fairclough DL, Cella D, Yount SE, Bonomi P, Johnson DH. Early change in patient-reported health during lung cancer chemotherapy predicts clinical outcomes beyond those predicted by baseline report: Results from Eastern Cooperative Oncology Group Study 5592. J Clin Oncol 21(8), 1536-1543, 2003. [PMID: 12697878]

6 Wang XS, Shi Q, Lu C, et al. Prognostic value of symptom burden for overall survival in patients receiving chemotherapy for advanced non-small cell lung cancer. Cancer 116(1), 137-145, 2010. [PMID: 19852033]

7 Kummar S, Williams PM, Lih CJ, et al. Application of molecular profiling in clinical trials for advanced metastatic cancers. J Natl Cancer Inst 107(4), djv003 [pages 001-006], 2015. [PMID: 25663694]

8 Barney BJ, Wang XS, Lu C, et al. Prognostic value of patient-reported symptom interference in patients with late-stage lung cancer. Qual Life Res 22, 2143-2150, 2013. [PMID: 23371797]

9 Shi Q, Trask PC, Wang XS, et al. Does recall period have an effect on cancer patients' ratings of the severity of multiple symptoms? Journal of Pain and Symptom Management 40(2), 191-199, 2010. [PMID: 20579835]

10 Cleeland CS, Mendoza TR, Wang XS, et al. Assessing symptom distress in cancer patients. Cancer 89(7), 1634-1646, 2000. [PMID: 11013380]

11 Mendoza TR, Wang XS, Lu C, et al. Measuring the symptom burden of lung cancer: the validity and utility of the Lung Cancer Module of the M. D. Anderson Symptom Inventory. The Oncologist 16, 217-227, 2011. [PMID: 21285393]

12 Cleeland CS. The M. D. Anderson Symptom Inventory. User Guide Version 1. Houston, TX: The University of Texas M. D. Anderson Cancer Center, 2014. https://www.mdanderson.org/education-and-research/departments-programs-and-labs/departments-and-divisions/symptom-research/symptom-assessment-tools/MDASI_userguide.pdf.

CLOSED EFFECTIVE 04/23/2018

SWOG Authorized Document

13 Mendoza TR, Wang XS, Lu C, et al. Measuring the symptom burden of lung cancer: the validity and utility of the Lung Cancer Module of the M. D. Anderson Symptom Inventory. The Oncologist 16, 217-227, 2011. [PMID: 21285393]

14 Barney BJ, Wang XS, Lu C, et al. Prognostic value of patient-reported symptom interference in patients with late-stage lung cancer. Qual Life Res 22, 2143-2150, 2013. [PMID: 23371797]

15 Wang XS, Shi Q, Lu C, et al. Prognostic value of symptom burden for overall survival in patients receiving chemotherapy for advanced non-small cell lung cancer. Cancer 116(1), 137-145, 2010. [PMID: 19852033]

16 Cleeland CS, Mendoza TR, Wang XS, et al. Assessing symptom distress in cancer patients. Cancer 89(7), 1634-1646, 2000. [PMID: 11013380]

17 Mendoza TR, Wang XS, Lu C, et al. Measuring the symptom burden of lung cancer: the validity and utility of the Lung Cancer Module of the M. D. Anderson Symptom Inventory. The Oncologist 16, 217-227, 2011. [PMID: 21285393]

18 Cleeland CS. The M. D. Anderson Symptom Inventory. User Guide Version 1. Houston, TX: The University of Texas M. D. Anderson Cancer Center, 2014. https://www.mdanderson.org/education-and-research/departments-programs-and-labs/departments-and-divisions/symptom-research/symptom-assessment-tools/MDASI_userguide.pdf

19 Brundage M, Blazeby J, Revicki D, et al. Patient-reported outcomes in randomized clinical trials: development of ISOQOL reporting standards. Qual Life Res 22(6), 1161-1175, 2013. [PMID: 22987144]

20 Calvert M, Blazeby J, Altman DG, et al, Reporting of patient-reported outcomes in randomized clinical trials: The CONSORT PRO Extension. JAMA 309(8), 814-822, 2013. [PMID: 23443445]

21 Reeve BB, Wyrwich KW, Wu AW, et al. ISOQOL recommends minimum standards for patient-reported outcome measures used in patient-centered outcomes and comparative effectiveness research. Quality of Life Research 22(8), 1889-1905, 2013. [PMID: 23288613]

22 Critical Path Institute, & Patient-Reported Outcome (PRO) Consortium Non-Small Cell Lung Cancer (NSCLC) Working Group. Preliminary development of the Non-Small Cell Lung Cancer - Symptom Assessment Questionnaire (NSCLC-SAQ). Tucson, AZ: Critical Path Institute, 2015. http://c-path.org/programs/pro/

23 Critical Path Institute, & Patient-Reported Outcome (PRO) Consortium Non-Small Cell Lung Cancer (NSCLC) Working Group. Preliminary development of the Non-Small Cell Lung Cancer - Symptom Assessment Questionnaire (NSCLC-SAQ). Tucson, AZ: Critical Path Institute, 2015. http://c-path.org/programs/pro/

24 Cella DF, Bonomi AE, Lloyd SR, et al. Reliability and validity of the Functional Assessment of Cancer Therapy -- Lung (FACT-L) quality of life instrument. Lung Cancer 12(3), 199-220, 1995. [PMID: 7655830]

25 Basen-Engquist K, Bodurka-Bovers D, Fitzgerald MA, et al. Reliability and validity of the Functional Assessment of Cancer Therapy-Ovarian. Journal of Clinical Oncology 19(6), 1809-1817, 2001. [PMID: 11251013]

26 Yost KJ, Cella D, Chawla A, et al. Minimally important differences were estimated for the functional Assessment of Cancer Therapy--Colorectal (FACT-C) instrument using a combination of distribution- and anchor-based approaches. Journal of Clinical Epidemiology 58, 1241-1251, 2005. [PMID: 16291468]

CLOSED EFFECTIVE 04/23/2018

SWOG Authorized Document

27 Yost KJ, Eton DT, Garcia SF, et al. Minimally important differences were estimated for six Patient-Reported Outcomes Measurement Information System-Cancer scales in advanced-stage cancer patients. Journal of Clinical Epidemiology 64(5), 507-516, 2011. [PMID: 21447427]

28 Zubrod CG, Schneiderman M, Frei E, et al. Appraisal of methods for the study of chemotherapy in man: Comparative therapeutic trial of nitrogen mustard and thiophosphoramide. J Chronic Diseases 11, 7-33, 1960. http://www.jameslindlibrary.org/zubrod-cg-schneiderman-m-frei-e-brindley-c-gold-gl-shnider-b-oviedo-r-gorman-j-jones-r-jonsson-u-colsky-j-chalmers-t-ferguson-b-dederick-m-holland-j-selawry-o-regelson-w-lasagna-l-ow/

29 Yost KJ, Eton DT, Garcia SF, et al. Minimally important differences were estimated for six Patient-Reported Outcomes Measurement Information System-Cancer scales in advanced-stage cancer patients. Journal of Clinical Epidemiology 64(5), 507-516, 2011. [PMID: 21447427]

30 Yost KJ, Eton DT. Combining distribution- and anchor-based approaches to determine minimally important differences: the FACIT experience. Evaluation & The Health Professions 28(2), 172-191, 2005. [PMID: 15851772]

31 Yost KJ, Sorensen MV, Hahn EA, et al, Cella D. Using multiple anchor- and distribution-based estimates to evaluate clinically meaningful change on the Functional Assessment of Cancer Therapy-Biologic Response Modifiers (FACT-BRM) instrument. Value in Health 8(2), 117-127, 2005. [PMID: 15804320]

32 Dueck AC, Mendoza TR, Mitchell SA, et al. Validity and reliability of the US National Cancer Institute's Patient-Reported outcomes Version of the Common Terminology Criteria for Adverse Events (PRO-CTCAE). JAMA Oncology 1(8):1051-1059, 2015. [PMID: 26270597]

33 Gangadhar TC, Vonderheide RH. Mitigating the toxic effects of anticancer immunotherapy. Nature Reviews Clinical Oncology 11(2), 91-99, 2014. [PMID: 24445516]

34 Cheung K, Oemar M, Oppe M, Rabin R. On behalf of the EuroQoL Group EQ-5D. User Guide. Basic information on how to use EQ-5D, 2009. http://www.euroqol.org/fileadmin/user_upload/Documenten/PDF/Folders_Flyers/EQ-5D-Y_User_Guide_v1.0_2014.pdf

35 Luo N, Johnson JA, Shaw JW, et al. Self-reported health status of the general adult U. S. population as assessed by the EQ-5D and Health Utilities Index. Medical Care 43(11), 1078-1086, 2005. [PMID: 16224300]

36 Pickard AS, De Leon MC, Kohlmann T, et al. Psychometric comparison of the standard EQ-5D to a 5 level version in cancer patients. Medical Care, 45(3), 259-263, 2007. [PMID: 17304084]

37 Pickard AS, Neary MP, Cella D. Estimation of minimally important differences in EQ-5D utility and VAS scores in cancer. Health and Quality of Life Outcomes 5(70), 2007. [PMID: 18154669]

38 Eton DT, Fairclough DL, Cella D, Yount SE, Bonomi P, Johnson DH. Early change in patient-reported health during lung cancer chemotherapy predicts clinical outcomes beyond those predicted by baseline report: Results from Eastern Cooperative Oncology Group Study 5592. J Clin Oncol 21(8), 1536-1543, 2003. [PMID: 12697878]

39 Cleeland CS. The M. D. Anderson Symptom Inventory. User Guide Version 1. Houston, TX: The University of Texas M. D. Anderson Cancer Center, 2014. https://www.mdanderson.org/education-and-research/departments-programs-and-labs/departments-and-divisions/symptom-research/symptom-assessment-tools/MDASI_userguide.pdf.

CLOSED EFFECTIVE 04/23/2018

SWOG Authorized Document

40 Basch E, Reeve BB, Mitchell SA, et al. Development of the National Cancer Institute's Patient-Reported Outcomes version of the Common Terminiology Criteria for Adverse Events (PRO-CTCAE). J Natl Cancer Inst 106(9), Published online September 29, 2014. [PMID: 25265940]

41 Cheung K, Oemar M, Oppe M, Rabin R. On behalf of the EuroQoL Group EQ-5D. User Guide. Basic information on how to use EQ-5D, 2009. http://www.euroqol.org/fileadmin/user_upload/Documenten/PDF/Folders_Flyers/EQ-5D-Y_User_Guide_v1.0_2014.pdf

42 Shi Q, Trask PC, Wang XS, et al. Does recall period have an effect on cancer patients' ratings of the severity of multiple symptoms? Journal of Pain and Symptom Management 40(2), 191-199, 2010. [PMID: 20579835]

43 Cheung K, Oemar M, Oppe M, Rabin R. On behalf of the EuroQoL Group EQ-5D. User Guide. Basic information on how to use EQ-5D, 2009. http://www.euroqol.org/fileadmin/user_upload/Documenten/PDF/Folders_Flyers/EQ-5D-Y_User_Guide_v1.0_2014.pdf

44 Mendoza TR, Wang XS, Lu C, et al. Measuring the symptom burden of lung cancer: the validity and utility of the Lung Cancer Module of the M. D. Anderson Symptom Inventory. The Oncologist 16, 217-227, 2011. [PMID: 21285393]

45 Cleeland CS, Mendoza TR, Wang XS, et al. Assessing symptom distress in cancer patients. Cancer 89(7), 1634-1646, 2000. [PMID: 11013380]

46 Mendoza TR, Wang XS, Lu C, et al. Measuring the symptom burden of lung cancer: the validity and utility of the Lung Cancer Module of the M. D. Anderson Symptom Inventory. The Oncologist 16, 217-227, 2011. [PMID: 21285393]

47 Cleeland CS. The M. D. Anderson Symptom Inventory. User Guide Version 1. Houston, TX: The University of Texas M. D. Anderson Cancer Center, 2014. https://www.mdanderson.org/education-and-research/departments-programs-and-labs/departments-and-divisions/symptom-research/symptom-assessment-tools/MDASI_userguide.pdf

48 Mendoza TR, Wang XS, Lu C, et al. Measuring the symptom burden of lung cancer: the validity and utility of the Lung Cancer Module of the M. D. Anderson Symptom Inventory. The Oncologist 16, 217-227, 2011. [PMID: 21285393]

49 Cohen J. Statistical power analysis for the behavioral sciences (Second Edition ed.). Hillsdale, New Jersey: Lawrence Erlbaum Associates, Publishers, 1988.

50 Sloan JA, Dueck AC. Issues for statisticians in conducting analyses and translating results for quality of life end points in clinical trials. J Biopharm Stat 14(1), 73-96, 2004. [PMID: 15027501]

51 Little RJA. A class of pattern-mixture models for normal incomplete data. Biometrika 81, 471-483, 1994. http://biomet.oxfordjournals.org/content/81/3/471.abstract.

52 Pauler DK, McCoy S, Moinpour C. Pattern mixture models for longitudinal quality of life studies in advanced stage disease. Stat Med 22(5), 795-809, 2003. [PMID: 12587106]

53 Fairclough DL. Design and analysis of quality of life studies in clinical trials, Second Edition (2nd ed.). Boca Raton, FL: Chapman & Hall/CRC, Taylor & Francis Group, LLC, 2010. http://file.zums.ac.ir/ebook/173-Design%20and%20Analysis%20of%20Quality%20of%20Life%20Studies%20in%20Clinical%20Trials,%20Second%20Edition-Diane%20L.%20Fa.pdf.

CLOSED EFFECTIVE 04/23/2018

SWOG Authorized Document

54 Furnival GM, Wilson RW. Regressions by leaps and bounds. Technometrics 16, 499-511, 1974. http://www.stat.washington.edu/adobra/classes/536/Files/week3/leapsandbounds.pdf.

55 Nunnally J.C. Psychometric theory (Second ed.). New York, NY: McGraw-Hill Publishing Co, 1978.

56 Nunnally J.C. Psychometric theory (Second ed.). New York, NY: McGraw-Hill Publishing Co, 1978.

57 Nunnally J.C. Psychometric theory (Second ed.). New York, NY: McGraw-Hill Publishing Co, 1978.

58 Cella DF, Bonomi AE, Lloyd SR, et al. Reliability and validity of the Functional Assessment of Cancer Therapy -- Lung (FACT-L) quality of life instrument. Lung Cancer 12(3), 199-220, 1995. [PMID: 7655830]

59 Basen-Engquist K, Bodurka-Bovers D, Fitzgerald MA, et al. Reliability and validity of the Functional Assessment of Cancer Therapy-Ovarian. Journal of Clinical Oncology 19(6), 1809-1817, 2001. [PMID: 11251013]

60 Yost KJ, Cella D, Chawla A, et al. Minimally important differences were estimated for the functional Assessment of Cancer Therapy--Colorectal (FACT-C) instrument using a combination of distribution- and anchor-based approaches. Journal of Clinical Epidemiology 58, 1241-1251, 2005. [PMID: 16291468]

61 Cohen J. Statistical power analysis for the behavioral sciences (Second Edition ed.). Hillsdale, New Jersey: Lawrence Erlbaum Associates, Publishers, 1988.

62 Cella D, Eton DT, Fairclough DL, et al. What is a clinically meaningful change on the Functional Assessment of Cancer Therapy -Lung (FACT-L) Questionnaire? Results from Eastern Cooperative Oncology Group (ECOG) Study 5592. Journal of Clinical Epidemiology 55, 285-295, 2002. [PMID: 11864800]

63 Yost KJ, Eton DT, Garcia SF, et al. Minimally important differences were estimated for six Patient-Reported Outcomes Measurement Information System-Cancer scales in advanced-stage cancer patients. Journal of Clinical Epidemiology 64(5), 507-516, 2011. [PMID: 21447427]

64 Yost KJ, Eton DT. Combining distribution- and anchor-based approaches to determine minimally important differences: the FACIT experience. Evaluation & The Health Professions 28(2), 172-191, 2005. [PMID: 15851772]

65 Yost KJ, Sorensen MV, Hahn EA, et al, Cella D. Using multiple anchor- and distribution-based estimates to evaluate clinically meaningful change on the Functional Assessment of Cancer Therapy-Biologic Response Modifiers (FACT-BRM) instrument. Value in Health 8(2), 117-127, 2005. [PMID: 15804320]

CLOSED EFFECTIVE 04/23/2018

SWOG Authorized Document
