# Supplementary material for: Nivolumab Plus Ipilimumab vs Nivolumab for Previously Treated Patients With Stage IV Squamous Cell Lung Cancer: The Lung-MAP S1400I Phase 3 Randomized Clinical Trial
Source: JAMA Oncol. 2021 Jul 15;7(9):1368–77. doi: 10.1001/jamaoncol.2021.2209 (PMC8283667; doi:10.1001/jamaoncol.2021.2209)
Supplement: Supplement 2. — S1400 Master Protocol [file jamaoncol-e212209-s002.pdf]

PRIVILEGED COMMUNICATION
FOR INVESTIGATIONAL USE ONLY

Distribution IRB Review Only 5/28/14
Activated June 16, 2014

SWOG

A BIOMARKER-DRIVEN MASTER PROTOCOL FOR PREVIOUSLYTREATED SQUAMOUS CELL LUNG CANCER (LUNG-MAP)

NCT #02154490

This is a potential FDA registration study. There will be additional centralized and on-site monitoring conducted in addition to routine audits. Sites must also maintain a study specific Trial Master File for this study (https://swog.org/Visitors/QA/Index.asp).

STUDY CHAIRS:

Vassiliki A. Papadimitrakopoulou, M.D.
(Medical Oncology)
M.D. Anderson Cancer Center
1515 Holcombe Blvd., Unit 432
Houston, TX 77030-4009
Phone: 713/792-6363
FAX: 713/792-1220
E-mail: vpapadim@mdanderson.org

Fred R. Hirsch, M.D., Ph.D. (Translational Medicine)
University of Colorado Cancer Center
P.O. Box 6511, MS 8117
Aurora, CO 80045
Phone: 303/724-3858
E-mail: fred.hirsch@ucdenver.edu

Philip C. Mack, Ph.D. (Translational Medicine)
UC Davis Cancer Center
Division of Hematology/Oncology
4501 X Street
Sacramento, CA 95817
Phone: 916/734-8022
FAX: 916/734-2361
E-mail: pcmack@ucdavis.edu

Roy S. Herbst, M.D., Ph.D.
(Steering Committee/Targeted Agent Selection Committee Co-Chair)
Yale Cancer Center
Section of Medical Oncology
333 Cedar Street, WWW221
New Haven, CT 06520-8028
Phone: 203/785-6879
FAX: 203/785-4116
E-mail: roy.herbst@yale.edu

Lawrence H. Schwartz, M.D. (Imaging)
Columbia University Medical College
180 Fort Washington Avenue, HP-3-320
New York, NY 10032
Phone: 212/305-8994
FAX: 212/305-4835
E-mail: lhs2120@cumc.columbia.edu.

* Erlotinib is not a current study agent effective 11/25/2014.

** Docetaxel is not a current study agent effective12/18/2015.

CURRENT STUDY AGENTS:

Available from Commercial Sources
Docetaxel ** (Taxotere®)(RP56976) (NSC 628503)
Erlotinib * (OSI-774, Tarceva®) (NSC 718781)

Available from Pharmaceutical Collaborators:
SWOG Held INDs
AZD4547 (NSC 765338) (IND-119672)
GDC-0032 (Taselisib) (NSC 778795) (IND-119672)
Ipilimumab (NSC 732442) (IND-119672)
MEDI4736 (NSC 778709) (IND-119672)
Nivolumab (NSC 748726) (IND-119672)
Palbociclib (PD-0332991) (NSC 772256) (IND-119672)
Talazoparib (BMN 673) (NSC 771561) (IND-119672)
Tremelimumab (NSC 744483) (IND-119672)

STUDY CHAIRS (contd.):

David R. Gandara, M.D.
(SWOG, Lung-MAP Co-Chair)
UC Davis Cancer Center
Division of Hematology/Oncology
4501 X Street
Sacramento, CA 95817
Phone: 916/734-3772
FAX: 916/734-7946
E-mail: drgandara@ucdavis.edu

BIOSTATISTICIANS:

Mary Redman, Ph.D.
Shannon McDonough, M.S.
Katherine Griffin, M.S.
Jieling Miao, M.S.
James Moon, M.S.
SWOG Statistical Center
1100 Fairview Ave N, M3-C102
P.O. Box 19024
Seattle, WA 98109-1024
Phone: 206/667-4623
FAX: 206/667-4408
E-mail: mredman@fredhutch.org
E-mail: smcdonou@fredhutch.org
E-mail: kgriffin@fredhutch.org
E-mail: jmiao@fredhutch.org
E-mail: jmoon@fredhutch.org

ALLIANCE STUDY CHAIR:

Everett Vokes, M.D.
University of Chicago Medical Center
E-mail: evokes@medicine.bsd.uchicago.edu

ECOG-ACRIN STUDY CHAIR:

Suresh Ramalingam, M.D.
Emory University
E-mail: suresh.ramalingam@emoryhealthcare.org

CCTG STUDY CHAIR:

Natasha Leighl, BSc, MMSc, M.D.
Univ. Health Network-Princess Margaret Hospital
E-mail: Natasha.leighl@uhn.ca

NRG STUDY CHAIR:

Jeff Bradley, M.D.
Washington University School of Medicine
E-mail: jbradley@radonc.wustl.edu

SWOG STUDY CHAIR:

Karen Kelly, M.D.
UC Davis Cancer Center
E-mail: karkelly@ucdavis.edu

PARTICIPANTS

ALLIANCE/Alliance for Clinical Trials in Oncology
ECOG-ACRIN/ECOG-ACRIN Cancer Research Group
CCTG/Canada CancerTrials Group
NRG/NRG Oncology
SWOG/SWOG

TABLE OF CONTENTS

Page

TITLE **1**
PARTICIPANTS **2**
TABLE OF CONTENTS **3**
CANCER TRIALS SUPPORT UNIT (CTSU) ADDRESS AND CONTACT INFORMATION **5**
SCHEMA **6**
1.0 OBJECTIVES **7**
1.1 Primary Objective of the Master Protocol (S1400) 7
1.2 Design #1: Phase II/III Design 7
1.3 Design #2: Phase II followed by Phase III (Sequential Phase II to Phase III) 8
1.4 Common Objectives for all sub-studies designs 9
2.0 BACKGROUND **9**
2.1 General Background 9
2.2 Choice of Disease Setting 10
2.3 Study Design 11
2.4 SP44 anti-C-MET Immunohistochemistry (IHC) Assay 12
2.5 Inclusion of Women and Minorities 12
3.0 DRUG INFORMATION **13**
4.0 STAGING CRITERIA **13**
5.0 ELIGIBILITY CRITERIA **14**
5.1 Screening/Pre-Screening Registration 15
5.2 Sub-study Registration 16
6.0 STRATIFICATION FACTORS **19**
7.0 TREATMENT PLAN **19**
7.1 Treatment 19
7.2 Radiology Review 19
7.3 Discontinuation of Treatment 20
7.4 Follow Up Period 20
8.0 TOXICITIES TO BE MONITORED AND DOSE MODIFICATIONS **20**
9.0 STUDY CALENDAR **20**
10.0 CRITERIA FOR EVALUATION AND ENDPOINT ANALYSIS **20**
10.1 Measurability of Lesions 20
10.2 Objective Status at Each Disease Evaluation 22
10.3 Best Response 24
10.4 Performance Status 25
10.5 Time to Death 25
10.6 Investigator-Assessed Progression-Free Survival 25
10.7 Progression-Free Survival by Central Review 25
10.8 Duration of Response (DoR) 25
11.0 STATISTICAL CONSIDERATIONS **26**
11.1 Design #1: Phase II/III Design 26
11.2 Design #2: Phase II followed by Phase III (Sequential Phase II to Phase III) 30
11.3 Blinded Independent Centralized Review (BICR) of PFS and/or ORR 36
11.4 Accrual Information 36
11.5 Screening Success Rate Evaluation 37
11.6 Treatment Arm Randomization Acceptance Rate Evaluation 37
11.7 Data and Safety Monitoring 37
12.0 DISCIPLINE REVIEW **38**
13.0 REGISTRATION GUIDELINES **38**
13.1 Registration Timing 38
13.2 Investigator/Site Registration 38
13.3 OPEN Registration Requirements 41
13.4 Registration Procedures 42
13.5 Exceptions to SWOG registration policies will not be permitted. 43
14.0 DATA SUBMISSION SCHEDULE **43**

14.1 Data Submission Requirements for Screening/Pre-Screening Registration 43
14.2 Master Forms 43
14.3 Data Submission Procedures 43
14.4 Data Submission Overview and Timepoints for Screening/Pre-Screening Registration 45
14.5 Data Submission FOR PATIENTS WHO WILL NOT BE REGISTERED TO ANY OF THE SUB-STUDIES 45
14.6 Data Submission FOR PATIENTS WHO HAVE PROGRESSED ON A SUB-STUDY AND WISH TO REGISTER TO A NEW SUB-STUDY 46
15.0 SPECIAL INSTRUCTIONS 46
15.1 SWOG Specimen Tracking System (STS) 46
15.2 Biomarker Profiling (Required) 47
15.3 Correlative Studies and Banking (Optional for Patients) 49
15.4 Radiology Review (Required for all Sub-studies) 50
15.5 S1400GEN Survey Ancillary Study (Optional for Patient) 51
16.0 ETHICAL AND REGULATORY CONSIDERATIONS 52
16.1 Adverse Event Reporting Requirements 54
17.0 BIBLIOGRAPHY 55
18.0 APPENDIX 57
18.1a Biomarker-Testing Algorithm and Clinical Assays 58
18.1b New York Heart Association Classification 64
18.1c Risk Based Monitoring Plan 65
18.1d Dose and Administration Schedules Overview 72
18.1e Specimen Flow Diagram 74
18.1f S1400GEN 75
18.2 List of Sub-study Attachments 84

CANCER TRIALS SUPPORT UNIT (CTSU) ADDRESS AND CONTACT INFORMATION

<table>
<tr><th>To submit site registration documents:</th><th>For patient enrollments:</th><th>Submit study data directly to the Lead Cooperative Group unless otherwise specified in the protocol:</th></tr>
<tr><td>Regulatory documentation must be submitted to the CTSU via the Regulatory Submission Portal:

Regulatory Submission Portal (Sign in at www.ctsu.org, and select the Regulatory Submission sub-tab under the Regulatory tab.)

Institutions with patients waiting that are unable to use the Portal should alert the CTSU Regulatory Office immediately at 1-866-651-2878 to receive further instruction and support.

Contact the CTSU Regulatory Help Desk at 1-866-651-2878 for regulatory assistance.</td><td>Please refer to the patient enrollment section of the protocol for instructions on using the Oncology Patient Enrollment Network (OPEN) which can be accessed at https://www.ctsu.org/OPEN_SYSTEM/ or https://OPEN.ctsu.org.

Contact the CTSU Help Desk with any OPEN-related questions at ctsucontact@westat.com.</td><td>Data collection for this study will be done exclusively through Medidata Rave. Please see the data submission section of the protocol for further instructions.

<u>Other Tools and Reports</u>:
Institutions participating through the CTSU continue to have access to other tools and reports available to the SWOG Workbench. Access this by using your active CTEP-IAM USER ID and password at the following url: https://crawb.crab.org/TXWB/ctsulogon.aspx.</td></tr>
<tr><td colspan="3">The most current version of the study protocol and all supporting documents must be downloaded from the protocol-specific Web page of the CTSU Member Web site located at https://www.ctsu.org. Access to the CTSU members' website is managed through the Cancer Therapy and Evaluation Program - Identity and Access Management (CTEP-IAM) registration system and requires user log on with CTEP-IAM username and password. Permission to view and download this protocol and its supporting documents is restricted and is based on person and site roster assignment housed in the CTSU RSS.

CTSU site should follow procedures outlined in the protocol for site registration. Patient Enrollment Adverse Event Reporting, Data Submission (including ancillary studies), and Drug Procurement.</td></tr>
<tr><td colspan="3"><u>For patient eligibility </u>questions contact the SWOG Data Operations Center by phone or email:

206-652-2267
<u>S1400</u>question@crab.org

<u>For treatment or toxicity related </u>questions contact S1400Medicalquery@swog.org.</td></tr>
<tr><td colspan="3"><u>For questions unrelated to patient eligibility, treatment, or data submission</u> contact the CTSU Help Desk by phone or e-mail:

CTSU General Information Line:
888-823-5923
ctsucontact@westat.com

All calls and correspondence will be triaged to the appropriate CTSU representative.</td></tr>
<tr><td colspan="3">The CTSU Web site is located at https://www.ctsu.org</td></tr>
</table>

SCHEMA

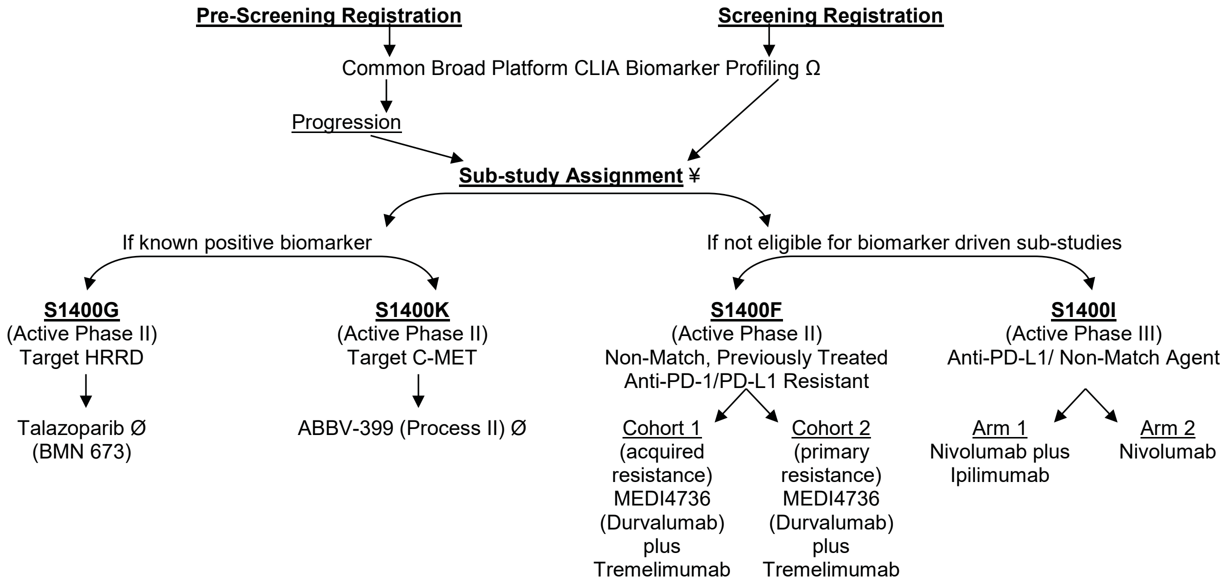

Ω Archival formalin-fixed paraffin-embedded (FFPE) tumor, fresh core needle biopsy if needed.

¥ Sub-study assignment will be determined by the SWOG Statistics and Data Management Center (SDMC). Sub-study assignment will be determined based on randomization for patients eligible for multiple sub-studies (see Section 11.0 for details).
Patients registered to the screening/pre-screening component, but not randomized to a treatment arm of any of the sub-studies will be followed until death or 3 years after screening/pre-screening registration, whichever comes first.

Ø Upon progression (as defined in Section 10.2d in **S1400**), patients may be eligible for another sub-study. The new sub-study assignment will be determined by the SWOG SDMC. (see Section 14.6).

1.0 OBJECTIVES

1.1 Primary Objective of the Master Protocol (**S1400**)

The overarching goal for this protocol is to establish a National Clinical Trials Network (NCTN) mechanism for genomically screening large but homogeneous cancer populations and subsequently assigning and accruing simultaneously to a multi-sub-study "Master Protocol". Biomarker-driven sub-studies in this protocol will evaluate a targeted therapy (TT) or targeted therapy combination (TTC) based on designated therapeutic biomarker-drug combinations, with the ultimate goal being approval of new targeted therapies in this setting. In addition, the protocol includes at least one "non-match" sub-study which will include all screened patients not eligible for any of the biomarker-driven sub-studies. This sub-study will evaluate a non-match therapy (NMT), also with the goal of approval. For all sub-studies the investigational therapy arm will be referred to as the investigational therapy arm. We hypothesize that this Master Protocol mechanism will yield definable and measurable efficiencies in terms of improving genomic screening of cancer patients for clinical trial entry, and improved time lines for drug-biomarker testing allowing for inclusion of the maximum numbers of otherwise eligible patients in comparison with currently employed "single screen-single trial" approaches.

This Protocol employs a "hybrid" master protocol in which the design for a given sub-study is chosen from a limited number of clinical trials designs based on the expected biomarker prevalence and background data. The design options for the sub-studies include a Phase II/III design and a Single Arm Phase II followed by a Randomized Phase III (under specified conditions). The objectives for each design are included in this section (Section 1.0) and the statistical considerations for each design are included in **S1400** Section 11.0.

1.2 Design #1: Phase II/III Design

a. Sub-study Primary Objectives

1. Phase II Component

The primary objective within the Phase II component is to evaluate if there is sufficient evidence to continue to the Phase III component of the sub-study by comparing investigator-assessed progression-free survival (IA-PFS) between investigational therapy versus standard therapy (SoC) in patients with advanced stage refractory squamous cell carcinoma (SCCA) of the lung.

2. Phase III Component

The co-primary objectives of the Phase III component are:

i. To determine if there is both a statistically and clinically-meaningful difference in IA-PFS among advanced stage refractory SCCA of the lung randomized to receive investigational therapy versus SoC.

ii. To compare overall survival (OS) in patients with advanced stage refractory SCCA of the lung randomized to investigational therapy versus SoC.

b. Sub-study Secondary Objectives

1. Phase II Component

i. To compare response rates (confirmed and unconfirmed, complete and partial responses) among patients randomized to receive investigational therapy versus SoC.

ii. To evaluate the frequency and severity of toxicities associated with investigational therapy versus SoC.

iii. To evaluate the duration of response (DoR) among patients who achieve a CR or PR by RECIST 1.1

2. Phase III Component

i. To compare the response rates (confirmed and unconfirmed, complete and partial) among patients randomized to receive investigational therapy versus SoC.

ii. To evaluate the frequency and severity of toxicities associated with investigational therapy versus SoC.

c. Treatment Arm Randomization Acceptance Rate Objective

To evaluate the treatment arm randomization acceptance rate (TARAR) within each treatment arm of each sub-study defined as the percentage of patients randomized to a treatment arm that receive any protocol treatment.

1.3 Design #2: Phase II followed by Phase III (Sequential Phase II to Phase III)

a. Sub-study Primary Objectives

1. Phase II Component

The primary objective within the Phase II component is to evaluate the objective response rate (confirmed and unconfirmed, complete and partial).

2. Phase III Component

The co-primary objectives of the Phase III component are:

i. To determine if there is both a statistically and clinically-meaningful difference in IA-PFS among advanced stage refractory SCCA of the lung randomized to receive investigational therapy versus SoC.

ii. To compare overall survival (OS) in patients with advanced stage refractory SCCA of the lung randomized to investigational therapy versus SoC.

b. Sub-study Secondary Objectives

1. Phase II component

i. To evaluate PFS and OS with investigational therapy

ii. To evaluate the duration of response (DoR) among patients who achieve a CR or PR (confirmed and unconfirmed) by RECIST 1.1

c. To evaluate the frequency and severity of toxicities associated with investigational therapy.

1. Phase III Component

i. To compare the response rates (confirmed and unconfirmed, complete and partial) among patients randomized to receive investigational therapy versus SoC.

ii. To evaluate the frequency and severity of toxicities associated with investigational therapy versus SoC.

1.4 Common Objectives for all sub-studies designs

a. Screening Success Rate Objective

To evaluate the screen success rate defined as the percentage of screened patients that register for a therapeutic sub-study. Screen success rates will be evaluated for the total screened population and by the subset of patients screened following progression on previous therapy or pre-screened on current therapy.

b. Translational Medicine Objectives

1. To identify additional predictive tumor/blood biomarkers that may modify response or define resistance to the TT/TTC beyond the chosen biomarker for biomarker-driven sub-studies.

2. To evaluate potentially predictive biomarkers for NMT in the non-match studies.

3. To identify potential resistance biomarkers at disease progression.

4. To establish a tissue/ blood repository from patients with refractory squamous cell carcinoma (SCCA) of the lung.

2.0 BACKGROUND

2.1 <u>General Background</u>

Despite several impressive therapeutic advances in recent years, cancer remains the second-leading cause of death in the United States and effective new therapies are still desperately needed. Developing a potential therapy from the initial discovery stage through clinical testing and regulatory review is a complicated, expensive, and often inefficient process that can take up to 15 years. Included among the many challenges of drug development are the difficulties in recruiting cancer patients to clinical trials, the extensive bureaucratic processes required to initiate any clinical trial, and lengthy regulatory review. Modernizing this process with innovative approaches and new clinical trial designs is of high importance.

Although the advent of targeted therapies holds great promise for improved efficacy, this also means that many patients may need to be screened before enough patients harboring the necessary genomic alteration are available for the trial to be completed. In order to address the expense and inefficiencies of the current drug development process and in hopes to identify promising compounds in selected populations, stakeholders began to develop and test innovative approaches for clinical trials, including development of algorithms for clinical trial designs facilitating early drug/biomarker co-development. *(1)* Phase II screening trials such as BATTLE (**B**iomarker-integrated **A**pproaches of **T**argeted

Therapy for **L**ung Cancer **E**limination) and **I**-SPY 2 TRIAL (**I**nvestigation of **S**erial Studies to **P**redict **Your** **T**herapeutic **R**esponse with **I**maging **A**nd mo**L**ecular Analysis **2**) are early trial examples using biomarker screening and facilitating addition of new drugs and biomarkers into the protocol on a "rolling" basis. *(2, 3)* Here we propose a Master Protocol for genomic screening and multi-sub-study testing of drug/biomarker combinations in a Phase II/III setting compatible with subsequent FDA approval. Genomic screening of a large patient resource provided by sites participating in the NCI National Clinical Trials Network (NCTN) is designed to identify a series of molecular targets/biomarkers for which there will be a new drug match, leading to appropriate sub-study assignment and drug treatment. Each molecular target included in **S1400** will be represented by a biomarker for which there will be an analytically validated diagnostic assay. This approach provides a basis for large-scale screening/clinical registration trial with the ability to screen patients, either through genomic analysis or immunohistochemistry based assays, with homogeneous eligibility criteria and direct them to a sub-study of the trial based on the results of screening diagnostic tests.

There are multiple advantages to a multi-sub-study Master Protocol, compared to the traditional alternative of multiple 2-arm registration studies. First, for drugs that have already shown promise in a biomarker-selected patient population, grouping these under a single trial, with a control (standard-of-care) arm will dramatically reduce the overall screen failure rate. For example, with the biomarker prevalence rates anticipated for molecular targets in this protocol, and a minimum of 4 drug/biomarker trial sub-studies open at any one time, a "hit rate" of well over 60% can be achieved, making the screening process worthwhile for patients and physicians alike. Second, there are processes and operational efficiencies gained by having a single master protocol, which could be amended as needed as drugs enter and exit the study. A master protocol will also provide consistency, as every drug for the disease would be tested in the identical manner. Sponsors may be encouraged to include their drug in a master registration trial if there were assurances that if pre-specified efficacy and safety criteria were met during the Phase II/III process, the drug and accompanying companion diagnostic would be approved. Finally, by improving the overall efficiency of drug development in a specific disease setting, this trial offers the advantage of bringing safe and effective drugs to patients sooner than they might otherwise be available.

2.2 Choice of Disease Setting

We have chosen to use lung cancer as the prototype disease for this Master Protocol. Lung cancer is the most common cancer today, with an estimated 228,190 new cases expected in the United States in 2013 alone. *(4)* Non-small cell lung cancer (NSCLC) is characterized by multiple and often independent mutations and potential therapeutic targets, and screening is rapidly becoming a part of treatment in some subtypes of lung cancer, such as adenocarcinoma, making it an excellent case study for a multi-sub-study biomarker driven Master Protocol.

Genomic characterization studies of lung adenocarcinomas conducted over the last 10 years have revealed a number of therapeutically targetable alterations, many of which have received therapeutic validation. This has led to a transformation of our standard of care approach for these patients that are now routinely molecularly genotyped in an attempt to pair the identified mutation with the appropriate targeted therapy. Similar progress in genotyping is being made for squamous cell carcinoma of the lung (SCCA) primarily led by The Cancer Genome Atlas (TCGA) but also other sequencing efforts. *(5, 6)* A number of the alterations that have been identified are targetable but relatively uncommon, while others are more common but not as readily actionable. *(7)* Lung SCCA remains an "orphan" group where substantial developments in targeted therapeutics have yet to be seen and all the targeted therapies so far approved in NSCLC, are largely ineffective. In hopes of identifying promising compounds in this setting, the selection of subgroups (genotype or phenotype-driven) is a strategy which is increasingly adopted in clinical trials. Thus, we are proposing a multi-sub-study Master Protocol combining Phase II and Phase

III, where interim endpoints are met, in a disease setting representing a major unmet need in cancer therapy: SCCA. This approach will provide the basis for FDA approval of new drugs with matching companion diagnostics. If this multi-sub-study Master Protocol strategy is successful, this type of biomarker-driven umbrella protocol could be used for registration trials in other settings. The FDA recently approved the PD-1 checkpoint inhibitor nivolumab for the treatment of lung SCCA based on a Phase 3 trial of nivolumab versus docetaxel based on overall survival benefit and checkpoint inhibitor therapy is a major component of the Master protocol strategy. *(8)*

2.3 Study Design

This is a prospective, multi-sub-study master registration protocol, in which patients with advanced stage lung SCCA (previously treated), are assigned to a biomarker-driven targeted therapy (TT) phase II study with the primary objective being overall response rate. If the objective response rate observed in the phase II study is judged sufficient the study will proceed into a Phase III trial in which patients will be randomized to biomarker-driven targeted therapy or SoC. Each sub-study is defined by a genotypically defined alteration (biomarker) in the tumor and a drug (or drug combination) that targets it. Each sub-study will function autonomously and will open and close independently of the other sub-studies. The candidate drugs must have demonstrated biologic activity against the target associated with a proposed predictive biomarker(s). Drug combinations in the experimental arm will be allowed in appropriate settings and where appropriate the control arm may consist of FDA approved targeted therapy such as erlotinib. Archival formalin-fixed paraffin-embedded (FFPE) tumor will be screened by a broad analytically validated testing next generation sequencing (NGS) platform centrally to establish eligibility within 10-14 days. This platform will be supplemented by individual immune-histochemical (IHC) protein assays performed in a CLIA setting as necessitated by the specific experimental agent used. Core needle biopsies for fresh tumor tissue may be necessary when archival tumor tissue is not available or on a drug-biomarker-specific basis.

Patients with tumors that do not match one of the currently active drug-biomarker combinations ("non-match" patients) will be assigned to a specific "non-match" sub-study and randomized to either treatment with a designated "non-match" drug (anticipated to initially consist of an agent targeting PD-L1) or SoC. This is a really important sub-study as it will serve as the anchor for **S1400** and will provide access to an exciting new therapy approach for patients whose tumors do not harbor the genomic abnormalities targeted (or express the biomarker) in the other sub-studies, while ensuring that the screen failure rate will be low for the trial overall.

As described in preliminary discussions with the FDA, it is anticipated that the broad Foundation Medicine NGS testing platform may itself be suitable for registration purposes, while other methods may be required for companion diagnostic status for a given experimental agent/biomarker protocol sub-study. *(9)* In addition, it is expected that patients will be "pre-screened" in advance during first-line therapy in order to reduce delay in **S1400** enrollment at the time of failure on first line therapy. Patients will be separately consented for screening and treatment randomization.

The Foundation Medicine NGS Assay to be used for identification of genomic alterations in the subject tumor in the master protocol for previously treated squamous cell lung cancer **S1400** will be based on Foundation Medicine's T5 assay for cancer gene profiling. For this study, pre-specified classification rules will be applied to data generated by the T5 assay to place patients into biomarker defined groups. The laboratory process used for the Foundation Medicine NGS Assay is a robust, multi-step protocol to generate targeted deep sequence data from adaptor-ligated sequencing libraries of DNA extracted from FFPE tissue. The workflow is executed in a Clinical Laboratory Improvement Amendments (CLIA)-certified and College of American Pathologists (CAP) accredited laboratory and begins with sample receipt and accessioning into a laboratory information management system (LIMS), incorporates the generation of targeted sequencing data on an NGS

platform, and culminates with a customized analysis pipeline capable of detecting genomic alterations with high accuracy. Multiple steps of the process are run on automated liquid handlers to ensure consistency/efficiency. The assay includes process-matched controls in each run, and defined quality control (QC) metrics are used throughout the process to monitor sample and assay performance.

2.4 SP44 anti-C-MET Immunohistochemistry (IHC) Assay

c-Met overexpression has been reported in NSCLC, with varying prevalence depending on method of evaluation and scoring. For example, c-Met positivity has been reported in 24.4%-29% of patients with squamous cell lung cancer. ([10],[11]) Though c-Met protein overexpression is common in NSCLC, strategies thus far to select patients based on overexpression for treatment with anti-c-Met monoclonal antibodies and small molecule inhibitors of c-Met have failed to show clinical benefit in large randomized studies. ABBV-399 (Process II) is a based on a different mechanism of action, i.e. to use the c-Met expression to deliver the toxin, and thus has a potential to improve the quality of life and outcome of patients with squamous cell lung cancer, based on its tolerability and preliminary efficacy.

2.5 Inclusion of Women and Minorities

This study was designed to include women and minorities, but was not designed to measure differences of intervention effects. Accrual is expected to reach between 500 and 800 patients per year. The minimum and maximum anticipated annual accrual in the ethnicity/race and sex categories are shown in the tables below.

Assuming 500 patients accrued in a year:

**Ethnic Category**	**Females**	**Males**	**Total**
Hispanic or Latino	6	9	15
Not Hispanic or Latino	210	275	485
**Total Ethnic**	216	284	500
**Racial Category**			
American Indian or Alaskan Native	1	0	1
Asian	4	7	11
Black or African American	16	26	42
Native Hawaiian or other Pacific Islander	2	3	5
White	193	248	441
**Racial Category: Total of all Subjects**	216	284	500

Assuming 800 patients accrued in a year:

Ethnic Category	Females	Males	Total
Hispanic or Latino	10	14	24
Not Hispanic or Latino	336	440	776
**Total Ethnic**	346	454	800
**Racial Category**			
American Indian or Alaskan Native	2	0	2
Asian	6	11	17
Black or African American	26	42	68
Native Hawaiian or other Pacific Islander	2	3	5
White	310	398	708
**Racial Category: Total of all Subjects**	346	454	800

3.0 DRUG INFORMATION

Please see Section 18.2 for sub-study specific drug information and sub-study specific treatment plans.

Investigator's Brochures

For information regarding Investigator's Brochures, please refer to SWOG Policy 15. For **S1400**, the investigational drugs are being provided under an IND held by SWOG. For INDs filed by SWOG, the protocol serves as the Investigator Brochure for the performance of the protocol. In such instances submission of the protocol to the IRB should suffice for providing the IRB with information about the drug. However, in cases where the IRB insists on having the official Investigator Brochure from the company, requests may be submitted to the CTSU website by completing the CTSU Request for Clinical Brochure.

4.0 STAGING CRITERIA

Patients must have Stage IV disease as outlined below (AJCC Cancer Staging Manual, 7th Edition, 2010):

Stage IV	Any T	Any N	M1a
	Any T	Any N	M1b

Primary Tumor (T)

TX Primary tumor cannot be assessed, or tumor proven by the presence of malignant cells in sputum or bronchial washings but not visualized by imaging or bronchoscopy
T0 No evidence of primary tumor
Tis Carcinoma in situ
T1 Tumor 3 cm or less in greatest dimension, surrounded by lung or visceral pleura, without bronchoscopic evidence of invasion more proximal than the lobar bronchus (i.e., not in the main bronchus)*
T1a Tumor 2 cm or less in greatest dimension
T1b Tumor more than 2 cm but 3 cm or less in greatest dimension
T2 Tumor more than 3 cm but 7 cm or less or tumor with any of the following features (T2 tumors with these features are classified T2a if 5 cm or less); Involves main bronchus, 2 cm or more distal to the carina; Invades visceral pleura (PL1 or PL2); Associated with

atelectasis or obstructive pneumonitis that extends to the hilar region but does not involve the entire lung

T2a Tumor more than 3 cm but 5 cm or less in greatest dimension

T2b Tumor more than 5 cm but 7 cm or less in greatest dimension

T3 Tumor more than 7 cm or one that directly invades any of the following: parietal pleural (PL3) chest wall (including superior sulcus tumors), diaphragm, phrenic nerve, mediastinal pleura, parietal pericardium; or tumor in the main bronchus (less than 2 cm distal to the carina* but without involvement of the carina; or associated atelectasis or obstructive pneumonitis of the entire lung or separate tumor nodule(s) in the same lobe

T4 Tumor of any size that invades any of the following: mediastinum, heart, great vessels, trachea, recurrent laryngeal nerve, esophagus, vertebral body, carina, separate tumor nodule(s) in a different ipsilateral lobe

* The uncommon superficial spreading tumor of any size with its invasive component limited to the bronchial wall, which may extend proximally to the main bronchus, is also classified as T1a.

Regional Lymph Nodes (N)

NX Regional lymph nodes cannot be assessed

N0 No regional lymph node metastases

N1 Metastasis in ipsilateral peribronchial and/or ipsilateral hilar lymph nodes and intrapulmonary nodes, including involvement by direct extension

N2 Metastasis in ipsilateral mediastinal and/or subcarinal lymph node(s)

N3 Metastasis in contralateral mediastinal, contralateral hilar, ipsilateral or contralateral scalene, or supraclavicular lymph node(s) Distant Metastasis (M)

M0 No distant metastasis

M1 Distant metastasis

M1a Separate tumor nodule(s) in a contralateral lobe tumor with pleural nodules or malignant pleural (or pericardial) effusion **

M1b Distant metastasis

** Most pleural (and pericardial) effusions with lung cancer are due to tumor. In a few patients, however, multiple cytopathologic examinations of pleural (pericardial) fluid are negative for tumor, and the fluid is non-bloody and is not an exudate. Where these elements and clinical judgment dictate that the effusion is not related to the tumor, the effusion should be excluded as a staging element and the patient should be classified as M0.

5.0 ELIGIBILITY CRITERIA

Each of the criteria in the following sections must be met in order for a patient to be considered eligible for the corresponding registration. Section 5.1 must be met prior to screening/pre-screening registration, while Section 5.2 must be met prior to sub-study registration. Use the spaces provided to confirm a patient's eligibility. For each criterion requiring test results and dates, please record this information on the **S1400** Onstudy Form and submit via Medidata Rave® (see Section 14.0). Any potential eligibility issues should be addressed to the Data Operations Center in Seattle at **S1400**question@crab.org prior to registration.

In calculating days of tests and measurements, the day a test or measurement is done is considered Day 0. Therefore, if a test is done on a Monday, the Monday 4 weeks later would be considered Day 28. This allows for efficient patient scheduling without exceeding the guidelines. **If Day 7, 14, 16, 28 or 42 falls on a weekend or holiday, the limit may be extended to the next working day.**

5.1 Screening/Pre-Screening Registration

a. Patients must have pathologically proven squamous cell carcinoma (SCCA) cancer of the lung confirmed by tumor biopsy and/or fine-needle aspiration. Disease must be Stage IV SCCA as defined in Section 4.0, or recurrent. The primary diagnosis of SCCA should be established using the current WHO/IASLC-classification of Thoracic Malignancies. *(12)* The diagnosis is based on H&E stained slides with or without specific defined IHC characteristic (p40/p63 positive, TTF1 negative) if required for diagnosis. Mixed histologies are not allowed.

b. Patients must either be eligible to be screened at progression on prior treatment or to be pre-screened prior to progression on current treatment. Patients will either consent to the Screening consent or the Pre-Screening consent, not both. These criteria are:

1. Screening at progression on prior treatment:
To be eligible for screening at progression, patients must have received at least one line of systemic therapy for any stage of disease (Stages I-IV) and must have progressed during or following their most recent line of therapy. For patients whose prior systemic therapy was for Stage I-III disease only (i.e. patient has not received any treatment for Stage IV or recurrent disease), the prior systemic therapy must have been a platinum-based chemotherapy regimen and disease progression on the platinum-based chemotherapy must have occurred within one year from the last date that patient received that therapy. For patients whose prior therapy was for Stage IV or recurrent disease, the patient must have received at least one line of a platinum-based chemotherapy regimen or checkpoint inhibitor therapy (e.g. Nivolumab or Pembrolizumab).

2. Pre-Screening prior to progression on current treatment:
To be eligible for pre-screening, current treatment must be for Stage IV or recurrent disease and patient must have received at least one dose of the current regimen. Patients must have previously received or currently be receiving a platinum-based chemotherapy regimen or checkpoint inhibitor therapy (e.g. Nivolumab or Pembrolizumab). Patients on first-line treatment are eligible upon receiving Cycle 1, Day 1 infusion. **Note: Patients will not receive their sub-study assignment until they progress and the S1400 Notice of Progression is submitted.**

c. Patients must have adequate tumor tissue available, defined as ≥ 20% tumor cells and ≥ 0.2 mm^3 tumor volume.
- The local interpreting pathologist must review the specimen.
- The pathologist must sign the **S1400** Local Pathology Review Form confirming tissue adequacy prior to screening/pre-screening registration.

Patients must agree to have this tissue submitted to Foundation Medicine for common broad platform CLIA biomarker profiling and c-Met IHC (see Section 15.1). If archival tumor material is exhausted, then a new fresh tumor biopsy that is formalin-fixed and paraffin-embedded (FFPE) must be obtained. A tumor block or FFPE slides 4-5 microns thick must be submitted. Bone biopsies are not allowed. If FFPE slides are to be submitted, at least 12 unstained slides plus an H&E stained slide, or 13 unstained slides must be submitted. However, it is strongly recommended that 20 FFPE slides be submitted. Note: Previous next-generation DNA sequencing (NGS) will be repeated if done outside this study for sub-study assignment.

Patients must agree to have any tissue that remains after NGS testing retained for the use of the Translational Medicine (TM) studies (if such TM studies are defined) within any sub-study the patient is enrolled in.

d. Patients must not have a known EGFR mutation, or ALK fusion. EGFR/ALK testing is not required prior to registration and is included in the FMI testing for screening/prescreening.

e. Patients must have Zubrod performance status 0-1 (see Section 10.4) documented within 28 days prior to screening/pre-screening registration.

f. Patients must be ≥ 18 years of age.

g. Patients must also be offered participation in banking for future use of specimens as described in Section 15.0.

h. Patients must be willing to provide prior smoking history as required on the **S1400** Onstudy Form.

i. As a part of the OPEN registration process (see Section 13.4 for OPEN access instructions) the treating institution's identity is provided in order to ensure that the current (within 365 days) date of institutional review board approval for this study has been entered in the system.

j. Patients must be informed of the investigational nature of this study and must sign and give written informed consent in accordance with institutional and federal guidelines.

k. U.S. patients who can complete the survey and the interview by telephone or email in English must be offered participation in the **S1400GEN** Survey Ancillary Study (see Sections 15.5 and 18.1f). NOTE: Patients enrolled to **S1400** prior to Revision #12 are not eligible for the **S1400GEN** Survey Ancillary Study. Study physicians will provide participants with a hard copy of the survey (at the time of informed consent) to improve tracking and comprehension during the interview.

5.2 Sub-study Registration

For patients screened at progression on prior treatment, a sub-study assignment from the SWOG Statistical Center should be received within 16 days of tissue submission.

For patients pre-screened prior to progression on current therapy, submission of the **S1400** Notice of Progression Form is required to receive a sub-study assignment. The sub-study assignment should be received from the SWOG Statistical Center within 1 day of submission of the **S1400** Notice of Progression (provided at least 16 days have passed since tissue submission). Patients must then register to the assigned sub-study in order to receive their treatment assignment.

After progression on an **S1400** sub-study, the **S1400** Request for New Sub-Study Assignment Form may be submitted to receive a new sub-study assignment (see Section 14.6). The new sub-study assignment should be received from the SWOG Statistical Center within 1 day of submission of the **S1400** Request for New Sub-Study Assignment Form. Patients must then register to the assigned sub-study to receive their treatment assignment.

Patient must meet the eligibility criteria listed in Section 5.0 of the assigned sub-study. The common eligibility criteria are included here. For ease of reference, these common eligibility criteria have also been incorporated into Section 5.0 of each of the sub-studies.

If patient does not meet the additional criteria listed in Section 5.0 of the assigned sub-study, submit the **S1400** Request for Sub-Study Reassignment Form. (See Section 18.1a for biomarker reporting.) Any potential eligibility issues should be addressed to the Data Operations Center in Seattle at S1400question@crab.org prior to registration.

a. Patients whose biomarker profiling results indicate the presence of an EGFR mutation or EML4/ALK fusion are not eligible. Due to existence of approved therapies the biomarker exclusion rules are as follows:

Gene	Alteration type	Ineligible Alteration
EGFR	Substitution	L858R, T790M, A289V, G719A, S768I, G719C, R108K, G598V, R222C, L62R, L861Q, P596L, V774M
	Indel	non-frame shifting insertions or deletions between amino acids 740 and 780, in exons 19 and 20, transcript NM_005228
	Fusion	None
	Amplification	None
ALK	Substitution	None
	Indel	None
	Fusion	EML4-ALK, CLIP4-ALK, CLTC-ALK, KIF5B-ALK, NPM1-ALK, RANB2-ALK, STRN-ALK, TFG-ALK
	Amplification	None

b. Patients must have progressed (in the opinion of the treating investigator) following the most recent line of therapy.

c. Patients must not have received any prior systemic therapy (systemic chemotherapy, immunotherapy or investigational drug) within 21 days prior to sub-study registration. Patients must have recovered (≤ Grade 1) from any side effects of prior therapy. Patients must not have received any radiation therapy within 14 days prior to sub-study registration. (See 5.2e for criteria regarding therapy for CNS metastases).

d. Patients must have measurable disease (see Section 10.1) documented by CT or MRI. The CT from a combined PET/CT may be used to document only non-measurable disease unless it is of diagnostic quality as defined in Section 10.1c. Measurable disease must be assessed within 28 days prior to sub-study registration. Pleural effusions, ascites and laboratory parameters are not acceptable as the only evidence of disease. Non-measurable disease must be assessed within 42 days prior to sub-study registration. All disease must be assessed and documented on the Baseline Tumor Assessment Form. Patients whose only measurable disease is within a previous radiation therapy port must demonstrate clearly progressive disease (in the opinion of the treating investigator) prior to registration. See Sections 15.0 and 18.1c for guidelines and submission instructions for required central radiology review.

e. Patients must have a CT or MRI scan of the brain to evaluate for CNS disease within 42 days prior to sub-study registration. Patient must not have leptomeningeal disease, spinal cord compression or brain metastases unless: (1) metastases have been locally treated and have remained clinically controlled and

asymptomatic for at least 14 days following treatment and prior to registration, AND (2) patient has no residual neurological dysfunction and has been off corticosteroids for at least 24 hours prior to sub-study registration.

f. Patient must have fully recovered from the effects of surgery at least 14 days prior to sub-study registration.

g. Patients must not be planning to receive any concurrent chemotherapy, immunotherapy, biologic or hormonal therapy for cancer treatment. Concurrent use of hormones for non-cancer-related conditions (e.g., insulin for diabetes and hormone replacement therapy) is acceptable.

h. Patients must have an ANC ≥ 1,500 mcL, platelet count ≥ 100,000 mcL and hemoglobin ≥ 9 g/dL obtained within 28 days prior to sub-study registration.

i. Patients must have adequate hepatic function as defined by serum bilirubin ≤ Institutional Upper Limit of Normal (IULN) and either ALT <u>or</u> AST ≤ 2 x IULN within 28 days prior to sub-study registration (if both ALT and AST are done, both must be ≤ 2 IULN). For patients with liver metastases, bilirubin and either ALT <u>or</u> AST must be ≤ 5 x IULN (if both ALT and AST are done, both must be ≤ 5 x IULN).

j. Patients must have a serum creatinine ≤ the IULN OR measured or calculated creatinine clearance ≥ 50 mL/min using the following Cockroft-Gault Formula:

$$\text{Calculated Creatinine Clearance} = \frac{(140 - \text{age}) \times (\text{actual body weight in kg})^{\dagger}}{72 \times \text{serum creatinine}^{*}}$$

Multiply this number by 0.85 if the patient is a female. These tests must have been performed within 28 days prior to sub-study registration.

† The kilogram weight is the patient weight with an upper limit of 140% of the IBW.
* Actual lab serum creatinine value with a minimum of 0.8 mg/dL.

k. Patients must have Zubrod performance status 0-1 (see Section 10.4) documented within 28 days prior to sub-study registration.

l. Patients must not have any Grade III/IV cardiac disease as defined by the New York Heart Association Criteria (i.e., patients with cardiac disease resulting in marked limitation of physical activity or resulting in inability to carry on any physical activity without discomfort), unstable angina pectoris, and myocardial infarction within 6 months, or serious uncontrolled cardiac arrhythmia (see Section 18.1b).

m. Patients must not have documented evidence of acute hepatitis or have an active or uncontrolled infection.

n. Patients with a known history of HIV seropositivity:

1. Must have undetectable viral load using standard HIV assays in clinical practice.
2. Must have CD4 count ≥ 400/mcL.
3. Must not require prophylaxis for any opportunistic infections (i.e., fungal, mAC, or PCP prophylaxis).
4. Must not be newly diagnosed within 12 months prior to sub-study registration.

o. Prestudy history and physical exam must be obtained within 28 days prior to sub-study registration.

p. No other prior malignancy is allowed except for the following: adequately treated basal cell or squamous cell skin cancer, *in situ* cervical cancer, adequately treated Stage I or II cancer from which the patient is currently in complete remission, or any other cancer from which the patient has been disease free for five years.

q. Patients must not be pregnant or nursing. Women/men of reproductive potential must have agreed to use an effective contraceptive method. A woman is considered to be of "reproductive potential" if she has had menses at any time in the preceding 12 consecutive months. In addition to routine contraceptive methods, "effective contraception" also includes heterosexual celibacy and surgery intended to prevent pregnancy (or with a side-effect of pregnancy prevention) defined as a hysterectomy, bilateral oophorectomy or bilateral tubal ligation. However, if at any point a previously celibate patient chooses to become heterosexually active during the time period for use of contraceptive measures outlined in the protocol, he/she is responsible for beginning contraceptive measures.

r. As a part of the OPEN registration process (see Section 13.4 for OPEN access instructions) the treating institution's identity is provided in order to ensure that the current (within 365 days) date of institutional review board approval for this study has been entered in the system.

s. Patients with impaired decision-making capacity are eligible as long as their neurological or psychological condition does not preclude their safe participation in the study (e.g., tracking pill consumption and reporting adverse events to the investigator).

t. Patients must be informed of the investigational nature of this study and must sign and give written informed consent in accordance with institutional and federal guidelines.

6.0 STRATIFICATION FACTORS

For sub-studies employing Design #1, within each sub-study, patients will be randomized between sub-study treatment arms using block randomization. See Section 6.0 in each sub-study for sub-study specific stratification factors.

7.0 TREATMENT PLAN

7.1 Treatment

See Section 18.2 for sub-study specific treatment information.

7.2 Radiology Review

a. To ensure the highest standards and consistency between different centers, all scans for disease assessment (baseline, interim and end of treatment scans) must be submitted to the National Cancer Institute’s National Clinical Trials Network (NCTN) Imaging and RT Quality Assurance Service Core (IROC) in Ohio for centralized review (see Section 15.0).

b. Centralized review will be performed by 3 radiology experts. The scans will be submitted to IROC. IROC will transmit the scans to the reviewers who will transmit the results to the SWOG Statistical Center.

c. Details of submission of scans to IROC for centralized review and on the central review process are listed in Section 15.0 and 18.1c.

7.3 Discontinuation of Treatment

All reasons for discontinuation of treatment must be documented on the Off Treatment Notice.

7.4 Follow Up Period

Patients who have been registered to the pre-screening/screening component, but not registered/randomization to a treatment arm of any of the sub-studies will be followed until sub-study registration or death, whichever comes first.

8.0 TOXICITIES TO BE MONITORED AND DOSE MODIFICATIONS

See Section 18.2 for sub-study specific toxicities to be monitored and dose modifications.

9.0 STUDY CALENDAR

See Section 18.2 for sub-study specific study calendars.

10.0 CRITERIA FOR EVALUATION AND ENDPOINT ANALYSIS

10.1 Measurability of Lesions

a. **Measurable disease**: Measurable disease is defined differently for lymph nodes compared with other disease and will be addressed in a separate section below.

1. Lesions that can be accurately measured in at least one dimension (longest diameter to be recorded) as ≥ 2.0 cm by chest x-ray, by ≥ 1.0 cm with CT or MRI scans, or ≥ 1.0 cm with calipers by clinical exam. All tumor measurements must be recorded in decimal fractions of centimeters.

   The defined measurability of lesions on CT scan is based on the assumption that CT slice thickness is 0.5 cm or less. If CT scans have slice thickness greater than 0.5 cm, the minimum size for a measurable lesion should be twice the slice thickness.

2. Malignant lymph nodes are to be considered pathologically enlarged and measurable if it measures ≥ 1.5 cm in **SHORT AXIS** (greatest diameter perpendicular to the long axis of the lymph node) when assessed by scan (CT scan slice recommended being no greater than 0.5 cm).

b. **Non-measurable disease**: All other lesions (or sites of disease), including small lesions (longest diameter < 1.0 cm or pathologic lymph nodes with ≥ 1.0 cm to < 1.5 cm short axis), are considered non-measurable disease. Note: Lymph nodes that have a short axis < 1.0 cm (10 mm) are considered non-pathological and should not be recorded or followed. Bone lesions, leptomeningeal disease, ascites, pleural/pericardial effusions, lymphangitis cutis/pulmonitis, inflammatory breast

disease, and abdominal masses (not followed by CT or MRI), are considered non-measurable as are previously radiated lesions that have not progressed.

c. Notes on measurability

1. For CT and MRIs, the same type of scanner should be used and the image acquisition protocol should be followed as closely as possible to prior scans. Body scans should be performed with breath-hold scanning techniques, if possible.

2. PET-CT: At present, the low dose or attenuation correction CT portion of a PET-CT is not always of optimal diagnostic CT quality for use with RECIST measurements. However, if the site can document that the CT performed as part of a PET-CT is of identical diagnostic quality to a diagnostic CT, then the CT portion of the PET-CT can be used for RECIST measurements and can be used interchangeably with conventional CT.

NOTE REGARDING DIAGNOSTIC QUALITY:

CT – Computed Tomograph Imaging

In order for a CT to be of diagnostic quality to be used in determining measurable disease, the slice thickness needs to match the protocol **Section 10.0**.

Recommended Scan mode:	Multi-detector and/or helical
Contrast Enhancement:	IV and oral contrast unless contraindicated
Slice Section thickness:	maximum 5mm, preferable 2.5mm or less
Slice Increment:	continuous or overlapping sections; no gaps
Imaging Region:	Thoracic inlet through adrenal glands (and appropriate scans if disease exists elsewhere)
Image Matrix size:	512 × 512 or better
Image Reconstruction / Filter :	Institutional standard

If a CT scan is performed with a slice thickness greater than 5 mm then lesions must be twice the slice thickness. If any PET/Spiral CT is used at baseline where the CT is of diagnostic quality, follow-up scans can be done by a spiral CT.

If any PET/Conventional CT is used at baseline where the CT is of diagnostic quality, follow-up scans can be done by conventional CT.

Institutions will have to submit radiology reports documenting that the CT used in PET/CT is of diagnostic quality. No other methods of assessments are interchangeable.

MRI – Magnetic Resonance Imaging

MRI can be performed using a 1.5 or 3.0 T field strength. If a MRI is performed instead of a CT, the MRI can be performed according to institutions clinical standard of care protocols with slice thickness of no more than 5mm (in transverse).

If an MRI scan is performed with a slice thickness greater than 5 mm, then lesions must be twice and above the slice thickness.

PET/CT – Positron Emission Tomography with FDG
When a FDG PET/CT is performed, the emission scans should be started in the range of 60 – 75 min after FDG injection, otherwise use your institution protocols. It is necessary for follow up scans that they are performed in an **identical way** to the baseline with the same PET/CT scanner and a variation in timing of no more than +/- 10 min. Preferably, schedule the patient for both baseline and follow-up scans at the same time of day (AM or PM) to improve reproducibility.

3. Ultrasound: Ultrasound is not useful in assessment of lesion size and should not be used as a method of measurement.

4. Cystic lesions that meet the criteria for radiographically defined simple cysts should not be considered as malignant lesions (neither measurable nor non-measurable) since they are, by definition simple cysts.

5. If a target lesion becomes very small some radiologists indicate that it is too small to measure. If the lesion is actually still present, a default measurement of 0.5 cm should be applied. If the radiologist believes the lesion has gone, a default measurement of 0.0cm should be recorded.

10.2 Objective Status at Each Disease Evaluation

Objective Status is to be recorded at each evaluation. All measurable lesions up to a maximum of 2 lesions per organ 5 lesions in total, representative of all involved organs, should be identified as *target* lesions at baseline. All other lesions (or sites of disease) including any measurable lesions over and above the 5 target lesions should be identified as *non-target* lesions. Measurements must be provided for target measurable lesions, while presence or absence must be noted for non-target measurable and non-measurable disease.

For studies that use disease progression as an endpoint, whole body scanning at specific intervals is necessary to determine that progression is NOT present outside of the “target” areas. Therefore, in these studies it is not acceptable to image only the “target” areas of the body in follow-up scans. For study-specific imaging requirements, see the Study Calendar in the appropriate sub-study specific section.

Disease evaluations for patients treated with MEDI4736 will also utilize an alternative system of immune-related response criteria (irRC) which is described in detail in **S1400A**.

a. **Complete Response (CR):** Complete disappearance of all target and non-target lesions (with the exception of lymph nodes mentioned below). No new lesions. No disease related symptoms. Any lymph nodes (whether target or non-target) must have reduction in short axis to < 1.0 cm. All disease must be assessed using the same technique as baseline.

b. **Partial Response (PR):** Applies only to patients with at least one measurable lesion. Greater than or equal to 30% decrease under baseline of the sum of appropriate diameters of all target measurable lesions. No unequivocal progression of non-measurable disease. No new lesions. All target measurable lesions must be assessed using the same techniques as baseline.

c. **Stable:** Does not qualify for CR, PR, Progression or Symptomatic Deterioration. All target measurable lesions must be assessed using the same techniques as baseline.

d. **Progression**: One or more of the following must occur: 20% increase in the sum of appropriate diameters of target measurable lesions over smallest sum observed (over baseline if no decrease during therapy) using the same techniques as baseline, as well as an absolute increase of at least 0.5 cm. Unequivocal progression of non-measurable disease in the opinion of the treating physician (an explanation must be provided). Appearance of any new lesion/site. Death due to disease without prior documentation of progression and without symptomatic deterioration (see Section 10.2e).

If a new lesion is equivocal, for example because of its small size, continued therapy and follow-up evaluation will clarify if it represents truly new disease. If repeat scans confirm there is definitely a new lesion, then progression should be declared using the date of the initial scan

Notes regarding new lesions: FDG-PET imaging can complement regular scans in identifying new lesions according to the following algorithm.

1. Negative FDG-PET at baseline, with a positive FDG-PET at follow-up is a sign of progression based on a new lesion.

2. No FDG-PET at baseline and a positive FDG-PET at follow-up corresponding to a potential new site of disease must have a confirmation by anatomical assessment (e.g. CT, MRI, x-ray) as new site of disease to be considered progressive disease. In such a case, the date of progressive disease will be the date of the initial abnormal FDG-PET.

e. **Symptomatic deterioration**: Global deterioration of health status requiring discontinuation of treatment without objective evidence of progression. Efforts should be made to obtain objective evidence of progression after discontinuation.

f. **Assessment inadequate, objective status unknown**. Progression or symptomatic deterioration has not been documented, and one or more target measurable lesions have not been assessed or inconsistent assessment methods were used.

g. Objective status notes:

1. Non-measurable and non-target measurable disease do not affect Objective Status in determination of CR (must be absent--a patient who otherwise has a CR, but who has non-measurable or non-target measurable disease present or not assessed, will be classified as having a PR). However, non-measurable and non-target lesions are included in determination of progression (if new sites of disease develop or if unequivocal progression occurs in the opinion of the treating physician).

2. An objective status of PR or stable cannot follow one of CR. Stable can follow PR only in the rare case that tumor increases too little to qualify as progression, but enough that a previously documented 30% decrease no longer holds.

3. In cases for which initial flare reaction is possible (hypercalcemia, increased bone pain, erythema of skin lesions), objective status is not progression unless either symptoms persist beyond 4 weeks or there is additional evidence of progression.

4. Lesions that appear to increase in size due to presence of necrotic tissue will not be considered to have progressed.

5. For bone disease documented on bone scan only, increased uptake does not constitute unequivocal progression. However, increase in the soft tissue component of a lesion as measured by CT or MRI would constitute progression.

6. Appearance of new pleural effusions does not constitute unequivocal progression unless cytologically proven of neoplastic origin, since some effusions are a toxicity related to therapy or other medical conditions. Increase in the size of an existing effusion does not constitute unequivocal progression, since the fluid status of the patient could alter the size of the effusion.

7. If CR determination depends on a lesion for which the status is unclear by the required tests, it is recommended the residual lesion be investigated with biopsy or fine needle aspirate.

10.3 Best Response

This is calculated from the sequence of objective statuses.

a. CR: Two or more objective statuses of CR a minimum of four weeks apart documented before progression or symptomatic deterioration.

b. PR: Two or more objective statuses of PR or better a minimum of four weeks apart documented before progression or symptomatic deterioration, but not qualifying as CR.

c. Unconfirmed CR: One objective status of CR documented before progression or symptomatic deterioration but not qualifying as CR or PR.

d. Unconfirmed PR: One objective status of PR documented before progression or symptomatic deterioration but not qualifying as CR, PR or unconfirmed CR.

e. Stable/no response: At least one objective status of stable/no response documented at least 6 weeks after registration and before progression or symptomatic deterioration, but not qualifying as anything else above.

f. Increasing disease: Objective status of progression within 12 weeks of registration, not qualifying as anything else above.

g. Symptomatic deterioration: Objective status of symptomatic deterioration within 12 weeks of registration, not qualifying as anything else above.

h. Inadequate assessment, response unknown: Progression or symptomatic deterioration greater than 12 weeks after registration and no other response category applies.

10.4 Performance Status

Patients will be graded according to the Zubrod Performance Status Scale.

POINT	DESCRIPTION
0	Fully active, able to carry on all pre-disease performance without restriction.
1	Restricted in physically strenuous activity but ambulatory and able to carry out work of a light or sedentary nature, e.g., light housework, office work.
2	Ambulatory and capable of self-care but unable to carry out anywork activities; up and about more than 50% of waking hours.
3	Capable of limited self-care, confined to bed or chair more than 50% of waking hours.
4	Completely disabled; cannot carry on any self-care; totally confined to bed or chair.

10.5 Time to Death

From date of sub-study registration (or date of screening/pre-screening registration if patient never enrolls in a sub-study) to date of death due to any cause. Patients last known to be alive are censored at date of last contact.

10.6 Investigator-Assessed Progression-Free Survival

From date of sub-study registration to date of first documentation of progression assessed by local review or symptomatic deterioration (as defined above), or death due to any cause. Patients last known to be alive without report of progression are censored at date of last disease assessment. For patients with a missing scan (or consecutive missing scans) whose subsequent scan determines progression, the expected date of the first missing scan (as defined by the disease assessment schedule) will be used as the date of progression.

10.7 Progression-Free Survival by Central Review

From date of sub-study registration to date of first documentation of progression assessed by central review or symptomatic deterioration (as defined above), or death due to any cause. Patients last known to be alive without report of progression are censored at date of last disease assessment. For patients with a missing scan (or consecutive missing scans) whose subsequent scan determines progression, the expected date of the first missing scan (as defined by the disease assessment schedule) will be used as the date of progression.

10.8 Duration of Response (DoR)

From date of first documentation of response (CR or PR) to date of first documentation of progression assessed by local review or symptomatic deterioration (as defined above), or death due to any cause among patients who achieve a response (CR or PR). Patients last known to be alive without report of progression are censored at date of last disease assessment. For patients with a missing scan (or consecutive missing scans) whose subsequent scan determines progression, the expected date of the first missing scan (as defined by the disease assessment schedule) will be used as the date of progression.

11.0 STATISTICAL CONSIDERATIONS

As stated above, the design of **S1400** is multi-sub-study design. The conduct of each sub-study proceeds independently of the other sub-studies with decisions at the interim and final analyses within one study having no impact on other sub-studies. This Protocol employs a “hybrid” master protocol in which the design for a given sub-study is chosen from a limited number of clinical trials designs based on the expected biomarker prevalence and background data. The design options for the sub-studies include Design #1 a Phase II/III design (see Section 11.1) and Design #2 a Single Arm Phase II followed by a Randomized Phase III (under specified conditions) (see Section 11.2). The objectives for each design are included in Section 1.0 and the statistical considerations for each design are included in this section.

Sub-study eligibility will be based on the results of biomarker analysis at screening. Patients with a single “positive” biomarker will be assigned to the associated sub-study. Patients with no “positive” biomarkers will be assigned to the non-match sub-study. Patients eligible for multiple sub-studies based on the results of the biomarker analysis will be randomized to sub-study assignment. The randomization ratio will be the ratio of the prevalence of the biomarkers allowing for a greater likelihood of assignment to the study with the lower prevalence (e.g. for two markers with 5% and 15% prevalence, the randomization ratio will be 3:1 in favor of the lower prevalence biomarker; for three biomarkers with 5%, 10% and 15% prevalence, the ratio will be 6:3:2, and so on). However, the ratio will be bounded such that no biomarker sub-study has more than a 4-fold chance of assignment. As sub-studies are closed or new sub-studies added the randomization ratio will be modified based on the sub-study biomarker prevalence for the actively accruing studies. Initial estimates of sub-study biomarker prevalence from sources outside this study will be used to determine the assumed prevalence of the sub-study biomarkers for determination of these sub-study assignment randomization ratios. If a specific sub-study biomarker prevalence estimate is substantially different from initial estimate, the prevalence estimates may be updated at most once during the conduct of the associated sub-study.

11.1 Design #1: Phase II/III Design

a. Primary Endpoint

<u>Phase II Component:</u> The primary objective within the Phase II component is to compare investigator-assessed PFS (IA-PFS) between investigational therapy versus standard of care (SoC) in patients with advanced stage refractory squamous cell carcinoma (SCCA) of the lung. The investigational therapy arm will be judged to have provided sufficient evidence to proceed to the Phase III component if the Phase II interim futility analysis at 55 progression events does not rule out the hypothesized improvement in median PFS (see Section 11.1c for details).

<u>Phase III Component:</u> The Phase III design has two co-primary objectives: an evaluation of IA-PFS and OS.

1. The primary PFS objective within the Phase III component of each sub-study is to rule out less than a 33% improvement (HR = 0.75) in median IA-PFS (or equivalently less than a one-month difference in medians) between investigational therapy and SoC in patients with advanced stage refractory squamous cell carcinoma (SCCA) of the lung.

2. The primary OS objective within the Phase III component of each sub-study is to compare overall survival (OS) in patients with advanced stage refractory SCCA of the lung randomized to receive investigational therapy versus SoC.

b. Secondary Endpoints

Phase II Component: Secondary objectives of the Phase II interim analysis will be to compare response rate (RR; confirmed and unconfirmed) and toxicity frequencies between the two arms within each sub-study.

Phase III Component: Secondary objectives of the Phase III component will be to compare response rate (RR; confirmed and unconfirmed), IA-PFS, censoring patients with symptomatic deterioration (SD) at the time of SD, and toxicity frequencies between the two arms within each sub-study.

c. Sample Size with Power Justification

The total sample size for each independently conducted Phase II/III sub-study is determined based on the biomarker prevalence, with all other design parameters the same across sub-studies. Details regarding the sample size for each sub-study is included in the Statistical Considerations in the sub-study protocol for each sub-study. (13)

Phase II Design: The Phase II interim futility analysis within each sub-study is to occur upon the observation of 55 investigator-assessed progression events. Sub-studies will follow either Plan A or Plan B for this interim analysis. Specification of plan for each sub-study is stated in the sub-study specific protocols (see Section 18.0).

Plan A: The size is based on a design that targets a 2-fold increase in median IA-PFS with investigational therapy (HR =0.5) with 90% power and a 10% 1-sided type I error rate. The futility boundary will be crossed if the alternative hypothesis of a 2-fold increase (HR = 0.5) is rejected at the 1-sided 10% level. This design has a 90% chance of stopping for futility under the null (1-α) and a 10% chance of falsely rejecting the alternative under a true 2-fold increase (1-Power). The approximate threshold associated with these parameters for continuation of the study past the Phase II interim futility analysis is the observation of at least a 41% improvement in median PFS (HR = 0.71).

Plan B: The size is based on a design that targets a 2.5-fold increase in median IA-PFS with investigational therapy (HR =0.4) with 95% power and a 4% 1-sided type I error rate. The futility boundary will be crossed if the alternative hypothesis of a 2.5-fold increase (HR=0.4) is rejected at the 1-sided 5% level. This design has a 96% chance of stopping for futility under the null and a 5% chance of falsely rejecting the alternative under a true 2.5-fold increase. The approximate threshold associated with these parameters for continuation of the study past the Phase II interim futility analysis is the observation of at least a 63% improvement in median PFS (HR=0.61).

Phase III Design: While a co-primary objective is the comparison of IA-PFS between the arms, the sample size for Phase III component of each sub-study is based on a design that targets a 50% improvement in median OS (HR=0.67) with 90% power and a 2.5% 1-sided type I error rate. This design requires 256 deaths.

The primary analysis of IA-PFS will be based on ruling out the null hypothesis of less than or equal to a 33% improvement in median PFS (an HR of 0.75 or greater). Assuming the median PFS in the control arm is 3 months, then, this evaluation is

ruling out a month or less difference between the arms. The expected number of IA-PFS events by the observation of 256 deaths. The final analysis for PFS will occur after completion of accrual, when either the 290 IA-PFS events have been observed or the maximum number of expected IA-PFS events has occurred if the sample size is less than 290 patients. With 290 PFS events and under the design assumptions, testing the IA-PFS null hypothesis of a HR equal to 0.75 at the 0.014 level has 90% power to detect HR of 0.5 (a 2-fold increase in median PFS). The approximate threshold for determining PFS is both clinically and statistically significant is a 75% improvement in median PFS (HR = 0.57). The 0.014 level for testing IA-PFS was chosen based on this desired level of clinical significance.

d. Analysis Plan Including Plans for Formal Interim Analysis

Primary analyses will be performed on an intent-to-treat basis. Stratified (using randomization stratification factors) log-rank test will be used to test the primary hypotheses related to PFS and OS comparing the two treatment arms at the levels specified below for the Phase II and Phase III components. A Cox proportional hazards (PH) model will be used to estimate the hazard ratios and associated confidence intervals. Analysis of response rates and toxicities will be performed using a chi-square or Fisher's exact test, as appropriate. The primary analysis of IA-PFS for the Phase III component will be done using a PH model score test (or in other words, a modified log-rank test at the PFS null hazard ratio of 0.75 [the 33% improvement in median PFS]). *(14)*

Secondary analysis of PFS and OS at the final analysis within each sub-study will be a weighted analysis (using Cox regression) standardizing the biomarker prevalence among patients with multiple biomarkers to the study population prevalence among all patients eligible as defined by the sub-study biomarker definition. The goal of this analysis is to estimate the treatment effects had all patients with the sub-study biomarker been assigned to the sub-study (as in a stand-alone trial).

<u>Phase II Interim Analyses</u>: The Phase II analysis is a standard interim analysis (a rolling interim analysis) and does not include a temporary halt of accrual for evaluation. This analysis will be performed upon the observation of 55 progression events.

Plan A: For this analysis, the alternative hypothesis of at least a 2-fold increase in median IA-PFS with investigational therapy versus SoC will be tested at the 1-sided 0.10 level, using a PH model score test evaluated at the alternative hypothesis of HR = 0.5. This design has a 90% chance of stopping for futility under the null hypothesis. The approximate threshold for continuation of the study (i.e. not rejecting the alternative hypothesis) is the observation of at least a 41% improvement in median IA-PFS (HR=0.71), under the design assumptions. If the alternative hypothesis is rejected, then the recommendation to the DSMB will be there is not sufficient evidence to continue to the Phase III portion of the study and the study should be permanently closed to accrual.

Plan B: For this analysis, the alternative hypothesis of at least a 2.5-fold increase in median IA-PFS with investigational therapy versus SoC will be tested at the 1-sided 0.05 level (1-Power = β), using a PH model score test evaluated at the alternative hypothesis of HR = 0.4. This design has a 96% chance of stopping for futility under the null hypothesis (1-α). The approximate threshold for continuation of the study (i.e. not rejecting the alternative hypothesis) is the observation of at least a 63% improvement in median PFS, under the design assumptions. If the alternative hypothesis is rejected, then the recommendation to the DSMB will be there is not sufficient evidence to continue to the Phase III portion of the study and the study should be permanently closed to accrual.

In addition to evaluating IA-PFS at this analysis (for both Plans A and B), the response rate (confirmed and unconfirmed) will be compared between the arms to determine if there is evidence to stop the study for early signs of efficacy. The outcome for this analysis will be the best objective response based on RECIST 1.1 which will be evaluated among patients registered at least 6 weeks before the interim analysis or patients with known value for objective response (i.e. CR/PR/SD/PD/death).

Response proportions will be compared using a 1-sided Fisher's exact test at the 0.001 level. If the null hypothesis is rejected at the 0.001 level, the recommendation will be for the DSMC to evaluate the entirety of the study data to determine if the study should be closed early for signs of efficacy. In other words, rejection of the null hypothesis for response is not an automatic stop for efficacy, additional efficacy outcomes such as IA-PFS should be supportive of early stopping as well.

Should neither the criteria for early stopping based on futility based on IA-PFS or early signs of efficacy based on response be met, the recommendation will be that study should continue into the Phase III portion of the study.

Phase III Interim Analyses: If the decision is to continue the study at the Phase II analysis, additional formal interim analyses are planned when 50% and 75% of the expected deaths have been observed with rules specified on the fixed-sample p-value scale.

Evidence suggesting early termination of the trial for futility/harm will be if the alternative hypothesis of at least 50% improvement in OS or the hypothesis of at least a 2-fold improvement in IA-PFS for the investigational therapy arm is rejected at the one-sided level 0.0025, using a PH model score test evaluated at the alternative hypothesis (e.g. H_a: HR=0.67 for OS and 0.5 for IA-PFS). *(15)*

Evidence suggesting early termination of the trial for superiority will be if the hypothesis of no difference in OS is rejected at the one-sided 0.0025 level using a log-rank test.

The associated approximate thresholds for ratio of medians (as measured by the inverse HR or exp (β) from a Cox model) and p-values (against the null) for OS are presented in Table 1.

Table 1: Interim Monitoring boundaries associated with monitoring rules

OS	Futility Bound		Efficacy Bound	
events	HR(SoC/Exp)	p-value	HR(SoC/Exp)	p-value
50%	0.95	0.614	1.64	0.0025
75%	1.04	0.391	1.50	0.0025
100%	1.28	0.024	1.28	0.024

Against the null hypothesis of HR = 1.

Final Analysis: If the decision is to continue the study to completion, the final analysis of IA-PFS will occur after completion of accrual, when either 290 progression events have been observed or the final analysis for OS is performed, whichever comes first.). The expected IA-PFS analysis time is between 6 and 12 months from study closure to accrual. The final analysis of OS will take place when at least 256 events have been observed. However, should events accumulate more slowly than anticipated, the maximum time to final analysis is when 18 months have passed since the completion of accrual. The analysis of IA-PFS will be a test against the null hypothesis of a 33% improvement in median IA-PFS (HR

= 0.75) at the 0.014 level (which has 90% power for a 2-fold increase in IA-PFS), with an approximate observed threshold of 75% improvement in median IA-PFS (HR=0.57) for significance (deemed both significant in statistical and clinical terms). In addition, it is considered that a clinically meaningful difference is the observation of at least a 2.25-month difference in median IA-PFS between the two arms for sub-studies evaluating single agent investigational therapy versus SoC. For sub-studies evaluating investigational therapy combinations, it is considered that a clinically meaningful difference is the observation of at least a 2.5-month difference in median IA-PFS between the two arms. This design has less than a 0.000001 chance of falsely rejecting the null hypothesis of an HR = 1, and therefore has a negligible effect on the study-wide false positive rate. Therefore, in this sense, this design has co-primary endpoints IA-PFS and OS. The final analysis of OS will be at level 0.024 to account for the effects of interim monitoring (using a modified Haybittle-Peto boundary). The adjusted level does not account for either the IA-PFS assessment for futility or the response assessment for efficacy at the Phase II interim analysis as the level of testing response is small (0.001) and the decision to stop for efficacy at this time point will also consider other clinical data.

The actual decision to terminate accrual early will be made by the SWOG Data and Safety Monitoring Committee (DSMC), and will consider IA-PFS, OS, response, and toxicities.

11.2 Design #2: Phase II followed by Phase III (Sequential Phase II to Phase III)

This design includes two options: an option for evalution of a targeted therapy in a biomarker-defined patient population and an evaluation of a non-match therapy in patients who have received and progressed on/following therapy with an immunotherapy agent.

a. Option for Biomarker-driven Subtudies

1. Primary Endpoint

<u>Single Arm Phase II Component:</u> The primary objective within the Phase II component is to evaluate the objective response rate (ORR) (confirmed and unconfirmed, complete and partial) in patients treated with investigational therapy with advanced stage refractory squamous cell carcinoma (SCCA) of the lung. The investigational therapy arm will be judged to have provided sufficient evidence to proceed to the Phase III component if the ORR is at least 25% (see Section 11.2c for details).

<u>Phase III Component:</u> The Phase III design has two co-primary objectives: an evaluation of IA-PFS and OS.

i. The primary PFS objective within the Phase III component is to compare investigator-assessed progression-free survival (IA-PFS) in patients with advanced stage refractory SCCA of the lung randomized to receive investigational therapy versus SoC.

ii. The primary OS objective within the Phase III component is to compare overall survival (OS) in patients with advanced stage refractory SCCA of the lung randomized to receive investigational therapy versus SoC.

2. Secondary Endpoints

Phase II component

i. To evaluate PFS and OS with investigational therapy

ii. To evaluate the duration of response (DoR) among patients who achieve a CR or PR by RECIST 1.1

iii. To evaluate the severity of toxicities associated with investigational therapy versus SoC.

3. Phase III Component

i. To compare the response rates (confirmed and unconfirmed, complete and partial) among patients randomized to receive investigational therapy versus SoC.

ii. To evaluate the frequency and severity of toxicities associated with investigational therapy versus SoC.

4. Sample Size with Power Justification

i. Phase II Design

A design with 90% power and 1-sided 0.05 level type I error would require 40 patients to rule out an ORR of 15% or less if the true ORR is 35% or greater. The observation of at least 10 responses of the 40 patients (25%) would be considered evidence to rule out an ORR of 15%.

A key secondary objective is an assessment of median IA-PFS (mPFS). If the ORR rate is less than 25% but the mPFS is at least 4.5 months, this may be considered sufficient evidence to continue to the follow-on Phase III. With 40 patients, this design has 90% power to rule out a median PFS of 3 months or less, if the true mPFS is 6 months, at the 0.05 1-sided level. The observation of an mPFS of at least 4.5 months would be considered evidence to rule out a mPFS of 3 months or less.

ii. Follow-on Phase III Design (if feasible)

If the conditions for continuation at the phase II are met and it is feasible to accrue to a phase III, then a follow-on randomized Phase III will be initiated. Evaluation of the feasibility of a phase III will commence around 75% accrual to the phase II. The SoC arm of the phase III study will not be defined until this time. The follow-on phase III will be submitted as an amendment.

The total sample size for the phase III is determined based on the biomarker prevalence, with all other design parameters the same across sub-studies using this design. Patients enrolled during the phase II component will not be included in the phase III study analyses.

While a co-primary objective is the comparison of IA-PFS between the arms, the sample size for Phase III component is based on a

design that targets a 75% improvement in median OS (HR=0.57) with 80% power and a 2.5% 1-sided type I error rate. This design requires 100 deaths. The median OS and PFS and associated sample size assumptions will be updated for each sub-study in the planning stage once a control arm has been identified.

iii. Phase III Feasibility Considerations

A follow-on randomized Phase III trial will be considered as feasible if the expected duration of accrual is approximately 3 years or less. Upon 75% accrual to the Single Arm component, the accrual rate to the sub-study will be assessed and if the average monthly accrual rate is at least 2-3 patients per month, depending on the designed total study accrual the Phase III will be considered feasible.

iv. Phase II Expansion Considerations

Accrual to a Phase III Trial is Not Feasible
If the Phase II data meets the definition of a positive Single Arm Phase II as defined in Section 11.2c (above) but it is determined that a Phase III trial is not feasible, accrual to the Single Arm Phase II may be expanded beyond 40 patients. If the ORR is at least 35% (the phase II alternative hypothesis) then the Phase II expansion will continue accrual for up to an additional 12 months or up to an additional 20 patients (whichever is reached first).

Accrual to a Phase III Trial Is Feasible
If feasible to accrual to a phase III and the ORR is greater than 40%, then discussions with between study leadership, the pharmaceutical collaborator, NCI, and FDA will commence to discuss the potential for FDA approval based on data from a Single Arm evaluation of the investigational therapy. In such a case, accrual will likely be expanded to a total of up to 100 patients treated with the investigational therapy and evaluate the response rate, duration of response, median PFS and OS, and toxicity rates in this expanded study population.

5. Analysis Plan

i. Phase II Analysis Plan

An interim analysis will take place when 20 patients are evaluable for response, with no halt in accrual. This interim analysis will only evaluate early stopping for futility. If 2 or fewer responses are observed, this will be considered evidence of futility and the recommendation will be to close the study for lack of evidence of efficacy of the regimen. If the study continues to full accrual, the observation of at least 10 responses will be considered evidence to rule out the null hypothesis of a 15% response rate.

Response rates and associated confidence intervals will be calculated. Survival IA-PFS, and DoR will be estimated using the method of Kaplan-Meier. The Brookmeyer-Crowley method will be used to calculate confidence intervals for median OS, IA-PFS, and DoR. With 40 patients, ORR and toxicity rates can be estimated within 16% with 95% confidence. Any toxicity with at least 5% prevalence has at least an 87%chance of being observed.

ii. Phase III Analysis Plan Including Plans for Formal Interim Analysis

The Phase III primary analyses will be performed on an intent-to-treat basis. Stratified (using randomization stratification factors) log-rank test will be used to test the primary hypotheses related to PFS and OS comparing the two treatment arms at the levels specified. A Cox proportional hazards (PH) model will be used to estimate the hazard ratios and associated confidence intervals. Analysis of response rates and toxicities will be performed using a chi-square or Fisher's exact test, as appropriate.

Secondary analysis of PFS and OS at the final analysis within each sub-study will be a weighted analysis (using Cox regression) standardizing the biomarker prevalence among patients with multiple biomarkers to the study population prevalence among all patients eligible as defined by the sub-study biomarker definition. The goal of this analysis is to estimate the treatment effects had all patients with the sub-study biomarker been assigned to the sub-study (as in a stand-alone trial).

Phase III Interim Analysis: If a Phase III trial is initiated, a formal interim analysis is planned when 50% of the expected deaths have been observed with rules specified on the fixed-sample p-value scale.

Evidence suggesting early termination of the trial for futility/harm will be if the alternative hypothesis of at least 50% improvement in OS for the investigational arm is rejected at the one-sided level 0.0025, using a PH model score test evaluated at the alternative hypothesis (Ha: HR=0.67 for OS). *(11)* Evidence suggesting early termination of the trial for superiority will be if the hypothesis of no difference in OS is rejected at the one-sided 0.0025 level using a log-rank test.

iii. Final Analysis

If the decision is to continue the study to completion, the final analysis of IA-PFS will occur after completion of accrual, when either 100 progression events have been observed or the final analysis for OS is performed, whichever comes first. The final analysis of OS will take place when at least 100 events have been observed. However, should events accumulate more slowly than anticipated, the maximum time to final analysis is when 18 months have passed since the completion of accrual. The analysis of IA-PFS will be a test against the null hypothesis at the 0.0001 level (which has 80% power for a 2.5-fold increase in IA-PFS), with an approximate observed threshold of 2-fold improvement in median IA-PFS (HR=0.5) for significance (deemed both significant in statistical and clinical terms). The final analysis of OS will be at level 0.024 to account for the effects of interim monitoring (using a modified Haybittle-Peto boundary). The adjusted level does not account for either the IA-PFS assessment for futility or the response assessment for efficacy at the Phase II interim analysis as the level of testing response is small (0.0001) and the decision to stop for efficacy at this time point will also consider other clinical data.

The actual decision to terminate accrual early will be made by the SWOG Data and Safety Monitoring Committee (DSMC), and will consider IA-PFS, OS, response, and toxicities.

b. Option for Non-Match Sub-Studies

This design is specific to non-match therapies in patients who have previously received and progressed on/following immunotherapy. Consideration for pursuing a Phase III trial, should a Phase II successfully reject the null hypothesis (as described below), is not automatic and would follow careful review of the study data and discussions with appropriate parties. The details here are specific to the Phase II design.

1. Primary Endpoint

The primary objective is to evaluate the objective response rate (ORR) (confirmed and unconfirmed, complete and partial) in patients treated with investigational non-match therapy with advanced stage refractory squamous cell carcinoma (SCCA) of the lung.

2. Secondary Endpoints

i. To evaluate PFS and OS with investigational therapy

ii. To evaluate the duration of response (DoR) among patients who achieve a CR or PR by RECIST 1.1

iii. To evaluate the severity of toxicities associated with investigational therapy versus SoC.

3. Sample Size with Power Justification

The accrual goal for this design is to accrue up to 60 eligible patients. A design with 82% power and 1-sided 0.05 level type I error would require 60 patients to rule out an ORR of 5% or less if the true ORR is 15% or greater. The observation of at least 6 responses of the 60patients (10%) would be considered evidence to rule out an ORR of 5%.

4. Analysis Plan

This design includes two interim analyses and a final analysis.

<u>Interim 1:</u> The first interim analysis will take place when 20 patients are evaluable for response. This interim analysis will only evaluate early stopping for futility. If no responses are observed, this will be considered evidence of futility and the recommendation will be to close the cohort for lack of evidence of efficacy of the regimen.

In addition to evaluating early stopping for futility, this analysis will evaluate the safety/tolerability of the regimen.

<u>Interim 2</u>: The second interim analysis will take place when 40 patients are evaluable for response. This interim analysis will also only evaluate early stopping for futility. If 3 or fewer responses are observed, this will be considered evidence of futility and the recommendation will be to close the cohort for lack of evidence of efficacy of the regimen.

Final Analysis: If a study is not closed to accrual at the first or second interim analysis, the study will proceed to full accrual of 60 evaluable patients. If the study continues to full accrual, the observation of at least 6 responses will be considered evidence to rule out the null hypothesis of a 5% response rate.

Summary of analyses:

Analysis	Sample Size	Futility	Continue/Positive
1st interim	20 patients	No responses	1+ response (5% RR)
2nd interim	40 patients	≤3 responses	4+ responses (10% RR)
Final	60 patients	≤5 responses	6+ responses (10% RR)

Evaluability for response: Evaluability for response is defined based on RECIST 1.1 and accrual to each cohort is to remain open while patients are being evaluated for response. However, if 20 or more eligible patients for the first interim analysis or 40 or more eligible patients for the second interim analysis, have made it to their second disease assessment and the required number of responses to continue past that interim analysis has not been observed, then the accrual will be placed in temporary closure until the response status for all patients in the interim analysis set is known.

The following table describes the likelihood of stopping at the interim analyses under a response rate of 5%, 10%, 15%, and 20% and the probability of a rejecting the null.

RR	Probability of stopping Interim 1	Probability of stopping Interim 2	Average sample size	Expected number of responses	Probability reject null
5%	36%	51%	36	2	5.6%
10%	12%	31%	49	5	46.8%
15%	4%	11%	56	8	82.2%
20%	1%	2%	59	12	96.5%

Response rates and associated confidence intervals will be calculated. Survival, IA-PFS, irRC-IA-PFS, and DoR will be estimated using the method of Kaplan-Meier. The Brookmeyer-Crowley method will be used to calculate confidence intervals for median OS, IA-PFS, and DoR. With 60 patients, ORR and toxicity rates can be estimated within 13% with 95% confidence. Any toxicity with at least 5% prevalence has at least a 95% chance of being observed.

With 60 patients, OS and PFS rates at landmark times can be estimated within 13% with 95% confidence.

11.3 Blinded Independent Centralized Review (BICR) of PFS and/or ORR

BICR is triggered if sub-study data is to be submitted to the FDA to support registration of the investigational therapy. If a sub-study is stopped at the Phase II analysis a complete case BICR will be performed. Otherwise, the BICR audit plan will proceed using the algorithm detailed in Dodd, et al (2011). PFS by Central Review (PFS-CR) is defined in Section 10.8. For the purposes of a PFS audit, the clinical irrelevance factor (CIF) will be the HR threshold of 0.57 (or equivalently an approximate 75% improvement in median PFS) as defined above to be a clinically meaningful difference. The initial audit size will be 33% of the total patients enrolled within a sub-study (approximately 100 patients). Using this initial audit sample, the audit sample size will be calculated based on the formula provided in Dodd et al. If the audit sample size is less than 33%, the audit PFS HR will be calculated as defined in Dodd, et. al 2011. *(16)* If the audit sample size is >33%, but less than 80%, then additional patient scans will be evaluated to increase the audit sample to the specified size. If the audit sample size is determined to be > 80% of the total sample size, a complete case audit will be performed. To define the equivalence region for the local and central review HR estimates an $\alpha = 0.05$ will be used (see Steps 5, 6, and 7 of the algorithm). The specific details of BICR progression assessment are included in the Risk Based Monitoring Plan (see Section 18.1d)

11.4 Accrual Information

There are 55,000 new cases of squamous cell carcinoma (SCCA) of the lung diagnosed each year in the United States and approximately 57% of these patients present with metastatic disease at diagnosis. Approximately 70% of early stage patients progress following initial therapy and essentially all metastatic patients (95%) progress. Further we assume that 50-60% of these patients will go on to receive 2nd and greater lines of therapy. It is therefore anticipated that the approximate available patient pool is 28,800 patients per year and of them, 2-3% will participate in this clinical trial.

Accrual projections are based on the following assumptions:

a. Pre-screening is allowed after year 1 of study. Once pre-screening is allowed, 50% of patients will be pre-screened and 50% of patients will be screened at progression. This implies that 50% of patients will be register to their assigned sub-study as soon as they are notified of their sub-study assignment and 50% of patients will need to progress on their current treatment and may even receive subsequent lines of treatment prior to registering to a sub-study.

b. The median time to enrollment for patients screened at progression is 2 months and the distribution of enrollment times is exponential. This assumption accounts for the time for sites and patients to work up patients and address any clinical issues prior to enrolling onto a sub-study.

c. The median time to enrollment for patients screened prior to progression is 9 months and the distribution of enrollment times is exponential. This assumption accounts for the time to progression on current therapy and potentially receipt of an additional line of therapy after progressing on therapy during pre-screening and prior to sub-study registration.

d. The anticipated screen success rate (percentage of patients screened who eventually enroll onto a substudy) is 50-60% in the first year of study and 70% thereafter, for patients screened at progression and 50% for patients pre-screened during prior treatment.

e. The rate of patients registering to **S1400** to be screened or pre-screened will be 300-500 patients/year in the first year and a half of study, between 400 and 600 in the 2.5-3.5 years after study activation, and between 500 and 800 patients per year thereafter.

Under these assumptions, the projected accrual to **S1400** and to the sub-studies is presented in the following Table 2:

Table 2	Total **S1400** Registrations	Total Sub-study Registrations
12 months	270-434	106-155
18 months	469-718	152-248
24 months	699-1035	224-362
36 months	1203-1839	474-722
48 months	1707-2643	734-1171
60 months	2211-3447	1032-1633

11.5 Screening Success Rate Evaluation

The screen success rate (SSR) is defined as the ratio of the number of patients that register to a sub-study (register to **S1400A**, **S1400B**, **S1400C**, etc.) over the total number of patients screened (i.e. that register to **S1400**). The SSR will be evaluated on a bi-annual basis and reported as part of the SWOG Group Meeting study reports to the SWOG Data and Safety Monitoring Committee. The SSR will be evaluated on 1) all **S1400** screen registrations, 2) among patients who are pre-screened during current treatment, and 3) among patients screened at progression.

Formal assessment of screening rates will occur annually and will be reported to the NCI and the Thoracic Malignancy Steering committee at the time of the SWOG Spring Group meetings. Consideration for discontinuation of pre-screening patients during current therapy will be the observation of less than a 40% screen success rate or if screen success rate in the pre-screened group is less than half the rate in the set of patients screened at progression.

11.6 Treatment Arm Randomization Acceptance Rate Evaluation

This evaluation only applies to randomized studies; this does not apply to the single arm component of Design #2. The treatment arm randomization acceptance rate (TARAR) within each treatment arm of each randomized sub-study is defined as the ratio of the number of patients who receive any protocol treatment over the number that are randomized to that sub-study treatment arm. The TARAR will be evaluated on a bi-annual basis and reported as part of the SWOG Group Meeting study reports to the SWOG Data and Safety Monitoring Committee.

Formal assessment of the TARAR will occur annually and will be reported to the NCI, and the Thoracic Malignancy Steering committee at the time of the SWOG Spring Group meetings.

11.7 Data and Safety Monitoring

A Data and Safety Monitoring Committee will oversee the conduct of the study. The Committee consists of four members from outside of the SWOG, 3 SWOG members, 3 non-voting representatives from the National Cancer Institute (NCI), and the Group Statistician (non-voting). The members of this Committee will receive confidential reports

every 6 months from the SWOG Statistical Center, and will meet at the Group's bi-annual meetings as necessary. The Committee will be responsible for decisions regarding possible termination and/or early reporting of the study.

12.0 DISCIPLINE REVIEW

This section does not apply to this study.

13.0 REGISTRATION GUIDELINES

13.1 Registration Timing

a. Patients must register to the **S1400** screening step within 5 calendar days prior to submitting tissue to Foundation Medicine.

b. Patients must plan to begin treatment within 7 calendar days after sub-study registration.

13.2 Investigator/Site Registration

Prior to the recruitment of a patient for this study, investigators must be registered members of a Cooperative Group. Each investigator must have an NCI investigator number and must maintain an "active" investigator registration status through the annual submission of a complete investigator registration packet to CTEP.

a. CTEP Registration Procedures

Food and Drug Administration (FDA) regulations and National Cancer Institute (NCI) policy require all individuals contributing to NCI-sponsored trials to register and to renew their registration annually. To register, all individuals must obtain a Cancer Therapy Evaluation Program (CTEP) Identity and Access Management (IAM) account (https://ctepcore.nci.nih.gov/iam). In addition, persons with a registration type of Investigator (IVR), Non-Physician Investigator (NPIVR), or Associate Plus (AP) (i.e., clinical site staff requiring write access to OPEN, RAVE, or TRIAD or acting as a primary site contact) must complete their annual registration using CTEP's web-based Registration and Credential Repository (RCR) (https://ctepcore.nci.nih.gov/rcr). Documentation requirements per registration type are outlined in the table below.

Documentation Required	IVR	NPIVR	AP	A
FDA Form 1572	✔	✔		
Financial Disclosure Form	✔	✔	✔	
NCI Biosketch (education, training, employment, license, and certification)	✔	✔	✔	

Documentation Required	IVR	NPIVR	AP	A
HSP/GCP training	✔	✔	✔	
Agent Shipment Form (if applicable)	✔			
CV (optional)	✔	✔	✔	

An active CTEP-IAM user account and appropriate RCR registration is required to access all CTEP and CTSU (Cancer Trials Support Unit) websites and applications. In addition, IVRs and NPIVRs must list all clinical practice sites and IRBs covering their practice sites on the FDA Form 1572 in RCR to allow the following:

- Added to a site roster
- Assigned the treating, credit, consenting, or drug shipment (IVR only) tasks in OPEN
- Act as the site-protocol PI on the IRB approval
- Assigned the Clinical Investigator (CI) role on the Delegation of Tasks Log (DTL).

Additional information can be found on the CTEP website at https://ctepcore.nci.nih.gov/iam/index.jsp For questions, please contact the RCR **Help Desk** by email at RCRHelpDesk@nih.gov.

b. CTSU Registration Procedures

This study is supported by the NCI Cancer Trials Support Unit (CTSU).

1. **Protocol Specific Tranining (PSR)**

A member of each institution (CRA or investigator, etc.) must complete the Protocol Specific Requirements (PSR) prior to patient registration. The PSR will need to be renewed prior to patient registration each time a new sub-study has been added. The PSR can be satisfied by completing the training online and submitting the verification at: https://swog.org/members/Training/S1400Training.asp.

To receive credit, institutions are to complete and submit the verification form located at the bottom of the webpage. The SWOG Protocol Coordinator and CTSU will be notified of completion. There is a turn-around time of three business days for processing this training requirement at CTSU after submission of the verification form before the first registration may occur. However, if a registration is pending, please notify the CTSU Regulatory staff at CTSURegOffice@ecogchair.org so they may be prompted to process the training completion as a priority.

2. **IRB Approval:**

Each investigator or group of investigators at a clinical site must obtain IRB approval for this protocol and submit IRB approval and supporting

documentation to the CTSU Regulatory Office before they can be approved to enroll patients. Assignment of site registration status in the CTSU Regulatory Support System (RSS) uses extensive data to make a determination of whether a site has fulfilled all regulatory criteria including but not limited to the following:

- An active Federal Wide Assurance (FWA) number
- An active roster affiliation with the Lead Network or a participating organization
- A valid IRB approval
- Compliance with all protocol specific requirements.

In addition, the site-protocol Principal Investigator (PI) must meet the following criteria:

- Active registration status
- The IRB number of the site IRB of record listed on their Form FDA 1572
- An active status on a participating roster at the registering site.

3. **Downloading Site Registration Documents:**

Site registration forms may be downloaded from the **S1400** or the specific sub-study protocol page located on the CTSU members' website. Permission to view and download this protocol and its supporting documents is restricted and is based on person and site roster assignment housed in the CTSU RSS.

- Go to https://www.ctsu.org and log in to the members' area using your CTEP-IAM username and password
- Click on the Protocols tab in the upper left of your screen
- Either enter the protocol # in the search field at the top of the protocol tree, or
- Click on the By Lead Organization folder to expand
- Click on the SWOG link to expand, then select S1400 or specific sub-study.
- Click on LPO Documents, select the Site Registration documents link, and download and complete the forms provided.

4. **Requirements for Site Registration:**

- CTSU Transmittal Sheet (optional)
- IRB approval (For sites not participating via the NCI CIRB; local IRB documentation, an IRB-signed CTSU IRB Certification Form, Protocol of Human Subjects Assurance Identification/IRB Certification/Declaration of Exemption Form, or combination is accepted)
- Other site registration requirements (i.e., laboratory certifications, protocol-specific training (PSR) certifications, or modality credentialing) must be submitted to the CTSU Regulatory Office or compliance communicated per protocol instructions.

5. **Submitting Regulatory Documents:**

Submit required forms and documents to the CTSU Regulatory Office via the Regulatory Submission Portal, where they will be entered and tracked in the CTSU RSS.

Regulatory Submission Portal: www.ctsu.org (members' area) → Regulatory Tab → Regulatory Submission

When applicable original documents should be mailed to:
CTSU Regulatory Office
1818 Market Street, Suite 1100
Philadelphia, PA 19103

6. **Checking Site's Registration Status:**

Site registration status can be verified on the members' section of the CTSU website.

- Go to https://www.ctsu.org and log in to the members' area using your CTEP-IAM username and password
- Click on the Regulatory tab at the top of your screen
- Click on the Site Registration tab
- Enter your 5-character CTEP Institution Code and click on Go

- Note: The status given only reflects compliance with IRB documentation and institutional compliance with protocol-specific requirements as outlined by the Lead Network. It does not reflect compliance with protocol requirements for individuals participating on the protocol or the enrolling investigator's status with the NCI or their affiliated networks.

13.3 OPEN Registration Requirements

The individual registering the patient must have completed the appropriate SWOG Registration Worksheet. The completed form must be referred to during the registration but should not be submitted as part of the patient data.

Patient enrollment will be facilitated using the Oncology Patient Enrollment Network (OPEN). OPEN is a web-based registration system available on a 24/7 basis. To access OPEN, the site user must have an active CTEP-IAM account (check at https://ctepcore.nci.nih.gov/iam) and a 'Registrar' role on either the LPO or participating organization roster. Registrars must hold a minimum of an AP registration type. If a DTL is required for the study, the registrar(s) must also be assigned the OPEN Registrar task on the DTL.

OPEN will also ask additional questions that are not present on the SWOG Registration Worksheet. The individual registering the patient must be prepared to provide answers to the following questions:

a. Institution CTEP ID

b. Protocol Number

c. Registration Step

d. Treating Investigator

e. Cooperative Group Credit

f. Credit Investigator

g. Patient Initials

h. Patient's Date of Birth

i. Patient SSN (SSN is desired, but optional. Do not enter invalid numbers.)

j. Country of Residence

k. ZIP Code

l. Gender (select one):
- Female Gender
- Male Gender

m. Ethnicity (select one):
- Hispanic or Latino
- Not Hispanic or Latino
- Unknown

n. Method of Payment (select one):
- Private Insurance
- Medicare
- Medicare and Private Insurance
- Medicaid
- Medicaid and Medicare
- Military or Veterans Sponsored NOS
- Military Sponsored (Including Champus & Tricare)
- Veterans Sponsored
- Self Pay (No Insurance)
- No Means of Payment (No Insurance)
- Other
- Unknown

o. Race (select all that apply):
- American Indian or Alaska Native
- Asian
- Black or African American
- Native Hawaiian or other Pacific Islander
- White
- Unknown

13.4 Registration Procedures

a. All site staff will use OPEN to enroll patients to this study. OPEN is integrated with the CTSU Enterprise System for regulatory and roster data and initializes the patient in the RAVE database. OPEN can be accessed at https://open.ctsu.org or from the OPEN tab on the CTSU members' side of the website at https://www.ctsu.org, or from the OPEN Patient Registration link on the SWOG CRA Workbench. Patient re-registrating to a sub-study will follow the same the same instructions as above. To assign an IVR or NPIVR as the treating, crediting, consenting, drug shipment (IVR only), or investigator receiving a transfer in OPEN, the IVR or NPIVR must list on their Form FDA 1572 in RCR the IRB number used on the site's IRB approval. If a DTL is required for the study, the IVR or NPIVR must also be assigned the appropriate OPEN-related tasks on the DTL.

b. Prior to accessing OPEN site staff should verify the following:

- All eligibility criteria have been met within the protocol stated timeframes and the affirmation of eligibility on the Registration Worksheet has been signed by the registering investigator or another investigator designate. Site staff should refer to Section 5.0 to verify eligibility

- All patients have signed an appropriate consent form and HIPAA authorization form (if applicable).

- The study site is listed as “approved” in the CTSU RSS.

c. Access requirements for OPEN:

The OPEN system will provide the site with a printable confirmation of registration and treatment information. Please print this confirmation for your records.

d. Further instructional information is provided on the OPEN tab of the CTSU members’ side of the CTSU website at https://www.ctsu.org or at https://open.ctsu.org. For any additional questions contact the CTSU Help Desk at 1-888-823-5923 or ctsucontact@westat.com.

13.5 Exceptions to SWOG registration policies will not be permitted.

a. Patients must meet all eligibility requirements.

b. Institutions must be identified as approved for registration.

c. Registrations may not be cancelled.

d. Late registrations (after initiation of treatment) will not be accepted.

14.0 DATA SUBMISSION SCHEDULE

14.1 Data Submission Requirements for Screening/Pre-Screening Registration

NOTE: See Section 14.0 for each sub-study data submission requirements.

Data must be submitted according to the protocol requirements for **ALL** patients registered, whether or not assigned treatment is administered, including patients deemed to be ineligible. Patients for whom documentation is inadequate to determine eligibility will generally be deemed ineligible.

14.2 Master Forms

Master forms can be found on the protocol abstract page on the SWOG website (www.swog.org) and (with the exception of the sample consent form and the Registration Worksheet) must be submitted on-line via the Web; see below for details.

14.3 Data Submission Procedures

Data collection for this study will be done exclusively through the Medidata Rave clinical data management system. Access to the trial in Rave is granted through the iMedidata application to all persons with the appropriate roles assigned in Regulatory Support System (RSS). To access Rave via iMedidata, the site user must have an active CTEP-IAM account (check at < https://eapps-ctep.nci.nih.gov/iam/index.jsp >) and the appropriate Rave role (Rave CRA, Read-Only, Site Investigator) on either the LPO or participating

organization roster at the enrolling site. To hold the Rave CRA role or CRA Lab Admin role, the user must hold a minimum of an AP registration type. To hold the Rave Site Investigator role, the individual must be registered as an NPIVR or IVR. Associates can hold read-only roles in Rave. If the study has a DTL, individuals requiring write access to Rave must also be assigned the appropriate Rave tasks on the DTL.

Upon initial site registration approval for the study in RSS, all persons with Rave roles assigned on the appropriate roster will be sent a study invitation e-mail from iMedidata. To accept the invitation, site users must log into the Select Login (https://login.imedidata.com/selectlogin) using their CTEP-IAM user name and password, and click on the “accept” link in the upper right-corner of the iMedidata page. Please note, site users will not be able to access the study in Rave until all required Medidata and study specific trainings are completed. Trainings will be in the form of electronic learnings (eLearnings), and can be accessed by clicking on the link in the upper right pane of the iMedidata screen.

Users that have not previously activated their iMedidata/Rave account at the time of initial site registration approval for the study in RSS will also receive a separate invitation from iMedidata to activate their account. Account activation instructions are located on the CTSU website, Rave tab under the Rave resource materials (Medidata Account Activation and Study Invitation Acceptance). Additional information on iMedidata/Rave is available on the CTSU members’ website under the Rave tab at www.ctsu.org/RAVE/ or by contacting the CTSU Help Desk at 1-888-823-5923 or by e-mail at ctsucontact@westat.com.

a. All participating institutions must submit data electronically via the Web using Medidata Rave® at the following url:

   https://login.imedidata.com/selectlogin

   1. If prompted, select the ‘CTEP-IAM IdP’ link.
   2. Enter your valid and active CTEP-IAM userid and password. This is the same account used for the CTSU members’ web site and OPEN.

b. You may also access Rave® via the SWOG CRA Workbench. Go to the SWOG web site (http://swog.org) and logon to the Members Area using your SWOG Roster ID Number and password. After you have logged on, click on *Workbenches*, then *CRA Workbench* to access the home page for the CRA Workbench and follow the link to Rave® provided in the left-hand navigation panel.

   To access the CRA Workbench the following must be done (in order):

   1. You are entered into the SWOG Roster and issued a SWOG Roster ID Number,
   2. You are associated as an investigator or CRA/RN at the institution where the patient is being treated or followed,
   3. Your Web User Administrator has added you as a web user and has given you the appropriate system permissions to view data for that institution.

   For assistance with points 1 and 2 call the Operations Office at 210/614-8808. For point 3, contact your local Web User Administrator (refer to the "Who is my Web User Administrator?" function on the swog.org Members logon page).

   For difficulties with the CRA Workbench, please email technicalquestion@crab.org.

c. Institutions participating through the Cancer Trials Support Unit (CTSU) please refer to the CTSU Participation Table on Page 5.

14.4 Data Submission Overview and Timepoints for Screening/Pre-Screening Registration

a. WITHIN 7 DAYS OF SCREENING/PRE-SCREENING REGISTRATION, SUBMIT:

S1400 Onstudy Form

Pathology report for initial diagnosis documenting histologic confirmation of squamous cell lung cancer.*

S1400 Local Pathology Review Form*

* NOTE: Upload via the Source Documentation: Baseline form in Rave®.

For patients who consent to the optional **S1400GEN** Survey Ancillary Study, submit the patient and physician contact information page from the patient consent form per Section 15.5b.3.

b. WITHIN 5 CALENDAR DAYS AFTER SCREENING/PRE-SCREENING REGISTRATION, SUBMIT TISSUE REQUIRED FOR BROAD PLATFORM CLIA BIOMARKER PROFILING:

Submit specimens to Foundation Medicine for biomarker profiling as specified in Section 15.0.

c. IF PATIENT CONSENTS SUBMIT OPTIONAL SPECIMENS:

Submit optional specimens for translational medicine studies and banking as specified in Section 15.0.

d. WITHIN 21 DAYS OF RECEIVING SUB-STUDY ASSIGNMENT:

Submit the **S1400** Status Update

e. EVERY THREE MONTHS FOR THE FIRST YEAR AFTER SCREENING/PRE-SCREENING REGISTRATION, THEN EVERY SIX MONTHS FOR THE SECOND YEAR AND ANNUALLY THEREAFTER UNTIL SUB-STUDY REGISTRATION:

Submit the Advanced NSCLC Follow-Up Form.

f. **PRE-SCREENED PATIENTS ONLY**: WITHIN 7 DAYS OF PROGRESSION:

Submit the **S1400** Notice of Progression.

g. AFTER RECEIVING SUB-STUDY ASSIGNMENT BUT PRIOR TO REGISTRATION ON ANY SUB-STUDY: IF PATIENT DOES NOT FULFILL THE SUB-STUDY SPECIFIC ELIGIBILITY CRITERIA:

Submit the **S1400** Request for Sub-Study Reassignment Form

14.5 Data Submission **FOR PATIENTS WHO WILL NOT BE REGISTERED TO ANY OF THE SUB-STUDIES**

a. WITHIN 7 DAYS OF DECISION NOT TO REGISTER PATIENT:

Submit the **S1400** Notice of Intention Not to Register.

All patients will receive a notification email with sub-study assignment along with complete gene panel results, provided the submitted tissue is adequate for testing. If the **S1400** Notice of Intention Not to Register Form is submitted prior to receipt of the notification email, it will be sent as usual upon receipt of the biomarker analysis from FMI.

b. EVERY SIX MONTHS FOR THE FIRST 2 YEARS AFTER SCREENING/PRE-SCREENING REGISTRATION THEN AT THE END OF YEAR 3 OR DEATH, WHICHEVER OCCURS FIRST:

Submit the Advanced NSCLC Follow-Up Form

c. WITHIN 28 DAYS OF KNOWLEDGE OF DEATH:

Submit the Notice of Death

14.6 Data Submission FOR PATIENTS WHO HAVE PROGRESSED ON A SUB-STUDY AND WISH TO REGISTER TO A NEW SUB-STUDY

a. AFTER PROGRESSION ON A SUB-STUDY:

Submit the **S1400** Request for New Sub-Study Assignment

Sites will receive a notification email with a new sub-study assignment, based on patient's biomarker results.

b. WITHIN 21 DAYS OF RECEIVING NEW SUB-STUDY ASSIGNMENT:

Submit the **S1400** Status Update Form

c. AFTER RECEIVING NEW SUB-STUDY ASSIGNMENT BUT PRIOR TO REGISTRATION ON NEW SUB-STUDY, IF PATIENT DOES NOT FULFILL THE SUB-STUDY SPECIFIC ELIGIBILITY CRITERIA:

Submit the **S1400** Request for Sub-Study Reassignment Form

d. UNTIL REGISTRATION TO NEW SUB-STUDY:

Continue to follow patient and submit Advanced NSCLC Follow-up Forms per Sections 9 and 14.4 of their current sub-study. After registration to the new sub-study, follow-up is still required on the previous sub-study.

e. IF PATIENT WILL NOT BE REGISTERED TO THE NEW SUB-STUDY:

WITHIN 7 DAYS OF DECISION NOT TO REGISTER PATIENT: Submit the **S1400** Notice of Intention Not to Register

Continue to follow patient and submit forms per Sections 9 and 14.4 of the most recent sub-study in which patient is enrolled.

15.0 SPECIAL INSTRUCTIONS

15.1 SWOG Specimen Tracking System (STS)

All specimen submissions for this study must be entered and tracked using the online SWOG Specimen Tracking System (STS). SWOG members may log on to STS via the CRA Workbench. To access the CRA Workbench, go to the SWOG Web site (http://swog.org) and logon to the Members Area. After you have logged on using your SWOG roster ID number and password, click on the *CRA Workbench* link to access the home page for CRA Workbench website. Non-SWOG members may access SpecTrack using their CTSU UserID and password on the SpecTrack login page located at https://spectrack.crab.org (select the option "SWOG - SWOG - CTSU"). SpecTrack start-up instructions (both written and demo) are available after signing in to SpecTrack.

A copy of the Shipment Packing List produced by the online Specimen Tracking system should be printed and placed in the pocket of the specimen bag if it has one, or in a separate resealable bag. STS laboratory IDs are used to identify laboratories to which specimens are shipped.

ALL SPECIMENS MUST BE LOGGED VIA THIS SYSTEM; THERE ARE NO EXCEPTIONS.

To report technical problems with Specimen Tracking, such as database errors or connectivity issues, please send an email to technicalquestion@crab.org. For procedural help with logging and shipping specimens, there is an introduction to the system on the Specimen Tracking main page (https://spectrack.crab.org/Instructions); or contact the Data Operations Center at 206/652-2267 to be routed to the Data Coordinator for further assistance.

15.2 Biomarker Profiling (Required)

Note: Previous NGS testing outside this study must be repeated for sub-study assignment.

Tissue for biomarker profiling and c-MET testing must be submitted within 5 calendar days of **S1400** screening/pre-screening registration.

a. The following must be submitted to Foundation Medicine, Lab #211:

1. Tissue block (preferred) or at least 12 (4-5 micron) unstained slides (20 slides are strongly recommended).

2. Hematoxilyn-eosin (H&E)-stained slide or Aperio H&E-stained slide. If H&E slide is not available, submit an extra unstained slide. An H&E slide is not required with tissue block submission.

3. Local pathology report from initial diagnosis

4. **S1400** Local Pathology Review Form:

   Tumor material must be reviewed by a local pathologist to ensure sufficient tumor cells are present in the sample. The local pathologist must review and sign off on the **S1400** Local Pathology Review form prior to enrolling patient noting that the tumor volume is at least 0.2 mm^3 and the tumor tissue contains at least 20% viable tumor cells. This form must be included with the tissue submitted to Foundation Medicine. In addition, this form must also be uploaded via the Source Documentation: Baseline form in Rave®.

Specimen collection kits are not being provided for this submission; sites will use institutional supplies.

b. When submitting tissue to Foundation Medicine, be prepared to provide answers to the following questions in the SWOG specimen Tracking System:

- Patient's date of birth
- Patient's sex
- Has the patient had any type of transplant? (yes/no)
- Patient's diagnosis (NSCLC)
- Site's local specimen ID number

- Tumor histology (Squamous)
- Primary tumor site
- Specimen tumor site (Lung, Lymph node, Other)
- Fine needle aspirate (Yes, No)
- Biopsy (Screening biopsy – CT image guided, Screening Biopsy – bronchoscopy, Archival Tissue)
- Date unstained slides were cut

In the online specimen tracking system, the appropriate SWOG laboratory for submission of tumor tissue for SWOG Repository Submission and next-generation DNA sequencing testing is identified as follows:

Lab #211: Foundation Medicine Inc.
Attn: Prospective Study Specimen

Contact: FMI S1400 Distribution List
Phone: 617/418-2200
E-mail: S1400@foundationmedicine.com

c. Tissue Specifications

Tissue biopsies are requested. Most optimally, core needle biopsies should be obtained. Cytology smear specimens are <u>not</u> sufficient for the molecular studies proposed. However, fine needle aspirates with good cellularity are acceptable as long as cell blocks are established. Specimens containing less than 80% nucleated cells require greater total volume and may not be suitable to assay. The key sample consideration is to submit a total mass of cells that is sufficient to extract the amount of DNA necessary for analysis. Bone biopsies are not allowed.

If patient's archival tumor material is not adequate for biomarker profiling, tumor tissue from a new biopsy must be obtained and submitted as outlined above (see **<u>S1400</u>** Funding Memorandum for reimbursement details).

If biomarker profiling could not be obtained from the submitted tissue, additional tissue will be requested.

d. Specimen Use

1. Next-generation DNA Sequencing (NGS)

Foundation Medicine will perform next-generation DNA sequencing. The molecular panel will require 8-10 sections (4-5 microns, 40 microns total). Remaining tissue will be sent to the SWOG Specimen Repository-Solid Tissue, Myeloma and Lymphoma Division, Lab #201, for use of the Translational Medicine studies within any sub-study the patient is enrolled in. SWOG Specimen Repository will prepare and ship the required specimens to the appropriate laboratory. The specimen will be kept until

there are no additional sub-studies for the patient to enroll in or the tissue is used up, whichever happens first. With patient's consent, any leftover tissue will remain at the SWOG Repository for future exploratory analysis.

Please see Section 18.1a for more details.

2. C-MET Assay

The c-MET IHC assay will be part of **S1400** screening, to identify patients for registration to **S1400K**. The assay is currently being employed in AbbVie's Phase 1 study to select c-MET positive patients. The assay utilizes the CONFIRM c-MET SP44 rabbit monoclonal Ab from Ventana, intended for IVD use (cat #790-4430, Lot G08106). The IHC cutoff for positivity in the current Phase 1 study is an H-score of ≥150 membrane staining. In squamous histology NSCLC, about 30% of patients are c-MET positive is per the literature (Genentech) with SP44 antibody IHC.

For SP44 assay testing, no on-site processing of specimens will be required. The tissue specimens collected for **S1400** biomarker profiling will be used. All **S1400** tissue specimens are shipped to Foundation Medicine, Inc (FMI). For patients with tumor blocks, FMI will prepare two (4-5 micron) unstained, charged, and unbaked FFPE slides, per patient. The slides will be shipped to ARUP for staining within 2 calendar days of FMI receiving the specimens.

Please see Sub-Study **S1400K** for more details.

15.3 Correlative Studies and Banking (Optional for Patients)

Specimens for correlative studies and banking (submitted to the SWOG Specimen Repository – Solid Tissue, Myeloma and Lymphoma Division, Lab #201) are considered optional for the patient:

a. With patient's consent, any remaining tumor tissue will be sent from Foundation Medicine to the SWOG Specimen Repository - Solid Tissue, Myeloma and Lymphoma Division, Lab #201, for future exploratory analysis.

b. With patient's consent, Peripheral Blood must be collected and submitted as follows:

1. Specimen Collection

Specimens must be collected at Screening or Pre-screening within 42 days prior to registration to a sub-study. See Section 15.0 of each of the sub-studies for additional timepoints.

Approximately 8-10 mL of blood must be collected in EDTA tubes. Blood should be processed within one hour after venipuncture. If immediate processing within this time frame is not possible, EDTA tubes that are not processed immediately should be refrigerated at 4˚C. The approximate time from collection to processing should be recorded as part of the patient's source documentation. EDTA tubes must be centrifuged at 800 g for 10 minutes at 4˚C for the collection of plasma. Plasma must be transferred to one 15 ml centrifuge tube and spun again at 800 g for an additional 10 minutes. Plasma must then be pipetted into 1 ml coded cryovials at 0.5 ml aliquots. Plasma must be clear before freezing; no cells

or debris should be present. Each buffy coat layer (the gray-white layer at the interface of blood cells and plasma, approximately 1 ml) from the blood tube must each be transferred into appropriately labeled 2-ml cryovials. Samples must be placed immediately in a -80˚C freezer to ensure long-term viability.

2. Specimen Submission

Samples can be shipped in batches, at least every 3 months if not more frequently, to the SWOG Specimen Repository – Solid Tissue, Myeloma and Lymphoma Division, Lab #201.

Specimen collection and submission instructions can be accessed on the SWOG Specimen Submission webpage (http://swog.org/Members/ClinicalTrials/Specimens/STSpecimens.asp).

3. Specimen collection kits are not being provided for this submission; sites will use institutional supplies.

15.4 Radiology Review (Required for all Sub-studies)

CT, PET/CT, and/or MRI images must be locally read and interpreted by the local site radiology service. If patient is registered to an **S1400** sub-study, imaging exams must then be submitted to the Imaging and Radiation Oncology Core (IROC) at Ohio via TRIAD Imaging Submission procedures for central data collection and quality control (QC) check as well as retrospective central review.

CT, PET/CT, and/or MRI images must be submitted to IROC Ohio for central review at the following timepoints:

a. See Section 15 of each of the sub-studies for additional timepoints.

All study participants must have a CT (or MR or PET/CT) exam prior to sub-study entry. Participants must then undergo additional imaging until progression of disease, see Section 15 of each of the sub-studies for details. The same imaging modality used for the pre-treatment exam must be used for the post-treatment exams (see Section 10.1c). Each exam should be performed per Section 18.1c. IROC will perform a QC of the imaging exams.

Clinical management and treatment decisions will be made by the treating physician based on local site assessments and other clinical appropriate considerations.
Central review of scans will not be triggered if the study will not be submitted to the FDA for FDA approval of the investigational therapy. Central review of scans will be triggered only if deemed necessary for FDA evaluation. A detailed description of the central radiology PFS review, including image acquisition parameters and image submission instructions, can be found in Section 18.1c.

b. TRIAD Digital Image Submission

TRIAD is the American College of Radiology's (ACR) image exchange application. TRIAD provides sites participating in clinical trials a secure method to transmit DICOM RT and other objects. TRIAD anonymizes and validates the images as they are transferred.

1. TRIAD Access Requirements:

TRIAD will be the sole means of image transfer to the IROC Ohio. TRIAD should be installed prior to study participant enrollment to ensure prompt secure, electronic submission of imaging.

- Site staff who submit images through TRIAD will need to be registered with the Cancer Therapy Evaluation Program (CTEP) and have a valid and active CTEP-IAM account (see Section 13.2).
- To submit images, the site user must be on the site's affiliate rosters and be assigned the 'TRIAD site user' role on the CTSU roster. Users should contact the site's CTSU Administrator or Data Administrator to request assignment of the TRIAD site user role.

2. TRIAD Installations:

After a user receives a CTEP-IAM account with the proper user role, he/she will need to have the TRIAD application installed on his/her workstation to be able to submit images. TRIAD installation documentation can be found by following this link https://triadinstall.acr.org/triadclient/.

This process can be done in parallel to obtaining your CTEP-IAM account username and password.

If you have any questions regarding this information, please send an e-mail to the TRIAD Support mailbox at TRIAD-Support@acr.org.

15.5 S1400GEN Survey Ancillary Study (Optional for Patient)

Ancillary Study to Evaluate Patient and Physician Knowledge, Attitudes, and Preferences Related to Return of Genomic Results in **S1400**.

a. Institutions must seek consent for the Optional Research Survey Study (**S1400GEN**) with in the screening consent forms to submit information for the following ancillary study:

"**S1400GEN** Ancillary Study to Evaluate Patient and Physician Knowledge, Attitudes, and Preferences Related to Return of Genomic Results in **S1400**."

b. Directions:

1. Site staff should download a copy of the Lung-MAP (**S1400GEN**) Appendix 1a Patient Survey and the **S1400GEN** Patient Survey Cover Letter from the protocol abstract page on the SWOG website (www.swog.org) and via the CTSU website and provide a copy of each to study physicians as they prepare to recruit patients to **S1400**. NOTE: The survey and cover sheet is provided to the patient to track the questions that will be asked during the phone interview. The patient may not complete the form other than via the phone interview (unless the study team decides, at a later date, to provide an online Patient Survey option. If the study team does provide an online Patient Survey option, sites will be notified and the online version of the patient survey will be found on the SWOG website mentioned above and be labeled Appendix 1b Patient Survey).

2. Study physicians will provide **S1400GEN** patient participants with a hard copy of the Lung-MAP (**S1400GEN**) Patient Survey and the **S1400GEN** Patient Survey Cover Letter at the time of informed consent. Patient

participation is optional and their care or involvement in the parent trial, **S1400**, will not be affected if they choose not to participate in the **S1400GEN** ancillary study.

3. After patient registration to **S1400GEN**, site staff will submit the patient's contact information found on the last page of the "Optional Research Survey Study (**S1400GEN**)" consent form to the **S1400GEN** study center at the Fred Hutchinson Cancer Research Center (Fred Hutch). Site staff must complete the "For Site Use Only" section prior to submission of the page (documenting the patient's SWOG ID number, initials, the name and contact for the site staff submitting the form and the name and contact information for the physician who enrolled the patient in **S1400**. The site staff must fax the information to <TBD fax number> within 7 calendar days after registration.

4. Fred Hutchinson Cancer Research Center (Fred Hutch) staff will contact the ancillary study patient participants (within 30 days after screening/pre-screening registration) to conduct the 30-minute telephone-based survey (or send them an email link to the survey if it is decided to offer that option). The hard copy of the Lung-Map (**S1400GEN**) Patient Survey, provided at time of consent, will help to improve patient tracking and comprehension during the telephone interview.

5. The 15-minute Lung-MAP (**S1400GEN**) Physician Survey will be administered online. A copy of it will be made available on the SWOG and CTSU webpages.

6. Study staff at Fred Hutch will be responsible for approaching, consenting, and sending a link to the Physician Surveys within 30 days of physicians registering a patient to the patient survey component of this ancillary study.

7. **S1400GEN** study participants (patients and physicians) will be mailed a $20 Amazon gift card to thank them for their time within 30 days of completing the surveys. Study staff at Fred Hutch will be responsible for distributing all survey compensation payments.

Questions regarding ancillary study should be directed to the Study Co-Chair, Dr. Josh Roth at 206-667-7867 or jroth@fredhutch.org.

c. For additional information see Section 18.1f of **S1400**.

16.0 ETHICAL AND REGULATORY CONSIDERATIONS

The following must be observed to comply with Food and Drug Administration regulations for the conduct and monitoring of clinical investigations; they also represent sound research practice:

Informed Consent

The principles of informed consent are described by Federal Regulatory Guidelines (Federal Register Vol. 46, No. 17, January 27, 1981, part 50) and the Office for Protection from Research Risks Reports: Protection of Human Subjects (Code of Federal Regulations 45 CFR 46). They must be followed to comply with FDA regulations for the conduct and monitoring of clinical investigations.

Institutional Review

This study must be approved by an appropriate institutional review committee as defined by Federal Regulatory Guidelines (Ref. Federal Register Vol. 46, No. 17, January 27, 1981, part 56) and the Office for Protection from Research Risks Reports: Protection of Human Subjects (Code of Federal Regulations 45 CFR 46.

Drug Accountability

An investigator is required to maintain adequate records of the disposition of investigational drugs according to procedures and requirements governing the use of investigational new drugs as described in the Code of Federal Regulations 21 CFR 312 and the CTEP Investigator's Handbook.

Publication and Industry Contact

The agents supplied by CTEP, DCTD, NCI used in this protocol are provided to the NCI under Collaborative Agreements (CRADA, CTA, CSA) between the Pharmaceutical Companies (hereinafter referred to as "Collaborators") and the NCI Division of Cancer Treatment and Diagnosis. Therefore, the following obligations/guidelines in addition to the provisions in the "Intellectual Property Option to Collaborator" (http://ctep.cancer.gov/industryCollaborations2/intellectual_property.htm) contained within the terms of award apply to the use of the Agents in this study:

1. Agents may not be used for any purpose outside the scope of this protocol, nor can Agents be transferred or licensed to any party not participating in the clinical study. Collaborators data for Agents are confidential and proprietary to Collaborators and shall be maintained as such by the investigators. The protocol documents for studies utilizing Agents contain confidential information and should not be shared or distributed without the permission of the NCI. If a copy of this protocol is requested by a patient or patient's family member participating on the study, the individual should sign a confidentiality agreement. A suitable model agreement can be downloaded from: http://ctep.cancer.gov.

2. For a clinical protocol where there is an investigational Agent used in combination with (an)other investigational Agent(s), each the subject of different Collaborative Agreements, the access to and use of data by each Collaborator shall be as follows (data pertaining to such combination use shall hereinafter be referred to as "Multi-Party Data"):

   a. NCI will provide all Collaborators with written notice regarding the existence and nature of any agreements governing their collaboration with NCI, the design of the proposed combination protocol, and the existence of any obligations which would tend to restrict NCI's participation in the proposed combination protocol.

   b. Each Collaborator shall agree to permit use of the Multi-Party Data from the clinical trial by any other Collaborator solely to the extent necessary to allow said other Collaborator to develop, obtain regulatory approval or commercialize its own investigational Agent.

   c. Any Collaborator having the right to use the Multi-Party Data from these trials must agree in writing prior to the commencement of the trials that it will use the Multi-Party Data solely for development, regulatory approval, and commercialization of its own Agent.

3. Clinical Trial Data and Results and Raw Data developed under a Collaborative Agreement will be made available exclusively to Collaborators, the NCI, and the FDA, as appropriate and unless additional disclosure is required by law or court order as described in the IP Option to Collaborator (http://ctep.cancer.gov/industryCollaborations2/intellectual_property.htm). Additionally, all Clinical Data and Results and Raw Data will be collected, used and disclosed consistent with all applicable federal statutes and regulations for the protection of human subjects,

including, if applicable, the *Standards for Privacy of Individually Identifiable Health Information* set forth in 45 C.F.R. Part 164.

4. When a Collaborator wishes to initiate a data request, the request should first be sent to the NCI, who will then notify the appropriate investigators (Group Chair for Cooperative Group studies, or PI for other studies) of Collaborator's wish to contact them.

5. Any data provided to the Collaborators for Phase III studies must be in accordance with the guidelines and policies of the responsible Data Monitoring Committee (DMC), if there is a DMC for this clinical trial.

6. Any manuscripts reporting the results of this clinical trial must be provided to CTEP by the Group office for Cooperative Group studies or by the principal investigator for non-Cooperative Group studies for immediate delivery to Collaborators for advisory review and comment prior to submission for publication. Collaborators will have 30 days from the date of receipt for review. Collaborator shall have the right to request that publication be delayed for up to an additional 30 days in order to ensure that Collaborator's confidential and proprietary data, in addition to the Collaborator's intellectual property rights, are protected. Copies of abstracts must be provided to CTEP for forwarding to Collaborators for courtesy review as soon as possible and preferably at least three (3) days prior to submission, but in any case, prior to presentation at the meeting or publication in the proceedings. Press releases and other media presentations must also be forwarded to CTEP prior to release. Copies of any manuscript, abstract and/or press release/media presentation should be sent to:

   E-mail: ncicteppubbs@mail.nih.gov

   The Regulatory Affairs Branch will then distribute them to the Collaborators. No publication, manuscript or other form of public disclosure shall contain any of the Collaborator's confidential/proprietary information.

Monitoring

This study will be monitored by the Clinical Data Update System (CDUS) Version 3.0. Cumulative CDUS data will be submitted quarterly to CTEP by electronic means. Reports are due January 31, April 30, July 31 and October 31.

Trial Master File

This study has the potential for FDA registration; therefore, all participating sites should be FDA "inspection ready". This entails maintaining a Trial Master File that includes essential documents that may be subject to FDA oversight. A list of essential documents is available on the SWOG website under QA/Audits, https://swog.org/Visitors/QA/Index.asp.

Confidentiality

Please note that the information contained in this protocol is considered confidential and should not be used or shared beyond the purposes of completing protocol requirements until or unless additional permission is obtained.

16.1 Adverse Event Reporting Requirements

See Section 16.0 in each of the sub-study appendices (Section 18.2) for sub-study specific adverse event reporting requirements.

17.0 BIBLIOGRAPHY

1 Gandara DR, Li Tianhong, Lara PN Jr., Mack PC, Kelly K, Miyamoto S, et al. Algorithm for codevelopment of new drug-predictive biomarker combinations: accounting for inter-and intrapatient tumor heterogeneity. Clin Lung Cancer. 2012 Jun 5. [Epub ahead of print], 2012.

2 Barker AD, Sigman CC, Kelloff GJ, Hylton NM, et al. I-SPY 2: an adaptive breast cancer trial design in the setting of neoadjuvant chemotherapy. Clin Pharmacol Ther 86(1):97-100, 2009. Epub 2009/05/15. doi: 10.1038/clpt.2009.68. PubMed PMID: 19440188.

3 Kim ES, Herbst RS, Wistuba, II, Lee JJ, Blumenschein GR, Jr., Tsao A, et al. The BATTLE trial: personalizing therapy for lung cancer. Cancer discovery 1(1):44-53, 2011. Epub 2012/05/16. doi: 10.1158/2159-8274.CD-10-0010. PubMed PMID: 22586319.

4 ACS. Cancer Facts and Figures 2013. Available from: http://www.cancer.org/acs/groups/content/@epidemiologysurveilance/documents/document/acspc-036845.pdf.

5 Hammerman PS, Lawrence MS, Voet D, Jing R, Cibulskis et al. Comprehensive genomic characterization of squamous cell lung cancers. Nature. 2012. Epub 2012/09/11. doi: 10.1038/nature11404. PubMed PMID: 22960745.

6 Heger M. TCGA Lung Cancer Study IDs Potential Drug Targets in Majority of Cases. GenomeWeb [Internet]. Available from: http://www.genomeweb.com/sequencing/tcga-lung-cancer-study-ids-potential-drug-targets-majority-cases, 2012.

7 Li t, Kung HJ, Mack PC, Gandara DR. Genotyping and genomic profiling of non-small cell lung cancer: implications for current and future therapies. 10:31(8):1039-49, JCO, 2013.

8 Brahmer J, Reckamp KL, Baas P, et al. N Nivolumab versus Docetaxel in Advanced Squamous-Cell Non-Small-Cell Lung Cancer. N Engl J Med. PMID [PMID: 26028407], 2015.

9 Frampton GM, Fichtenholtz A, Otto GA, et al. Development and validation of a clinical cancer genomic profiling test based on massively parallel DNA sequencing. Nature Biotechnology. Advance online publication. doi: 10.1038/NBT.2696, 2013.

10 Edelman M Spigel D, O'Byrne K, Mok T, Mocci S, Yu W, Paton V, Paz-Ares L. The prevalence of MET expression by immunohistochemistry (IHC) in the MetLung (OAM4971g) trial: a randomized, placebo-controlled, phase III study with erlotinib + onartuzumab (MetMab) vs erlotinib + placebo in patients with previously treated non-small cell lung cancer Journal of Thoracic Oncology 8:S2-S1348, [Abstract MO12.07], 2013.

11 Watermann I, Schmitt B, Stellmacher F, et al: Improved diagnostics targeting c-MET in non-small cell lung cancer: expression, amplification and activation? Diagn Pathol 10:130, 2015.

12 Frampton GM, Fichtenholtz A, Otto GA, et al. Development and validation of a clinical cancer genomic profiling test based on massively parallel DNA sequencing. Nature Biotechnology. Advance online publication. doi: 10.1038/NBT.2696, 2013.

13 Redman MW, Goldman BH, LeBlanc M, Schott A, Baker L. Modeling the relationship between progression-free survival and overall survival: the phase II/III trial. Clin Cancer Res. (in press), 2013

14 Harrington D, Fleming T, Green S. In Crowley J and Johnson RA Eds. Survival Analysis. Hayward, CA: IMS Lecture Notes Monograph Series, 2:269-286, 1982.

15 Redman MW, Goldman BH, LeBlanc M, Schott A, Baker L. Modeling the relationship between progression-free survival and overall survival: the phase II/III trial. Clin Cancer Res 2013;19:xx-xx (in press)

16 Dodd LE, Korn EL, Freidlin B, Gray R, Bhattacharya S. An audit strategy for progression-free survival. Biometrics. ;67(3):1092-9. doi: 10.1111/j.1541-0420.2010.01539.x. Epub 2011 Jan 6. PMID: 21210772, 2011.

18.0 APPENDIX

18.1 **S1400** Appendices

a. Biomarker-Testing Algorithm and Clinical Assays

b. New York Heart Association Classification

c. Risk- Based Monitoring Plan

d. Dose and Administration Schedules Overview

e. Specimen Flow Diagram

f. **S1400GEN** Ancillary Study

18.2 List of Sub-Study Attachments

18.1a Biomarker-Testing Algorithm and Clinical Assays

Biomarker Reporting

Biomarker Screening and Reporting to SWOG
All patients will be screened with the NGS-based assay. Specimens will be sent from the site to Foundation Medicine, Inc. (FMI) using the SWOG Specimen Tracking System. FMI will perform the NGS-based assay. FMI will upload sub-study biomarker eligibility status report and a report containing the complete gene panel results for each patient to a Secure File Transport Protocol (sFTP) site within 16 days after submission of the specimen. An automated process will upload the data into the SWOG database.

Sites will be notified of patient tissue that fails the biomarker screening along with the failure reason by the SWOG Statistical Center within 16 days after submission of the specimen.

Sub-study assignment and Biomarker Reporting for patients screened at progression
Sites will receive the patient's sub-study assignment via email from the SWOG Statistical Center within 1 day from receipt of the biomarker analysis from FMI for patients successfully screened at the time of progression. In addition to containing the sub-study assignment, this email will include the report containing the complete gene panel results and educational material on the interpretation or lack thereof of the biomarker report. The treating physician is responsible for providing this information to the patients.

If at any time it is determined that the patient will not register to a sub-study (either before or after sub-study assignment), the **S1400** Notice of Intention Not to Register form must be submitted. Sites will receive the email containing the sub-study assignment along with the complete gene panel results, as described above, upon receipt of the biomarker analysis from FMI. The treating physician is responsible for providing this information to the patients. Should the decision to not register a patient to their assigned sub-study be reversed, sub-study registration is allowed despite submission of the **S1400** Notice of Intention not to Register form.

Sub-study assignment and Biomarker Reporting for patients pre-screened during current treatment
Sites will receive the patient's sub-study assignment via email from the SWOG Statistical Center within 1 day from receipt of the **S1400** Notice of Progression (provided at least 16 days have passed since the screening registration). The email will include the report containing the complete gene panel results and educational material on the interpretation or lack thereof of the biomarker report. The treating physician is responsible for providing this information to the patients.

If at any time it is determined that the patient will not register to a sub-study (prior to progression or after sub-study assignment), the **S1400** Notice of Intention Not to Register form must be submitted. Sites will receive an email containing the complete gene panel results and educational material, upon receipt of the **S1400** Notice of Intention Not to Register Form. If the **S1400** Notice of Intention Not to Register form is submitted prior to receipt of the complete gene panel results, these results will be provided within 1 day from receipt at the SWOG statistical center. The treating physician is responsible for providing this information to the patients. Should the decision to not register a patient to their assigned sub-study be reversed, sub-study registration is allowed despite submission of the Notice of Intention not to Register form. If the **S1400** Notice of Intention Not to Register Form was submitted prior to progression, submission of the **S1400** Notice of Progression is required to receive the patient's sub-study assignment.

NGS-Based Cancer Gene Test

Assay Description
The next-generation DNA sequencing (NGS) (or massively parallel) assay based on Foundation Medicine's FoundationOne™ assay for cancer gene profiling is a Clinical Trial Assay for this study. It is used to identify genomic alterations in squamous NSCLC on which assignment of patients into a sub-study with the appropriate matched treatment is based. Subjects without specific genomic alterations are eligible for the non-match arm.

The laboratory process described below for the assay is a robust, multi-step protocol to generate targeted deep sequence data from adaptor-ligated sequencing libraries of DNA extracted from FFPE tissue. The workflow is executed in a Clinical Laboratory Improvement Amendments (CLIA)-certified and College of American Pathologists (CAP) accredited laboratory and begins with sample receipt and accessioning into a laboratory information management system (LIMS), incorporates the generation of targeted sequencing data on an NGS platform, and culminates with a customized analysis pipeline capable of detecting genomic alterations with high accuracy. Multiple steps of the process are run on automated liquid handlers to ensure consistency/efficiency. The assay includes process-matched controls in each run, and defined quality control (QC) metrics are used throughout the process to monitor sample and assay performance.

DNA Extraction and Library Construction
A 4 µm section of a hematoxylin and eosin-stained slide is pathology reviewed to ensure a sample volume of ≥ 0.2 mm^3, nucleated cellularity ≥ 80% or ≥ 30,000 cells and that ≥ 20% of the nuclei in the sample are derived from the tumor. A macro-dissection to enrich specimens between 10% ≤ and ≤ 20% tumor content is performed when warranted. DNA is extracted from 40 µm of unstained FFPE sections, typically 4 x 10 µm sections, by overnight digestion in a proteinase K buffer for 8-18 hours followed by purification with the KingFisher Flex with customized extraction kits. Double-stranded DNA (dsDNA) is quantified by a PicoGreen fluorescence assay using the provided lambda DNA standards (Invitrogen). 50-1000 ng of dsDNA in 55 uL water in microTUBEs is fragmented to ~200 bp by sonication (175 sec, 30 % duty, 450 Peak Incident Power, 200 cycles/burst; Covaris LE2200) before purification using a 90 uL volume of AMPure® XP Beads (Agencourt®). Solid-phase reversible immobilization (SPRI) purification containing mixes for end repair, dA addition and ligation, are performed in 96-well plates (Eppendorf) on a Bravo Benchbot (Agilent) using the "with-bead" protocol [1]to maximize reproducibility and library yield. *(1)* Indexed (8 bp barcodes) sequencing libraries are PCR amplified with HiFi™ Hotstart (Kapa) for 10 cycles, 90 uL SPRI purified and quantified Oligreen fluorescence assay (Invitrogen) and sized on a LabChip® GX (Caliper); size selection is not performed. Samples yielding less than 22 ng of extracted DNA or 500 ng of DNA library or less than 140 ng of sequencing library are failed.

Hybrid Selection & Sequencing
Solution hybridization is performed using a > 50-fold molar excess of a pool of individually synthesized 5'-biotinylated DNA 120 bp oligonucleotides (Integrated DNA Technology). The baits target ~1.5 Mb of the human genome including all coding exons of 287 cancer-related genes, 47 introns of 19 genes frequently re-arranged in cancer, plus > 3,500 polymorphisms located throughout the genome. Baits were designed by taking overlapping 120 bp DNA sequence intervals covering target exons (60 bp overlap) and introns (20 bp overlap), with a minimum of three baits per target; SNP targets were allocated 1 bait each. Intronic baits were filtered for repetitive elements as defined by the UCSC Genome RepeatMasker track. *(2)* Hybrid selection of targets demonstrating reproducibly low coverage was boosted by increasing the number of baits for these targets. As described previously, 500-2000 ng of sequencing library is lyophilized in a 96-well plate and suspended in

water, heat denatured at 95°C for 5 minutes and then incubated at 68°C for 5 minutes before addition of the baitset reagent in hybridization buffer. *(3)* After a 24 hour incubation, the library-bait duplexes are captured on paramagnetic MyOne™ streptavidin beads (Invitrogen) and off-target library is removed by washing one time with 1X SSC at 25°C and four times with 0.25 X SSC at 55°C. The PCR master mix is added to directly amplify (12 cycles) the captured library from the washed beads. *(4)* After amplification, the samples are 90 uL SPRI purified, quantified by PicoGreen fluorescence assay (Invitrogen) and sized on a LabChip® GX (Caliper). Libraries are normalized to 1.75 nM and pooled, prior to 49 x 49 paired end sequencing using manufacturer's protocols on the Illumina HiSeq 4,000 system targeting at least 250 x median unique sequence coverage. Any protocol updates will be validated by demonstrating equivalent or superior performance on key metrics, including coverage.

Sequence data processing
Sequence data is mapped to the human genome (hg19) using BWA aligner v0.5.8c. PCR duplicate read removal and sequence metric collection is performed using Picard 1.47 (http://picard.sourceforge.net) and Samtools 0.1.16. *(5, 6)* Local alignment optimization is performed using Genome Analysis Toolkit (GATK) 1.0.4705. *(7)* Variant calling is performed only in genomic regions targeted by the test.

Base substitution detection
Base substitution detection is performed using a Bayesian methodology, which allows detection of novel somatic mutations at low mutant allele frequency (MAF) and increased sensitivity for mutations at hotspot sites through the incorporation of tissue-specific prior expectations. *(8)* Reads with mapping quality < 25 are discarded, as are base calls with quality ≤ 2. The equations governing this evaluation are:

$$P(Mutation\ present\,|\,Read\ data\ "R") = P(Frequency\ of\ mutation\ "F" > 0|R) = 1 - P(F = 0|R)$$

$$P(F = 0|R) = \frac{P(R|F = 0)P(F = 0)}{\sum_{i=0}^{n} P\left(R\middle|F = \frac{i}{n}\right) P\left(F = \frac{i}{n}\right)}$$

$$P(F = 0) = 1 - prior\ expectation\ "p"\ of\ the\ mutation\ in\ cancer\ type$$

$$P\left(F = \frac{i}{n}|i > 0\right) = {}^{p}/_{n}\quad (e.g., n = 100)$$

$$P\left(R\middle|F = \frac{i}{n}\right)$$

is evaluated with a multinomial distribution of the observed allele counts at each candidate mutation site, using empirically observed error rates. Call candidates are issued if $P(F > 0|R) > 99\%$. Final calls are made at MAF ≥ 5% (MAF ≥1% at hotspots) after filtering for strand bias (Fisher's test, p<1e-6), read location bias (KS test, p <1e-6), and presence in 2 or more normal controls.

Indel detection
To detect indels, *de novo* local assembly in each targeted exon is performed using the de-Bruijn approach. *(9)* Key steps are:

- Collecting all read-pairs for which at least one read maps to the target region.
- Decomposing each read into constituent k-mers and constructing an enumerable graph representation (de-Bruijn) of all candidate non-reference haplotypes present.

- Evaluating the support of each alternate haplotype with respect to the raw read data to generate mutational candidates. All reads are compared to each of the candidate haplotypes via ungapped alignment, and a read ‘vote’ for each read is assigned to the candidate with best match. Ties between candidates are resolved by splitting the read vote, weighted by the number of reads already supporting each haplotype. This process is iterated until a ‘winning’ haplotype is selected.
- Aligning candidates against the reference genome to report mutation calls. Indel candidates arising from direct read alignment are also considered.

Filtering of indel candidates is carried out as described for base substitutions above (strand bias p<1e-10, MAF ≥ 3% at hotspots), with an empirically increased MAF thresh-hold at repeats and adjacent sequence quality metrics as implemented in GATK: % of neighboring base mismatches < 25%, average neighboring base quality > 25, average number of supporting read mismatches ≤ 2.

Copy number alteration detection

Copy number alterations (CNAs) are detected using a comparative genomic hybridization (CGH)-like method. First, we obtain a log-ratio profile of the sample by normalizing the sequence coverage obtained at all exons and ~3,500 genome-wide SNPs against a process-matched normal control. This profile is segmented and interpreted using allele frequencies of sequenced SNPs to estimate tumor purity and copy number at each segment. Briefly, if S_i is a genomic segment at constant copy number in the tumor, let l_i be the length of S_i, r_{ij} be the coverage measurement of exon *j* within S_i, and f_{ik} be the minor allele frequency of SNP *k* within S_i. We seek to estimate p, tumor purity, and C_i, the copy numbers of S_i. We jointly model r_{ij} and f_{ik}, given p and C_i: $r_{ij} \sim N(\log_2 \frac{p*C_i+(1-p)*2}{p*(\sum_i l_i C_i)/\sum_i l_i+(1-p)*2}, \sigma_{ri})$ and $f_{ik} \sim N(\frac{p*M_i+(1-p)}{p*C_i+(1-p)*2}, \sigma_{fi})$, where M_i is the copy number of minor alleles at S_i, distributed as integer $0 \le M_i \le C_i$. σ_{ri} and σ_{fi} reflect noise observed in the CGH and SNP data, respectively. Fitting is performed using Gibbs sampling, assigning absolute copy number to all segments. Model quality is reviewed and alternative explanations considered[9], and amplifications are called at segments with ≥6 copies (or ≥ 7 for triploid/≥ 8 for tetraploid tumors) and homozygous deletions at 0 copies, in samples with purity ≥ 20%. *(10)*

Gene fusions.

Genomic rearrangements are identified by analyzing chimeric read pairs (read pairs for which reads map to separate chromosomes, or at a distance of over 10 Mbp). Pairs are clustered by genomic coordinate of the pairs, and clusters containing at least five chimeric pairs (3 known fusions) are identified as rearrangement candidates. Filtering of candidates is performed by mapping quality (MQ>30) and distribution of alignment positions (standard deviation > 10). Rearrangements are annotated for predicted function (e.g., creation of fusion gene).

<u>Laboratory</u>

Clinical sample testing is done centrally at Foundation Medicine (FMI)'s CAP-accredited/CLIA-certified diagnostic laboratory (Cambridge, MA).

<u>Tumor Scoring</u>

Positivity for each mutation for sub-study eligibility will be defined as presence of an alteration call by the methods above, with the list of eligible alterations specified in Section 5.0 of each sub-study. The estimated mutant allele frequency, read depth, or copy level are recorded for each alteration, as appropriate. The definition of eligible alterations remains fixed for the duration of each sub-study. Any analysis software updates are subject to a Good Clinical Practice (GCP)-compliant change control process (available upon request), which must demonstrate equivalent or superior accuracy of alteration calls. *(11)*

<u>Validation</u>

The assay platform is commercially available. Validation studies were conducted on previously characterized cancer cell lines known to contain defined sets of genomic alterations in line with guidelines established by the Next Generation Sequencing: Standardization of Clinical Testing work group. *(12)* The NGS assay was found to be highly accurate in identifying genomic alternations, with sensitivity >99% for detection of base substitutions in samples in which as few as 5% of the nuclei were derived from cancer cells containing the alterations, 98% for detection of insertions and deletions in samples in which as few as 10% were derived from cancer cells containing the alterations, and > 95% for detection of copy number alterations in which as few as 30% of the nuclei were derived from cancers cells containing the alterations. In aggregate, the positive predictive value was 99% in the validation study. The validation procedure is discussed in detail in the attached publication with supplementary information. *(13)*

REFERENCES

1 Fisher S, et al. A scalable, fully automated process for construction of sequence-ready human exome targeted capture libraries. Genome Biol 12, R1, 2011.

2. Karolchik D, et al. The UCSC Table Browser data retrieval tool. Nucleic Acids Res 32, D493-496, 2004.

3. Gnirke A, et al. Solution hybrid selection with ultra-long oligonucleotides for massively parallel targeted sequencing. Nat Biotechnol 27, 182-189, 2009.

4 Van Loo P, et al. Allele-specific copy number analysis of tumors. Proc Natl Acad Sci U S A 107, 16910-16915, 2010.

5. Li H, Durbin R. Fast and accurate long-read alignment with Burrows-Wheeler transform. Bioinformatics 26, 589-595, 2010.

6. Li H, et al. The Sequence Alignment/Map format and SAMtools. Bioinformatics 25, 2078-2079, 2009.

7. DePristo MA, et al. A framework for variation discovery and genotyping using next-generation DNA sequencing data. Nat Genet 43, 491-498, 2011.

8. Forbes SA, et al. COSMIC: mining complete cancer genomes in the Catalogue of Somatic Mutations in Cancer. Nucleic Acids Res 39, D945-950, 2011.

9 Compeau PE, Pevzner PA, Tesler G. How to apply de Bruijn graphs to genome assembly. Nat Biotechnol 29, 987-991, 2011.

10 Gargis AS, Kalman L, Berry MW, Bick DP, et al. Assuring the quality of next-generation sequencing in clinical laboratory practice. Nat Biotechnol 30:1033-1036, 2012. Pubmed PMID: 23138292

11 Gargis AS, Kalman L, Berry MW, Bick DP, et al. Assuring the quality of next-generation sequencing in clinical laboratory practice. Nat Biotechnol 30:1033-1036, 2012. Pubmed PMID: 23138292.

12 Gargis AS, Kalman L, Berry MW, Bick DP, et al. Assuring the quality of next-generation sequencing in clinical laboratory practice. Nat Biotechnol 30:1033-1036, 2012. Pubmed PMID: 23138292.

13 Frampton GM, et al. Development and validation of a clinical cancer genomic profiling test based on massively parallel DNA sequencing. Nat Biotechnol 31, 1023-1031, 2013.

18.1b New York Heart Association Classification

Class	Cardiac Symptoms	Need for Limitations	Physical Ability Additional Rest*	To Work**
I	None	None	None	Full Time
II	Only moderate	Slight or occasional	Usually only slight	Usually full time
III	Defined, with less than ordinary activity	Marked	Usually moderate	Usually part time
IV	May be present even at rest, & any activity increases discomfort	Extreme	Marked	Unable to work

* To control or relieve symptoms, as determined by the patient, rather than as advised by the physician.

** At accustomed occupation or usual tasks.

18.1c Risk Based Monitoring Plan

On Site Auditing

NCI guidelines for Auditing Clinical Trials for the National Clinical Trials Network (NCTN) Program, Community Clinical Oncology Program (CCOP)/NCI Community Oncology Research Program (NCORP) and Research Bases: http://ctep.cancer.gov/branches/ctmb/clinicalTrials/docs/ctmbauditguidelines.pdf.

The Quality Assurance Program of the Groups participating in the NCTN was developed to enhance the reliability and validity of clinical trials data through the use of routine monitoring procedures which includes auditing as one component. The purpose of an audit is to document the accuracy of data submitted to the Data Operations Center and to verify compliance with protocol and regulatory requirements. The program also surveys data management practices at each institution in order to provide educational support to the sites regarding issues related to data quality, data management, and other aspects of quality assurance.

Each institution is audited at least once every three years, but remains at annual risk of an audit. Routine monitoring of Institutional Performance Review reports and timeliness of reporting of Serious Adverse Events is conducted to identify institutions that may require more frequent audits.

The audit team consists of qualified individuals capable of providing a medical assessment of the patient cases (Quality Assurance representative from the research base, physician, nurse or experienced clinical research associate [CRA]). A number of patients equal to 10% of the accrual since the last audit with a minimum of three are randomly selected for review at each institution. In addition, a limited review of eligibility and consent only is conducted for at least one unannounced case at each on site audit.

The major objective of the audit process is to verify study data that could affect the interpretation of primary study endpoints. This is done through independent verification of study data against the source documents. Primary source documentation reviewed during an audit includes the following: research records, hospital charts, clinic charts, lab reports, x-rays, scans, radiotherapy reports, operative reports, pathology reports and other special studies required by protocol.

By comparing the data collection forms submitted to each Groups' Data Operations Center with the primary records and referring to the protocol, the audit team reviews the records to determine compliance with protocol requirements for eligibility, treatment administration, response assessment, toxicity reporting and general data quality. NCTN investigators and institutions are expected to follow the protocol and lead Group policy in treating patients registered on Group protocols. Among other requirements, investigators/institutions must follow Group's dosing principles requirements for reporting Serious Adverse Events, and follow-up of all patients.

The audit team verifies that the protocol and its amendments received initial and continuing IRB review and approval and that safety reports and serious adverse events were submitted to the IRB. Investigational drug accountability record forms (DARFs) are reviewed and random patients are cross referenced against the medical record. A tour of the pharmacy is conducted to verify security and storage conditions as well as the physical inventory. Auditors also verify that the current IRB-approved version of the consent form was signed prior to registration and that subjects were informed of new findings that could affect their willingness to participate in the study.

The audit report is comprised of three components: 1) conformance to IRB and informed consent requirements, 2) the pharmacy and use of NCI DARFs, and 3) patient case review. An acceptable rating requires no deficiencies, few lesser deficiencies, or major deficiencies

that were addressed prior to the audit. Institutions found to be "unacceptable" or "acceptable, but requires “follow-up” on any component are required to submit a written response and/or corrective and preventative (CAPA) action plan. Failure to submit a written response including a corrective and preventative action plan within the required timeframe will result in suspension of registration privileges. A re-audit of any component rated as unacceptable will be conducted within one year after the unacceptable audit. An unacceptable rating for the same audit component on two consecutive audits will result in probation. Accrual will be suspended pending submission of a site improvement plan that addresses key infrastructural issues contributing to poor performance. An unacceptable rating at the second re-audit may result in termination from the group. If systematic misrepresentation of data is identified, an immediate repeat audit is scheduled by the representatives from the Group with the NCI and/or the FDA present.

In some cases, non-compliance for issues such as timeliness of data submission, SAE reporting and submission of specimens is monitored off site rather than scheduling a re-audit. Failure to show improvement may result in scheduling of a re-audit or other disciplinary action.

Results of all Quality Assurance Audits are reported to the NCI, the Principal Investigator of the institution that was audited, and representative of the Group. Protocol specific audit results are also sent to the Lead Group to inform the statisticians, data chairs and study chairs of all discrepancies involving eligibility, treatment, toxicity or response assessment.

The Quality Assurance Program performs their educational role through several mechanisms including presentations during the Group Meetings online Clinical Trials Training Courses, collaboration with others such as Pharmacy Committees and Statistical Centers to develop training tools, and memos and newsletter articles that are distributed to all Group institutions to educate research staff about changes in regulatory and quality assurance issues and audit procedures.

<u>On Site Monitoring</u>

In addition to the standard auditing process outlined above, the following additional requirements will be implemented for this study:

- First on-site monitoring visit at each institution within 3 months of first patient registration to a sub-study.
- On site monitoring for all sites with patients registered to a sub-study twice per year. Additional monitoring visits to a site will be scheduled in response to several factors - rate of accrual, previous monitoring visit results, centralized electronic monitoring outcome, change in staff, etc.
- An exception to the onsite monitoring requirement will be allowed in the following circumstances:
  - Sites that use a centralized pharmacy and data management team may be monitored at this central location.
  - Sites that had an acceptable pharmacy audit in the last year may be audited off site at a central location.
  - Sites that had an acceptable patient case review outcome at their last audit and have not enrolled patients to any new sub-studies may be put on an annual schedule.

Two full time monitors with 100% effort to **S1400** are in charge of conducting monitoring visits. Additional monitoring responsibilities are shared by the SWOG audit team members at the Operations Office and Statistical Center. Additional monitoring visits may be sub-contracted with other Network Groups, if necessary.

Centralized Data Coordinator Monitoring at the SWOG Statistics and Data Management Center

The SWOG Statistics and Data Management Center (SDMC) will support the risk based monitoring approach for this trial with the following actions:

- Monitor data quality through routine review of submitted data such as on-study, baseline and follow up tumor assessment, lab, treatment, off treatment, and follow up case report forms to identify and follow-up on missing data, inconsistent data, data outliers, and potential protocol deviations that may be indicative of systemic or significant errors in data collection and reporting at a site.

- Analyze site characteristics, performance metrics and clinical data to identify trial sites with characteristics correlated with poor performance or noncompliance through the SWOG Institutional Performance Reporting mechanism and other available reports.

- Verify critical source data remotely via the collection and review of pathology, radiology and applicable lab reports. This includes the review and confirmation of appropriate disease classification as determined by the pathology report, and assessment of response to treatment utilizing RECIST 1.1 based on scan reports uploaded to the Electronic Data Capture (EDC) system and submitted follow-up tumor assessment forms.

- To assure data are as consistent, complete and accurate as possible, all subject data must undergo careful review by Data Coordinators (DCs). After verifying that all data forms required to determine eligibility have been received or at a time point designated when all the required forms should have been received, the DC reviews the data and completes an initial evaluation.

The initial review includes the following:

- Determine that all required data fields on each form were completed and are consistent with other data.
- Determine if all prestudy tests and exams were performed within protocol specified time limits.
- Determine if each eligibility criterion was met and properly documented.
- Review and confirm pathology based on the pathology report uploaded to the EDC system.
- Verify that stratification and/or descriptive factors (if applicable) were correctly identified at registration.
- Verify that the subject received the assigned study treatment and correct dose(s).
- Verify that the treatment was started within the time limit indicated in the protocol (if applicable).
- Determine if adverse events reported are consistent with other data and entered as required by study specifications.

- Post internal notes to add additional information which may be useful to the study sponsor, monitors, or statisticians, but which do not require action by site personnel.
- Use the query tool to request additional data classifications and corrections of the CRA.

The DC will perform subsequent review of data when new data become available or queries are answered. Regular review will also occur while patients are still on-study, at the time of progression, once they are removed from study and at the time of death.

Subsequent reviews include the following:

- Determine if all required data fields on each newly submitted form were completed and consistent with other data.
- Evaluate all new treatment documentation for correct treatment and dose.
- Conduct assessment of response to treatment utilizing RECIST 1.1 based on scan reports uploaded to the EDC and submitted follow-up tumor assessment forms.
- Review and code any new concomitant medications as required by study specifications.
- Evaluate if the subject is or should be off protocol treatment per protocol criteria.
- Review and evaluate death if death of subject is reported.
- Use the query tool to request additional data classifications and corrections.
- Post internal query notes to add additional information that may be useful to the study sponsor, monitor, or statisticians but which do not require action by site personnel.
- Review site responses to the queries and the corrected or amended eCRF pages. When corrections and responses are considered satisfactory, queries are closed by the data coordinators. Unsatisfactory responses are re-queried and tracked.
- Perform re-evaluations promptly after responses to queries are received.

Centralized Monitoring Review by the SDMC Monitors (Implemented as of Revision # 9)

Off-site monitoring includes auditable elements for the Screening/Pre-Screening Registration for the first two patients registered to **S1400** at each new study site or sites where an on-site **S1400** audit has not occurred.

Within one month after the **S1400** Registration, a SDMC monitor will email the head CRA at the study site with the instructions for uploading auditable elements for the remote review. Site staff are to upload source documents to support the Eligibility Criteria (Section 5.0) and applicable forms submitted in Rave® for the Screening/Pre-Screening Registration. The review will also consist of timeliness of data submission, specimen collection and specimen submission.

Required Documents for Uploading to Rave®:
Screening/Pre-Screening Informed Consent Form Pages:

- Title Page
- Response to Future Contact question
- Responses to Samples for Future Research Studies questions
- Signature Page

Eligibility:

- **S1400** Registration Worksheet (signed and dated by the Registering Investigator)
- Source Documents to Support Section 5.0 Eligibility Criteria
- Source Documents to Support the **S1400** Onstudy Form
- Source Documents to Support Other Forms if applicable (Examples: Notice of Progression, Status Update, Notice of Death, etc.)

Safety Specific Centralized Monitoring

Each Serious Adverse Event (SAE) report submitted (via National Cancer Institute systems CTEP-AERS) will be reviewed by the SWOG SAE Coordinator. For each report supporting documentation will be requested and compiled with the report and sent to the Physician Reviewer. As mentioned below all sites will undergo mandatory training and this will include training regarding SAE reporting. SWOG regularly monitors timeliness of SAE reporting and addresses any issues of poor performance with individual sites.

The study will be monitored for under reporting/missed Serious Adverse Events: The SWOG SAE Coordinator receives a weekly report from the data base that includes all adverse events that are submitted through routine submission that potentially also meet expedited reporting criteria but for which no CTEP-AERS report is found. The Coordinator is responsible for following up with the responsible site to ensure that SAEs are not missed/under-reported.

The study will be monitored for trends in Serious Adverse Events: A “new SAE on study” report is generated each time a new Serious Adverse Event is entered into the SWOG data base. It is a cumulative report that lists all SAEs reported for the protocol. This allows those who review the report to identify concerning trends in reported events; events that may be occurring at greater intensity (higher toxicity grade) or frequency than expected. The SAE Coordinator and Physician Reviewer are responsible for regularly monitoring this report as well as the Study Chair, and assigned Statisticians.

Additional Approaches to be Used

- Mandatory training of key site personnel prior to first patient registration.
- Timely review of all monitoring reports to identify sites that require additional training, monitoring, disciplinary action, etc.
- Routine monthly communication between monitor and site staff to assess potential problem areas, provide feedback, identify staff turnover, etc.
- Additional mandatory centralized training to be provided to all sites if major changes to the protocol occur or common problem areas are identified.

Blinded Independent Central Review of Response and Progression-Free Survival Endpoint

All participants will undergo serial CT or MRI imaging: baseline/pre-treatment and every 6 weeks until progression of disease (as determined by local site assessment).

Collection and storage of all images as well as management of the independent review process will be the responsibility of the National Cancer Institute’s (NCTN) Imaging and RT Quality Assurance Service Core (IROC).

The same imaging modality MUST be used for an individual patient throughout the course of the trial, with the exception of a PET/Spiral CT is used at baseline where the CT is of diagnostic quality, follow-up scans can be done by a spiral CT or if a PET/conventional CT is used at baseline where the CT is of diagnostic quality, follow-up scans can be done by conventional CT. No other methods of assessments are interchangeable. The pre- and post-treatment CT or MRI images must be submitted to IROC for all study participants. CT is the preferred imaging modality unless there is a medical contraindication.

Clinical management and treatment decisions will be made by the treating physician based on local site assessments and other clinically appropriate considerations. The study will collect and archive 100% of scans for randomized patients through the time of confirmed

progression. These will be ready for independent review should a sub-study terminate early for a large effect on PFS or ORR.

At the time that central review is indicated, SWOG will provide IROC with the registration date and patient number, along with a blinded code indicating which reviewer will be assigned to each case. This ensures that each reader reviews a balanced number of cases from each study arm. An experienced team of radiologists will be identified by IROC. A designated radiologist will review each image for assessment of progression of disease per RECIST 1.1 criteria. The same reviewer will read all study images of an individual study participant. Data from local reviews will not be provided to the central reviewers in order to keep the central reviewers blinded to the results of the local investigator-assessed PFS.

The actual progression will be determined at the SWOG Statistical Center, using a composite of the IROC assessment, in conjunction with possible data on progression or symptomatic deterioration (see Section 10.7). Results of the central review will NOT be communicated to the local site. Decisions regarding clinical management of the patient will be made by the treating physician based on local site assessments/reviews and other clinical considerations.

The BICR audit analysis will proceed following the algorithm specified in Dodd et.al 2011. *(1)* Specific details for the inputs of this algorithm are described in Section 11.5.

Review of pre-treatment CT/MRI exam will be performed as follows: The reader will review all anatomic areas (chest, abdomen) imaged and available. The target lesions to be evaluated will be defined/determined by the review of the pre-treatment exam. A maximum of 5 target lesions will be defined with a maximum of 2 in a single organ. The 5 target lesions will be chosen with representation from all organs involved with the tumor. Additional significant non-target lesions and areas of non-measurable disease will be noted. A screen capture of each target lesion, annotated with a pointer and a lesion reference number assigned to the target lesion, will be generated and archived. This will be used on subsequent reads to ensure concordance of lesions on follow-up/post-treatment exams. Measurements will be made from the axial scan (generally the post contrast scan) that best demonstrates the lesion as distinct from background. All measurements will be made by electronic calipers. A screen capture of the actual measurement axis with calipers will be saved and archived with the exam permanently at IROC.

Review of post-treatment image exam(s) will be performed as follows: The pre-treatment annotated exam and CRF will be reviewed to ensure lesion concordance. Readers will review all images for the current time point prior to making measurements. All measurements will be made by electronic calipers. A screen capture of the actual measurement axis with calipers will be saved and archived with the exam permanently at IROC.

1. Communication of Monitoring Results

   The monitoring team will meet monthly to share all aspects of monitoring (on sites, centralized, safety, alternative). When needed, the SWOG Executive Officer for Quality Assurance will be consulted.

   Routine site audits will be reported according to NCI Clinical Trials Monitoring Branch requirements. Additional monitoring visits (those performed at greater frequency than required by the Clinical Trials Monitoring Branch) will be maintained in the CTMB-AIS data base and regularly reviewed by SWOG monitoring staff.

Summarized results of all monitoring visits will be provided annually to the study's Data and Safety Monitoring Committee, the SWOG Board of Governors, the study team and the FDA.

2. Management of Noncompliance

Issues of particular concern related to patient safety and questions of site fraud will be managed according to SWOG standard policies and the policies of the NCI Clinical Trials and Monitoring Branch for auditing of clinical trials under the NCI National Clinical Trials Network (NCTN) Program.

Where important deviations are discovered, additional site training components will be developed and implemented.

As with standard NCTN procedures, sites will be required to develop and implement corrective action plans in response to any deficiencies identified at a monitoring visit.

3. Ensuring Quality Monitoring

All staff involved in monitoring are required to undergo training in the principles of clinical investigations and human subject's protection. They are also required to complete the same protocol specific training required of the site staff.

All monitoring and auditing process for the study will be reviewed by study leadership twice per year to ensure conformance to the monitoring plan.

4. Monitoring Plan Amendments

At each formal review of the monitoring plan and conformance to it, the study leadership will make a recommendation regarding the need for amendments to the monitoring plan. These amendments will be reviewed and approved by NCI and provided in this protocol section and will be submitted to the FDA.

REFERENCES

1 Harrington D, Fleming T, Green S. In Crowley J and Johnson RA Eds. Survival Analysis. Hayward, CA: IMS Lecture Notes Monograph Series, 2:269-286, 1982.

18.1d Dose and Administration Schedules Overview

1.0 List of Sub-studies

Table 1.1 Sub-studies Open to Accrual

Sub-study	Investigational Agent(s)	Target	Manufacturer	Structural Class	Dose	Route	Day	Cycle	Control	Activation Date
**S1400I**	Nivolumab + Ipilimumab	Anti-PD-Linhibitor, CTLA-4 inhibitor (non-match arm)	Bristol-Myers Squibb	Monoclonal antibody (CD279)	3 mg/kg 1 mg/kg	IV (Over 30/60 minutes)	1	14 days/42 days (q 3 cycles)	Nivolumab	12/18/15
**S1400G**	Talazoparib	PARP inhibitor	Medivation	Small molecule	1000 mcg	PO	1-21	21 days	N/A	02/07/2017
**S1400F**	MEDI4736 + Tremelimumab	Anti-PD-1/PD-L1 Inhibitor Resistant	Astra Zeneca	monoclonal antibody (1gG1, 1gG2)	1500 mg 75 mg	IV (Over 60 minutes)	1	28 days (Treme – Cycle 1-4 only)	N/A	10/2/2017

[a] Arm 2 – The control arm Docetaxel has been closed to accrual with Revision #3

Table 1.2 Sub-studies Closed to Accrual

Sub-study	Investigational Agent(s)	Target	Manufacturer	Structural Class	Dose	Route	Day	Cycle	Control	Closure Date
**S1400A**	MEDI4736	PD-L1 inhibitor (non-match arm)	MedImmune, Inc.	Monoclonal antibody (IgG1κ)	10 mg/kg	IV (over 60 minutes)	1	14 days	N/A [a]	12/18/15
**S1400B**	GBC-0032	PI3K inhibitor	Genentech	Small molecule	4 mg QD	PO	1-21	21 days	N/A [a]	12/12/16
**S1400C**	Palbociclib	CDK4/6 inhibitor	Pfizer, Inc.	Small molecule	125 mg QD	PO	1-21, followed by 7 days off	28 days	N/A [a]	09/01/16
**S1400D**	AZD4547	FGFR1/2/3 inhibitor	Astra Zeneca	Small molecule	80 mg BID	PO	1-21	21 days	N/A[a]	10/31/16
**S1400E**	Rilotumumab + Erlotinib	HGF/c-MET inhibitor	Amgen	Monoclonal antibody (IgG2)	15 mg/kg	IV	1	21 days	Erlotinib	11/25/14

[a] Arm 2 – The control arm Docetaxel has been closed to accrual with Revision #3

2.0 Protocol Instructions for Addition and Release of Investigational Agents

The protocol is structured to enable seamless addition and release of investigational agents over the course of the trial.

When an investigational agent is added or released from use in this trial, the following will require updating or addition:

- SCHEMA
- Section 18.1d: Overview of all investigational agents/sub-studies. Each investigational agent falls into one of two categories: 1) Activated Sub-studies, and 2) Inactivated Sub-studies.
- Section 18.2: List of individual sub-study protocols. As investigational agents/sub-studies are added to the trial, they will be listed in alphabetical order. Sub-study-specific Model Treatment Consent

Adding a Sub-study to the Trial
The procedure for adding a new sub-study depends on its readiness. If all requirements have been met for activation (drug supply, contracts, etc.), a one-step process is followed. If activation is pending fulfillment of one or more requirements for activation, a two-step process is followed. For example, agent supply may not yet be available for distribution, although all other requirements have been addressed.

If activation of a new sub-study is pending, the protocol is amended to include: 1) the new sub-study section 2) a separate, sub-study-specific treatment consent, and 3) an updated Section 18.1d showing the new agent in Table 1.1 (Activated Sub-studies). The protocol amendment will be considered a major modification to the protocol, requiring CTEP amendment review, full IRB committee review; however, assignment to the already active sub-studies is not affected. Assignment to the new sub-study at an individual site may begin after IRB approval has been obtained.

If all requirements for activation of a new sub-study have already been met, the protocol is amended to include: 1) the new sub-study section, 2) a separate sub-study treatment consent, 3) an updated Section 18.1d showing the new agent in Table 1.1 (Activated Sub-studies) 4) and the new sub-study in the SCHEMA. The protocol amendment will be considered a major modification to the protocol and will require CTEP amendment review and full IRB committee review. Assignment to the sub-study at an individual site can begin after IRB approval has been obtained.

Releasing a Sub-study from the Trial
When an investigational agent/sub-study is closed to accrual, a protocol administrative change will be generated that includes: 1) an updated Section 18.1d showing the sub-study moved from Table 1.1 (Sub-studies Open to Accrual) to Table 1.2 (Sub-studies Closed to Accrual), 2) a watermark ("**Closed Effective** XX/XX/XXXX ") on the sub-study section, which will remain in the protocol, 3) a watermark ("**Closed Effective** XX/XX/XXXX") on the separate, sub-study-specific treatment consent, and 4) removal of the sub-study from SCHEMA in **S1400**. Assignment to the active sub-studies is not affected.

18.1e Specimen Flow Diagram

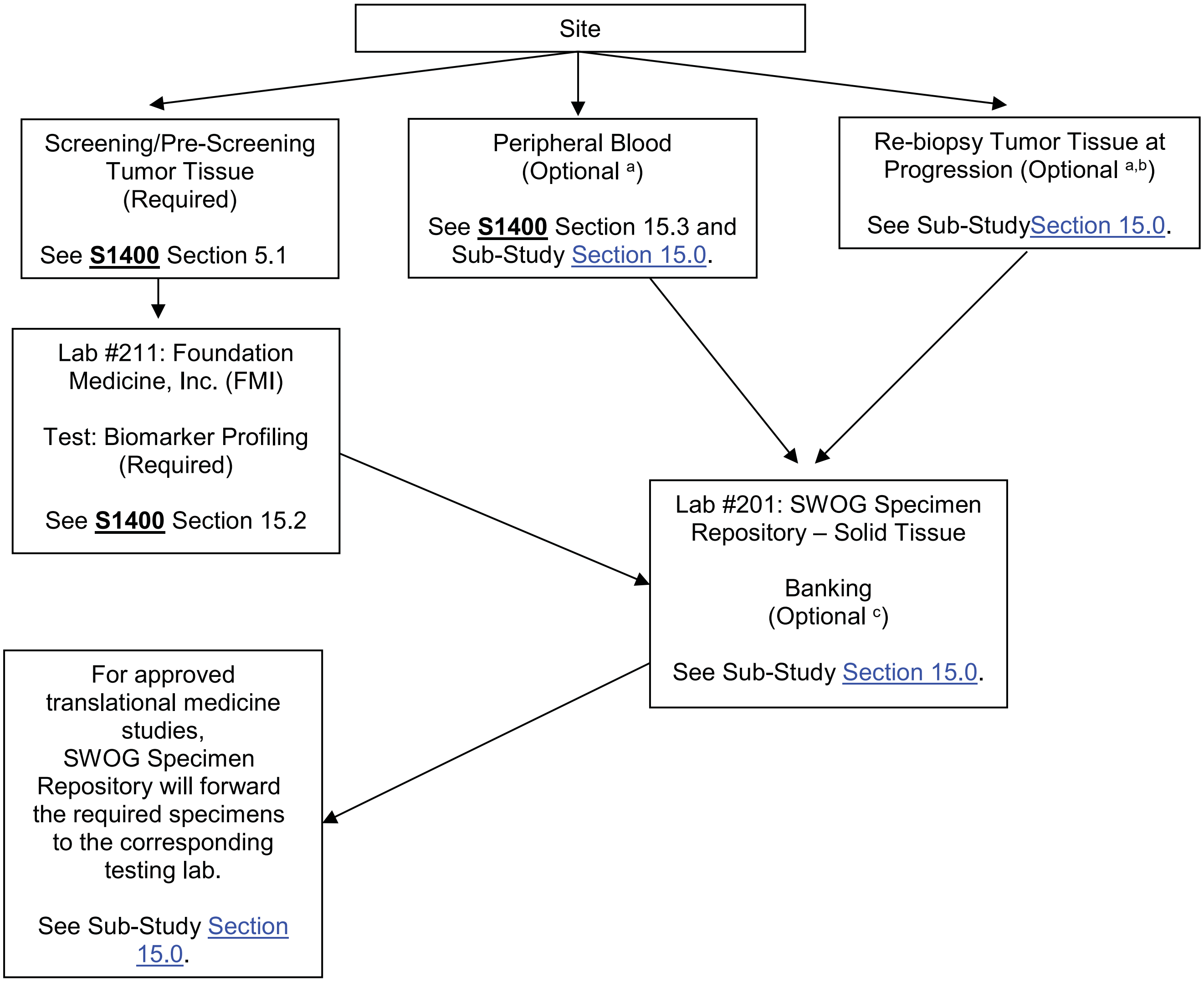

a With patient's consent.

b Among patients who initially responded to protocol treatment.

c Remaining tissue will be sent to the SWOG Specimen Repository-Solid Tissue, Myeloma and Lymphoma Division, Lab #201, for use of the Translational Medicine studies within any sub-study the patient is enrolled in. SWOG Specimen Repository will prepare and ship the required specimens to the appropriate laboratory. The specimen will be kept until there are no additional sub-studies for the patient to enroll in or the tissue is used up, whichever happens first. With patient’s consent, any leftover tissue will remain at the SWOG Repository for future exploratory analysis.

18.1f **S1400GEN**

Ancillary Study to Evaluate Patient and Physician Knowledge, Attitudes, and Preferences Related to Return of Genomic Results in **S1400**

STUDY CHAIRS:

Scott Ramsey MD, PhD
Joshua A. Roth, PhD, MHA
Hutchinson Institute for Cancer Outcomes Research
1100 Fairview Avenue North
Seattle, WA 98109
Phone: 206-667-7846
FAX: 206-667-5977
E-mail: sramsey@fredhutch.org
jroth@fredhutch.org

Dawn L. Hershman, MD, MS
Katherine Crew, MD, MS
Herbert Irving Comprehensive Cancer Center
Columbia University
161 Fort Washington, 1068
New York, NY 10032
Phone: 212 305-1945
FAX: 212 305-0178
E-mail:dlh23@cumc.columbia.edu
kd59@cumc.columbia.edu

The purpose of this exploratory pilot study is to evaluate patient and physician knowledge, attitudes, and preferences about genomic profiling findings in the SWOG 1400 (**S1400**) study. This data will be gathered using a one-time only survey. The survey questions are principally focused on evaluating patient attitudes and preferences about return of incidental somatic mutation findings suggestive of a germline mutation in the context of a biomarker-driven clinical trial. Additionally, the study evaluates patient and physician knowledge of the differences between germline and somatic mutations, and physician attitudes and preferences about return of incidental genomic findings. The results of this study will be used to generate hypotheses for future research regarding return of genomic results in biomarker-driven clinical trial settings.

SCHEMA

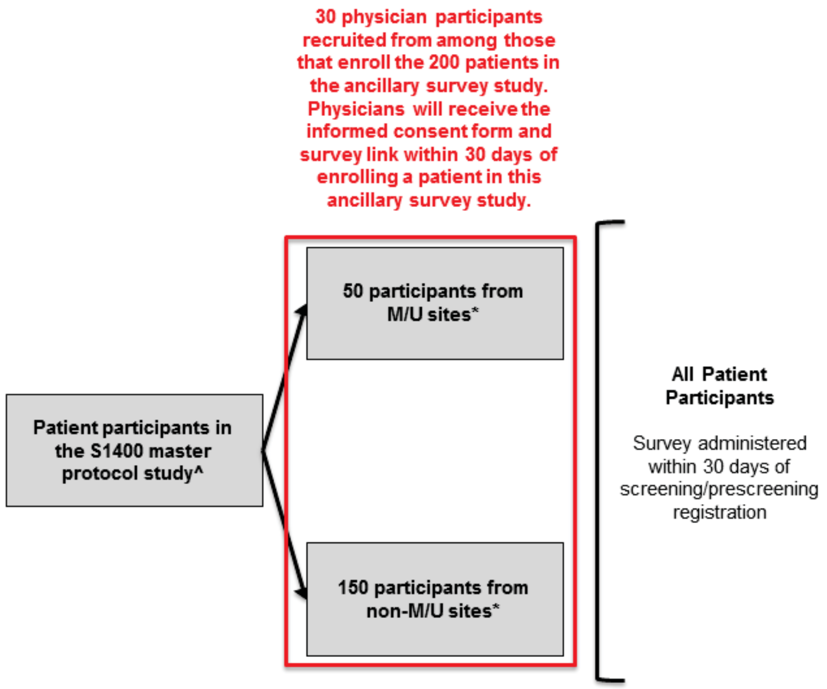

NCORP=NCI Community Oncology Research Program, M/U=Minority/Underserved

^Consent for this study is included in the S1400 master protocol screening and pre-screening consent forms.
*Accrual will continue until a minimum of 100 patients are enrolled in the ancillary study.

1.0 OBJECTIVES

1.1 Primary Objective

To evaluate patient attitudes and preferences about return of somatic mutation findings suggestive of a germline mutation in the **S1400** master protocol trial.

1.2 Secondary Objective(s)

a. To evaluate **S1400** study physician attitudes and preferences about return of somatic mutation findings suggestive of a germline mutation in the **S1400** master protocol trial.

b. To evaluate **S1400** patients' and study physicians' knowledge of cancer genomics.

c. To evaluate **S1400** patients' and study physicians' knowledge of the design of the **S1400** master protocol trial.

d. To explore whether physician and patient knowledge of cancer genomics and attitudes and preferences about return of genomic profiling findings are correlated.

2.0 BACKGROUND

Rationale

'Master protocol' or 'biomarker-driven' trials (hereafter referred to as 'biomarker-driven') that are guided by genomic profiling results are rapidly increasing in number, and may represent the beginning of a new paradigm in clinical trial research in oncology. *(1,2)* Because these types of trials involve many independently conducted sub-studies that may involve different eligibility criteria and treatments, they are particularly complex to design and communicate. *(3,4)* As such, there is a threat that patients and study physicians participating in the initial genomic screening portion of biomarker-driven trials may not be fully informed about the many nuances of the return of genomic results, relationships between genomic results and eligibility for participation in sub-studies, or alternative treatment options (or lack thereof) within sub-studies. Furthermore, patient knowledge and attitudes about the return of genomic profiling results remains poorly studied outside the scope of a given clinical trial (hereafter referred to as "incidental findings"), and protocols for return of incidental findings in clinical trial settings are not established as they are in clinical practice. *(5)* Several studies have evaluated patient knowledge, attitudes, and preferences about genomic profiling in clinical care, but no studies have investigated these issues in the context of a biomarker-driven clinical trial. *(6,7,8,9,10)* As a result, there is urgent need to develop understanding of patient and study physician perspectives on genomic testing in clinical trials to: 1) evaluate if they have sufficient understanding of genomic principles to discuss the implications of participation in such trials, 2) improve return of genomic result processes in ongoing trials, and 3) inform the design of future biomarker-driven trials.

SWOG **1400** (hereafter referred to as '**S1400'**) is an example of an ongoing biomarker-driven clinical trial where the issues outlined above are particularly pertinent. **S1400** is a phase II/III screening/clinical registration protocol that involves genomic profiling of patients with advanced stage squamous cell lung cancer to direct patients to sub-studies investigating targeted (or 'matched') and 'non-matched' therapies *(11,12)* These study characteristics create an ideal setting to study patient and physician knowledge, attitudes, and preferences about genomic findings because: 1) patients and study physician knowledge of the genomic testing procedures and return of results can be evaluated in a natural setting; 2) the Foundation Medicine next-generation sequencing platform used in the study has the ability to detect incidental genomic findings (i.e. genetic alterations not directly related to decisions in the **S1400** trial), such as the 56 recommended by the American College of Medical Genetics3--making study participants uniquely able to provide insights about attitudes and preferences related to incidental findings based on their own experience and feelings; and 3) study patients have poor survival prognosis, so they are in a natural position to reflect on the important issue of preferences for return of genomic testing results to family members in the event that they die prior to receiving the results themselves. *(13,14)* Furthermore, many organizations and individual researchers have noted the need for studies evaluating these issues both in cancer patients in general, and in cancer patients in clinical trial settings. *(15,16,17)*

We propose to conduct an ancillary survey study focused on patient and physician knowledge, attitudes, and preferences about somatic mutation findings suggestive

of a germline mutation alongside **S1400** to leverage the opportunities outlined above. Specifically, we will administer a one-time survey to a sample of **S1400** patients (n=200) at the master protocol screening step, as well as **S1400** study physicians (n=30) providing care to those patients enrolled in this ancillary study. The ancillary study sample will be enriched for patients from geographically diverse National Cancer Institute Community Oncology Research Program (NCORP) Minority/Underserved (M/U) sites to enhance statistical power to evaluate differences in knowledge, attitudes, and preferences by education level, race/ethnicity, urban/rural status, social support level, and other factors. This exploratory study can provide an initial view of the landscape of knowledge, attitudes, and preferences about genomic profiling in biomarker-driven trials; allow evaluation of heterogeneity in these factors by matched/non-matched status, race/ethnicity, and socioeconomic status; and can be used to generate hypotheses to be evaluated in future biomarker-driven trials.

This ancillary study is important because it will provide timely insight into patient and study physician knowledge, attitudes, and preferences about return of genomic profiling findings in an innovatively designed National Cancer Institute biomarker-driven trial. The results from this ancillary study can inform efforts to improve patient and physician understanding of genomic information in the context of clinical trials, including incidental genomic findings. Additionally, the study's findings can be used to design patient- and physician-centered processes for return of genomic findings in clinical trial settings, and to generate hypotheses about issues related to return of genomic findings in future biomarker-driven clinical trials.

Study Data

The attached preliminary patient and physician surveys (Appendix 1 & 2) include questions that were developed with the input of behavioral science and genomics ethical, legal, and social implications (ELSI) experts to evaluate issues unique to return of genomic results in the context of the **S1400** study. The survey questions were both adapted from prior studies of patient and physician knowledge, attitudes, and preferences about genomic testing in clinical cancer care, and created specifically for the purposes of this study. *(18,19,20,21,22)*

The survey questions evaluate the domains outlined below in bold text.

Knowledge of cancer genomics and return of results in the S1400 trial

- Patient and physician knowledge of the distinctions between somatic and germline mutation testing
- Patient and physician knowledge of the types of genomic results that are returned in the **S1400** trial
- Patient and physician knowledge of the likely outcomes of 'matched' and 'non-matched' treatments

Attitudes about return of genomic results in the S1400 trial

- Patient attitudes about the genomic testing information presented in the **S1400** trial
- A limited number of open-ended questions provide deeper insights into patient attitudes (motivations and concerns) about participating in the **S1400** trial
- Physician attitudes about return of different types of genomic findings in the **S1400** trial

Preferences for return of genomic results in the S1400 trial

- Patient and physician preferences for return of different types of genomic findings in the S1400 trial Patient and physician preferences for return of results to family members in the event of germline mutation findings or somatic mutation findings suggestive of germline mutation
- Patient preferences for return of genomic findings to family members in the event of their death prior to receiving results themselves

The patient survey (Appendix 1) will be reduced and simplified with the input of patients. The physician survey (Appendix 2) will be reduced and simplified with the input of oncology physicians. The survey development process is described below.

First, we will conduct cognitive debriefing interviews, using preliminary drafts of the patient and physician surveys, with local convenience samples of advanced cancer patients, one lung cancer patient advocate (n=5) and oncologists (n=3 physicians). The purpose of this survey cognitive debriefing phase is to test understanding of the survey questions, impacts of alternative phrasing and formatting approaches, and redundancies in question domains. We will aim to recruit a minimum of 4 patients who are not Caucasian/white and/or have a lower educational status (e.g. less than college education) to ensure that we receive early feedback from patients representing these important sub-groups. We will recruit the patient participants for the cognitive debriefing interviews from the Columbia University MU-NCORP site. Study staff at Fred Hutchinson Cancer Research Center will recruit, consent, and conduct the physician cognitive debriefing using oncologists who are members of the Fred Hutch/UW Cancer Consortium. The physicians will complete an online survey and a study team member will conduct an interview with them afterwards to gather their feedback. The Patient and Physician surveys will then be modified according to the feedback received.

Second, we will conduct a quality control check of the modified surveys with 10 **S1400** patients and 3 **S1400** study physicians to evaluate participant ability to complete the surveys and to detect potential issues prior to fielding the final survey. The quality control checks will use methods similar to the actual surveys with patients completing the survey by telephone and physicians completing the survey online.

After completing the cognitive debriefing and quality control check, the investigator team will reevaluate the survey questions, simplify problematic phrasing and/or structure, and reduce the number of survey questions so that it can be completed within targeted periods of time (25-30 minutes for the telephone-based patient survey and 10-15 minutes for the web-based physician survey). We will re-engage our lung cancer patient advocate to assist in this process as needed. There may be a future option for participants to complete the survey online if participants strongly prefer and request that option.

3.0 ELIGIBLITY

All U.S. English language speaking patients registered to the **S1400** master protocol study will be eligible for participation in this ancillary study.

All **S1400** study physicians that have patients who enroll in this ancillary study will be eligible for participation in this ancillary study.

4.0 STRATIFICATION FACTORS

This study will use a stratified recruitment approach to accrue a total sample of 200 patients enriched for representation of patients from NCORP minority-underserved (M/U) sites. The proposed sampling approach will result in the following sub-groups: 1) patients from NCORP non-M/U sites (N=150) and 2) patients from NCORP M/U sites (N=50). This stratified recruitment approach is expected to provide a diverse sample in terms of educational level, race/ethnicity, rural/urban residence, socioeconomic status, social support level, and study site—providing enhanced statistical power to conduct exploratory evaluation of differences in genomic testing knowledge, attitudes, and preferences between sub-groups.

Potential patient participants (i.e. those enrolled in **S1400** master protocol) will provide consent for participation in this ancillary study in an appendix to the **S1400** master protocol screening and pre-screening consent forms. **S1400** site study staff will download the Lung-MAP (**S1400GEN**) Patient Survey and **S1400GEN** Patient Survey Cover Letter from the **S1400** Protocol page on the SWOG website. Study physicians will provide patient participants with a hard copy of the survey (at the time of informed consent) to improve tracking and comprehension during the telephone interview. The surveys will be conducted over the phone. The option to complete the surveys online may be added at a later date if requested by a large number of **S1400GEN** participants. Following informed consent, a trained researcher from Fred Hutchinson Cancer Research Center (Fred Hutch) will contact ancillary study participants (within 30 days of screening/prescreening registration) to conduct the telephone-based survey. The patient survey is targeted to take 25-30 minutes or less to complete. Participants will be mailed a $20 gift card to compensate them for their time within 30 days of completing the telephone interview. Staff at Fred Hutch will be responsible for distributing the survey compensation payments.

Additionally, we will recruit and survey 30 study physicians that are providing care to patients enrolled in the **S1400** ancillary study over an 18-month period beginning with ancillary study activation. This physician-patient matched design will allow us to evaluate associations between physician and patient genomic profiling knowledge, attitudes, and preferences. Fred Hutch staff will email a letter of introduction asking for physician participation, provide a Physician informed consent form, and a link to the online physician survey within 30 days of physicians registering a patient to the patient survey component of this ancillary study. The physician survey is targeted to take 15-20 minutes or less to complete. Physician participants will be sent a $20 gift card in compensation for their time within 30 days of completing the online survey by study staff at Fred Hutch if they provide their contact information at the end of the survey.

5.0 STATISTICAL CONSIDERATIONS

Sample Size

Target accrual has been set by budget to a sample size of 200 patient participants and 30 physician participants. We do not present formal power calculations given that this is an exploratory pilot study intended to generate hypotheses for evaluation in future studies.

Analysis

The primary objective of this pilot study is to conduct an exploratory evaluation of patient attitudes and preferences about return of somatic mutation findings suggestive of a germline mutation in the **S1400** study. Since this study is hypothesis generating, and will include a sample diverse in educational level, race/ethnicity, socio-economic status, urban/rural setting, and social support level we will compare responses between sub-groups defined by these factors and promising items will be examined in a future study. Patient questionnaire responses will be profiled and descriptive statistics will be provided. Additionally, we will conduct unadjusted and multivariate logistic regression analyses to evaluate the association between patient characteristics (e.g. educational level, race/ethnicity, socio-economic status, education, urban/rural setting, and social support level) and attitudes and preferences related to return of genomic findings. Multivariate analyses will adjust for demographic, socioeconomic, and cancer-specific clinical factors, and we will conduct formal tests of multiplicative interaction as appropriate. Similar exploratory analyses will be conducted to evaluate the secondary objectives of patient knowledge about cancer genomics and the design of the **S1400** master protocol trial.

Given the small number of study physicians who will be enrolled (n=30), power to compare responses between groups and to explore associations with patient questionnaire responses will be limited. Physician questionnaire responses about knowledge, attitudes, and preferences about return of genomic findings in S1400 will be profiled and descriptive statistics provided. Correlation between patient and study physician responses will be evaluated using the chi squared statistic.

No formal interim analyses will be conducted in support of this exploratory survey study.

Though the surveys (Appendix 1 and 2) ask only hypothetical questions about incidental genomic findings (i.e. patients are not asked about their specific results), it is possible that some patients may experience stress or concern as a result of participating in the survey. To assess and address this issue, we conclude the patient surveys with a question asking if the questions caused substantial stress or worry, and will advise patients to speak with a member of their healthcare team about any issues of concern.

6.0 ENDPOINTS

6.1 Primary Endpoint

Patient attitudes and preferences about return of somatic mutation findings suggestive of a germline mutation

6.2 Secondary Endpoint

a. Patient knowledge of cancer genomics

b. Physician attitudes and preferences about return of genomic profiling results

c. Physician knowledge of cancer genomics

7.0 BIBLIOGRAPHY

1 Redman MW, Allegra CJ. The Master Protocol Concept. Seminars in oncology. 42(5):724-730, 2015.

2 Herbst RS, Gandara DR, Hirsch FR, et al. Lung Master Protocol (Lung-MAP)-A Biomarker-Driven Protocol for Accelerating Development of Therapies for Squamous Cell Lung Cancer: SWOG S1400. Clinical cancer research: an official journal of the American Association for Cancer Research. 21(7):1514-1524, 2015/

3 Redman MW, Allegra CJ. The Master Protocol Concept. Seminars in oncology. 42(5):724-730, 2015;.

4 Herbst RS, Gandara DR, Hirsch FR, et al. Lung Master Protocol (Lung-MAP)-A Biomarker-Driven Protocol for Accelerating Development of Therapies for Squamous Cell Lung Cancer: SWOG S1400. Clinical cancer research: an official journal of the American Association for Cancer Research. 2015;21(7):1514-1524.

5 Green RC, Berg JS, Grody WW, et al. ACMG recommendations for reporting of incidental findings in clinical exome and genome sequencing. Genetics in medicine: official journal of the American College of Medical Genetics. 15(7):565-574. 2013.

6 Gray SW, Hicks-Courant K, Lathan CS, Garraway L, Park ER, Weeks JC. Attitudes of patients with cancer about personalized medicine and somatic genetic testing. Journal of oncology practice / American Society of Clinical Oncology. 8(6):329-335, 322 p following 335, 2012.

7 Gray SW, Hicks-Courant K, Cronin A, Rollins BJ, Weeks JC. Physicians' attitudes about multiplex tumor genomic testing. Journal of clinical oncology: official journal of the American Society of Clinical Oncology. 32(13):1317-1323, 2014.

8 Miller FA, Hayeems RZ, Bytautas JP, et al. Testing personalized medicine: patient and physician expectations of next-generation genomic sequencing in late-stage cancer care. European journal of human genetics: EJHG. 22(3):391-395, 2014.

9 Blanchette PS, Spreafico A, Miller FA, et al. Genomic testing in cancer: patient knowledge, attitudes, and expectations. Cancer. 120(19):3066-3073, 2014.

10 Middleton A, Morley KI, Bragin E, et al. Attitudes of nearly 7000 health professionals, genomic researchers and publics toward the return of incidental results from sequencing research. European journal of human genetics: EJHG. 2015.

11 Herbst RS, Gandara DR, Hirsch FR, et al. Lung Master Protocol (Lung-MAP)-A Biomarker-Driven Protocol for Accelerating Development of Therapies for Squamous Cell Lung Cancer: SWOG S1400. Clinical cancer research: an official journal of the American Association for Cancer Research. 21(7):1514-1524, 2015.

12 Steuer CE, Papadimitrakopoulou V, Herbst RS, et al. Innovative Clinical Trials: The LUNG-MAP Study. Clinical pharmacology and therapeutics. 97(5):488-491, 2015.

13 Breitkopf CR, Petersen GM, Wolf SM, et al. Preferences Regarding Return of Genomic Results to Relatives of Research Participants, Including after Participant Death: Empirical Results from a Cancer Biobank. The Journal of law, medicine & ethics: a journal of the American Society of Law, Medicine & Ethics. 43(3):464-475, 2015.

14 Wolf SM, Branum R, Koenig BA, et al. Returning a Research Participant's Genomic Results to Relatives: Analysis and Recommendations. The Journal of law, medicine & ethics: a journal of the American Society of Law, Medicine & Ethics. 43(3):440-463, 2015;.

15 Parsons DW, Roy A, Plon SE, Roychowdhury S, Chinnaiyan AM. Clinical tumor sequencing: an incidental casualty of the American College of Medical Genetics and Genomics recommendations for reporting of incidental findings. Journal of clinical oncology: official journal of the American Society of Clinical Oncology. 32(21):2203-2205, 2014;.

16 Raymond VM, Gray SW, Roychowdhury S, et al. Germline Findings in Tumor-Only Sequencing: Points to Consider for Clinicians and Laboratories. Journal of the National Cancer Institute. 108(4), 2016;.

17 Bombard Y, Robson M, Offit K. Revealing the incidentalome when targeting the tumor genome. Jama. 310(8):795-796, 2013.

18 Gray SW, Hicks-Courant K, Lathan CS, Garraway L, Park ER, Weeks JC. Attitudes of patients with cancer about personalized medicine and somatic genetic testing. Journal of oncology practice / American Society of Clinical Oncology. 8(6):329-335, 322 p following 335, 2012.

19 Gray SW, Hicks-Courant K, Cronin A, Rollins BJ, Weeks JC. Physicians' attitudes about multiplex tumor genomic testing. Journal of clinical oncology: official journal of the American Society of Clinical Oncology. 32(13):1317-1323, 2014.

20 Miller FA, Hayeems RZ, Bytautas JP, et al. Testing personalized medicine: patient and physician expectations of next-generation genomic sequencing in late-stage cancer care. European journal of human genetics: EJHG. 22(3):391-395, 2014.

21 Blanchette PS, Spreafico A, Miller FA, et al. Genomic testing in cancer: patient knowledge, attitudes, and expectations. Cancer. 120(19):3066-3073, 2014.

22 Middleton A, Morley KI, Bragin E, et al. Attitudes of nearly 7000 health professionals, genomic researchers and publics toward the return of incidental results from sequencing research. European journal of human genetics: EJHG. 2015.

18.2 List of Sub-study Attachments

S1400A: (Non-Match sub-study): Anti-PD-L1 – MEDI4736

S1400B: PI3K - GBC-0032

S1400C: CDK4/6 – Palbociclib

S1400D: FGFR – AZD4547

S1400E: HGF – Rilotumumab + Erlotinib versus Erlotinib

S1400F: (Non-Match sub-study): Anti-PD-1/PD-L1 Inhibitor Resistant Durvalumab (MEDI4736) plus Tremelumumab

S1400G: HRRD-Talazoparib (BMN 673)

S1400I: (Non-Match sub-study): Nivolumab + Ipilimumab versus Nivolumab

S1400K: C-MET – ABBV-399 (Process II)
