# Supplementary material for: Nivolumab Plus Ipilimumab vs Nivolumab for Previously Treated Patients With Stage IV Squamous Cell Lung Cancer: The Lung-MAP S1400I Phase 3 Randomized Clinical Trial
Source: JAMA Oncol. 2021 Jul 15;7(9):1368–77. doi: 10.1001/jamaoncol.2021.2209 (PMC8283667; doi:10.1001/jamaoncol.2021.2209)
Supplement: Supplement 3. — eTable 1. Gene Alterations Detected on FoundationOne Next-Generation Sequencing Screening eTable 2. Tumor Mutational Burden and PD-L1 Expression eTable 3. Patient Characteristics, Total Population and Population With PD-L1 and TMB Results eTable 4. Cox Model Results Evaluating the Interaction Between TMB as a Continuous Variable and Treatment Arm for the Association With Overall Survival Within PD-L1 Subsets eTable 5. Responses Reported by Treatment Arm and TMB in the Subset of Patients With PD-L1 Expression <1% eTable 6. Patient-Reported-Outcome Common Terminology Criteria for Adverse Events (PRO-CTCAE) by Arm (N, %) eFigure 1. Duration of Response by Treatment Arm eFigure 2. Distribution and Comparison of TMB Levels and PDL1 Expression Levels eFigure 3. Overall Survival Across Levels of TMB in Subgroup With Tumor PD-L1 < 1% eFigure 4. Immune-Related Adverse Events [file jamaoncol-e212209-s003.pdf]

Supplementary Online Content

Gettinger SN, Redman MW, Bazhenova L, et al. Nivolumab plus ipilimumab vs nivolumab for previously treated patients with stage IV squamous cell lung cancer: the Lung-MAP S1400I phase 3 randomized clinical trial. *JAMA Oncol*. Published online July 15, 2021. doi:10.1001/jamaoncol.2021.2209

eTable 1. Gene Alterations Detected on FoundationOne Next-Generation Sequencing Screening

eTable 2. Tumor Mutational Burden and PD-L1 Expression

eTable 3. Patient Characteristics, Total Population and Population With PD-L1 and TMB Results

eTable 4. Cox Model Results Evaluating the Interaction Between TMB as a Continuous Variable and Treatment Arm for the Association With Overall Survival Within PD-L1 Subsets

eTable 5. Responses Reported by Treatment Arm and TMB in the Subset of Patients With PD-L1 Expression < 1%

eTable 6. Patient-Reported-Outcome Common Terminology Criteria for Adverse Events (PRO-CTCAE) by Arm (N, %)

eFigure 1. Duration of Response by Treatment Arm

eFigure 2. Distribution and Comparison of TMB Levels and PDL1 Expression Levels

eFigure 3. Overall Survival Across Levels of TMB in Subgroup With Tumor PD-L1 <1%

eFigure 4. Immune-Related Adverse Events

This supplementary material has been provided by the authors to give readers additional information about their work.

© 2021 Gettinger SN et al. *JAMA Oncology.*

eTable 1. Gene Alterations Detected on FoundationOne Next Generation Sequencing Screening

	**TOTAL (n=252)**	
**Full FMI Panel Alterations**	n	%
**Short Variants**		
TP53	232	92.1
CDKN2A	53	21
MLL2	48	19
PTEN	38	15.1
LRP1B	29	11.5
NFE2L2	28	11.1
ARID1A	21	8.3
PIK3CA	18	7.1
RB1	17	6.7
FBXW7, KRAS, NOTCH1	12	4.8
NF1	10	4.0
TET2	9	3.6
KEAP1	8	3.2
ATRX, KDM6A, STED2	7	2.8
ATM	6	2.4
AKT1, ASXL1, BRCA2, CREBBP, DNMT3A, EP300, STK11	5	2.0
ARID2, BAP1, FANCA, NCOR1, NOTCH2	4	1.6
APC, BCORL1, CASP8, HRAS, KIT, MET, MUTYH, NOTCH3, PBRM1, PIK3R1, PTCH1, SMAD4, SPEN	3	1.2
BRAF, BRCA1, CDC73, CDK12, CHEK2, CHUK, CIC, EGFR, ERBB4, FAM123B, FGFR2, FGFR3, GRIN2A, MEN1, MRE11A, MSH2, NF2, NOTCH4, PIK3CG, PMS2, PRDM1, RUNX1T1, SMARCA4, TRRAP, TSC1, TSC2	2	0.8
AKT3, ATR, BACH1, BCOR, BLM, BRIP1, CDKN1B, CDKN2B, ERBB3, EZH2, FANCC, FANCF, FANCI, FLT3, FLT4, GATA1, GATA3, GNAS, IKZF1, JAK2, KDM5C, KDR, MAP2K4, MAP3K1, MAP3K13, MLH1, MPL, MYD88, NFKBIA, NRAS, NSD1, UP93, PAK3, PALB2, PIK3C2G, PIK3R2, PRKAR1A, RAD50, RET, RNF43, SF3B1, SMARCB1, SMARCD1, SMO, STAG2, STAT4, SUFU, VHL	1	0.4
Copy Number Alterations		
SOX2	83	32.9
PIK3CA	54	21.4

© 2021 Gettinger SN et al. *JAMA Oncology.*

FGF12	45	17.9
CDKN2A	35	13.9
CDKN2B, FGFR1	28	11.1
FGF3, FGF4	26	10.3
CCND1, FGF19	25	9.9
ZNF703	23	9.1
EGFR, MYST3	14	5.6
CCND2, MYC, PTEN	12	4.8
AKT2	11	4.4
CRKL, FGF23, FGF6, KDM5A	10	4.0
KRAS	9	3.6
CCNE1, MYCL1	8	3.2
FGF10, MDM2, REL, RICTOR	7	2.8
ERBB2, LRP1B, NFKBIA	6	2.4
BCL2L2, IRS2, KIT, NKX2-1	5	2.0
CDK6, RB1	4	1.6
AXL, EPHA3, HGF, KDM6A, KDR, TP53	3	1.2
ARID1A, EPHB1, ERBB3, FGF14, JAK2, MCL1, MYCN, PDGFRA, SMAD4, SRC, TOP1,	2	0.8
ALK, BRAF, BRCA2, CCND3, CDK4, CIC, CREBBP, ERBB4, FBXW7, FGFR4, IGF1R, IGF2, IKBKE, MAP2K2, MDM4, NF1, NOTCH3, PBRM1, PIK3CG, RET, RPTOR, SUFU, ZNF217	1	0.4
Rearrangements		
LRP1B	3	1.2
CDK12, CDKN2A, CREBBP, FGFR3, MAP3K13, NOTCH2	2	0.8
ARID1A, ARID2, BACH1, BAP1, CIC, CTNNA1, CTNNB1, FAM123B, FANCF, FBXW7, FGFR1, KDM6A, MLL2, MSH6, NFKBIA, NOTCH3, PAX5, SMAD4, SMARCA4, STK11, TET2, TNFAIP3, VHL	1	0.4

© 2021 Gettinger SN et al. *JAMA Oncology.*

eTable 2. Tumor Mutational Burden and PD-L1 Expression

	All Patients (N=252)	Nivolumab/ Ipilimumab Arm (N=125)	Nivolumab Arm (N=127)	
**PD-L1 Expression**				
0%	63 (39%)	27 (36%)	36 (42%)	
1-4%	28 (17%)	10 (13%)	18 (21%)	
5-49%	32 (20%)	17 (22%)	15 (18%)	
≥50%	38 (24%)	22 (29%)	16 (19%)	
Not done	91 (36%)	49 (39%)	42 (33%)	
**FMI TMB Score**				
Median	10.9	10.9	10.9	
Range	2.2-67.1	2.4-67.1	2.2-35.1	
Interquartile range	7.7-15.7	7.5-15.7	7.7-16.5	
≥10	128 (55%)	66 (57%)	62 (54%)	
Not determinable	21 (8%)	9 (7%)	12 (9%)	

© 2021 Gettinger SN et al. *JAMA Oncology.*

eTable 3. Patient Characteristics, Total Population and Population With PD-L1 and TMB Results

	**TOTAL (n=252)**		**With PD-L1 AND TMB Results (n=149)**	
AGE				
Median	67.5		67.5	
Minimum	41.8		41.8	
Maximum	90.3		84.1	
SEX				
Males	169	67%	100	67%
Females	83	33%	49	33%
HISPANIC				
Yes	3	1%	1	1%
No	246	98%	147	99%
Unknown	3	1%	1	1%
RACE				
White	206	82%	121	81%
Black	33	13%	21	14%
Asian	4	2%	1	1%
Pacific Islander	1	0.4%	1	1%
Native American	3	1%	3	2%
Multi-Racial	1	0.4%	0	0%
Unknown	4	2%	2	1%
NUMBER OF PRIOR THERAPIES				
One	211	84%	127	85%
Two or more	41	16%	22	15%
NUMBER OF PRIOR SYSTEMIC THERAPIES FOR STAGE IV DISEASE				
0	70	28%	53	36%

© 2021 Gettinger SN et al. *JAMA Oncology.*

1	150	60%	80	54%
2	13	5%	6	4%
3 or more	4	1%	1	1%
Not asked	15	6%	9	6%
PERFORMANCE STATUS				
0	71	28%	43	29%
1	181	72%	106	71%
WEIGHT LOSS PAST 6 MONTHS				
< 5%	179	71%	104	70%
5 - < 10%	43	17%	25	17%
10 - < 20%	26	10%	17	11%
>=20%	4	2%	3	2%
SMOKING STATUS				
Current Smoker	102	40%	56	38%
Former Smoker	147	58%	93	62%
Never Smoker	3	1%	0	0%
BRAIN METASTASES				
Yes	20	8%	10	7%
No	232	92%	139	93%
LIVER METASTASES				
Yes	53	21%	26	17%
No	199	79%	123	83%
LOCALIZED PALLIATIVE RADIATION THERAPY				
Yes	85	34%	58	39%
No	167	66%	91	61%
RADIATION THERAPY WITH CURATIVE INTENT				
Yes	86	34%	52	35%
No	166	66%	97	65%

© 2021 Gettinger SN et al. *JAMA Oncology.*

eTable 4. Cox Model Results Evaluating the Interaction Between TMB as a Continuous Variable and Treatment Arm for the Association With Overall Survival Within PD-L1 Subsets

PD-L1 Subset	N (events)	P-value for Interaction between treatment and continuous TMB
0%	56 (47)	**0.06**
1-4%	28 (23)	0.71
5-49%	30 (25)	0.65
50+%	35 (27)	0.71

eTable 5. Responses Reported by Treatment Arm and TMB in the Subset of Patients With PD-L1 Expression < 1%

n/N, response rate (95% CI)	TMB	
	< 10	≥10
Nivolumab Arm	2/17, 12% (0% - 27%)	2/15, 13% (0% - 31%)
Nivolumab+Ipilimumab Arm	1/9, 11% (0% - 32%)	4/15, 27% (4% - 49%)

© 2021 Gettinger SN et al. *JAMA Oncology.*

eTable 6. Patient-Reported-Outcome Common Terminology Criteria for Adverse Events (PRO-CTCAE) by Arm (N, %)

	**Score >0**			**Score ≥3**		
PRO-CTCAE[1]	Nivolumab (N=71)	Nivolumab+ Ipilimumab (N=75)	p-value[2]	Nivolumab (N=71)	Nivolumab+ Ipilimumab (N=75)	p-value[2]
**Without Baseline Adjustment**						
Diarrhea frequency[3]	52 (73.2)	53 (70.7)	.85	14 (19.7)	10 (13.3)	.37
Itching severity[4]	50 (70.4)	54 (72.0)	.86	10 (14.1)	14 (18.7)	.51
Fatigue severity[4]	69 (97.2)	72 (96.0)	1.0	28 (39.4)	36 (48.0)	.32
Fatigue interference[5]	66 (93.0)	70 (93.3)	1.0	34 (47.9)	47 (62.7)	.10
**With Baseline Adjustment**						
Diarrhea frequency[3]	37 (52.1)	39 (52.0)	1.0	12 (16.9)	10 (13.3)	.64
Itching severity[4]	41 (57.8)	43 (57.3)	1.0	9 (12.7)	11 (14.7)	.81
Fatigue severity[4]	42 (59.2)	50 (66.7)	.39	22 (31.0)	29 (38.7)	.39
Fatigue interference[5]	46 (64.8)	47 (62.7)	.86	28 (39.4)	31 (41.3)	.87

[1] Recall period is the last 7 days.

[2] Determined by use of the Fisher exact test.

[3] Frequency defined as: 0=none; 1=rarely; 2=occasionally; 3=frequently; 4=almost constantly.

[4] Severity defined as: 0=none; 1=mild; 2=moderate; 3=severe; 4=very severe.

[5] Interference with daily activities defined as: 0=not at all; 1=a little bit; 2=somewhat; 3=quite a bit; 4=very much.

© 2021 Gettinger SN et al. *JAMA Oncology.*

eFigure 1. Duration of Response by Treatment Arm

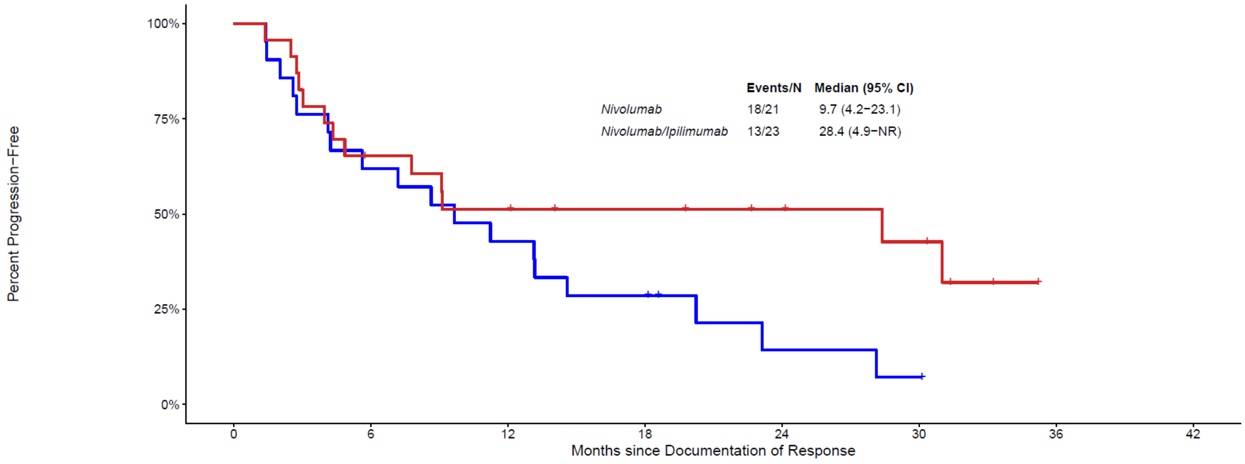

Number at risk (number of events)

Nivolumab Arm	21 (0)	13 (8)	9 (12)	6 (15)	2 (17)	1 (18)	0 (18)	0 (18)
Nivolumab/Ipilimumab Arm	23 (0)	14 (8)	11 (11)	9 (11)	7 (11)	5 (12)	0 (13)	0 (13)

© 2021 Gettinger SN et al. *JAMA Oncology.*

eFigure 2. Distribution and Comparison of TMB Levels and PDL1 Expression Levels

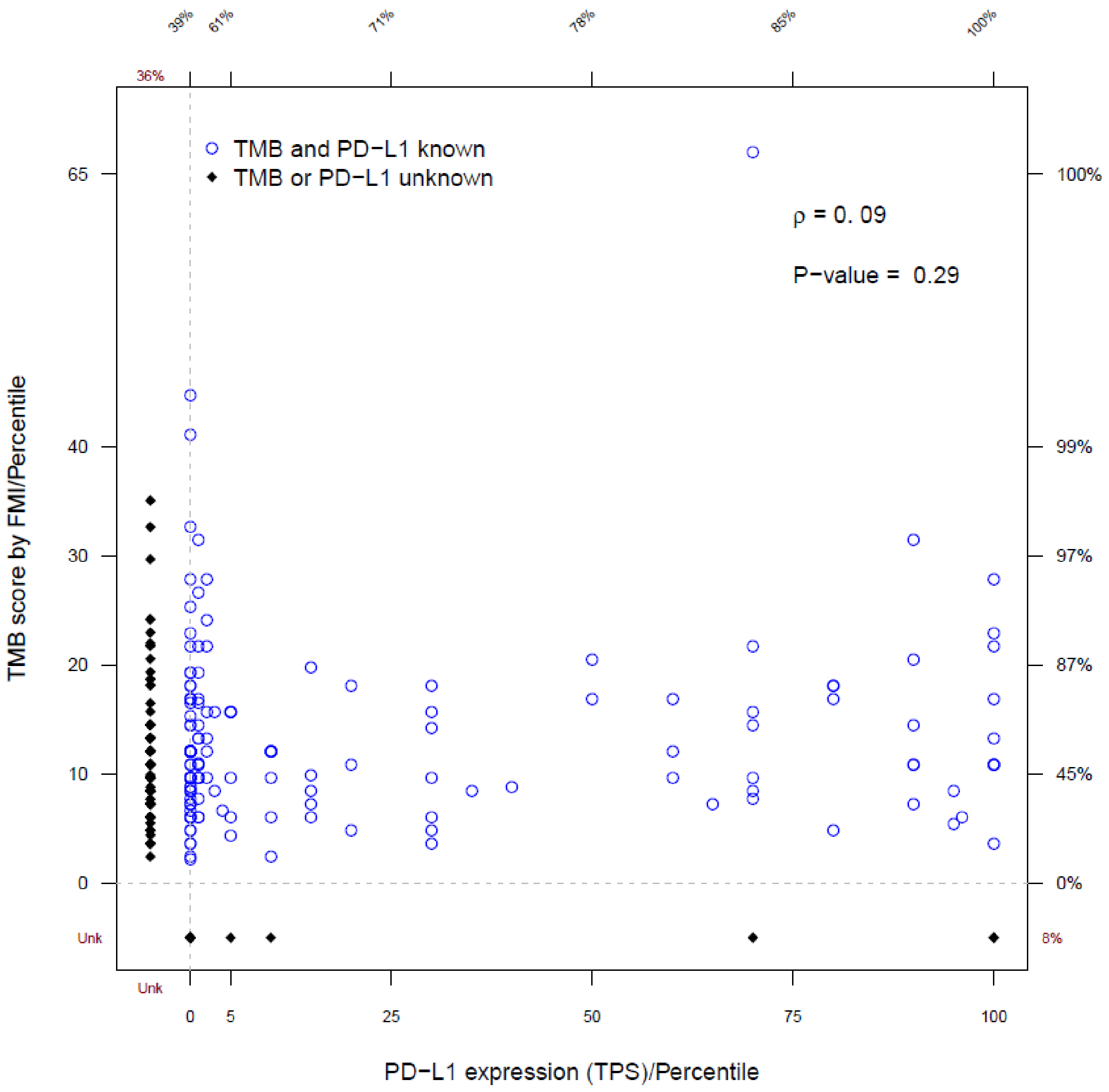

© 2021 Gettinger SN et al. *JAMA Oncology.*

eFigure 3. Overall Survival Across Levels of TMB in Subgroup With Tumor PD-L1 <1%

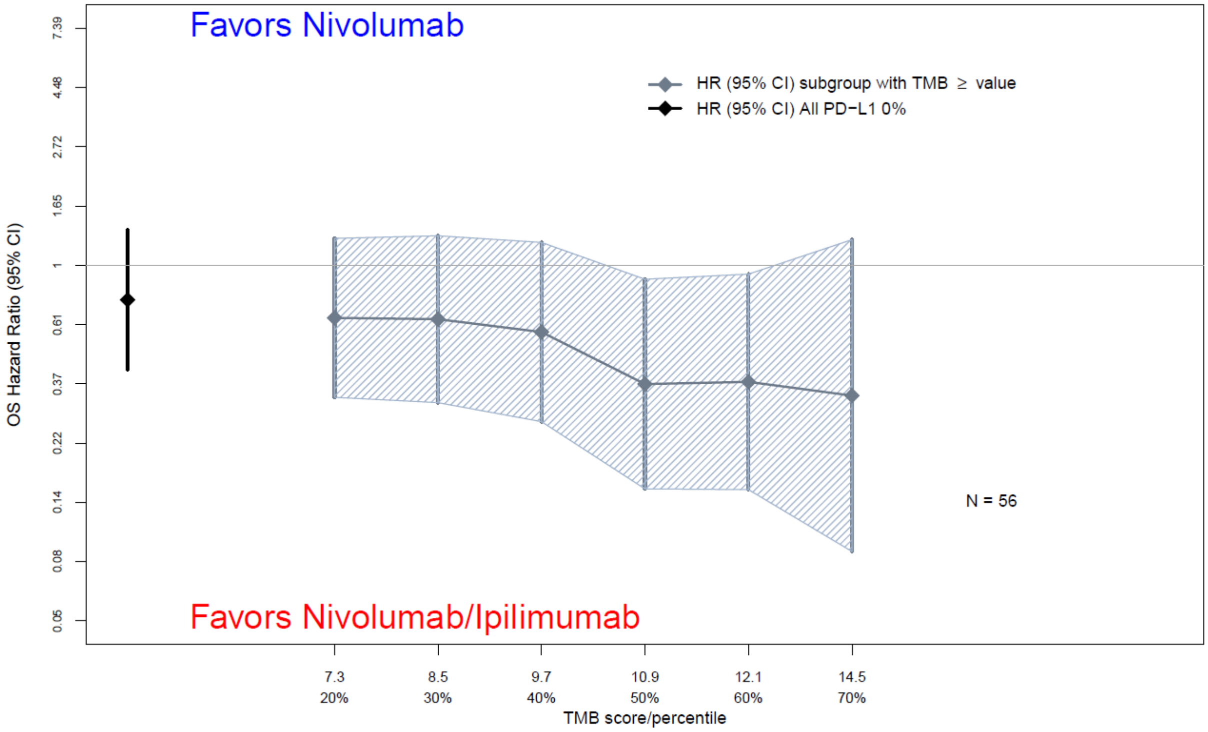

© 2021 Gettinger SN et al. *JAMA Oncology.*

eFigure 4. Immune-Related Adverse Events

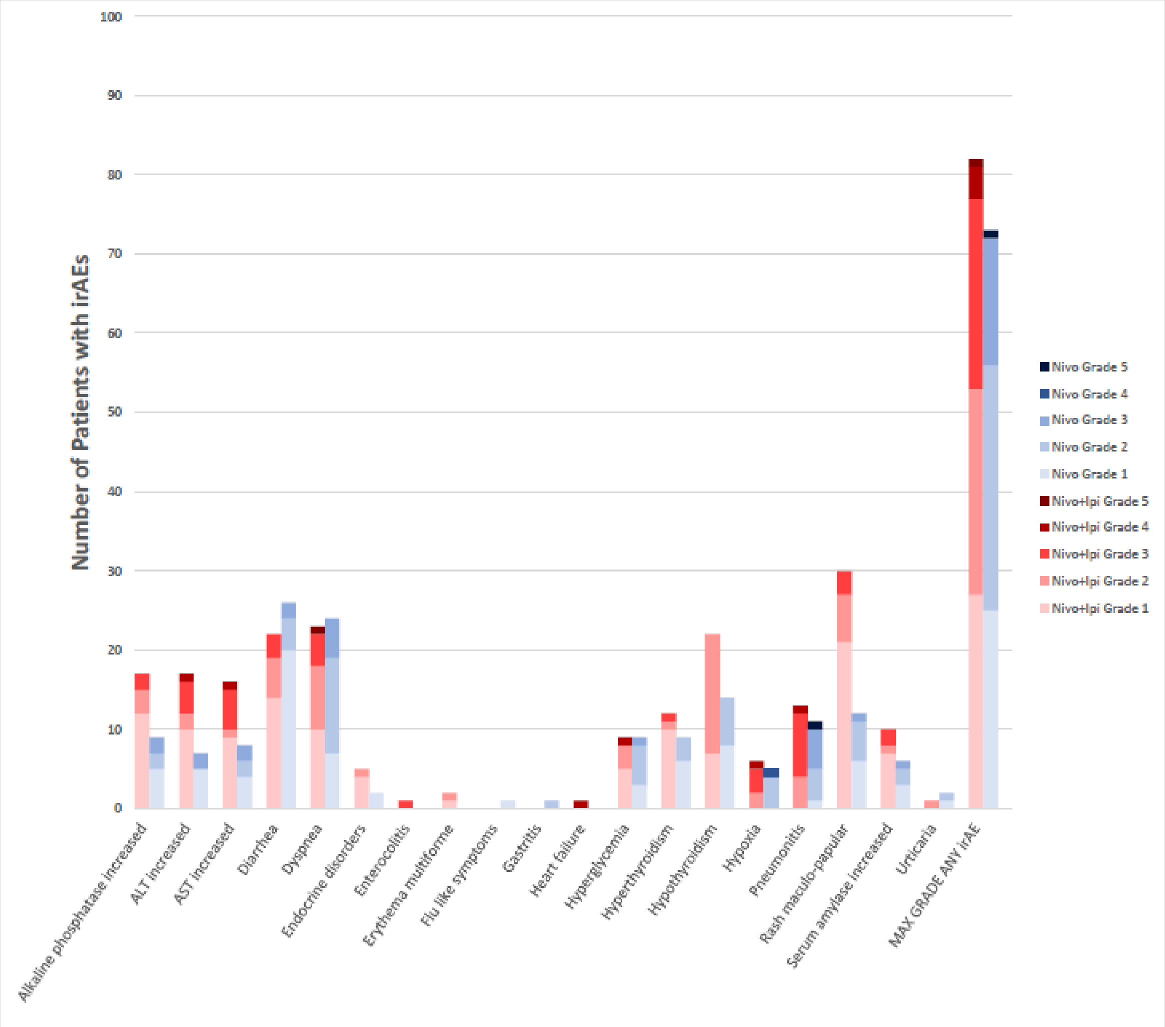

© 2021 Gettinger SN et al. *JAMA Oncology.*
