# Supplementary material for: Nivolumab Plus Ipilimumab vs Nivolumab for Previously Treated Patients With Stage IV Squamous Cell Lung Cancer: The Lung-MAP S1400I Phase 3 Randomized Clinical Trial
Source: JAMA Oncol. 2021 Jul 15;7(9):1368–77. doi: 10.1001/jamaoncol.2021.2209 (PMC8283667; doi:10.1001/jamaoncol.2021.2209)
Supplement: Supplement 4. — Data Sharing Statement [file jamaoncol-e212209-s004.pdf]

Data Sharing Statement

Gettinger. Nivolumab Plus Ipilimumab vs Nivolumab for Previously Treated Patients With Stage IV Squamous Cell Lung Cancer. *JAMA Oncol*. Published July 15, 2021. doi:10.1001/jamaoncol.2021.2209

Data
Data available: Yes
Data types: Deidentified participant data, Data dictionary
How to access data: National Clinical Trials Network (NCTN) Data Archives
When available: With publication

Supporting Documents
Document types: None

Additional Information
Who can access the data: The National Cancer Institute's (NCI) NCTN/NCORP Data Archive is a centralized, controlled-access database for sharing datasets generated from clinical trials of the National Clinical Trials Network (NCTN) and the NCI Community Oncology Research Program (NCORP). These datasets are made available on appropriate terms and conditions to researchers who wish to analyze the data in secondary studies to enhance the public health benefit of the original work.
Types of analyses: The National Cancer Institute's (NCI) NCTN/NCORP Data Archive is a centralized, controlled-access database for sharing datasets generated from clinical trials of the National Clinical Trials Network (NCTN) and the NCI Community Oncology Research Program (NCORP). These datasets are made available on appropriate terms and conditions to researchers who wish to analyze the data in secondary studies to enhance the public health benefit of the original work.
Mechanisms of data availability: The National Cancer Institute's (NCI) NCTN/NCORP Data Archive is a centralized, controlled-access database for sharing datasets generated from clinical trials of the National Clinical Trials Network (NCTN) and the NCI Community Oncology Research Program (NCORP). These datasets are made available on appropriate terms and conditions to researchers who wish to analyze the data in secondary studies to enhance the public health benefit of the original work.
